# Supplementary material for: A NANOG‐pERK reciprocal regulatory circuit regulates Nanog autoregulation and ERK signaling dynamics
Source: EMBO Rep. 2022 Sep 6;23(11):e54421. doi: 10.15252/embr.202154421 (PMC9638859; doi:10.15252/embr.202154421)
Supplement: Supplementary file 1 — Appendix [file EMBR-23-e54421-s010.pdf]

A NANOG-pERK reciprocal regulatory circuit mediates *Nanog* autoregulation and ERK signaling dynamics.

Hanuman T. Kale, Rajendra Singh Rajpurohit, Debabrata Jana, Vijay V. Vishnu, Mansi Srivastava, Preeti R. Mourya, Gunda Srinivas, P. Chandra Shekar*

Table of Contents

Appendix Figure S1 – page 2

Appendix Figure S2 – page 3

Appendix Table S1. Oligonucleotide sequences used in this study – page4

Appendix Table S2. Reagents used in this study- page 7

Appendix Figure S3 -Myoplasma analysis- page 10

Appendix Figure S4 - STR analysis- page 11

Appendix Table S3. Oligonucleotide sequences used for STR analysis- page 12

Appendix references -page 13

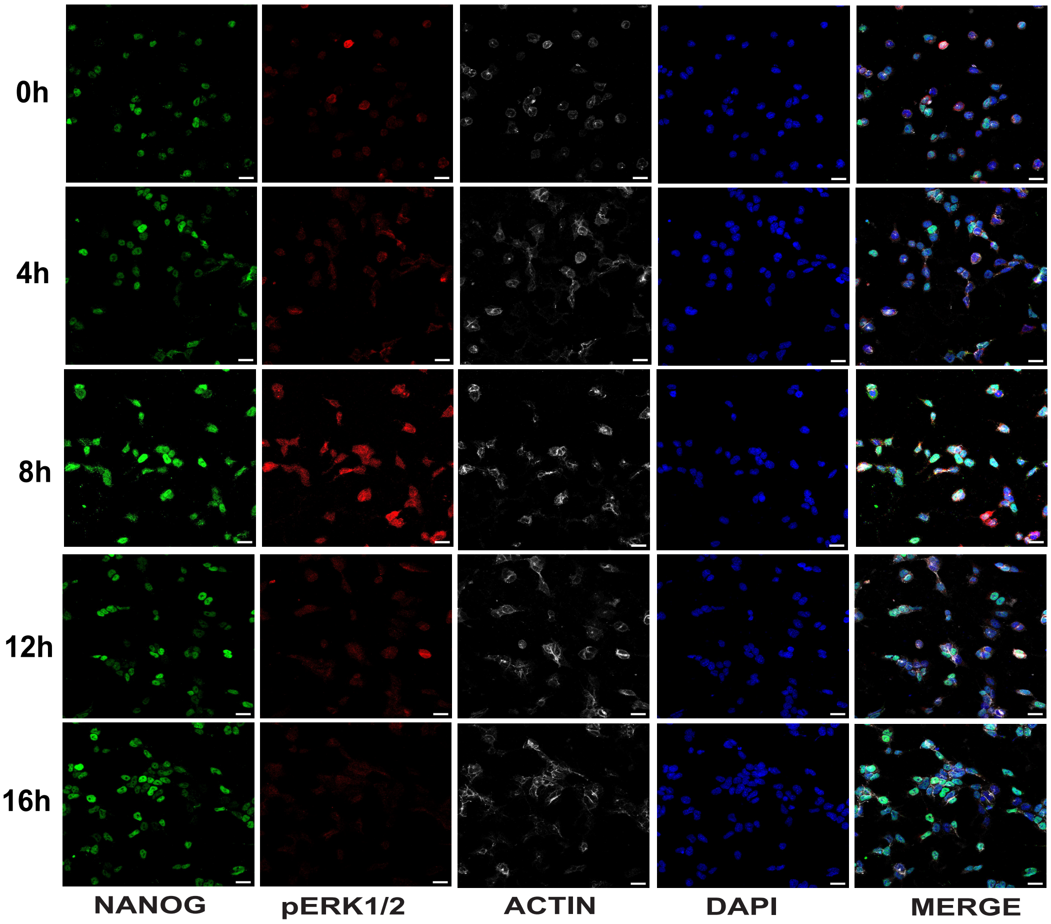

Appendix Figure S1. Data related to Fig 6.

NANOG regulates ERK signaling dynamics and heterogeneity.

Immunocytochemistry at different time intervals of culture of NiRFP2A ESCs after sorting of 10% of Nanog-high cells. pERK (red), NANOG (green) and nuclear stain (blue). pERK and NANOG were detected in 10% Nanog-high cells immediately after sorting. Most of the cells expressed NANOG and few of these cells expressed pERK also. pERK showed very high heterogeneity at 0 hrs. pERK expression increased gradually with further increase in NANOG until 8 hrs. pERK1//2 was highest at 8 hrs with concomitant high expression of NANOG. Most of the cells expressed pERK by 8 hrs. pERK expression decrease after 8 hrs coinciding with decrease of NANOG. few cells expressed pERK by 12 hrs. The pERK expression further decreased by 16hrs followed by decrease in NANOG expressing cells. Scale bar 25 µM.

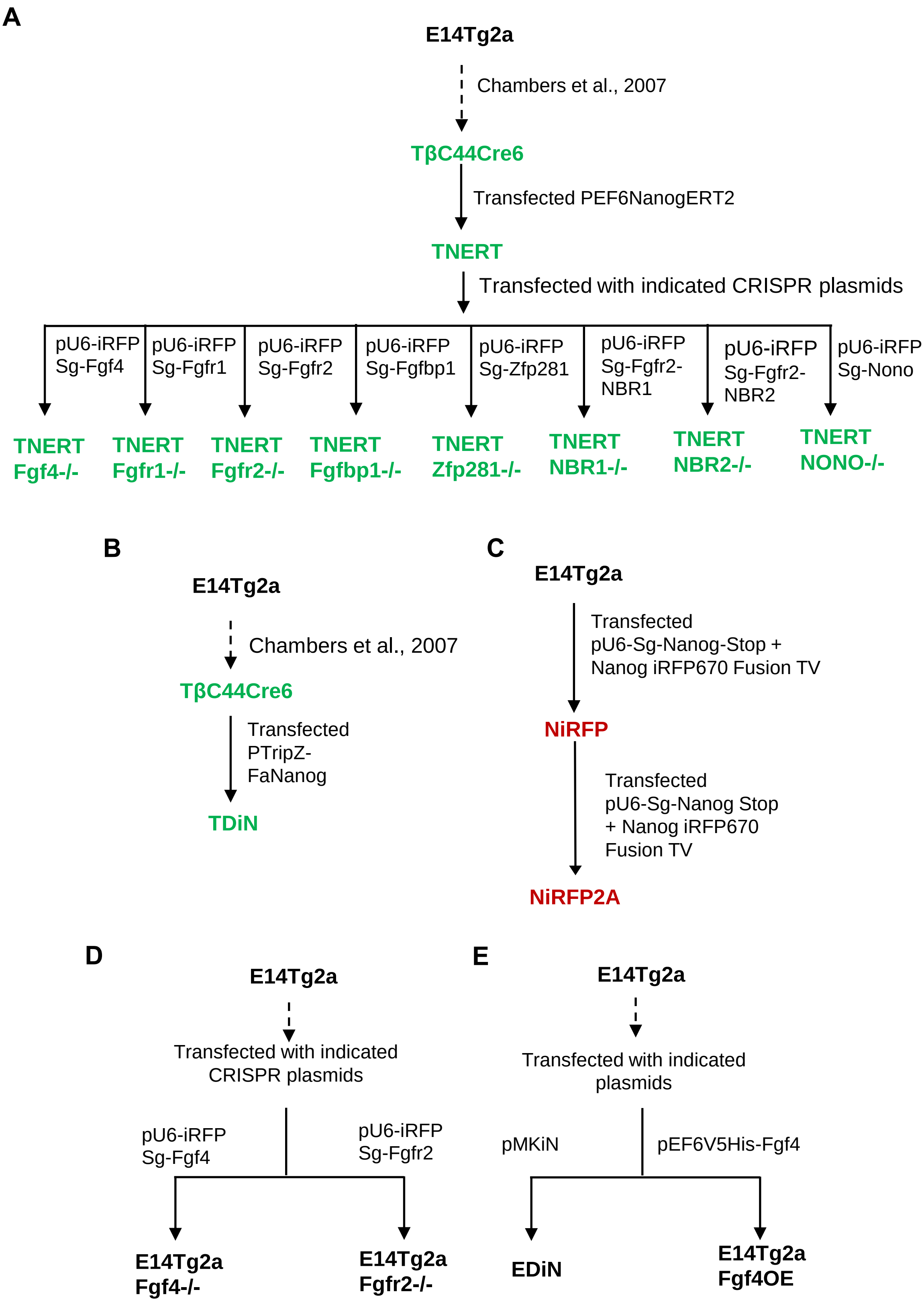

Appendix Figure S2: Information related to methods. A pedigree chart of cell lines used in this study

(A) Flow chart illustrating the lineage and process of generation of TNERT and generation of knock out cells lines in TNERT background. (B) A flow chart describing derivation of TDiN. (C) A flow chart depicting derivation of NiRFP2A from E14Tg2a. (D) A flow chart depicting derivation of Fgf4-/- and Fgfr2-/- ES cell lines from E14Tg2a. (E) A flowchart depicting derivation of EDiN and Fgf4OE (over expression) ES cell lines from E14Tg2a.

Appendix Table S1. Oligonucleotide sequences used in this study.

Name of the Oligo	Sequence
**Oligos for paired sgRNA Knock-out**	
Fgf4 sgRNA1	CACCGCCGTGCGTGAGTTCGAGCTG
	AAACCAGCTCGAACTCACGCACGGG
Fgf4 sgRNA2	CACCGCGAAACGCGGCCGACCAC
	AAACGTGGTCGGCCCGCGTTTCGC
Fgfr1 sgRNA1	CACCGCGTCATCATCTTCCGAGGAT
	AAACATCCTCGGAAGATGATGACGC
Fgfr1 sgRNA2	CACCGTTCTCTGGGGATGTCCAGTA
	AAACTACTGGACATCCCCAGAGAAC
Fgfr2 sgRNA1	CACCGCTGGGGGCGCTTCATCTGCC
	AAACGGCAGATGAAGCGCCCCCAGG
Fgfr2 sgRNA2	CACCGCTCAGTGTAAGTAGGTTCC
	AAACGGAACCTACTTACACTGAGC
Fgfbp1 sgRNA1	CACCGCACAGTCTTGGCCCACATTA
	AAACTAATGTGGGCCAAGACTGTGC
Fgfbp1 sgRNA2	CACCGATGTCGCCTGTAACATGTTG
	AAACCAACATGTTACAGGCGACATC
Fgfr2 NBR1 sgRNA1	CACCGTCGGAGCAGCTAGGCGAACT
	AAACAGTTCGCCTAGCTGCTCCGAC
Fgfr2 NBR1 sgRNA2	CACCGTTGTGTTTAGGGCCCCCCCT
	AAACAGGGGGGGCCCTAAACACAAC
Fgfr2 NBR2 sgRNA1	CACCGTGGAAGCAGCGGATGTTCGT
	AAACACGAACATCCGCTGCTTCCAC
Fgfr2 NBR2 sgRNA2	CACCGACCGGAGCTGCTCTCGGATC
	AAACGATCCGAGAGCAGCTCCGGTC
Nanog -stop codon sgRNA	CACCGTATGAGACTTACGCAACATC
	AAACGATGTTGCGTAAGTCTCATAG
Zfp281 sgRNA1	CACCGAGGCCTGGCTGCGGAGAGG
	AAACCCTCTCCGCAGCCAGGCCTC
Zfp281 sgRNA1	CACCGCGGGGTATGAAAATCGGCAG
	AAACCTGCCGATTTTCATACCCCGG
Nono sgRNA1	CACCGTTTTAATGATGGGTACCATC
	AAACGATGGTACCCATCATTAAAAC
Nono sgRNA1	CACCGTTCCGAGAGAGCGTCAAGAC
	AAACGTCTTGACGCTCTCTCGGAAC
**Genotyping primers**	
Fgf4 knock-out	CGCAGCACTCACCGAACTCA
	TGCCCACGTTGCAGTAGAGC
Fgfr1 knock-out	GTCTAGACAGGGCGAATGCTGTTT
	ACTTGAACTTCACCGTCTTGGCAG
Fgfr2 knock-out	TCTGGGTTTAAGCAAGTTGGCACT
	CAGCATACATGGTGGGTCAGAGAG
Fgfbp1 knock-out	GAAAGTGAGAAGCTGAGTGAATGG
	TATATGCACCTAGGTTTGTGGTCC
Fgfr2 NBR1 knock-out	GAAGAAACTGCTGGAGTGTGGTCA
	AGGGTAGTTCCAGGATACCTCAGC
Fgfr2 NBR2 knock-out	AGAGGCTTTGGATGACTCTGCAAC
	GCGATGATCTCGGAGGAAAACTCC

Zfp281 knock-out	TGTGGAGAGGACGGCGTTATTTTT
	TGAAACCCATACTACACTGGCTGC
Nono Knock out	AGGTTTCCCTGCTTGTCTTTGTCT
	GCAGCAACGCCCTTAATTTCAACA
Nanog-iRFP670	ACCCAGGGGTGACAAAGTATTCCAA
	GCATTTTCCGTAATGCGCGTGATCC
Nanog-sfGFP	ACCCAGGGGTGACAAAGTATTCCAA
	CGTTTGTAGCATCACCTTCACCCTC
Nanog-T2AmCherry	ACCCAGGGGTGACAAAGTATTCCAA
	TCGCCCTTGCTCACCATTGGCCCGGGATTCTCTTC
**q-RT-PCR primers**	
	CTGCCATTAATGTGGCCATCCC
Dusp6	GTGTTCTCATTCCAGTCGCTGC
	TGGTCCCCACAGTTTGCCTAGTTC
Nanog	CAGGTCTTCAGAGGAAGGGCGA
	GTGGAGGAAGCCGACAACAATGA
Oct4	CAAGCTGATTGGCGATGTGAG
	TTTTCTAGTCGGCATCACCG
Sox2	ACAAGAGAATTGGGAGGGGT
	GTGCAGCTTGCAGCAGTAAC
Klf4	AGCGAGTTGGAAAGGATAAAGTC
	CAGTCCAGAATACCAGAGTGGAA
Rex1	ACTCTAGGTATCCGTCAGGGAAG
	CCCTGCGGAGACAGGTAACAG
Fgfr2	AGCGTCAGCTTATCTCTGGGGA
	ACCCTGTAGCTCCCTACTGGAC
Fgfr1	TGGCATAGCGAACCTTGTAGCC
	ACACGAGGGACAGTCTTCTGGA
Fgf4	TAGGCGTTGTAGTTGTTGGGCA
	CTAACCTCAAGCTGGTGAACCCC
Fgfbp1	TCTCTAATGGCCATGGTCTGGGT
	CAACGGCACAGTCAAGGCCGA
Gapdh	CCCTTCAAGTGGGCCCCGG
	CCACCGATTGCTTGGAAGTTGG
Spry2	AGGTCTTGGCAGTGTGTTCACC
	AGCCCAGTACTCAGGCTTGT
Pre-*Nanog-1*	AGCATCACAACACGCACCT
	GCCAGCAGATGGCATAATTT
Pre-*Nanog-2*	TGATGGCAATGCTGAGGTTA
	GTCCCAGCTGGTGTGACTCT
Pre-*Oct4*	TCTTCTGCTTCAGCAGCTTG
	GACTACCTGCTGGGCCTCAAAA
Pre-*Fgf4*	TACTCAGCCCCCGAGACTACTAC
	CCCGTCCCTTCCCAAATCTGATA
Pre-*Fgfr2*	TCACCTTGGGTCAGGATAACAAG
	GACTACCTGCTGGGCCTCAAAA
Pre-*Fgfbp1*	TACTCAGCCCCCGAGACTACTAC
Pre-*Fgfr1*	CCCGTCCCTTCCCAAATCTGATA
	TCACCTTGGGTCAGGATAACAAG

5'UTR Of Nanog	GGTGATACGTTGGCCTTCTAGT
	TTCTCAAATACACACAAGAGCCTTA
**q-ChIP-PCR primers**	
Nanog 5.5 kb	GTGGGTGCACACAGAGAACAAC
	CTGAGAGCTCAGGCCCACAAAG
Nanog -4.9 kb	AACATTCCTTTCCCCACCCACA
	AAGAGGTGGCTGGTAGCCAAAA
Nanog -4.7 kb	TGGGGTAAACTTAAGGCTATGG
	AGCTCTAAGCCGGTTCTCATTT
Nanog -3.9 kb	CCCTACCTCTCCTGAGGTGTGA
	CATGCCTGAGGAAGTCAGAGGA
Nanog -3.4 kb	TGTAGCCCTTGGTTAGTCCGAG
	GGCAGGCATCACCAAAGTCATT
Nanog -1.9 kb	GGTTCAGTCAGGCTGGGCAAT
	CTGCTGCCACACTATCACTGTC
Nanog -1.0 kb	AGCCGACTTAAGCTGGGTTAGA
	TGCTCTAGCTGGTCCCAAACTC
Nanog -0.05 kb	TAGGGTAGGAGGCTTGAGGGG
	AAGTCAGAAGGAAGTGAGCCGC
Nanog +.5 kb	CCGGTGATACGTTGGCCTTCTA
	ACTGCCCCCGAACATATTCCAA
Nanog +1.4 kb	GTTAGGAATGAACGGGTGGGGA
	AGTAGACAGCCCTGAAAGCAGC
Fgf4 +3.5 kb	GCCCAGAACCCAATTTTTATGCAC
	CAAAGTCCCAGAGCCATTCCCTT
Fgf4 -3.0 kb	TTAGCTCGCTTCAGGGAATGCTT
	TTGCTGTCTGTAGCCTCCCATAA
Fgf4 -6.5 kb	CACAAAGGTCGCTTAAGTGGTGG
	ACACGATTTCCAGACTCCTCCAG
Fgf4 -6.9 kb	TTAGGCACCCAAAGGCAGAATTG
	GTCCTGTTATTCATGGCAGGGGA
Fgfr1-5kb	TGGCCTTGGATGAATTGTTGGC
	TTCCACCTCCCTTCAGGACACT
Fgfr1+2.5 kb	TGGGGTGGTGTCTCTTCCTTTCAG
	CAAGCCATTAGGGAGGGAGGCAA
Fgfbp1-1.4 kb	TCCAGTGTGTGTGGTAAACAGGT
	AACACTGCCTCTGGATGGTCTAC
Fgfr2 -2.5 kb	TTTTGTCCCACTTCTTGGGGC
	AATCTTCCACCAGCCTGGACTC
Fgfr2 -4.4 kb	AAACAACGTAACGCATCCACTGT
	TGCACAGATGACCTCTCGGAAC
Oct4-0.3kb	TCTCCAGAGGATGGCTGAGTG
	CCAGGAGGCCTTCATTTTCAAC
HoxC11+0.13kb	CACCGTCTCTTCCTTCCTACCC
	GACGAGTAGCTGTTCCGATGGT
OFR-Chr6(43023477..43023592)	TGTGTGTTTGTGCACCGTGTGTGA
	TAGGTCCAATTCCCACCACCAGCAT

Appendix Table S2: Reagents used in this study

REAGENT or RESOURCE	SOURCE	IDENTIFIER
Antibodies		
Anti-NANOG	Thermo Fisher Scientific	Cat# 14-5761-80, RRID: AB_763613
Anti-NANOG	Cell signaling Technology	Cat# 8822, RRID: AB_11217637
Nanog Polyclonal Antibody	Thermo Fisher Scientific	Cat# PA5-47376, RRID: AB_2607022
Anti-OCT3/4	Cell signaling Technology	Cat# 83932, RRID: AB_2721046
Anti-OCT3/4	Thermo Fisher Scientific	Cat# 14-5841-82, RRID: AB_914301
Anti-SOX 2	Cell signaling Technology	Cat# AMAb91307, RRID: AB_2665892
Anti-FGFR2	Thermo Fisher Scientific	Cat# PA1-24763, RRID: AB_780623
Anti-FGFR2	R&D Systems	Cat# MAB6843, RRID: AB_2103395
Anti-FGFR1	Cell signaling Technology	Cat# 9740, RRID: AB_11178519
FGFBP1 Polyclonal Antibody	Thermo Fisher Scientific	Cat# PA5-77220, RRID: AB_2720947
Anti-FGF4	Thermo Fisher Scientific	Cat# PA5-20483, RRID: AB_11152903
Anti-ERK	Cell signaling Technology	Cat# 9102, RRID: AB_330744
Anti-P-ERK	Cell signaling Technology	Cat# 4370, RRID: AB_2315112
Anti-P-ERK	Cell signaling Technology	Cat#9101, RRID: AB_331646
Anti-Trimethyl Histone H3(Lys4) (C42D8)	Cell signaling Technology	Cat# 9751, RRID: AB_2616028)
Anti-Trimethyl Histone H3(Lys27)	Merck Millipore	Cat# 07-449, RRID: AB_310624
Anti- β-ACTIN	Sigma-Aldrich	Cat# A2228, RRID: AB_476697
Anti-HDAC2	Thermo Fisher Scientific	Cat# 51-5100; RRID: AB_2533908
Anti-RNA polymerase II Antibody, clone CTD4H8	Merck Millipore	Cat# 05-623, RRID: AB_309852
Nanog (eBioMLC-51)	Thermo Fisher Scientific	Cat#14-5761-80 RRID:AB_763613
Oct4	Thermo Fisher Scientific	Cat#14-5841-37RRID AB_2865147
Chemicals, Peptides, and Recombinant Proteins		
rhFGF4	R&D Systems	Cat# 7460-F4-025
rhFGFBP1	R&D Systems	Cat# 1593-FB-025
Human FGF4 recombinant protein	Thermo Fisher Scientific	Cat# PHG0154
Human BMP4 recombinant protein	Thermo Fisher Scientific	Cat# PHC9534
Human EGF recombinant protein	Thermo Fisher Scientific	Cat# 01-107

Human Insulin recombinant protein	Thermo Fisher Scientific	Cat# RP-10908
Human bFGF recombinant protein	Thermo Fisher Scientific	Cat# RP-8628
G418 disulfate salt	Sigma-Aldrich	Cat# A1720
Doxycycline Hyclate	Sigma-Aldrich	Cat# D9891
SU5402	Sigma-Aldrich	Cat# SML0443
PD0325901	Sigma-Aldrich	Cat# PZ0162
CHIR99021	Sigma-Aldrich	Cat# SML1046
(Z)-4-Hydroxytamoxifen	Sigma-Aldrich	Cat# H7904
LIF	Made in-house	
Heparan sulfate sodium salt	Sigma-Aldrich	Cat# H7640
o-Phenylenediamine dihydrochloride	Sigma-Aldrich	Cat# P8287
Deposited Data		
Experimental Models: Cell Lines		
E14Tg2a		Chambers et al., 2007
TNGA		Chambers et al., 2007
TβC44Cre6		Chambers et al., 2007
TNERT	This Study	
NiRFP2A	This Study	
TDiN	This Study	
OGNM	This Study	
EDiN	This Study	
NisGFPDiN	This Study	
NsGiR	This Study	
TNERTFgfr2-/-	This Study	
TNERTFgfr1-/-	This Study	
TNERTFgf4-/-	This Study	
TNERTFgfbp1-/-	This Study	
TNERTNBR1-/-	This Study	
TNERTNBR2-/-	This Study	
TNERTNono-/-	This Study	
E14Tg2aFgf4-/-	This Study	
E14Tg2aFgfr2-/-	This Study	
E14Tg2aFgf4OE	This Study	
Oligonucleotides		
Oligos used for sgRNA cloning, genotyping, qPCR – RTPCR and ChIP-PCR	Supplemental Table 1	
Recombinant DNA		
pU6-(BbsI)-CBh-Cas9-T2A-mCherry	Addgene 64324	(Weber et al., 2015)
Mouse Oct4-GFP GOF18 transgenic reporter	Addgene 60527	(Gafni et al., 2013)
Nanog iRFP670 Fusion Targeting vector	This Study	
Nanog sfGFP Fusion Targeting vector	This Study	
Nanog-2A-mCherry	Addgene 59995	(Faddah et al., 2013)
pEF6V5His-Fgf4	This Study	

PEF6NanogERT2	This Study	
PTripZ-FaNanog	This Study	
pU6-iRFP	This Study	
pU6-iRFP Sg-Fgf4	This Study	
pU6-iRFP Sg-Fgfr1	This Study	
pU6-iRFP Sg-Fgfr2	This Study	
pU6-iRFP Sg-Fgfbp1	This Study	
pU6-iRFP Sg-Zfp281	This Study	
pU6-iRFP Sg-Nono	This Study	
pU6-iRFP Sg-Fgfr2-NBR1	This Study	
pU6-iRFP Sg-Fgfr2-NBR2	This Study	
pU6-iRFP Sg-Nanog-Stop	This Study	
pMKiN	This Study	
Software and Algorithms		
ImageJ	ImageJ	RRID: SCR_003070
FlowJo	BD Bioscience	RRID: SCR_008520
Integrative genomics viewer	Broad Institute	RRID: SCR_011793
SDS	Applied Biosystems	RRID: SCR_015806
Image Lab	Bio-rad	RRID: SCR_014210
GraphPad Prism	GraphPad	RRID: SCR_002798
Other		
Pierce Protein A/G Magnetic beads	Thermo Fisher Scientific	Cat# 88803
BD LSR Fortessa	BD Bioscience	N/A
MoFLo XDP	Beckman Coulter	N/A
Gallios Flowcytometer	Beckman Coulter	N/A
Chemidoc MP imaging system	Bio-rad	
Zeiss Axio Observer	Zeiss	N/A

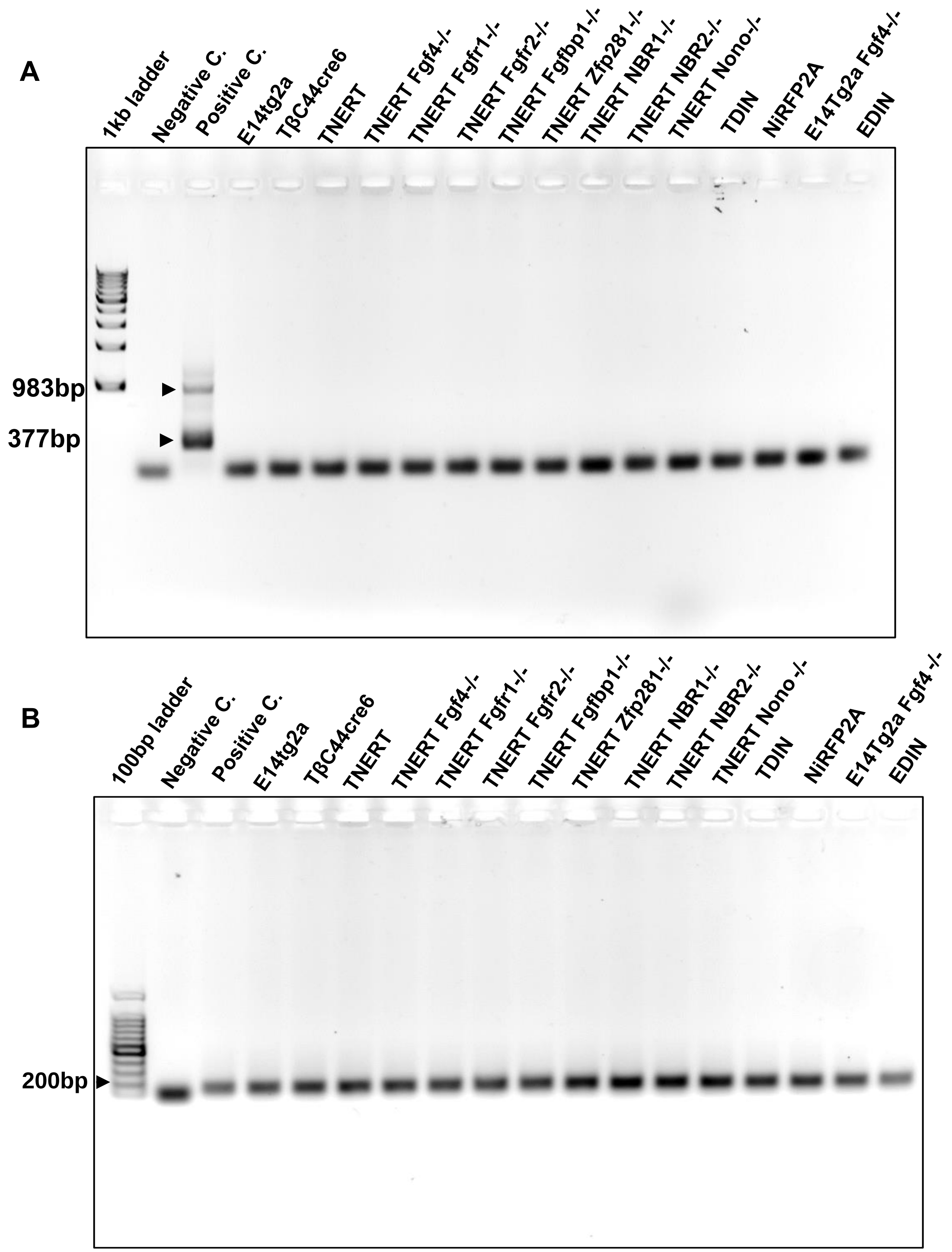

Appendix Figure S3: Mycoplasma contamination analysis of the cell lines using PCR detection.

(A) Agarose gel electrophoresis image of PCR performed to detect the presence of mycoplasma genome in genomic DNA isolated from the mentioned cell lines using lookOut mycoplasma PCR detection kit. Negative C. does not contain any template. Positive C. contains DNA template coding for 16sRNA of mycoplasma. (B) Agarose gel electrophoresis image of PCR performed to detect the presence of mouse genomic DNA and absence of PCR inhibitors in the DNA preparation used for mycoplasma detection using a primer set specific to mouse genomic DNA.

A

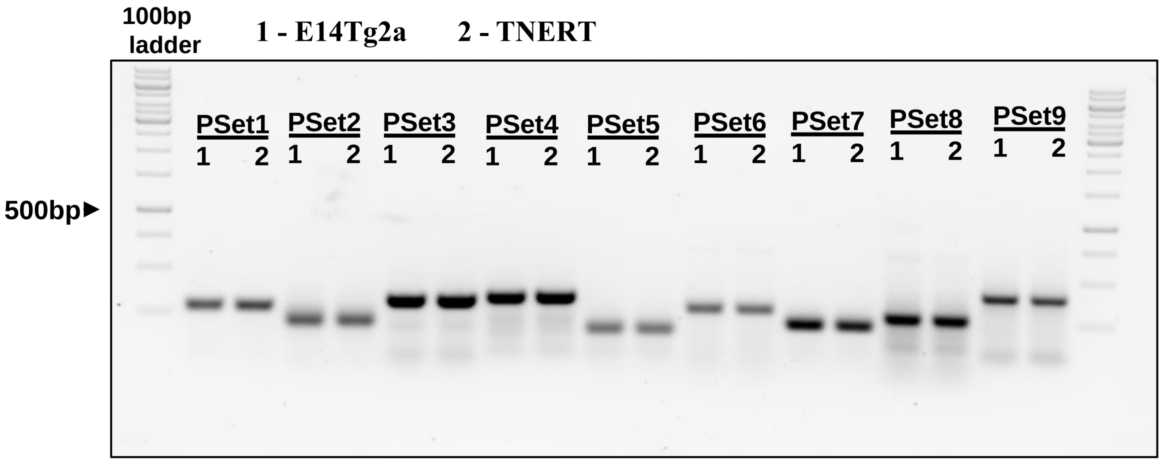

B

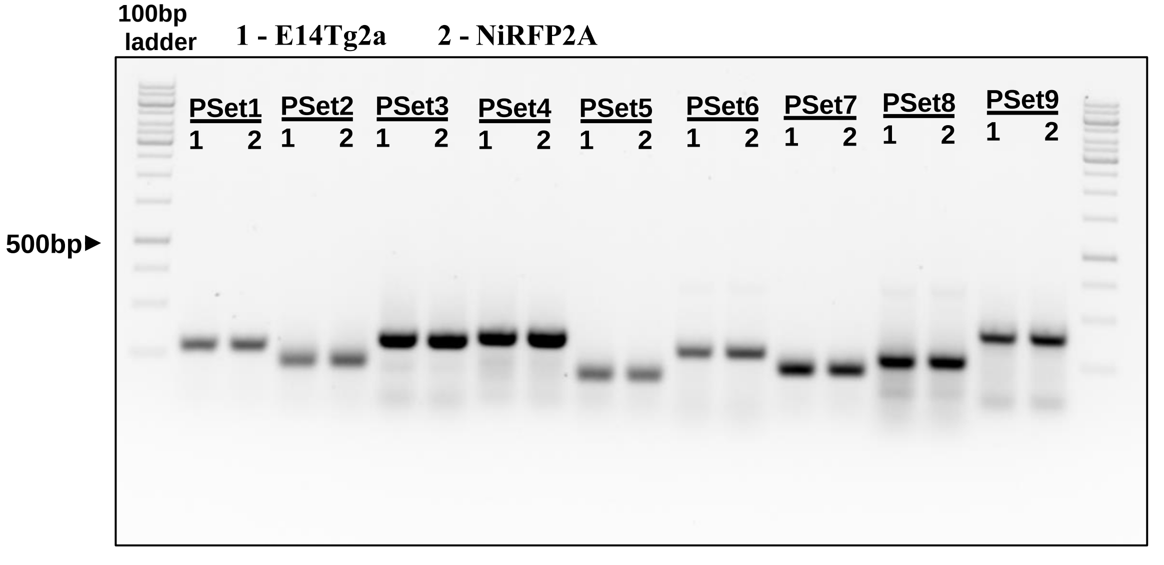

Appendix Figure S4: STR analysis to authenticate the identity of the cell lines

(A) Agarose gel electrophoresis image of PCR performed using primers specific to STR regions of mice. TNERT cell line showed identical STR profile to its parent cell line E14Tg2a. (B) Agarose gel electrophoresis image of PCR performed using primers specific to STR regions of mice. NiRFP2A cell line showed identical STR profile to its parent cell line E14Tg2a. The STR regions and sequence of the primers is given in Appendix table S3.

Appendix table S3: PSet- Primer set used for STR analysis

Primer Set (PSet)	Primer sequence	STR regions	Expected size
PSet 1	TCTTTCTCCTTTTGTGTCATGC	STR -18-3 F	281–313 bp
	TCAAAGTTGGGGTTACAGAATG	STR -18-3 F	
PSet 2	AAGCTTCTCTGGCCATTTGA	4-2 F	217–248bp
	TTCATAAACTTCAAGCAATGACA	4-2 R	
PSet 3	AGTCCACCCAGTGCATTCTC	6-7 F	333–515 bp
	CATGTGGCTGGTATGCTGTT	6-7 R	
PSet 4	GGCTCTCTCACACCTCATCC	9-2 F	318–360 bp
	TCCATGAATCCAGACATTCC	9-2 R	
PSet 5	TCTGGGCGTGTCTGTCATAA	15-3 F	157–222 bp
	TTCTCAGGGAGGAGTGTGCT	15-3 R	
PSet 6	TTTGCAACAGCTCAGTTTCC	6-4 F	276–311 bp
	AATCGCTGGCAGATCTTAGG	6-4 R	
PSet 7	CAAAATTGTCATTGAACACATGTAA	12-1 F	222–259 bp
	CAATGGTCAAGAAATACTGAAGTACAA	12-1 R	
PSet 8	CGTTTTACCTGGCTGACACA	5-5 F	258–298 bp
	GATGCTTGCCTGTTCCTAGC	5-5 R	
PSet 9	GGATGGATGGATGGATGAAA	X-1 F	357–442 bp
	AAGGTATATATCAAGATGGCATTATCA	X-1 R	

Appendix References

1. I. Chambers *et al.*, Nanog safeguards pluripotency and mediates germline development. **450**, 1230-1234 (2007).
2. D. A. Faddah *et al.*, Single-cell analysis reveals that expression of nanog is biallelic and equally variable as that of other pluripotency factors in mouse ESCs. **13**, 23-29 (2013).
3. T. Weber *et al.*, Increasing the efficiency of homology-directed repair for CRISPR-Cas9-induced precise gene editing in mammalian cells. *Nature biotechnology* **33**, 543-548 (2015).
4. O. Gafni *et al.*, Derivation of novel human ground state naive pluripotent stem cells. *Nature* **504**, 282-286 (2013).
5. N. Festuccia *et al.*, Esrrb is a direct Nanog target gene that can substitute for Nanog function in pluripotent cells. **11**, 477-490 (2012).
6. V. Karwacki-Neisius *et al.*, Reduced Oct4 expression directs a robust pluripotent state with distinct signaling activity and increased enhancer occupancy by Oct4 and Nanog. **12**, 531-545 (2013).
7. W.-W. Tee, S. S. Shen, O. Oksuz, V. Narendra, D. J. C. Reinberg, ERK activity promotes chromatin features and RNAPII phosphorylation at developmental promoters in mouse ESCs. **156**, 678-690 (2014).
8. C. Ma *et al.*, Nono, a bivalent domain factor, regulates Erk signaling and mouse embryonic stem cell pluripotency. **17**, 997-1007 (2016).
