# Supplementary material for: A NANOG‐pERK reciprocal regulatory circuit regulates Nanog autoregulation and ERK signaling dynamics
Source: EMBO Rep. 2022 Sep 6;23(11):e54421. doi: 10.15252/embr.202154421 (PMC9638859; doi:10.15252/embr.202154421)
Supplement: Supplementary file 2 — Expanded View Figures PDF [file EMBR-23-e54421-s001.pdf]

Expanded View Figures

Figure EV1. Residual MEK1/2 activity in the ground state prevents complete derepression of *Nanog*.

A (top) Schematic depiction of NiRFP2A with both alleles of *Nanog* fused to iRFP coding sequences in frame with last coding sequence. (Middle) CRISPR mediated knock-in strategy of the iRFP-loxP-IRES-NeoR-loxP cassette into *Nanog* locus. The sgRNA includes the stop codon of the *Nanog* gene. The location of the genotyping primers (FP/RP) for the knock-in is marked by the arrows. (Bottom) Genotyping of the NiRFP2A clones, a 2.9 kb band is amplified only in the knock-in clones as one of the primers is complementary to a sequence outside the left homology arm and the other primer is complementary to the iRFP sequence.

B Relative quantification of OCT4 and SOX2. The expression is normalized relative to HDAC2 levels and expression levels of OCT4 and SOX2 in SL ($n \geq 3$).

C, D Relative pERK expression levels in indicated time points and treatments ($n = 3$).

E Western blot of pERK and ERK in 1 μM PD and increasing concentrations of CHIR in SL media.

F Western blot of pERK and ERK in 3 μM CHIR and increasing concentrations of PD in SL media. G, H Relative pERK expression levels in indicated concentrations of CHIR and PD, respectively ($n \geq 3$).

G, H Relative pERK expression levels in indicated concentrations of CHIR and PD, respectively ($n \geq 3$).

Data information: $n \geq 3$ biological replicates (each dot represents a biological replicate). Data are presented as mean ± SEM in B–D and G–H. *$P < 0.05$, **$P < 0.01$, ***$P < 0.001$, ****$P < 0.0001$ and ns = not significant (paired two-tailed Student's t-test).
Source data are available online for this figure.

© 2022 The Authors

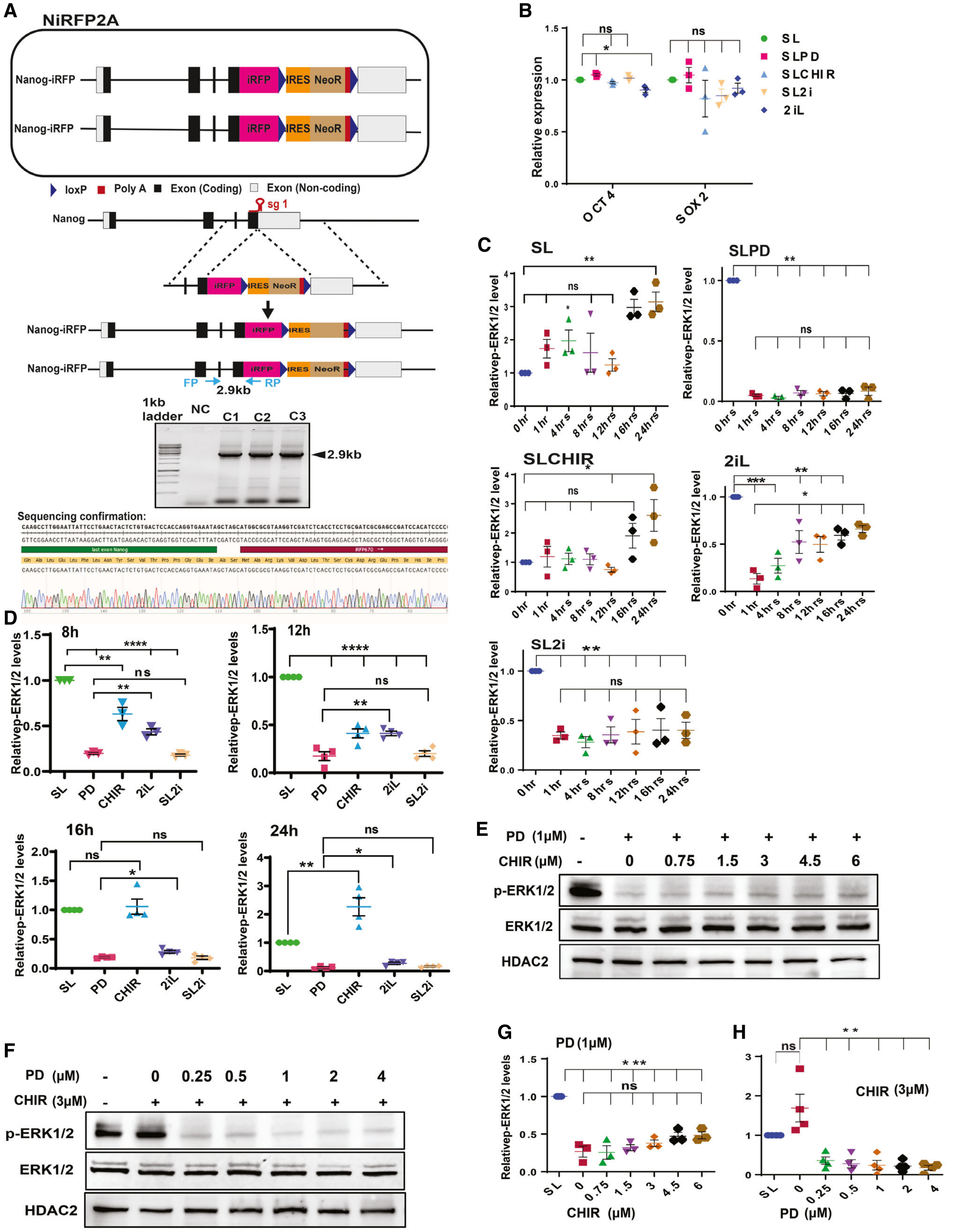
A
NiRFP2A
Nanog-iRFP
Nanog-iRFP
iRFP
IRES NeoR
loxP
Poly A
Exon (Coding)
Exon (Non-coding)
sg 1
Nanog
FP
RP
2.9kb
1kb ladder
NC
C1
C2
C3
Sequencing confirmation:
B
Relative expression
OCT4
SOX2
SL
SLPD
SLCHIR
SL2i
2iL
C
SL
SLPD
SLCHIR
2iL
SL2i
Relativep-ERK1/2 level
D
8h
12h
16h
24h
Relativep-ERK1/2 levels
E
PD (1μM)
CHIR (μM)
p-ERK1/2
ERK1/2
HDAC2
F
PD (μM)
CHIR (3μM)
G
PD (1μM)
CHIR (μM)
H
CHIR (3μM)
PD (μM)

Figure EV1.

Figure EV2. FGF autocrine signaling pathway components are essential for *Nanog* autoregulation.

A Immunofluorescence of NANOG in TNERT cells after 30 min treatment with or no OHT. Scale bars 5 μM.

B (left) Schematic of Doxycycline inducible TDiN cell line, generated by the introduction of a Tetracycline inducible Flag-Avi-NANOG transgene in Tβc44Cre6 cell line. (Right) Western blot of NANOG in TDiN cells after 48 h treatment with or no Doxycycline.

C FACS profiles of TNERT treatment with or no OHT in SLCHIR and SL2i. The dotted line represents the FACS profile of unstained E14Tg2a cells used as negative control (−ve c).

D FACS profiles of TDiN cell line in SL, SLCHIR, 2iL, SL2i, and SLPD. The dotted line represents the FACS profile of unstained E14Tg2a cells used as negative control (−ve c). (Bottom right) *Nanog*:GFP population median of TDiN in indicated treatments ($n = 3$).

E *Nanog*:GFP population median of TNERT, and TDiN treated with SU5402, with OHT/Doxycycline or no OHT/Doxycycline.

F (top) CRISPR-based knock-out strategy using paired sgRNA to knock-out *Fgf4* in TNERT. The sgRNAs are positioned at the beginning and the near end of exon II. The deletion results in the loss of the start codon and a part of the coding region in exon II. The dotted line represents the deleted region of the gene. FP and RP represent the relative positions of the genotyping primers. (Middle left) genotyping PCR of TNERTFgf4$^{-/-}$ clones. The WT allele gives an amplicon of 332 bp and the knock-out allele has a smaller amplicon by 32 bps or more. (Middle right) The relative abundance of FGF4 in media of TNERT and TNERTFgf4$^{-/-}$ clones 48 h after OHT treatment. (Bottom) Chromatogram of the TNERTFgf4$^{-/-}$ clones showing the sequences at the junction of the deletion.

G (top) Schematic of the gene structure of *Fgfbp1* and the relative positions of the two sgRNAs used for paired sgRNA knock-out strategy. One sgRNA is complimentary to 5′UTR and the other to the 3′ end of the coding region of the only exon. (Middle left) Genotyping PCR showing a WT amplicon of 1,220 bps and an amplicon around 400 bps in case of deletion. (Middle right) The relative abundance of FGFBP1 in media of TNERT and TNERTFgfbp1$^{-/-}$ clones 48 h after OHT treatment. (Bottom) Chromatogram of the TNERTFgfbp1$^{-/-}$ clone showing the sequences at the junction of the deletion.

H Strategy for knock-out of *Fgfr1* in TNERT cells. The schematic depicts the gene structure of *Fgfr1* with the relative positions of the two sgRNAs. One sgRNA targets the 3′end of Intron 8 and the other exon10. (Middle left) Genotyping PCR shows a WT allele amplicon at 494 bp and a knock-out allele with smaller amplicons around 150 bp. (Middle right) Western blot analysis of FGFR1 in TNERT and TNERTFgfr1$^{-/-}$ clones. (Bottom) chromatogram showing the sequence of the deleted region.

I A paired sgRNA strategy to knock-out *Fgfr2* in TNERT. (Top) The schematic represents the *Fgfr2* gene structure, with relative positions of the sgRNAs. One sgRNA target exon2 and the other sgRNA targets the coding region of the last exon approximately 100 kb apart. The dotted line represents the region of deletion in the *Fgfr2* gene. (Middle left) PCR genotyping shows a 665 bp amplicon when at least one allele of *Fgfr2* is deleted. This genotyping strategy cannot distinguish between +/− and −/− genotypes. (Middle right) Western blot analysis of FGFR2 protein in the *Fgfr2* targeted clones distinguishing the +/− and −/− clones. (Bottom) chromatogram represents the sequence of the genotyping amplicon indicating the exact sites of deletion.

Data information: $n \geq 3$ biological replicates (each dot represents a biological replicate). Data are presented as mean ± SEM in D and E. $^{**}P < 0.01$, $^{***}P < 0.001$, $^{****}P < 0.0001$ and ns = not significant (paired two-tailed Student's *t*-test).
Source data are available online for this figure.

 © 2022 The Authors

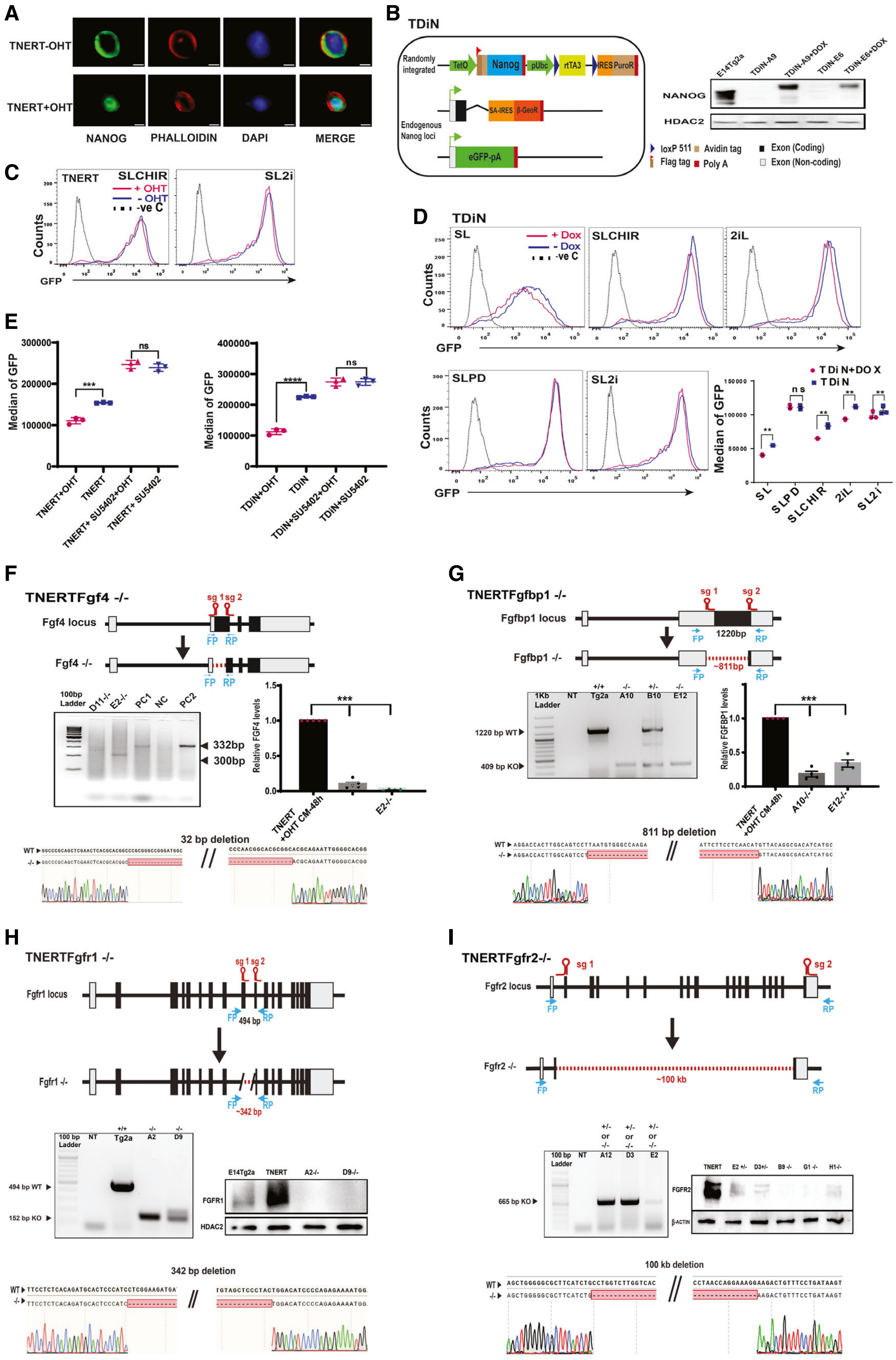
A
TNERT-OHT
TNERT+OHT
NANOG PHALLOIDIN DAPI MERGE
B TDiN
Randomly integrated
TetO Nanog pUbc rtTA3 IRES PuroR
Endogenous Nanog loci
SA-IRES β-GeoR
eGFP-pA
E14Tg2a TDiN-A9 TDiN-A9+DOX TDiN-E6 TDiN-E6+DOX
NANOG
HDAC2
loxP 511 Avidin tag Exon (Coding)
Flag tag Poly A Exon (Non-coding)
C
TNERT SLCHIR SL2i
+ OHT
- OHT
-ve C
Counts
GFP
D TDiN
SL SLCHIR 2iL SLPD SL2i
+ Dox
- Dox
-ve C
Counts
GFP
TDiN+DOX
TDiN
Median of GFP
E
Median of GFP
TNERT+OHT TNERT TNERT+ SU5402+OHT TNERT+ SU5402
TDiN+OHT TDiN TDiN+SU5402+OHT TDiN+SU5402
F
TNERTFgf4 -/-
Fgf4 locus
Fgf4 -/-
sg 1 sg 2
FP RP
100bp Ladder D11-/- E2-/- PC1 NC PC2
332bp
300bp
Relative FGF4 levels
TNERT +OHT CM-48h E2-/-
32 bp deletion
G
TNERTFgfbp1 -/-
Fgfbp1 locus
Fgfbp1 -/-
1220bp
~811bp
1Kb Ladder NT +/+ Tg2a -/- A10 +/- B10 -/- E12
1220 bp WT
409 bp KO
Relative FGFBP1 levels
TNERT +OHT CM-48h A10-/- E12-/-
811 bp deletion
H
TNERTFgfr1 -/-
Fgfr1 locus
Fgfr1 -/-
494 bp
~342 bp
100 bp Ladder NT +/+ Tg2a -/- A2 -/- D9
494 bp WT
152 bp KO
E14Tg2a TNERT A2-/- D9-/-
FGFR1
HDAC2
342 bp deletion
I
TNERTFgfr2-/-
Fgfr2 locus
Fgfr2 -/-
~100 kb
100 bp Ladder NT A12 D3 E2
665 bp KO
TNERT E2 +/- D3+/- B9 -/- G1 -/- H1-/-
FGFR2
β-ACTIN
100 kb deletion

Figure EV2.

Figure EV3. *Nanog* enhances the expression of FGF autocrine signaling pathway components.

A Browser tracks of NANOG enrichment in Fragment Per Kilobase of transcripts per Million (FPKM) in Oct4$^{+/+}$ cells (normal NANOG levels) and Oct4$^{+/-}$ cells (higher NANOG levels at *Fgf4*, *Fgfbp1*, *Fgfr1*, and *Fgfr2* loci) (Data ref: Karwacki-Neisius *et al*, 2013b).

B Histogram of FGFR1 expression on the cell surface analyzed by immunostaining and FACS of fixed but unpermeabilized TNERT cells treated with (red) or no OHT (blue). The dotted line represents the FACS profile of E14Tg2a cells stained with control IgG and secondary antibody used as negative control (−ve c).

C Relative expression levels of pERK, FGFR2, and FGFR1 at indicated time points after OHT treatment in TNERT cells ($n > 3$).

D ELISA-based relative quantification of FGF4 and FGFBP1in media from EDiN cells cultured with or no Doxycycline ($n = 3$). EDiN cell was generated by introducing a Doxycycline inducible Flag-Avi-NANOG transgene in E14Tg2a cells.

E *Nanog*:GFP population median of Tβc44Cre6 treated with OHT-induced conditioned media collected after different time points ($n = 3$).

F (top) Schematic of TNERTZfp281$^{-/-}$ cells, (upper-middle) CRISPR-based paired guide knock-out strategy indicating the relative position of the sgRNAs, FP and RP indicate the genotyping primers. (Lower middle) Genotyping PCR indicating +/− and −/− clones. (Bottom) The sequencing chromatogram of the deleted region confirms the exact site of deletion, followed by RT-qPCR analysis of the *Zfp281* transcripts.

G *Nanog*:GFP population median of Tβc44Cre6 treated with conditioned media from TNERT + OHT 0 h, TNERTFGF4$^{-/-}$ + OHT 48 h, Tβc44Cre6 48 h, E14Tg2a-FGF4-OE (overexpression) 48 h, TNERT + OHT 48 h and 50 ng/ml FGF4 ($n = 3$).

H, I ELISA-based relative quantities of FGF4 and FGFBP1 in media from TNERT after 18, 24, and 48 h of OHT treatment ($n = 3$).

J ELISA-based relative quantities of FGF4 in conditioned media from cell lines—TNERT + OHT 0 h, TNERTFGF4$^{-/-}$ + OHT 48 h, E14Tg2a-FGF4-OE 48 h (overexpression), TNERT−/+OHT 48 h, and 50 ng/ml FGF4 ($n = 3$).

K ELISA-based relative quantities of FGFBP1 in conditioned media from various cell lines—TNERT + OHT 0 h, TNERT-Fgfbp1$^{-/-}$ 48 h + OHT, TNERT 48 h −/+OHT, and 50 ng/ml FGBP1 ($n = 3$).

Data information: $n \geq 3$ biological replicates (each dot represents a biological replicate). Data are presented as mean ± SEM in C and D–K. *$P < 0.05$, **$P < 0.01$, ***$P < 0.001$, ****$P < 0.0001$ and ns = not significant (paired two-tailed Student's *t*-test).

 © 2022 The Authors

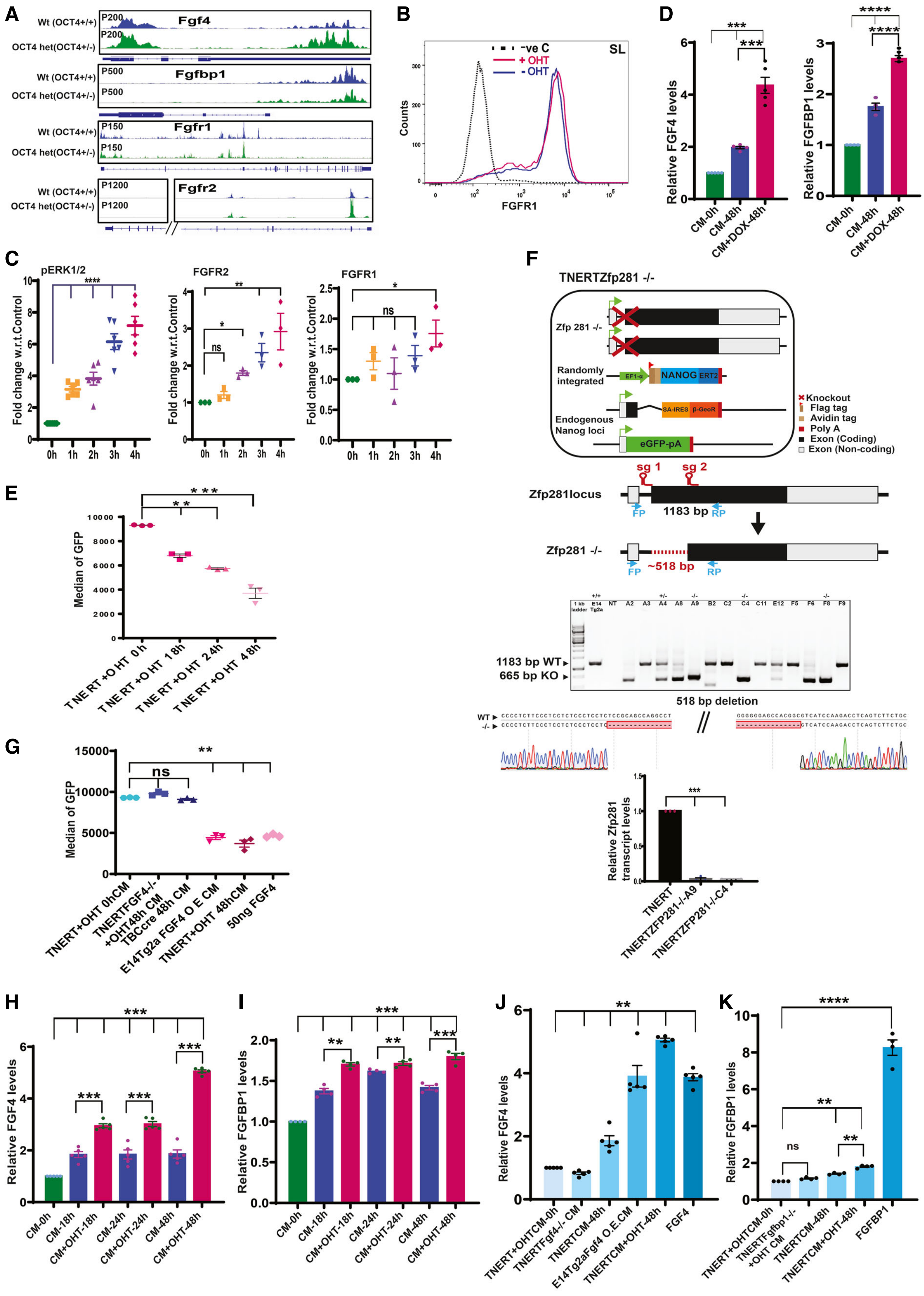
A
Fgf4
Fgfbp1
Fgfr1
Fgfr2
Wt (OCT4+/+)
OCT4 het(OCT4+/-)
B
-ve C
+ OHT
- OHT
SL
Counts
FGFR1
D
Relative FGF4 levels
Relative FGFBP1 levels
CM-0h
CM-48h
CM+DOX-48h
C
pERK1/2
FGFR2
FGFR1
Fold change w.r.t.Control
0h 1h 2h 3h 4h
E
Median of GFP
TNERT +OHT 0h
TNERT +OHT 18h
TNERT +OHT 24h
TNERT +OHT 48h
F
TNERTZfp281 -/-
Zfp 281 -/-
Randomly integrated
NANOG ERT2
Endogenous Nanog loci
eGFP-pA
Knockout
Flag tag
Avidin tag
Poly A
Exon (Coding)
Exon (Non-coding)
Zfp281locus
1183 bp
Zfp281 -/-
~518 bp
1183 bp WT
665 bp KO
518 bp deletion
Relative Zfp281 transcript levels
TNERT
TNERTZFP281-/-A9
TNERTZFP281-/-C4
G
Median of GFP
TNERT+OHT 0hCM
TNERTFGF4-/- +OHT48h CM
TBCcre 48h CM
E14Tg2a FGF4 O E CM
TNERT+OHT 48hCM
50ng FGF4
H
Relative FGF4 levels
I
Relative FGFBP1 levels
CM-0h
CM-18h
CM+OHT-18h
CM-24h
CM+OHT-24h
CM-48h
CM+OHT-48h
J
Relative FGF4 levels
TNERT+OHTCM-0h
TNERTFgf4-/- CM
TNERTCM-48h
E14Tg2aFgf4 O.E.CM
TNERTCM+OHT-48h
FGF4
K
Relative FGFBP1 levels
TNERT+OHTCM-0h
TNERTFgfbp1-/- +OHT CM
TNERTCM-48h
TNERTCM+OHT-48h
FGFBP1

Figure EV3.

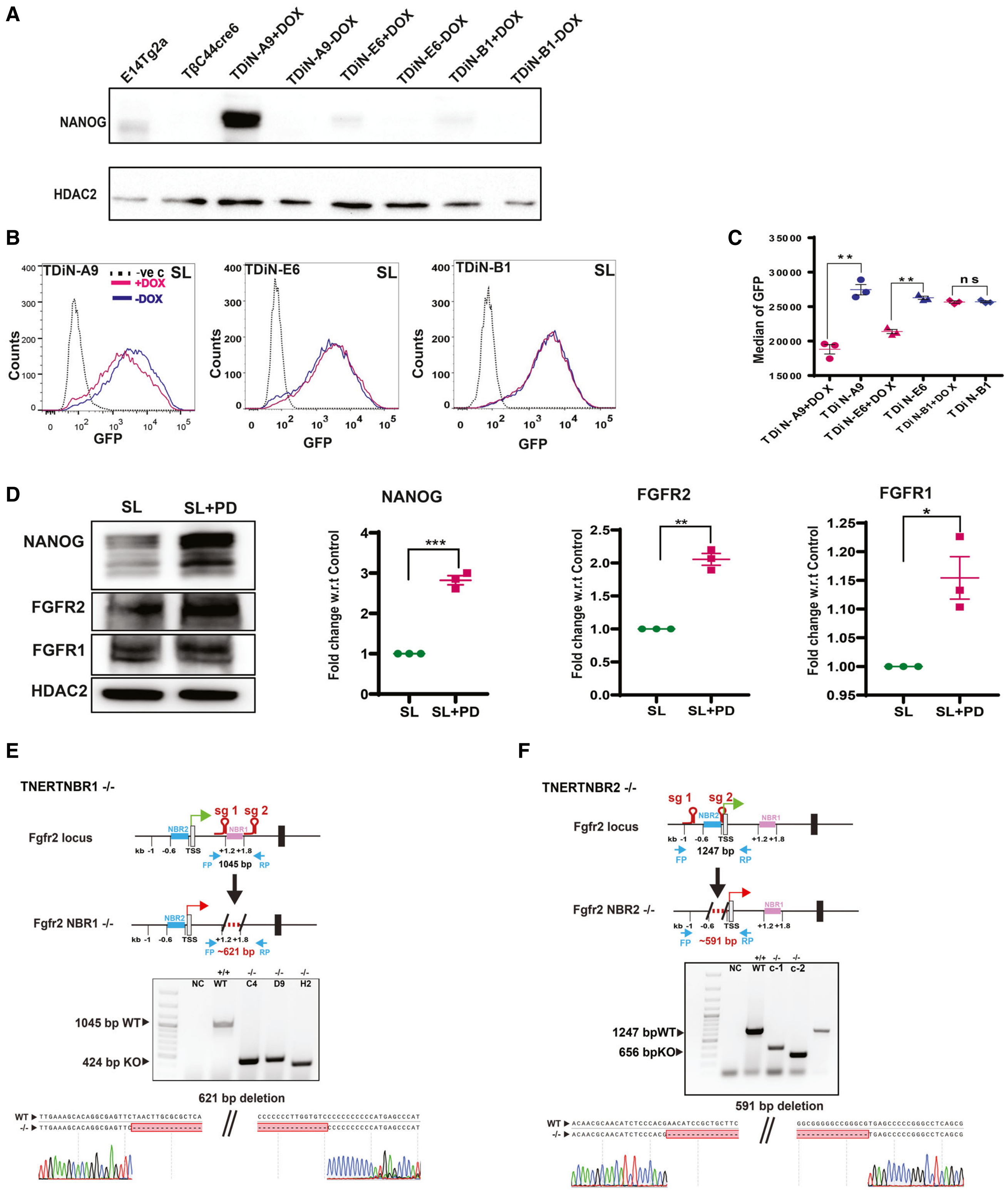

Figure EV4.

 © 2022 The Authors

Figure EV4. NANOG-induced FGFR2 triggers autoregulation predominately in the ES cell population with higher *Nanog* expression.

A Western blot analysis of Flag-Avi-NANOG in different clones of TDiN treated with or no Doxycycline showing different levels of NANOG expression relative to E14Tg2a. The clones show different levels of expression Flag- Avi-NANOG upon Doxycycline treatment.
B FACS profiles of *Nanog*:GFP in TDiN clones treated with or no Doxycycline. The dotted line represents the FACS profile of unstained E14Tg2a cells used as negative control (−ve c).
C *Nanog*:GFP population median of TDiN clones (n = 3).
D (left) Western blot of NANOG, FGFR2, and FGFR1 in E14Tg2a cultured in SLPD for 48 h. (Right) Fold change in expression of NANOG, FGFR2, and FGFR1 in E14Tg2a cultured in SLPD relative to SL (n = 3).
E (top) Schematic of strategy for deletion of NANOG Binding Region 1 (NBR1) in TNERT indicating the position of the NBR1 and the relative position of the sgRNA pair. The sgRNAs are complementary to sequences around 1.2 kb and 1.8 kb downstream of TSS. FP and RP indicate the relative position of the primers for genotyping. (Middle) Genotyping of TNERT NBR1 knock-out clones. The WT shows an amplicon of 1,045 bp, upon deletion around 600 bp sequence comprising multiple NANOG binding sites is deleted. (Bottom) Sequence and chromatogram of the genotype PCR amplicon indicating the exact sequence of the junction of deletion in TNERTNBR1$^{-/-}$ clone.
F Schematic of strategy for deletion of NANOG Binding Region 2 (NBR2) in TNERT indicating the position of NBR2 and relative position of the sgRNA pair. The sgRNAs are complementary to sequences around TSS and 0.6 kb upstream of TSS of *Fgfr2*. (Middle) Genotyping of the TNERTNBR2 knock-out clones. The WT shows an amplicon of 1,247 bp. The knock-out would lead to deletion of around 690 bps and a smaller amplicon of around 650 bps. (Bottom) Sequence and chromatogram of the PCR amplicon from TNERT knock-out clones showing the exact site of deletion in TNERT NBR2$^{-/-}$ clone.

Data information: $n \geq 3$ biological replicates (each dot represents a biological replicate). Data are presented as mean ± SEM in C and D. *$P < 0.05$, **$P < 0.01$, ***$P < 0.001$ and ns = not significant (paired two-tailed Student's t-test).
Source data are available online for this figure.

Figure EV5. ERK interacts and recruits NONO to repress *Nanog* transcription.

A (left) FACS profile of TNERTTcf15$^{-/-}$ treated with or no OHT (n = 3). The dotted line represents the FACS profile of unstained E14Tg2a cells used as negative control (−ve c). (Right) *Nanog*:GFP population median of TNERT and TNERTTcf15$^{-/-}$ treated with or no OHT (n = 3).
B A CRISPR-based knock-out strategy using paired sgRNA, to knock-out of *Nono* in TNERT cells. (Top) The schematic represents the mouse *Nono* gene structure with relative positions of the two sgRNAs flanking the second coding exon of *Nono*. FP and RP indicate the relative position of genotyping primers. The dotted line indicates the region of deletion in the *Nono* gene. (Middle) Genotyping PCR of the *Nono*$^{-/-}$ deletion in TNERT. The WT allele gave an amplicon of 711 bp and the deleted allele shows a smaller amplicon of 330 bp; followed by sequence and chromatogram indicating the deletion site (bottom) Western blot analysis of NONO protein in TNERT and TNERTNono$^{-/-}$ clones.
C (C)*Nanog*:GFP population median of TNERT and TNERTNono$^{-/-}$ treated with or no OHT (n = 3).
D The relative abundance of pERK in TNERT treated with or no OHT and TNERTNono$^{-/-}$ with OHT (n = 4).
E Browser tracks of pERK, NONO, POL2, H3K4me3, H3K27me3 enrichment in Fragment Per Kilobase of transcripts per Million (FPKM) on *Nanog* gene (Data ref: Ma *et al*, 2016b; Data ref: Tee *et al*, 2014b).
F ChIP-qPCR analysis of POL2 enrichment on Nanog locus in E14Tg2a (WT) and Nono$^{-/-}$ cell lines. Refer to the schematic in Fig 5F for the coordinates of S1–S9.
G ChIP-qPCR analysis of pERK, NONO, H3K27, H3K4, and POL2 enrichment on a non-genic region and a known validated target locus. The non-genic region named ORF Free Region (OFR) corresponds to Chr6:43023477 + 43023592 (mm10). *HoxC11* and *Oct4* were used as a known validated target genes for ChIP-qPCR.
H The relative abundance of NANOG after 0, 2, 4, 6, and 8 h of Cycloheximide (CHX) chase cultured in SL, PD, and FGF4 (n = 3).

Data information: $n \geq 3$ biological replicates (each dot represents a biological replicate). Data are presented as mean ± SEM in A, C, D, and F–H. *$P < 0.05$, **$P < 0.01$, ***$P < 0.001$, ****$P < 0.0001$ and ns = not significant (paired two-tailed Student's t-test).
Source data are available online for this figure.

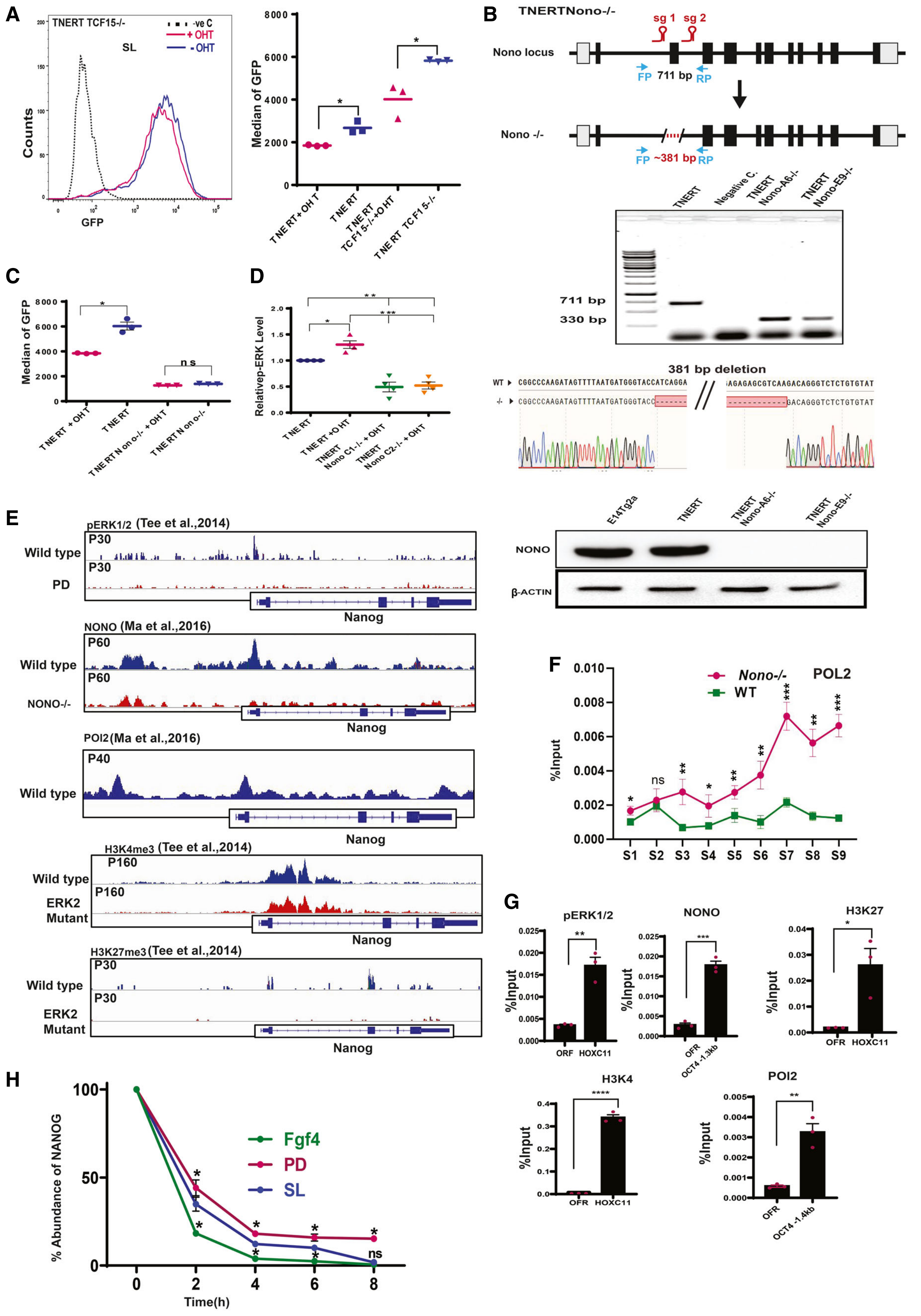

Figure EV5.

 © 2022 The Authors
