# Supplementary figures and images for: A NANOG‐pERK reciprocal regulatory circuit regulates Nanog autoregulation and ERK signaling dynamics
Source: EMBO Rep. 2022 Sep 6;23(11):e54421. doi: 10.15252/embr.202154421 (PMC9638859; doi:10.15252/embr.202154421)

# EV1E

|           |   |   |      |     |   |     |   |
|-----------|---|---|------|-----|---|-----|---|
| PD(1μM)   | - | + | +    | +   | + | +   | + |
| CHIR(1μM) | - | 0 | 0.75 | 1.5 | 3 | 4.5 | 6 |

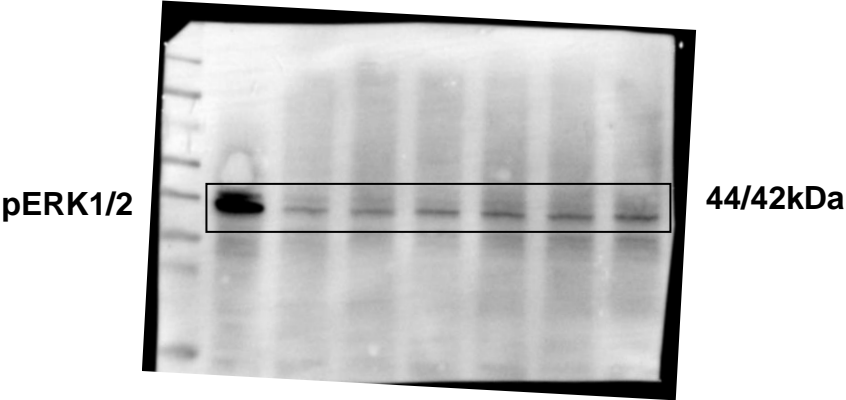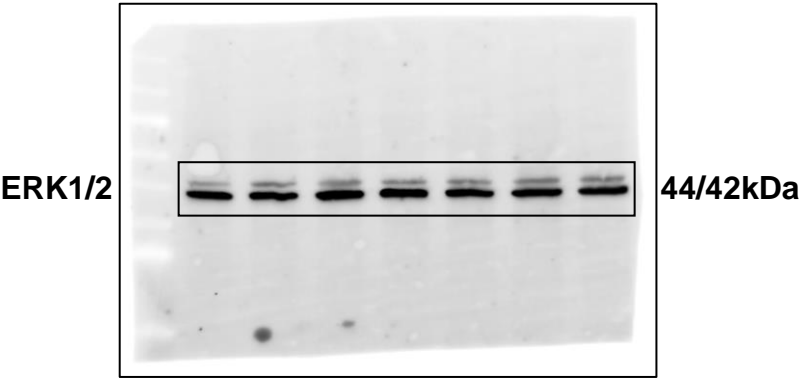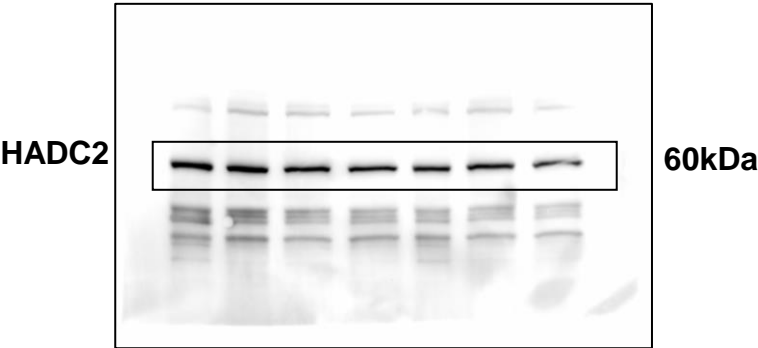

# EV1F

|           |   |   |      |     |   |   |   |
|-----------|---|---|------|-----|---|---|---|
| PD(1μM)   | - | 0 | 0.25 | 0.5 | 1 | 2 | 4 |
| CHIR(1μM) | - | + | +    | +   | + | + | + |

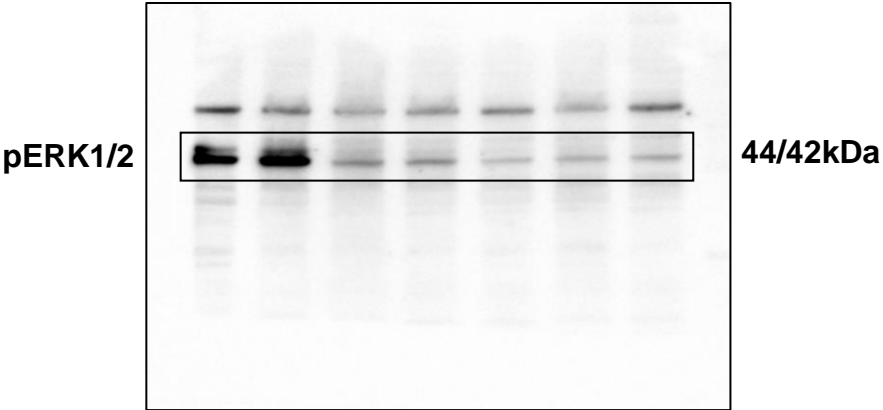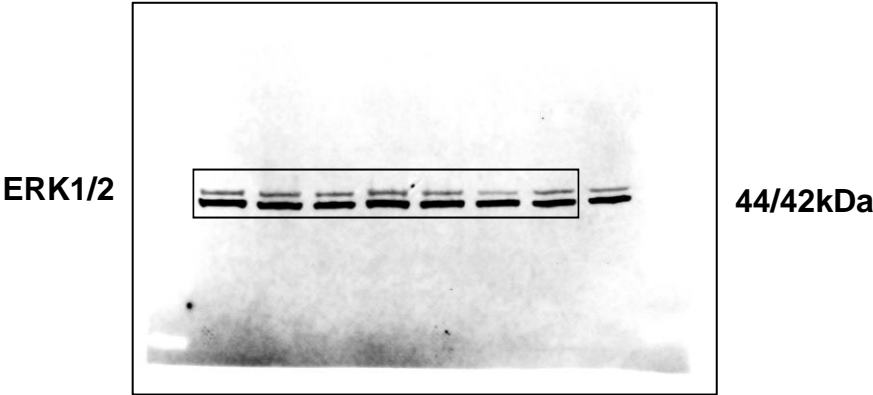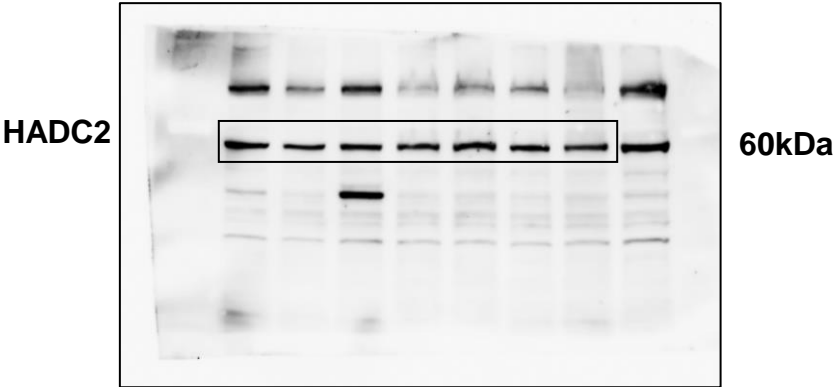

Supplement: Supplementary file 3 — Source Data for Expanded View [file EMBR-23-e54421-s008.zip › EV_Figure_Source_Data/EMBOR-2021-54421V4-Figure_EV1_Source_Data-sd.pdf]

EV2 B

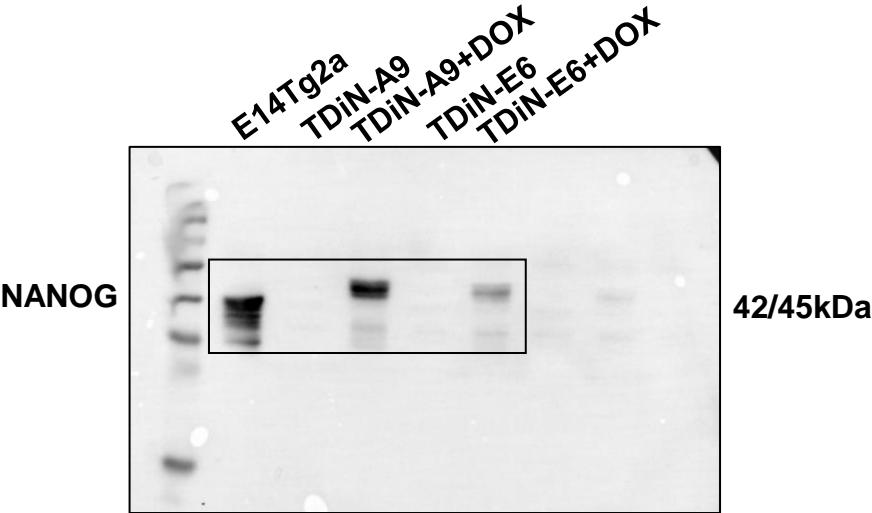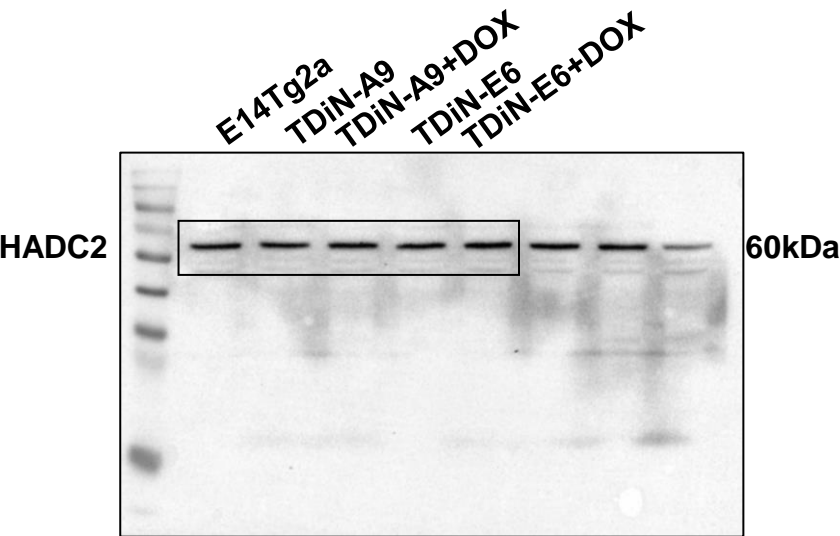

EV2H

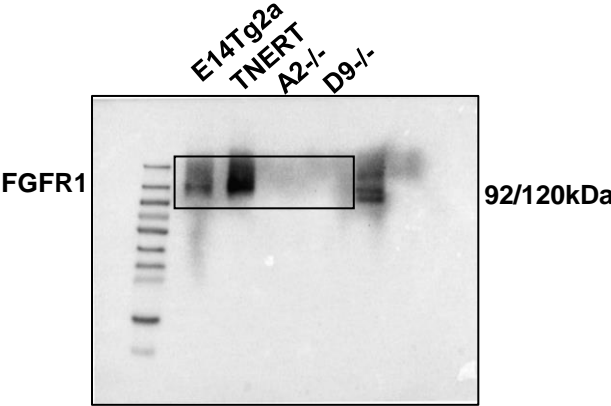

EV2I

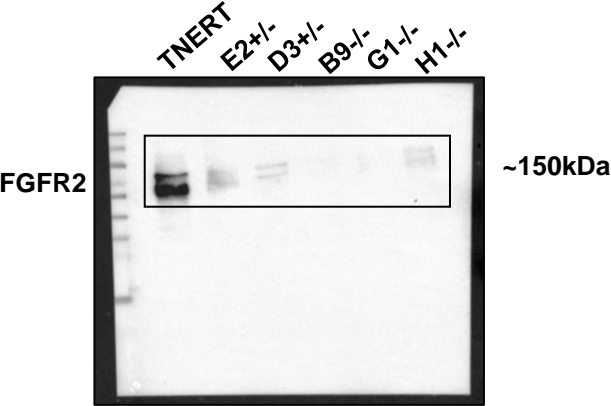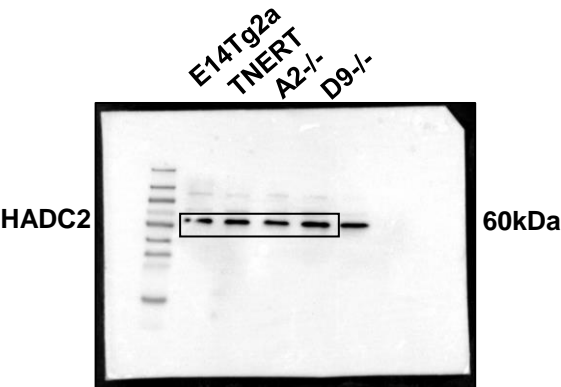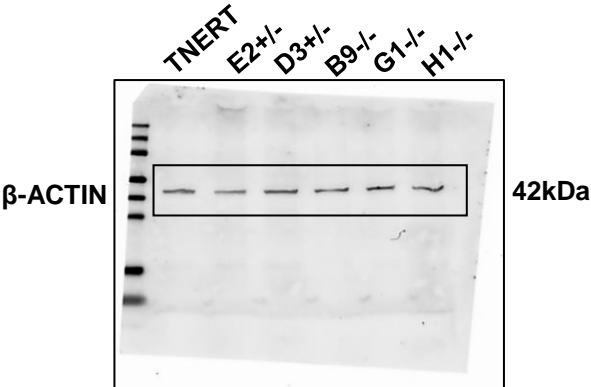

Supplement: Supplementary file 3 — Source Data for Expanded View [file EMBR-23-e54421-s008.zip › EV_Figure_Source_Data/EMBOR-2021-54421V4-Figure_EV2_Source_Data-sd.pdf]

EV4 A

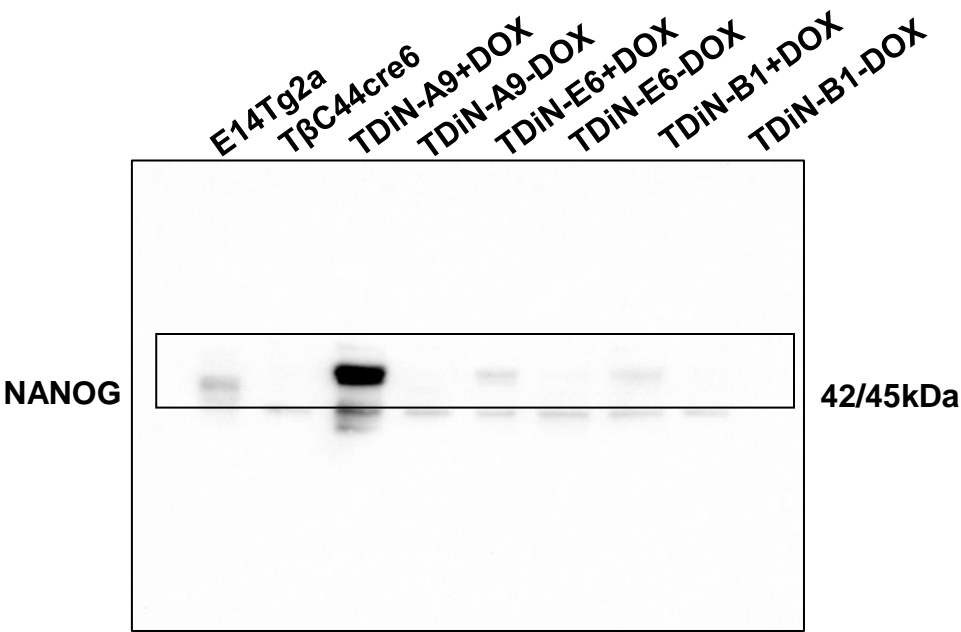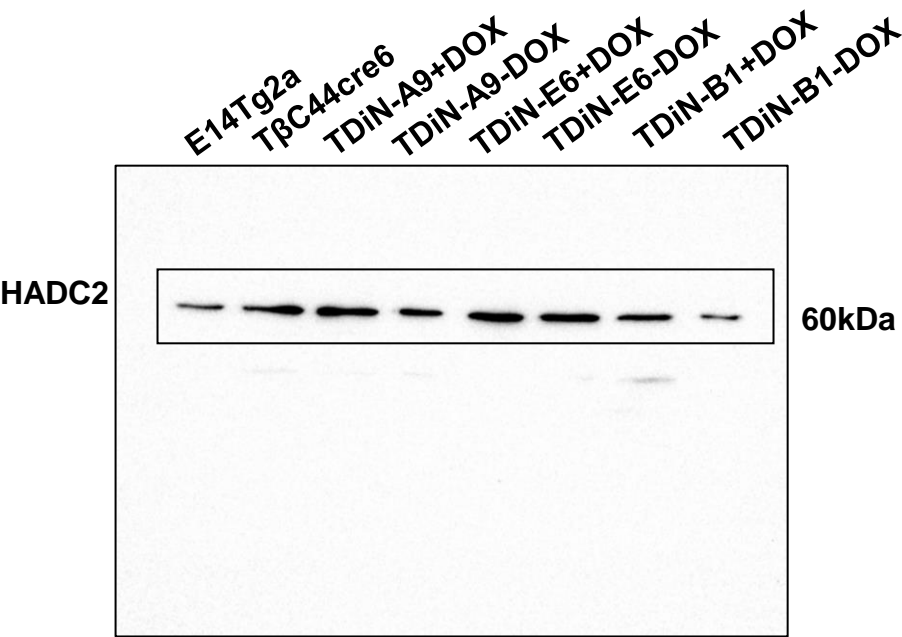

# EV4 D

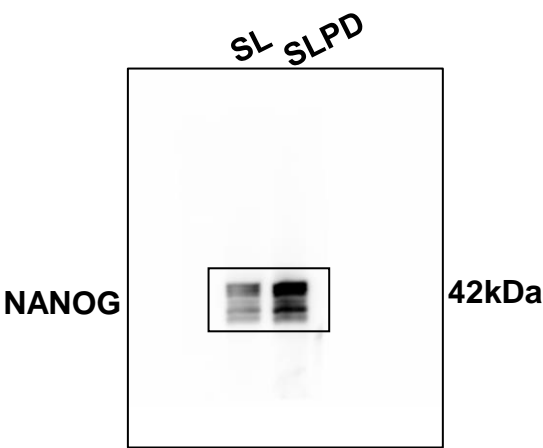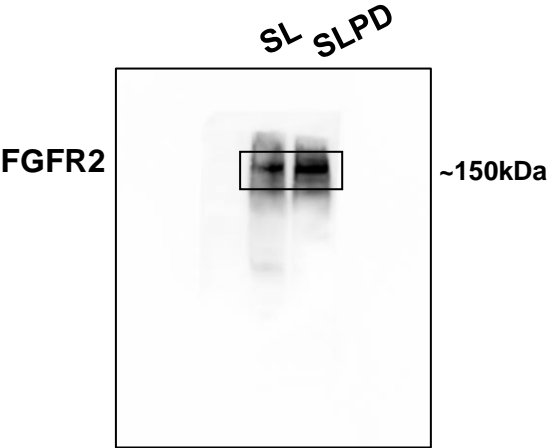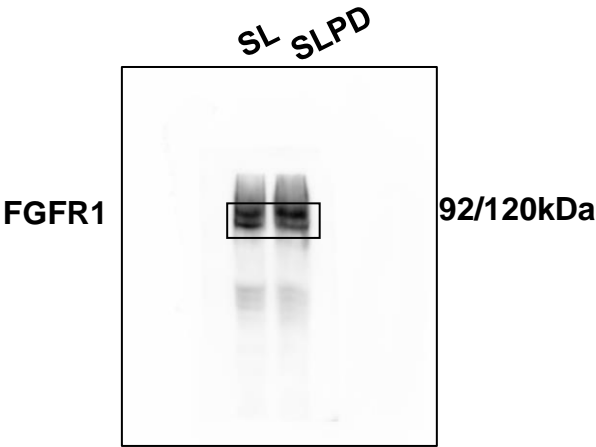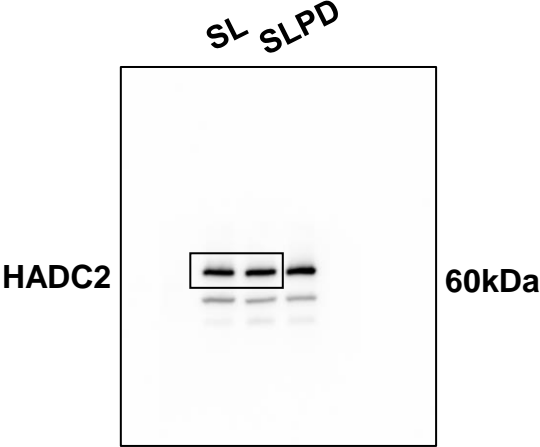

Supplement: Supplementary file 3 — Source Data for Expanded View [file EMBR-23-e54421-s008.zip › EV_Figure_Source_Data/EMBOR-2021-54421V4-Figure_EV4_Source_Data-sd.pdf]

# EV5 B

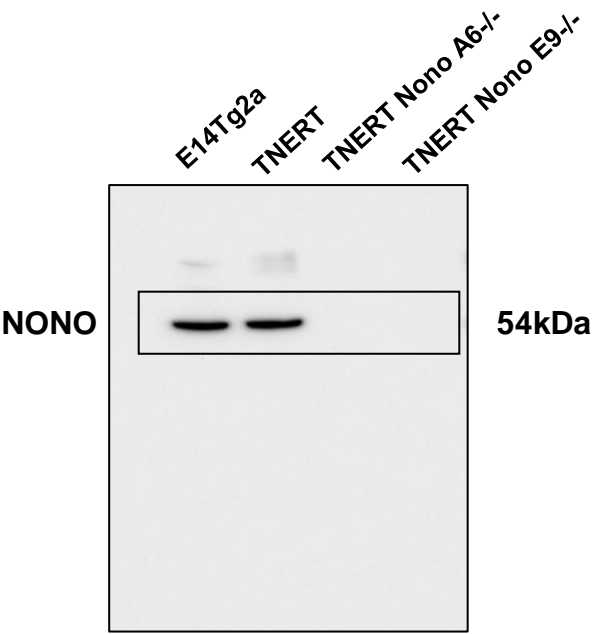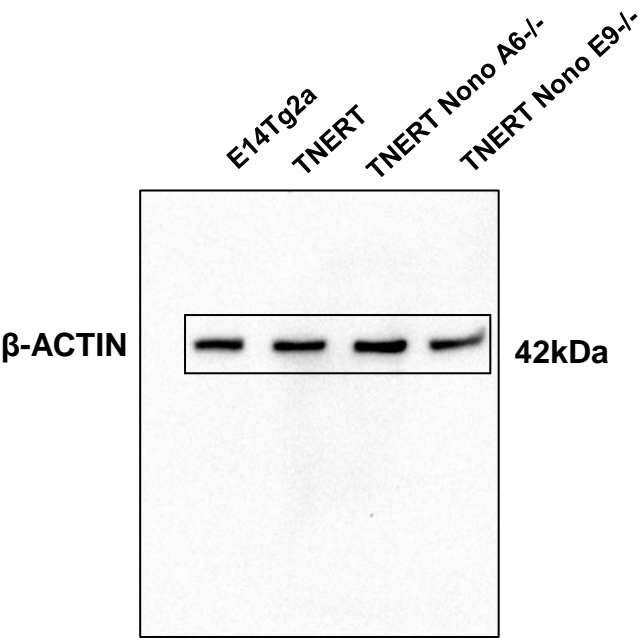

Supplement: Supplementary file 3 — Source Data for Expanded View [file EMBR-23-e54421-s008.zip › EV_Figure_Source_Data/EMBOR-2021-54421V4-Figure_EV5_Source_Data-sd.pdf]

# Figure1E

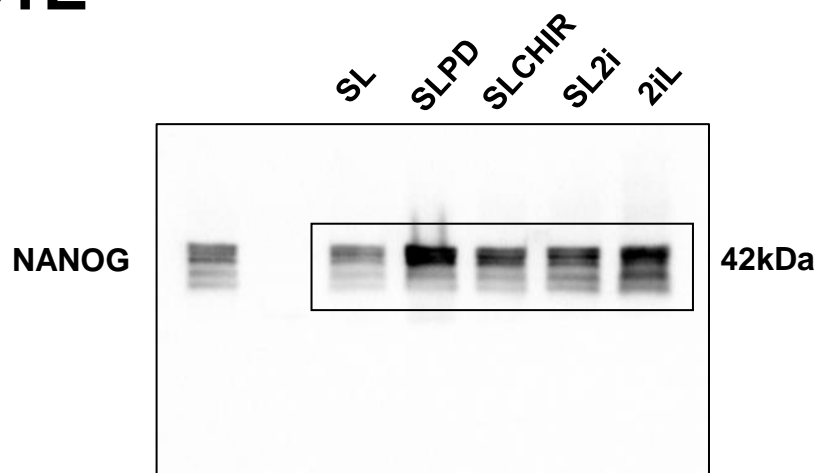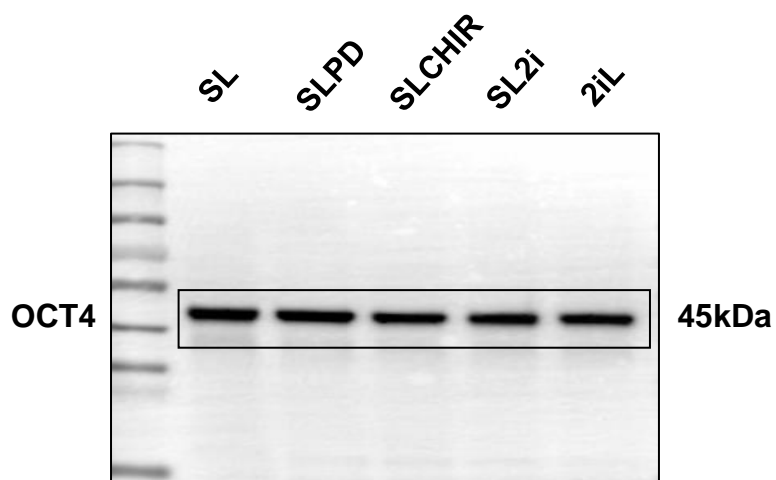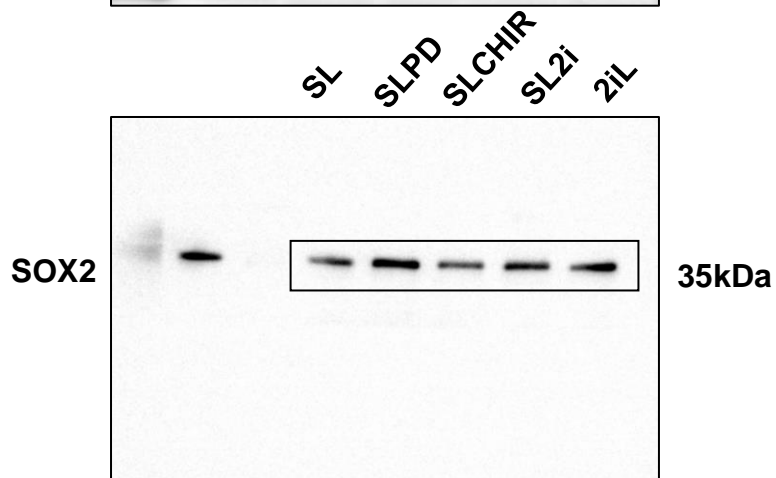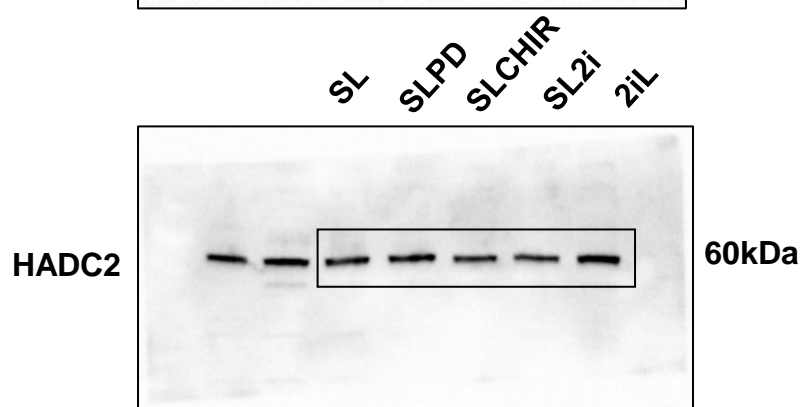

# Figure1F

SL

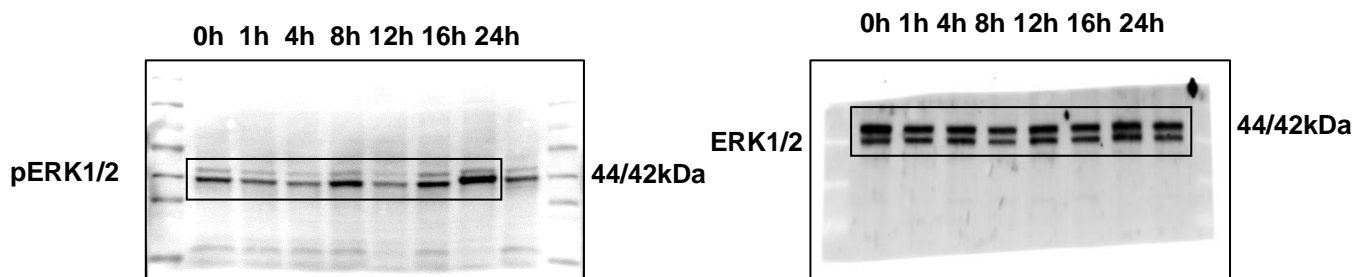

SLPD

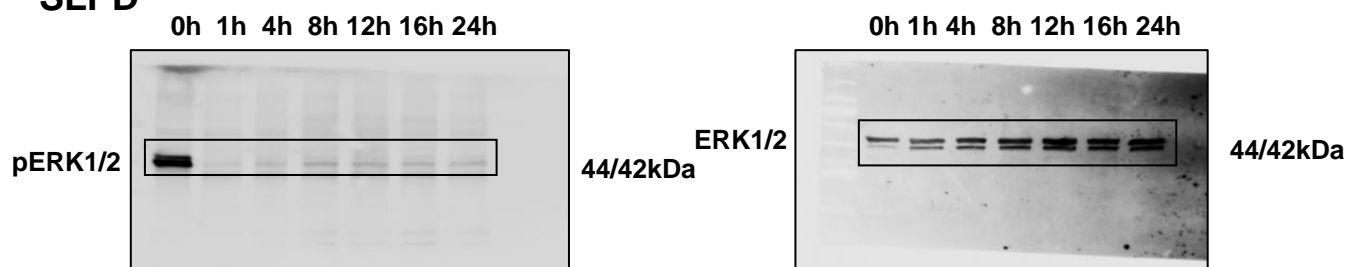

SLCHIR

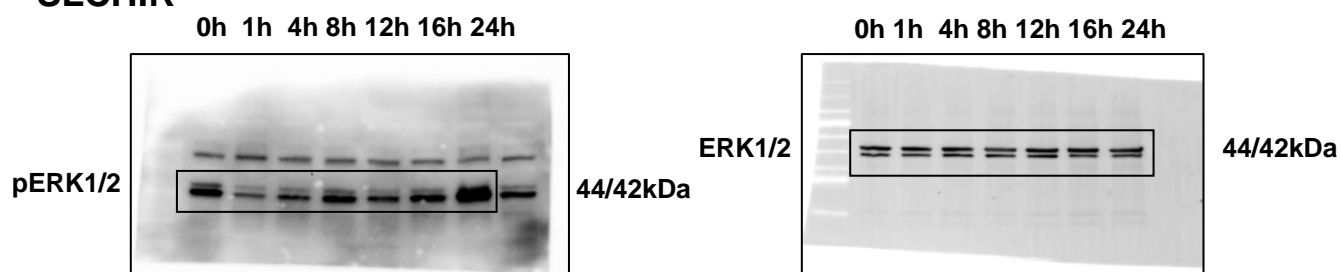

2iL

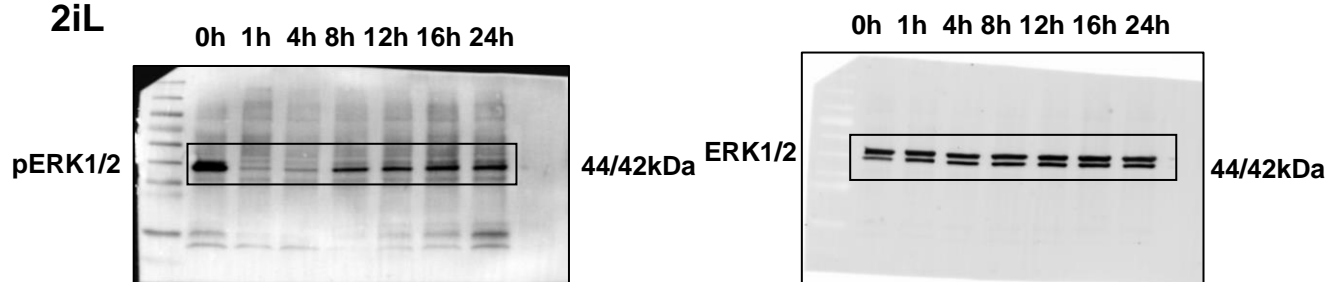

SL2i

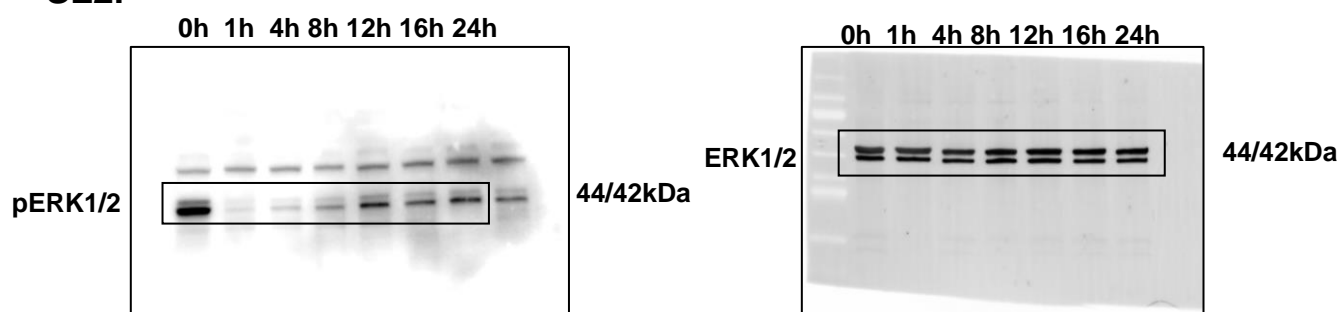

# Figure1G

8h

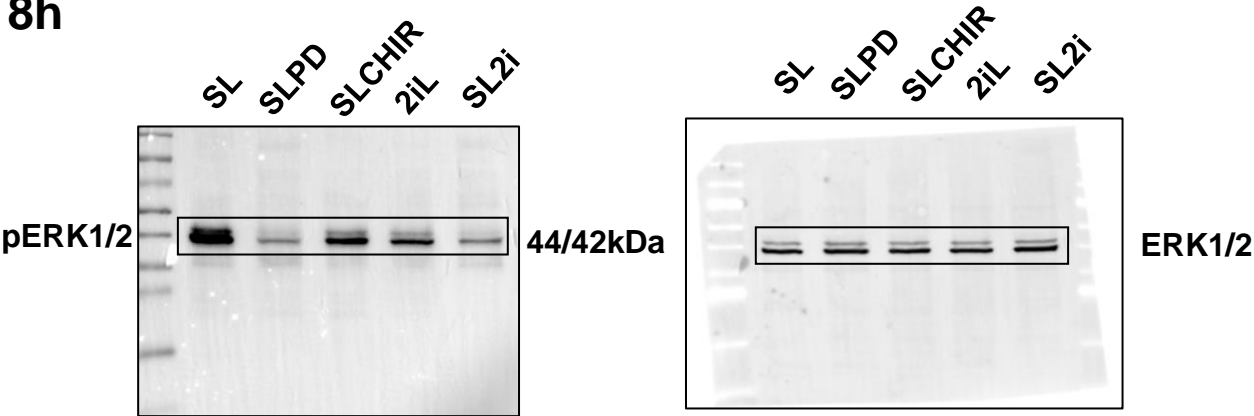

12h

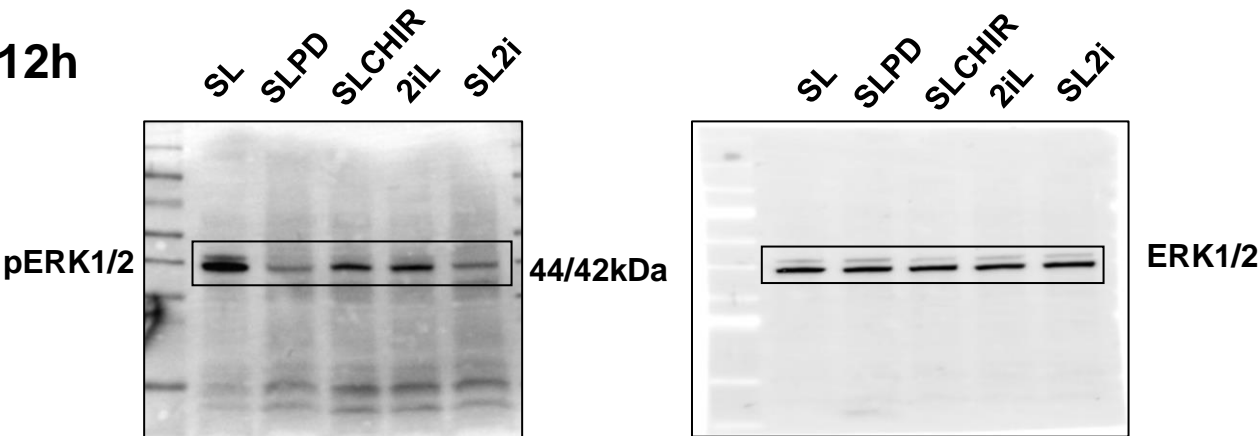

16h

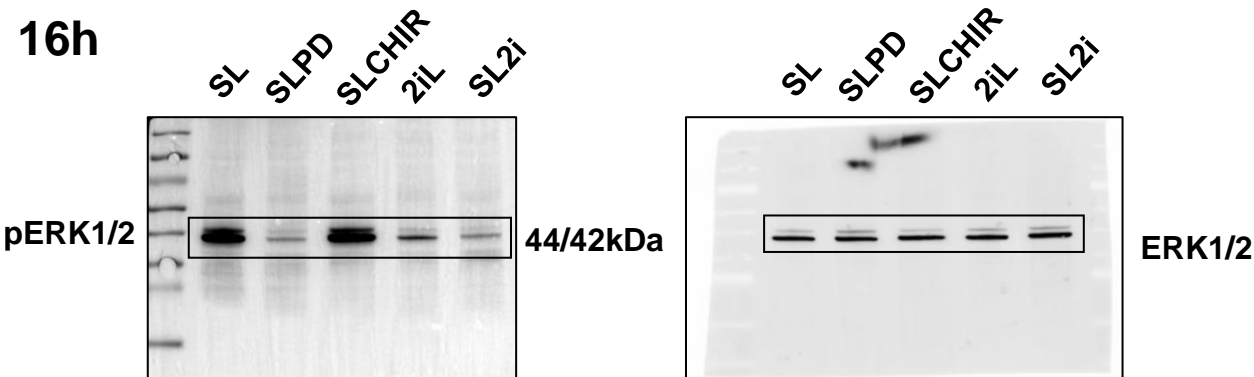

24h

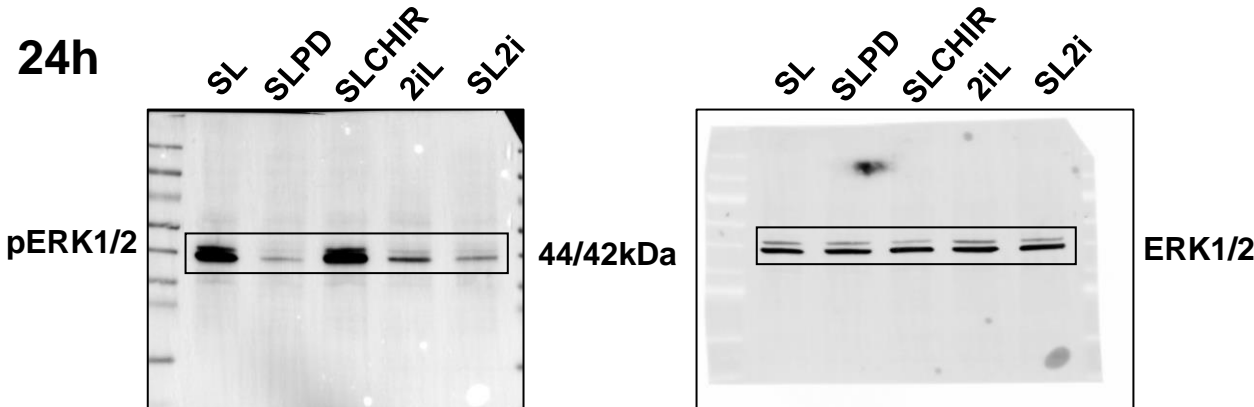

Figure1H

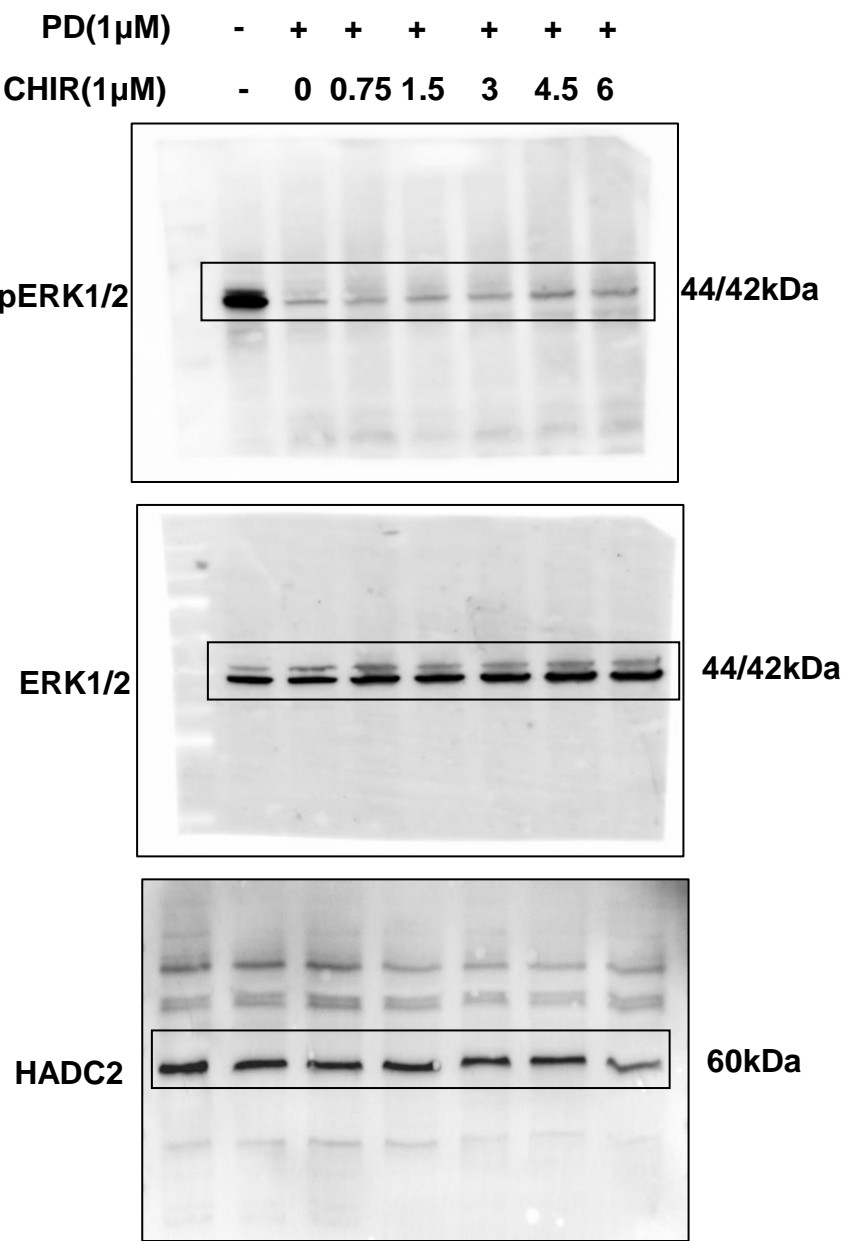

Figure1I

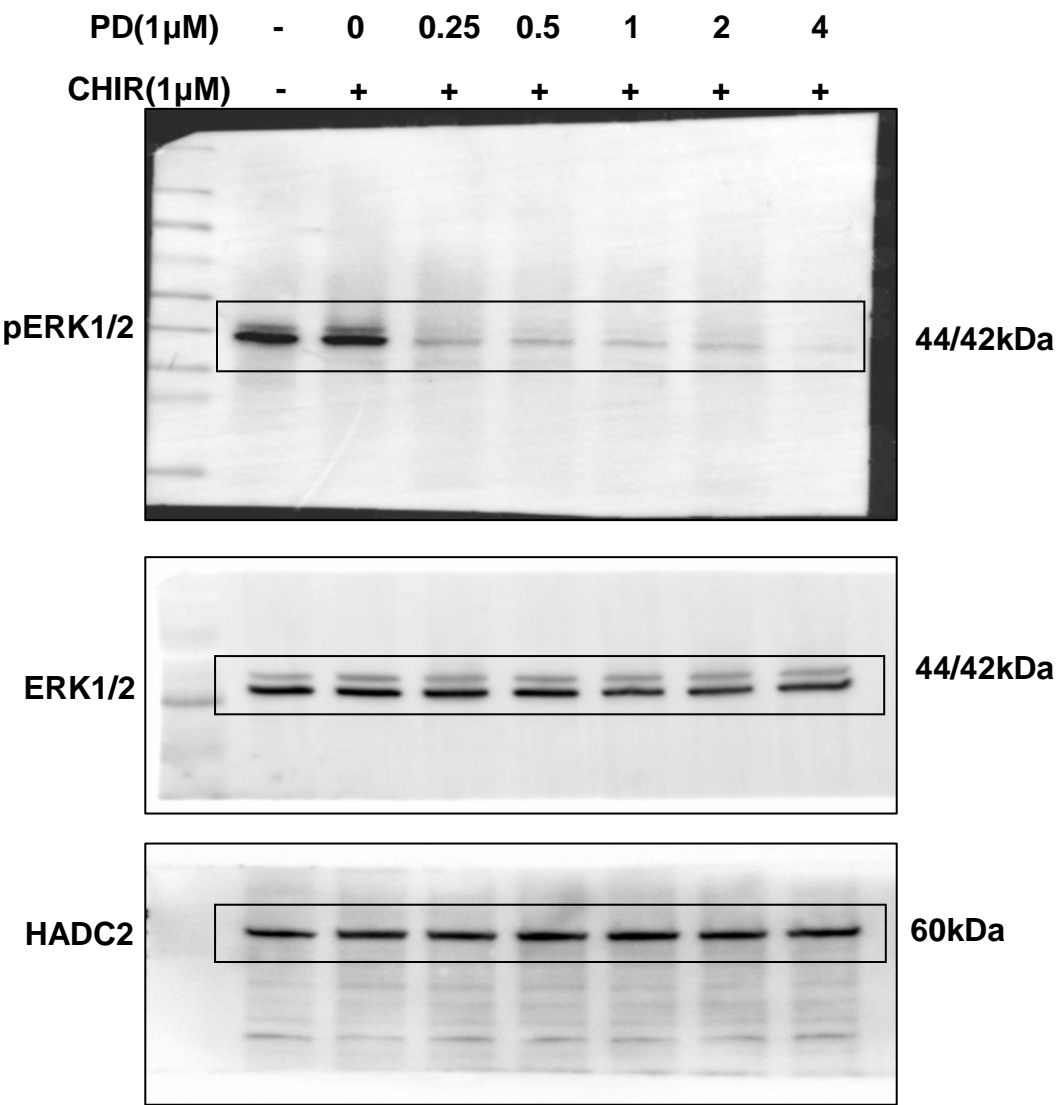

Supplement: Supplementary file 5 — Source Data for Figure 1 [file EMBR-23-e54421-s003.pdf]

## Figure2G

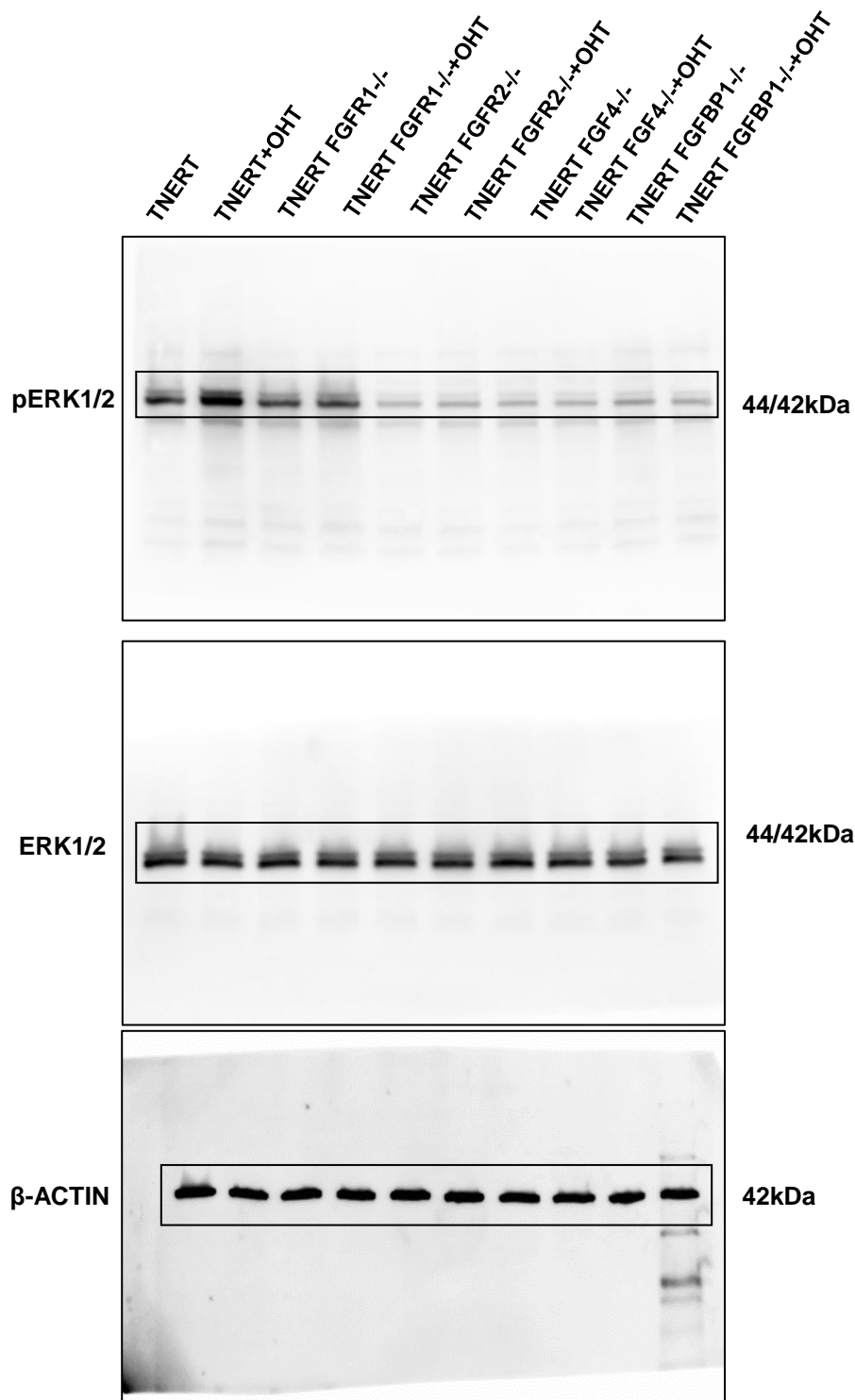

Supplement: Supplementary file 6 — Source Data for Figure 2 [file EMBR-23-e54421-s006.pdf]

Figure3E

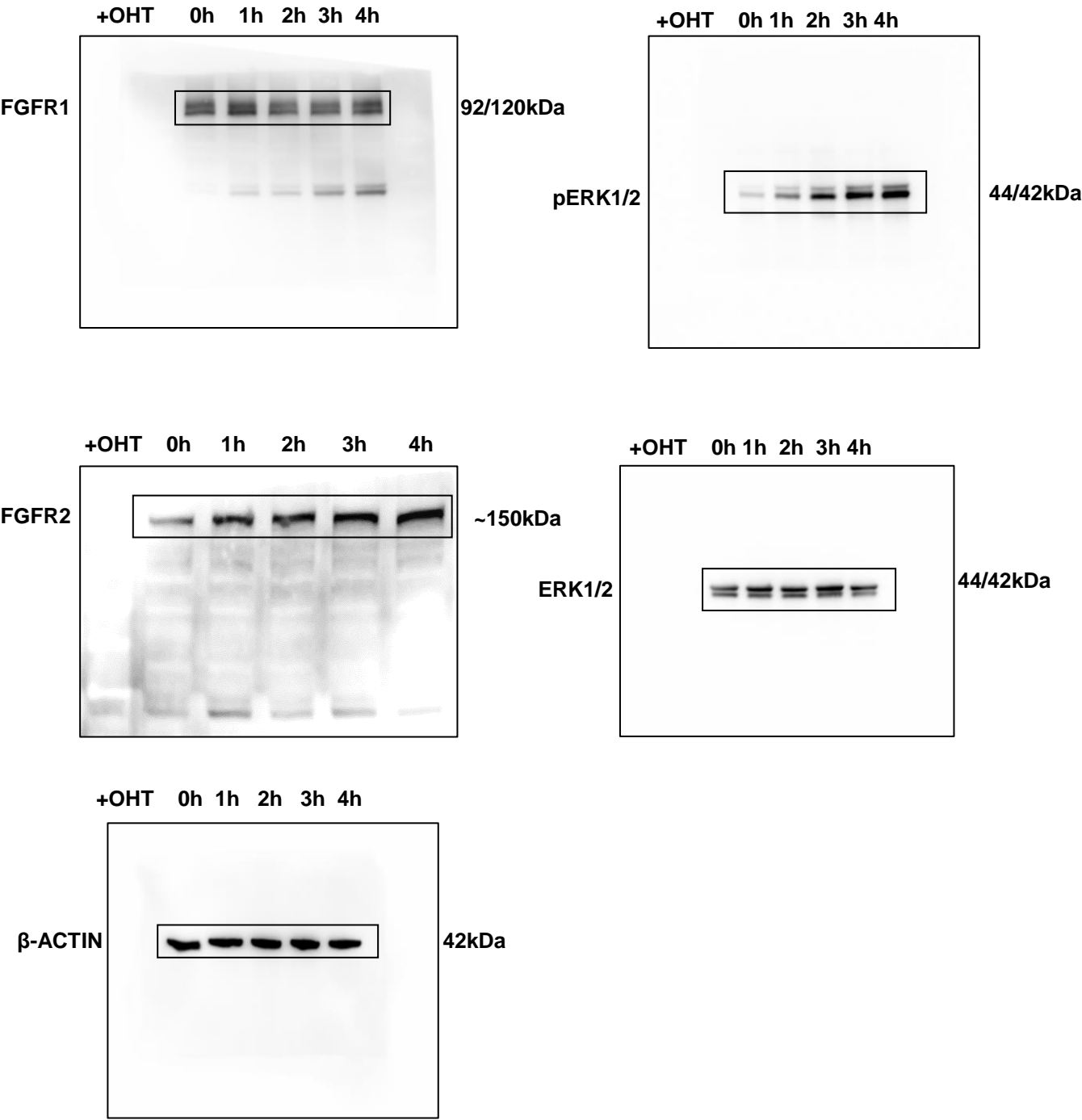

Supplement: Supplementary file 7 — Source Data for Figure 3 [file EMBR-23-e54421-s004.pdf]

Figure 5C

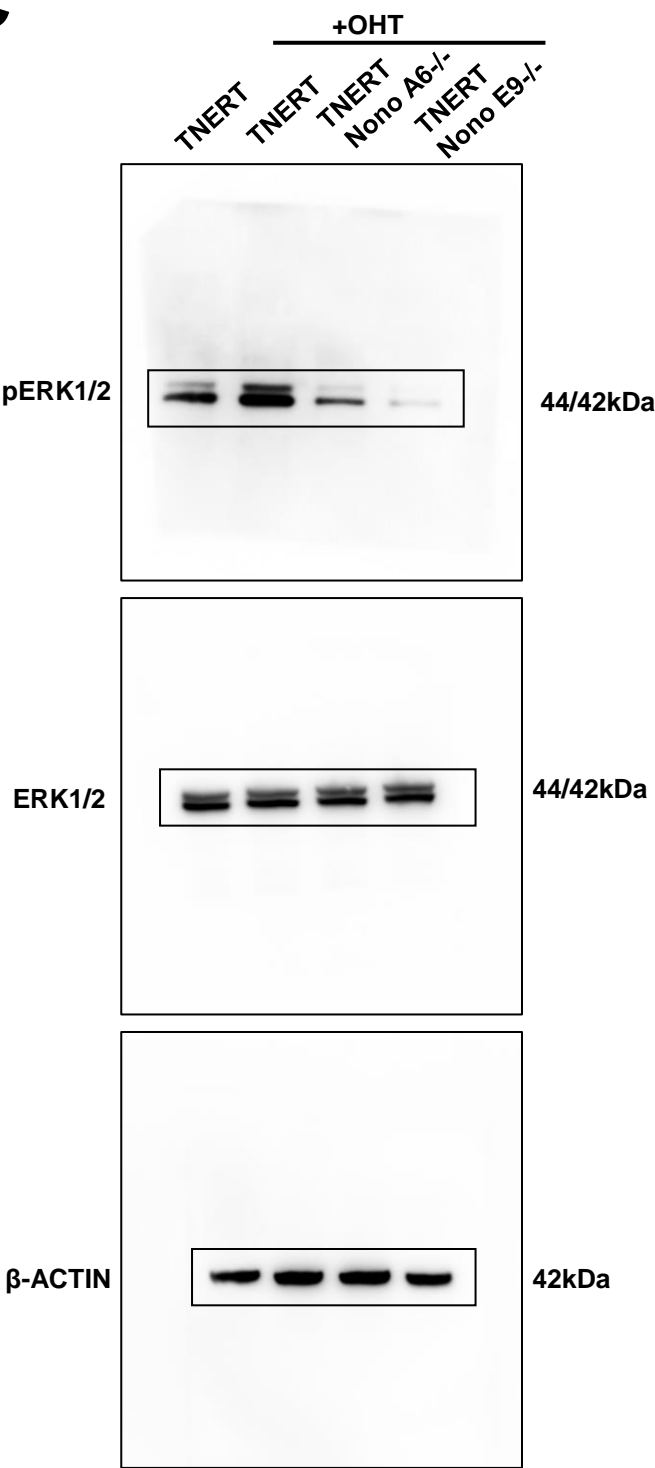

Figure 5D

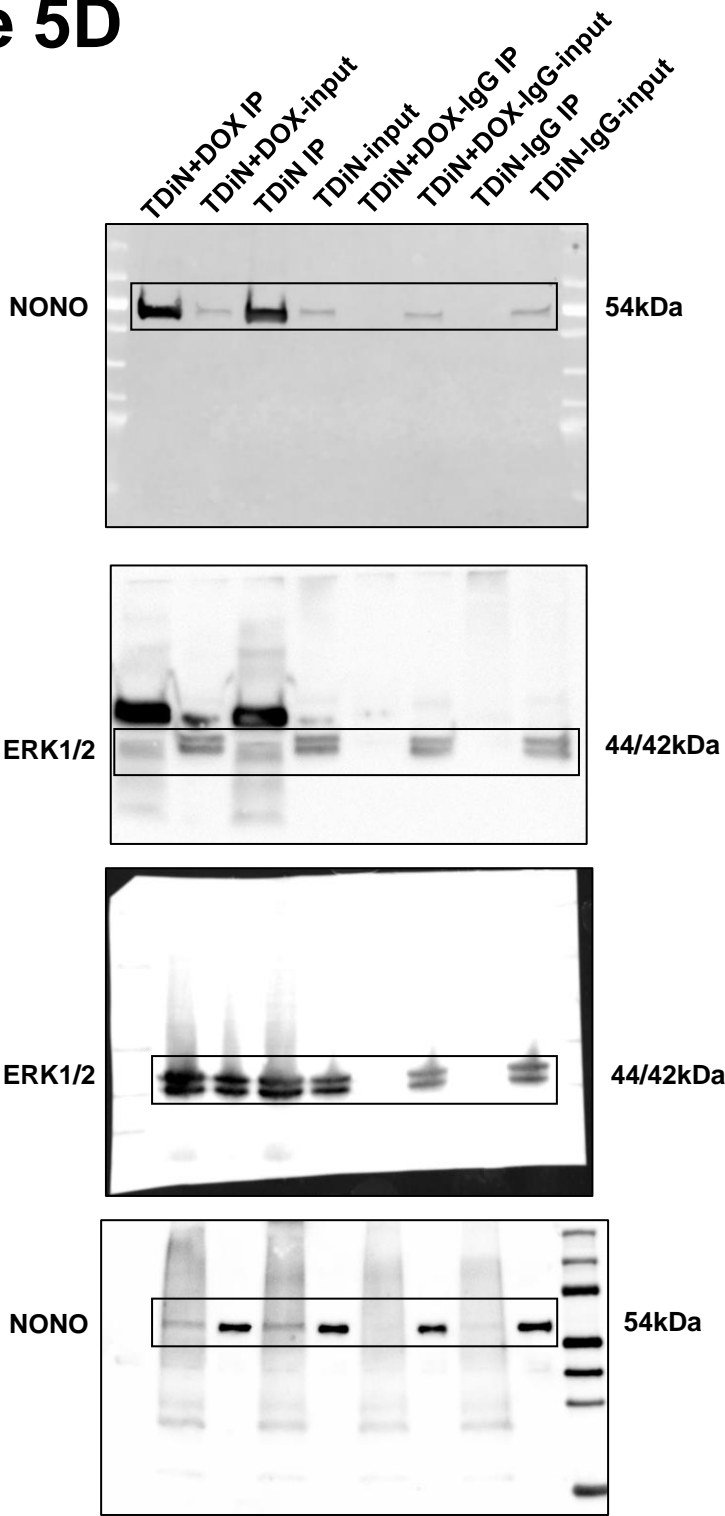

Figure 5E

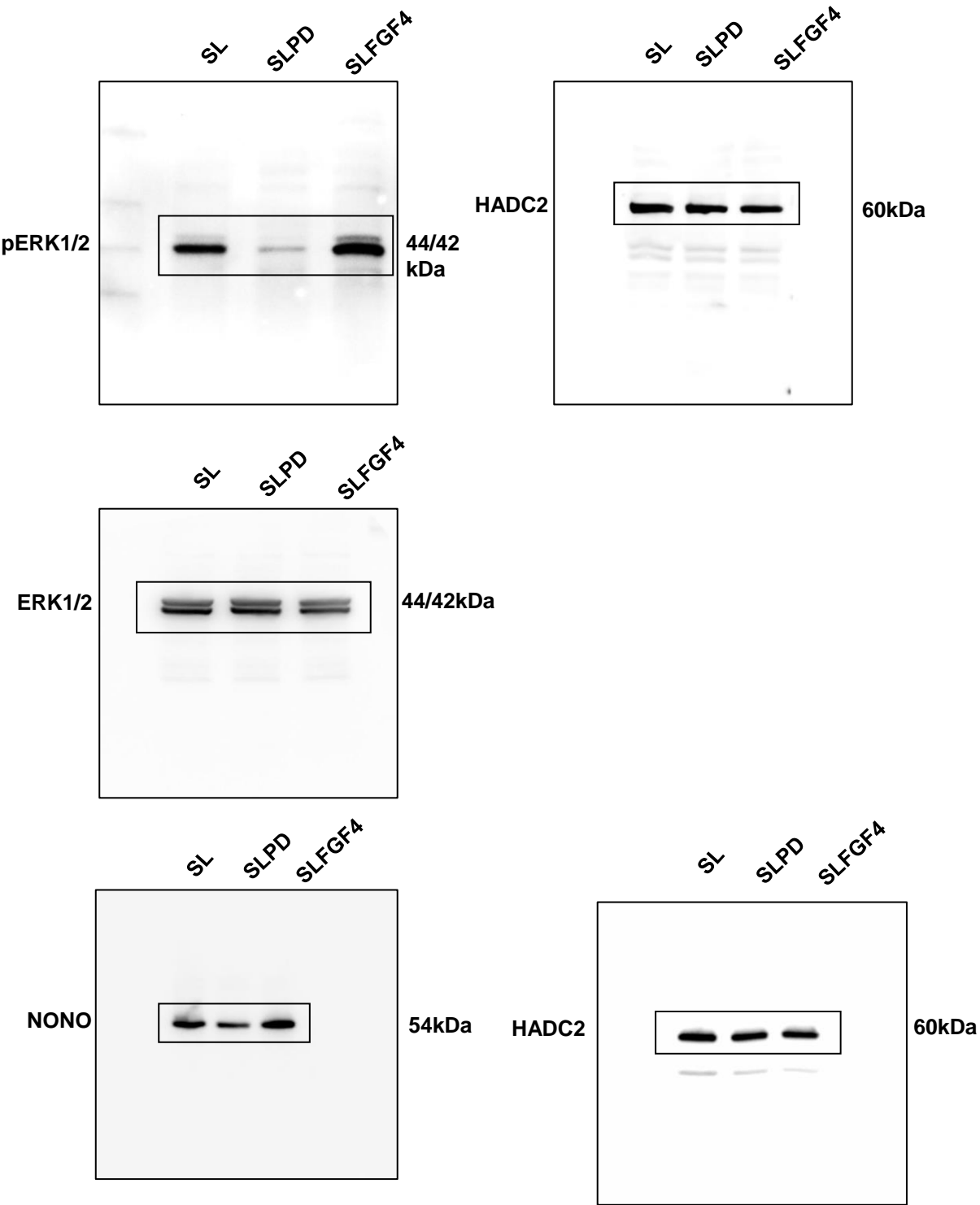

# Figure 5L

## SL

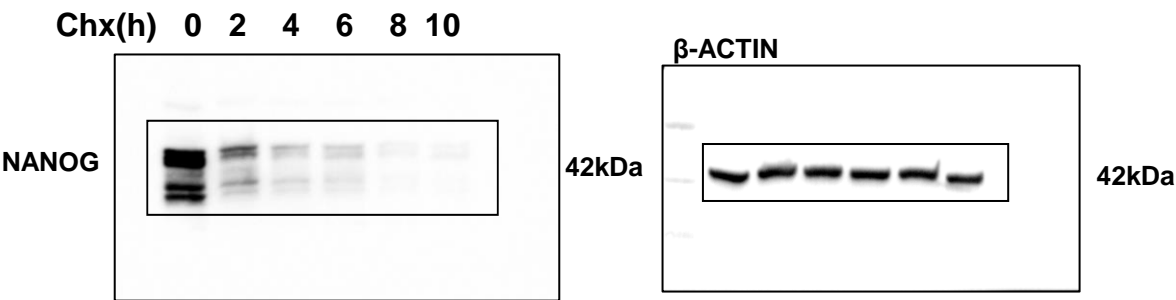

## SLPD

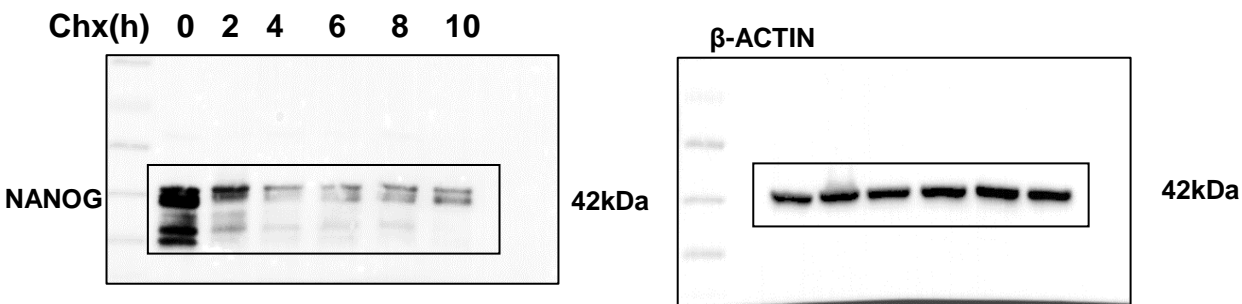

## SLFGF4

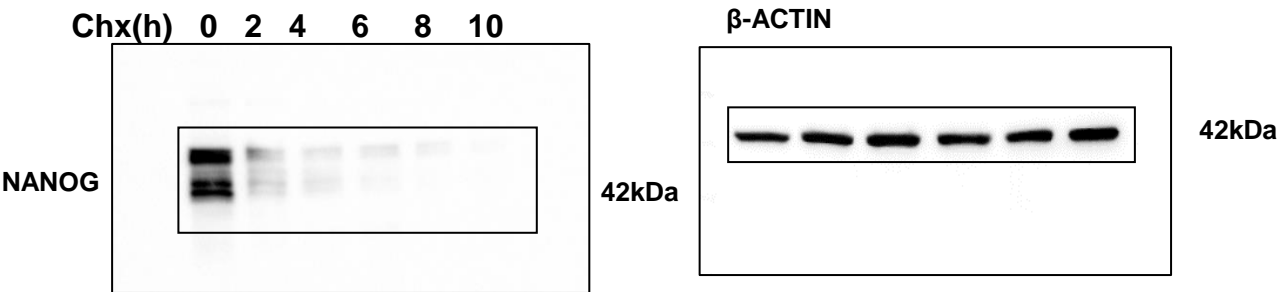

Supplement: Supplementary file 8 — Source Data for Figure 5 [file EMBR-23-e54421-s007.pdf]

# Figure 6C

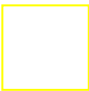 Indicates cropped area

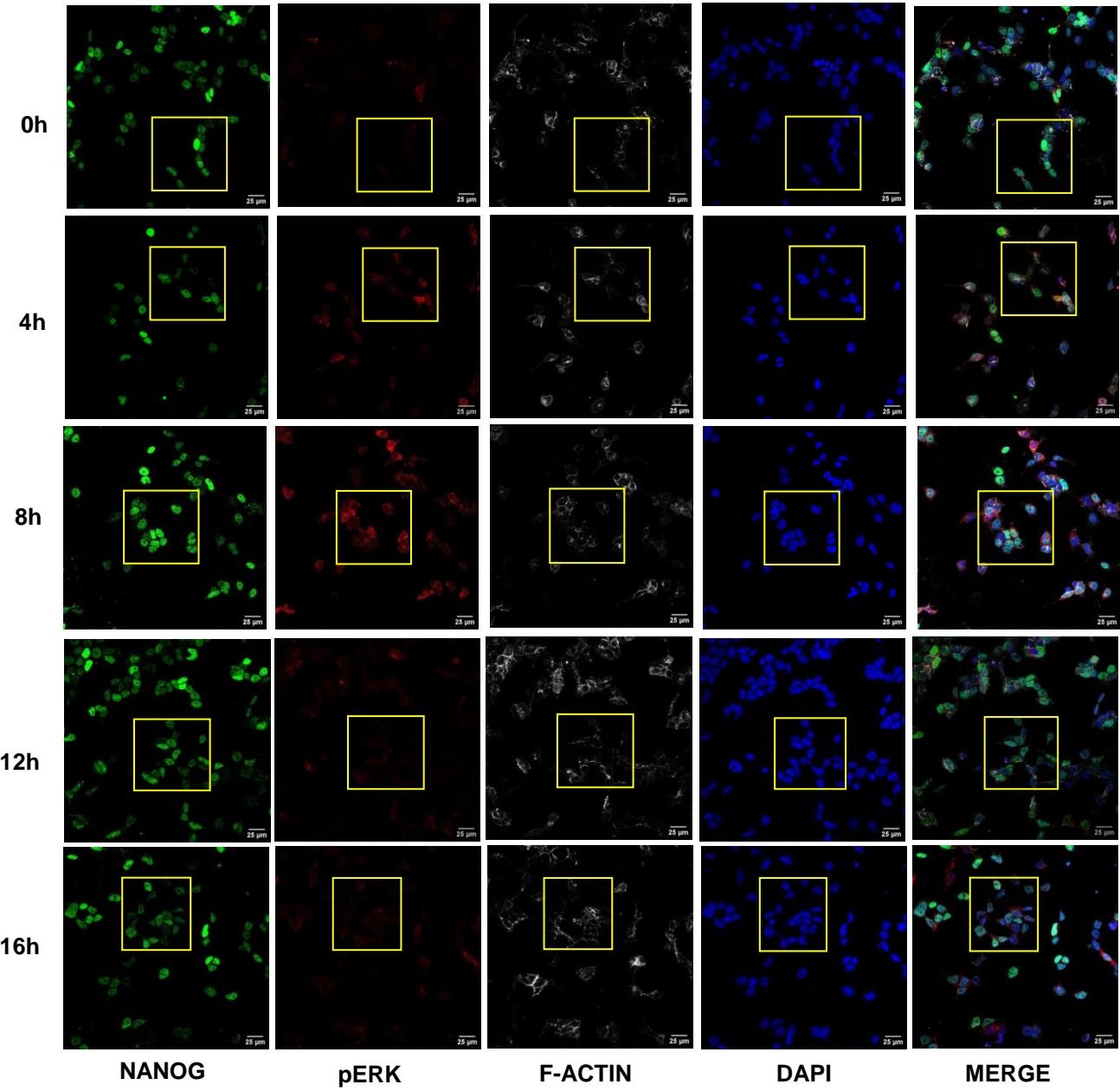

Figure 6D

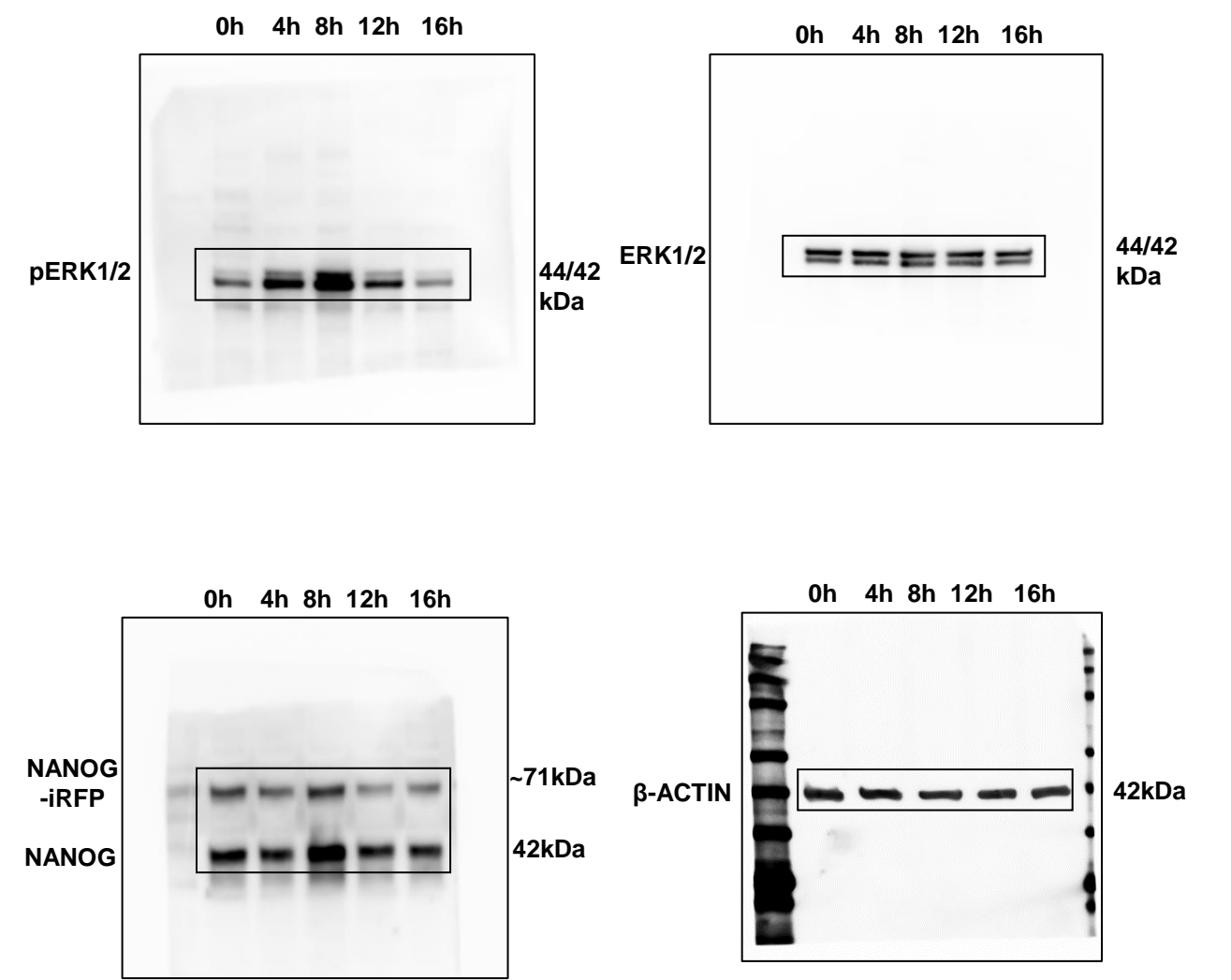

Supplement: Supplementary file 9 — Source Data for Figure 6 [file EMBR-23-e54421-s005.pdf]
