# Supplementary material for: A NANOG‐pERK reciprocal regulatory circuit regulates Nanog autoregulation and ERK signaling dynamics
Source: EMBO Rep. 2022 Sep 6;23(11):e54421. doi: 10.15252/embr.202154421 (PMC9638859; doi:10.15252/embr.202154421)
Supplement: Supplementary file 4 — PDF+ [file EMBR-23-e54421-s009.pdf]

Article

SOURCE DATA TRANSPARENT PROCESS OPEN ACCESS

EMBO reports

A NANOG-pERK reciprocal regulatory circuit regulates *Nanog* autoregulation and ERK signaling dynamics

Hanuman T Kale[1], Rajendra Singh Rajpurohit[1], Debabrata Jana[1], Vijay V Vishnu[1], Mansi Srivastava[1,2], Preeti R Mourya[1], Gunda Srinivas[1] & P Chandra Shekar[1,2,*]

Abstract

The self-renewal and differentiation potential of embryonic stem cells (ESCs) is maintained by the regulated expression of core pluripotency factors. Expression levels of the core pluripotency factor *Nanog* are tightly regulated by a negative feedback autorepression loop. However, it remains unclear how ESCs perceive NANOG levels and execute autorepression. Here, we show that a dose-dependent induction of *Fgfbp1* and *Fgfr2* by NANOG activates autocrine-mediated ERK signaling in *Nanog*-high cells to trigger autorepression. pERK recruits NONO to the *Nanog* locus to repress transcription by preventing POL2 loading. This *Nanog* autorepression process establishes a self-perpetuating reciprocal NANOG-pERK regulatory circuit. We further demonstrate that this reciprocal regulatory circuit induces pERK heterogeneity and ERK signaling dynamics in pluripotent stem cells. Collectively our data suggest that NANOG induces *Fgfr2* and *Fgfbp1* to activate ERK signaling in *Nanog*-high cells to establish a NANOG-pERK reciprocal regulatory circuit. This circuit regulates ERK signaling dynamics and *Nanog* autoregulation in pluripotent cells.

Keywords autoregulation; ERK; ESC; FGFR2; Nanog
Subject Categories Signal Transduction; Stem Cells & Regenerative Medicine
DOI 10.15252/embr.202154421 | Received 1 December 2021 | Revised 9 August 2022 | Accepted 17 August 2022 | Published online 6 September 2022
EMBO Reports (2022) 23: e54421

Introduction

Embryonic stem (ES) cells are characterized by long-term self-renewal and the potential to differentiate into all cell types of the germ layers. ES cells cultured in the presence of serum and LIF manifest transcriptional and functional heterogeneity. The heterogeneous expression of transcription factors like *Nanog*, *Rex1*, *Stella*, *Esrrb*, *Klf4*, and *Tbx3* determine differential fate choice (Chambers *et al*, 2007; Hayashi *et al*, 2008; Toyooka *et al*, 2008; van den Berg *et al*, 2008; Kalmar *et al*, 2009; Niwa *et al*, 2009; Festuccia *et al*, 2012; Filipczyk *et al*, 2013; Torres-Padilla & Chambers, 2014). The core pluripotency factor, *Nanog* was identified as a factor conferring LIF-independent self-renewal to ES cells by inhibiting differentiation (Chambers *et al*, 2003; Mitsui *et al*, 2003). ERK signaling represses *Nanog* to induce heterogeneous and monoallelic expression of *Nanog* in ESC (Hamazaki *et al*, 2006; Chambers *et al*, 2007; Miyanari & Torres-Padilla, 2012). ERK signaling drives differentiation and affects the self-renewal of ESC (Lanner & Rossant, 2010). The function of ERK is predominately restricted to cytoplasmic signaling cascades. ERK can also interact with transcription machinery like POL2 and NONO (a bivalent domain factor) to directly regulate transcription (Tee *et al*, 2014a; Ma *et al*, 2016a). ESC can be efficiently derived by inhibition of MEK1/2 and GSK3β with small molecules PD0325901 and CHIR99021, respectively. This culture condition is referred to as 2iL. The heterogeneous expression of *Nanog* is abolished in 2iL. The expression of *Nanog* is restricted in ES cells to ensure their potential to differentiate by negative feedback autorepression and other repressive mechanisms (Hamazaki *et al*, 2006; Pereira *et al*, 2006; Mora-Castilla *et al*, 2010; Fidalgo *et al*, 2011, 2012; Navarro *et al*, 2012; Kim *et al*, 2014). Among the multiple mechanisms that regulate *Nanog*, which mechanisms are utilized by the pluripotent cells to restrict *Nanog* by autorepression remain unknown. Although *Nanog* autorepression was shown to operate independently of OCT4/SOX2 (Navarro *et al*, 2012) and dependent on ZFP281 (Fidalgo *et al*, 2012) it is unclear how the NANOG protein levels are perceived by cells to trigger autorepression.

Here, we show that ERK signaling is essential for *Nanog* autorepression. NANOG induces *Fgfr2* and *Fgfbp1* exclusively in the high-*Nanog* ESCs to trigger feedback repression by autocrine-mediated activation of ERK signaling. We show that pERK recruits NONO to the *Nanog* locus to repress *Nanog* transcription by affecting POL2 loading. We show that the *Nanog* autoregulation process results in a self-perpetuating NANOG-pERK reciprocal regulatory loop. Our results establish that the NANOG-pERK reciprocal regulatory loop is the basis of ERK signaling dynamics and pERK heterogeneity in pluripotent stem cells. Together our data show that the NANOG-

1 CSIR-Centre for Cellular and Molecular Biology, Hyderabad, India
2 Academy of Scientific and Innovative Research (AcSIR), Ghaziabad, India
*Corresponding author. Tel: +91 4027192890; E-mail: csp@ccmb.res.in

© 2022 The Authors. Published under the terms of the CC BY 4.0 license.

pERK axis may not merely be viewed as a mechanism to regulate *Nanog* but also a mechanism by which ERK dynamics and heterogeneity is induced in pluripotent cells.

Results

Residual MEK1/2 activity in the ground state prevents complete derepression of *Nanog*

Transcriptional regulation is the major mechanism regulating *Nanog* heterogeneity, biallelic expression, and autorepression (Fidalgo *et al*, 2012). To uncouple the influence of MEK1/2 and GSK3β on *Nanog* expression in naïve state of pluripotency, we analyzed the activity of *Nanog* promoter reported by GFP in TβC44Cre6 cell line (Chambers *et al*, 2007) in combinations of MEK1/2 and GSK3β inhibitors. TβC44Cre6 cell line is *Nanog* null ESC in which a Neomycin resistance cassette is knocked in into one allele of *Nanog* and GFP into another allele. *Nanog* expression was increased above the basal level (SL) in all treatments. *Nanog* promoter activity was higher in SLPD relative to 2iL (Fig 1A). To analyze NANOG protein dynamics, we generated a NiRFP2A cell line with both endogenous alleles of Nanog expressing NANOG-IRFP fusion protein (Fig EV1A). Higher NANOGiRFP in SLPD (Fig 1B), confirmed the highest induction of *Nanog* transcript and protein in SLPD. To dismiss the interference of genetic modifications in the *Nanog* locus on its expression (Faddah *et al*, 2013), we analyzed its expression in E14Tg2a cells. The *Nanog* transcript (Fig 1C), transcriptional activity (Fig 1D), and protein (Fig 1E) were highest in SLPD, unlike OCT4 protein which changed very little (Figs 1C–E and EV1B). SLPD and 2iL contain 1 μM PD, higher *Nanog* expression in SLPD indicated MEK inhibition alone increased *Nanog* expression more than the combined inhibition of MEK1/2 and GSK3β in 2iL/SL2i. We analyzed pERK to investigate the possible modulation of MEK1/2 activity by GSK3β (Yun *et al*, 2005). The pERK remained undetectable for up to 4 h in SLPD and 2iL/SL2i. It gradually increased in 2iL after 8 h but remained undetectable in SLPD (Figs 1F and EV1C). The pERK in SLCHIR and 2iL significantly exceeded SL and SLPD, respectively, by 24 h (Figs 1G and EV1D), suggesting a long-term CHIR treatment enhanced MEK1/2 activity in ESCs. Further, the PD and CHIR dose-responsive experiments confirmed that the pERK positively correlated with the CHIR concentrations (Figs 1H and I, and EV1E–H). Collectively, our data demonstrate that *Nanog* attains higher expression in MEK1/2 inhibition than in 2iL. GSK3β activity negatively modulates MEK1/2 activity and its inhibition by CHIR increases pERK in 2iL over time.

FGF autocrine signaling pathway components are essential for *Nanog* autoregulation

Nanog autorepression was shown to function in 2iL (Navarro *et al*, 2012). We show residual MEK activity persists in 2iL and *Nanog* expression is lower in 2iL than SLPD. We asked if all repressive mechanisms including the *Nanog* autorepression are abolished in SLPD. We generated two NANOG restoration systems by integrating Flag-Avi-NANOG-ER^{T2} and a Doxycycline-inducible Flag-Avi-NANOG transgene in Tβc44Cre6 (Chambers *et al*, 2007) to derive the TNERT and TDiN cell lines, respectively (Figs 2A and EV2A and B). The repression of endogenous *Nanog*:GFP upon induction of transgenic NANOG by OHT/Dox is a functional readout of *Nanog* autoregulation. *Nanog*:GFP was repressed in OHT/Dox-induced TNERT/TDiN in all treatments except SLPD (Figs 2A and EV2C and D). The data from distinct induction systems conclusively establish an essential role of MEK1/2 in *Nanog* autoregulation.

FGF signaling is the predominant inducer of MEK/ERK in pluripotent cells (Kunath *et al*, 2007; Lanner & Rossant, 2010) we investigated its role in autoregulation. NANOG induction failed to repress *Nanog*:GFP in the presence of FGFR inhibitor (SU5402) in TNERT and TDiN, suggesting an essential role of FGFRs (Figs 2B and EV2E). FGFR1, FGFR2, and FGF4 are major receptors and ligands of FGF signaling in early embryos (Kang *et al*, 2017; Molotkov *et al*, 2017) FGFBP1 is a carrier protein that enhances FGF signaling. We analyzed single cell seq data of mouse preimplantation embryos from Park *et al* (2015) *Fgfbp1* expression was induced in the early blastocyst and increased in later stages (Fig 2C). We deleted *Fgfr1*, *Fgfr2*, *Fgf4*, and *Fgfbp1* in TNERT cells to analyze their role in autoregulation (Fig 2D and EV2F–I). Except in $TNERTFgfr1^{-/-}$, *Nanog*:GFP and its transcript were not repressed in $TNERTFgfr2^{-/-}$, $TNERTFgf4^{-/-}$, and $TNERTFgfbp1^{-/-}$ cells upon OHT induction (Fig 2E and F). Consistent with these observations, the pERK levels in all mutants except $TNERTFgfr1^{-/-}$ were lower than TNERT and did not significantly increase upon OHT treatment (Fig 2G). We observed induction of *Nanog*:GFP expression of different magnitudes in all mutant cell lines relative to TNERT. None of the mutants showed *Nanog*:GFP induction equivalent to SLPD suggesting a possibility of additional growth factor signaling pathways contributing to pERK activity in ESCs (Fig 2H). Collectively, our data suggest that FGF autocrine signaling and its components FGFR2, FGF4, and FGFBP1 are essential for *Nanog* autoregulation.

NANOG enhances the expression of FGFR2, FGF4, and FGFBP1

We analyzed the expression of FGF autocrine signaling components during the time course of OHT induction. *Fgf4*, *Fgfr2*, *Fgfr1*, and *Fgfbp1* transcripts were induced within 1–2 h (Fig 3A). Increased pre-mRNA indicated transcriptional activation of these genes (Fig 3B). ChIP-seq data analysis identified NANOG occupancy on *Fgf4*, *Fgfbp1*, *Fgfr1*, and *Fgfr2*, which was further enhanced in $Oct4^{+/-}$ cells that have higher NANOG (Fig EV3A) (Karwacki-Neisius *et al*, 2013a, Data ref: Karwacki-Neisius *et al*, 2013b). To analyze the dosage-dependent occupancy of NANOG on these genes, we generated EDiN cell line by introducing a Doxycycline-inducible Flag-Avi-NANOG transgene in E14Tg2a. ChIP-PCR confirmed NANOG occupancy on *Fgf4*, *Fgfbp1*, *Fgfr1*, and *Fgfr2*, which was further enhanced in PD (Fig 3C) and EDiN + Dox (Fig 3D) which express higher NANOG. The data suggest a dose-dependent occupancy of NANOG on the FGF signaling component genes. FGFR1, FGFR2, and pERK were significantly increased upon OHT induction in TNERT suggesting NANOG could induce FGFR1, FGFR2, and pERK (Figs 3E and EV3B). The strength of FGF signaling depends on facilitation by carrier proteins (Tassi *et al*, 2001), the affinity of ligands (Ornitz *et al*, 1996; Zhang *et al*, 2006) and subsequent subcellular trafficking of the FGFRs (Auciello *et al*, 2013; Francavilla *et al*, 2013) The induction of NANOG enhanced FGFR2 on the cell surface (Fig 3F), unlike the FGFR1 (Fig EV3C) suggesting NANOG specifically enhances FGFR2. Intriguingly, FGFR2 expression exhibited a

 © 2022 The Authors

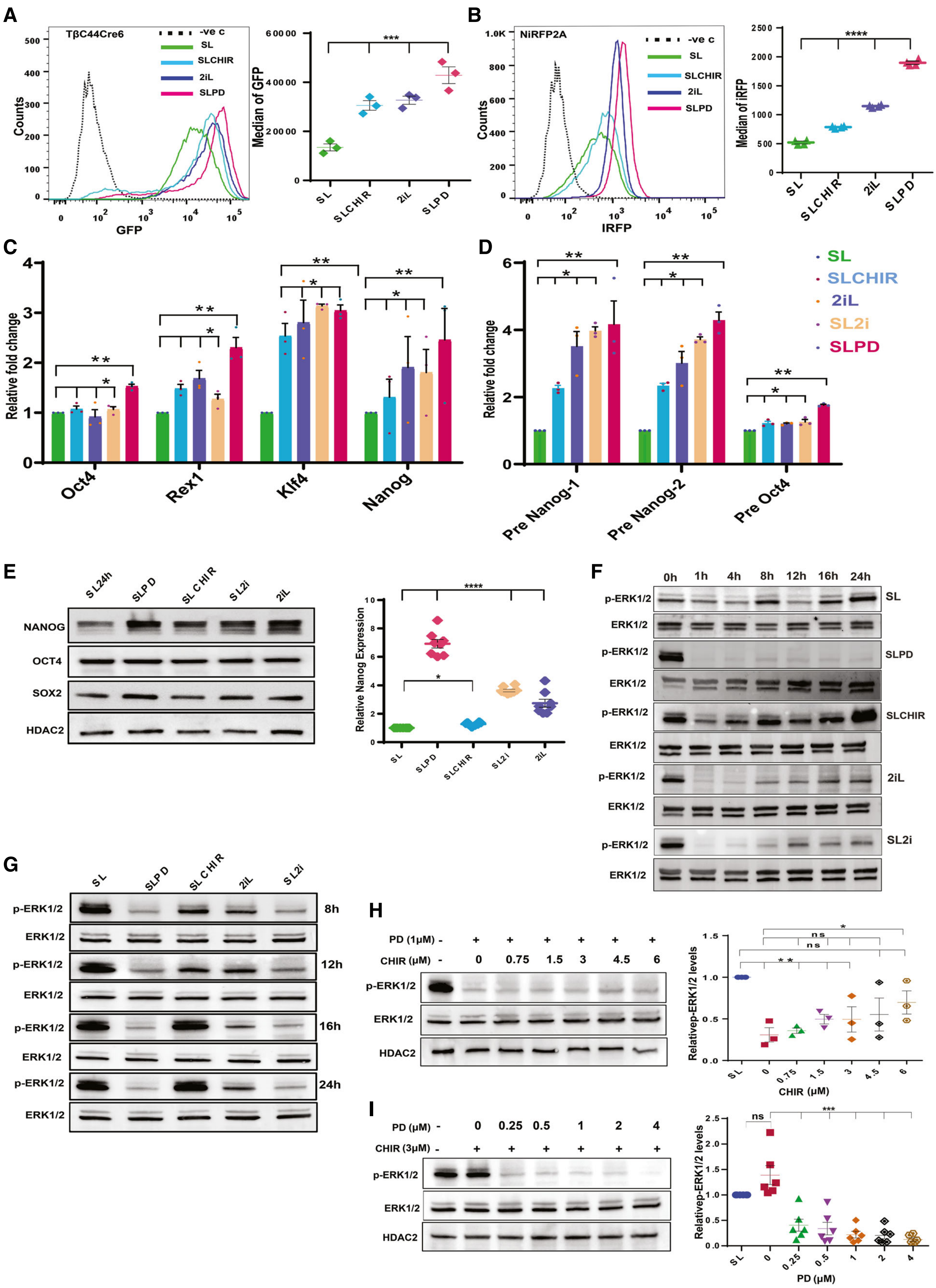
A
TβC44Cre6
-ve c
SL
SLCHIR
2iL
SLPD
Counts
GFP
Median of GFP
***
B
NiRFP2A
IRFP
Median of iRFP
****
C
Relative fold change
Oct4
Rex1
Klf4
Nanog
D
Pre Nanog-1
Pre Nanog-2
Pre Oct4
SL
SLCHIR
2iL
SL2i
SLPD
E
NANOG
OCT4
SOX2
HDAC2
Relative Nanog Expression
F
0h 1h 4h 8h 12h 16h 24h
p-ERK1/2
ERK1/2
G
8h
12h
16h
24h
H
PD (1μM)
CHIR (μM)
Relativep-ERK1/2 levels
CHIR (μM)
I
PD (μM)
CHIR (3μM)
PD (μM)

Figure 1.

◀ **Figure 1. Residual MEK1/2 activity in the ground state prevents complete derepression of *Nanog*.**

A (left) FACS profiles of TβC44Cre6 cultured in indicated conditions for three passages. TβC44Cre6 is a *Nanog* null cell line, where *β-geo* cassette is inserted into one allele and GFP into another allele of the *Nanog* gene. The cells were cultured in Serum + LIF (SL) in the presence of the 1 μM MEK1/2 inhibitor—PD0325901 (SLPD) or 3 μM GSK3β inhibitor—CHIR99021 (SLCHIR) or in serum-free media—N2B27 with PD0325901, CHIR99021, and LIF (2iL). The dotted line shows the FACS profile of unstained E14Tg2a cells used as negative control (−ve c). (right) *Nanog*:GFP population median of TβC44Cre6 (n = 3).
B (left) FACS profile of NANOG-iRFP protein in NiRFP2A cells cultured in indicated conditions for three passages. The dotted line represents the FACS profile of unstained E14Tg2a cells used as negative control (−ve c). (right) *NANOG*-iRFP population median of NiRFP2A (n = 4).
C RT-qPCR of pluripotency factors in indicated conditions (SL2i = SL + PD0325901 + CHIR99021) (n = 3).
D RT-qPCR analysis of pre- mRNA of *Nanog* and *Oct4* (n = 3).
E (left) Western blot of NANOG, OCT4, and SOX2. (right) Relative NANOG levels as estimated by densitometry (n = 8). NANOG was nearly 7-fold more in PD, which is twice that of 2iL/SL2i.
F Western blot of pERK and ERK at 0, 1, 4, 8, 12, 16, and 24 h after media change in indicated treatments (n = 3).
G Western blot of pERK and ERK in SLPD, SLCHIR, 2iL, and SL2i after 8, 12, 16, and 24 h of culture relative to SL, where the cells in SL were harvested 24 h after the media change (n > 3).
H (left) Western blot of pERK and ERK in 1 μM PD and increasing concentrations of CHIR in serum-free N2B27 media. (right) Relative pERK levels (n = 3).
I (left) Western blot of pERK and ERK in 3 μM CHIR and increasing concentrations of PD in serum-free N2B27 media. (right) Relative pERK levels (n = 6).

Data information: $n \geq 3$ biological replicates (each dot represents a biological replicate). Data are presented as mean ± SEM in A–E, H, and I. *$P < 0.05$, **$P < 0.01$, ***$P < 0.001$, ****$P < 0.0001$ and ns = not significant (paired two-tailed Student's t-test).
Source data are available online for this figure.

negatively skewed bimodal distribution resembling *Nanog* expression (Chambers *et al*, 2007) (Fig 3F). The NANOG induction increased FGF4 and FGFBP1 secretion by TNERT (Figs 3G and H, and EV3D). Collectively the data shows that increased NANOG enhances FGFR2 on the cell surface, and secretion of FGF4 and FGFBP1 to intensify the FGF autocrine signaling. NANOG induces and enhances FGF autocrine signaling through FGFR2 to execute *Nanog* autoregulation.

Nanog autoregulation is a non-cell autonomous process mediated by FGF autocrine/paracrine signaling

Nanog autorepression is suggested to operate by a cell-autonomous process through intracellular proteins NANOG, ZFP281, and NURD complex (Fidalgo *et al*, 2012). Non-cell autonomous function of *Nanog* in the induction of primitive endoderm (Messerschmidt & Kemler, 2010; Frankenberg *et al*, 2011) and essentiality of secreted proteins FGF4 and FGFBP1 in autoregulation prompted us to investigate non-cell autonomous mechanisms. We assessed the ability of conditioned media from OHT-induced TNERT cells, to repress *Nanog*:GFP in Tβc44Cre6 lacking *Nanog* (Fig 3I). The conditioned media was sufficient to repress the *Nanog*:GFP (Figs 3J and EV3E), suggesting that autoregulation predominately operates via non-cell autonomous mechanisms besides the mechanism proposed earlier (Fidalgo *et al*, 2012). NANOG seems to be essential for triggering autoregulation through FGF autocrine signaling but does not participate in repression. Further, the repression of *Nanog*:GFP in OHT treated TNERTZfp281$^{-/-}$ cell line relative to untreated (Fig EV3F) suggests that ZFP281 is dispensable for *Nanog* autoregulation (Fig 3K).

To evaluate whether FGF4 secretion was the causative factor of *Nanog* autoregulation in the conditioned media, we treated Tβc44Cre6 with conditioned media from cells with loss or gain of FGF4. The conditioned media from an E14Tg2a cell line overexpressing FGF4 or supplementation of FGF4 (50 ng/ml) could repress *Nanog*:GFP. Conversely, the conditioned media from OHT-induced TNERTFgf4$^{-/-}$ cells failed to repress *Nanog*:GFP, suggesting FGF4 is the key secreted factor essential for *Nanog* autoregulation (Figs 3L and EV3G). The ELISA analysis confirmed the secretion and accumulation of FGF4 and FGFBP1 in the conditioned media (Fig EV3H–K). Collectively, our data establish that *Nanog* autoregulation is a non-cell autonomous process triggered by NANOG by augmenting FGF autocrine signaling.

NANOG-induced FGFR2 triggers autoregulation predominately in ES cell populations with higher *Nanog* expression

Nanog autoregulation was proposed to restrict NANOG levels within limits to retain the differentiation potential (Fidalgo *et al*, 2012; Navarro *et al*, 2012). Autoregulation is expected to operate only in *Nanog*-high cells in a population. To evaluate this logic, we used TDiN cell lines with different induction levels of NANOG (Fig EV4A). The strength of *Nanog* autoregulation was found to be dependent on the NANOG levels and was completely abolished in TDiN clones with low NANOG (Fig EV4B and C). Further, the *Nanog*:GFP was repressed only in 10% population of the TNERT with the highest *Nanog* expression but not in the lowest 10% (Fig 4A). Our experiments conclude that *Nanog* autoregulation predominately operates in a subpopulation of cells with higher *Nanog*.

FGF4 and FGFBP1 are secreted proteins, hence cannot distinguish between the *Nanog*-high and low cells in culture. Whereas FGFRs are essential for autoregulation and are retained on the cells, we asked if FGFRs distinguish *Nanog*-high cells from low cells in a population. We analyzed the correlation between the expression of FGFR1, FGFR2, and NANOG in E14Tg2a by FACS. FGFR2 and FGFR1 showed a fair correlation with NANOG, which was further enhanced for FGFR2-NANOG in SLPD (r = 0.56), whereas increased moderately for FGFR1-NANOG (r = 0.46) in SLPD (Fig 4B) where NANOG levels are higher. These observations were further strengthened by western blot analysis which showed increased NANOG, FGFR2, and FGFR1 in SLPD (Fig EV4D). These data suggested FGFR2 expression levels correlate and respond to NANOG concentration in the cells more than FGFR1. FACS analysis showed high FGFR2 in the 10% NANOG high population and lower FGFR2 in the 10% NANOG low population (Fig 4C). We analyzed the NANOG binding sequences in the *Fgfr2* locus. Two NANOG binding regions (NBR) with multiple NANOG binding sequences were identified in the *Fgfr2* locus from the ChIP-seq (Data ref: Karwacki-Neisius

 © 2022 The Authors

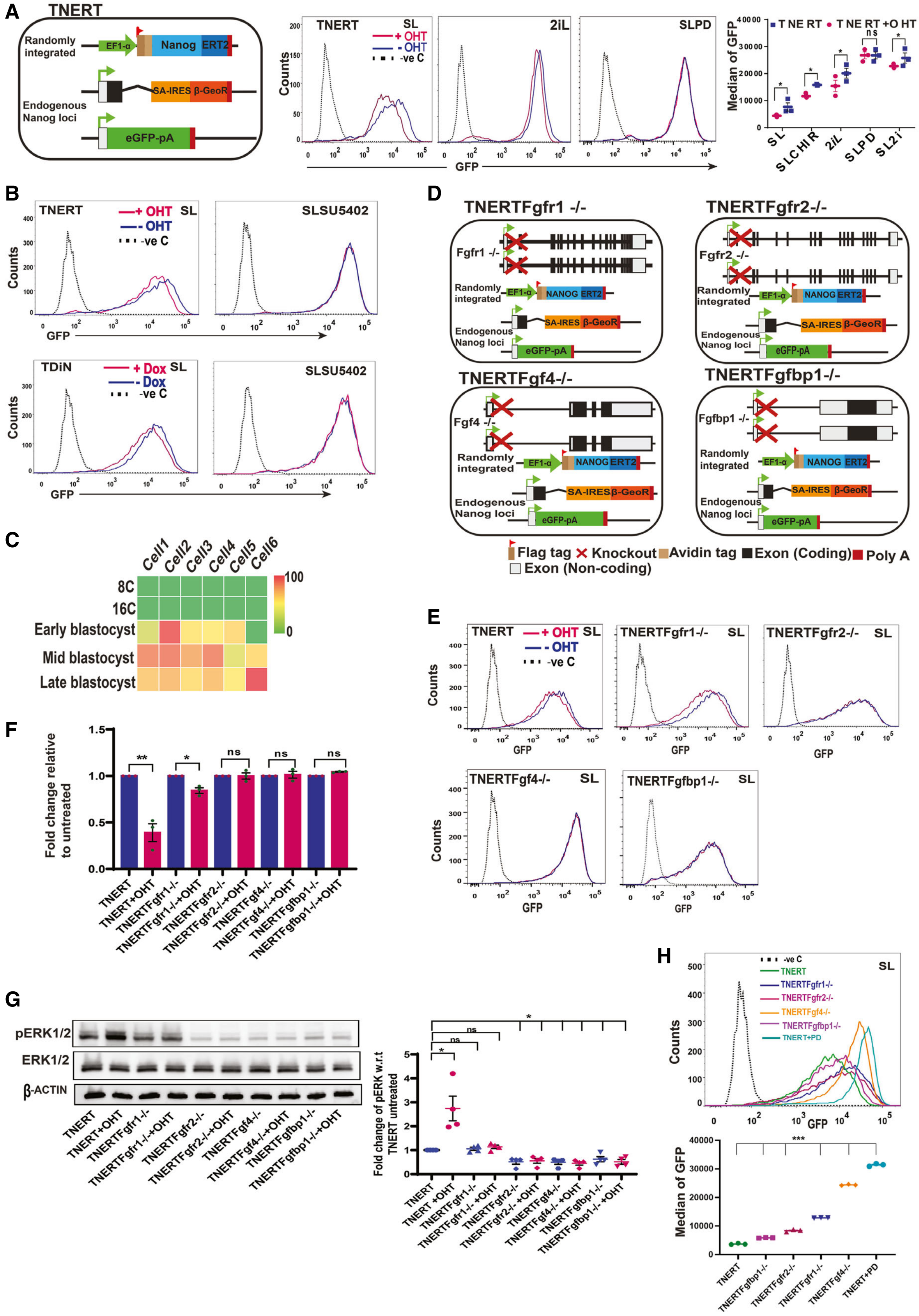
A
TNERT
Randomly integrated
EF1-α
Nanog
ERT2
Endogenous Nanog loci
SA-IRES β-GeoR
eGFP-pA
Counts
GFP
+ OHT
- OHT
-ve C
SL
2iL
SLPD
Median of GFP
SLCHIR
SL2i
B
SLSU5402
TDiN
+ Dox
- Dox
C
Cell1
Cell2
Cell3
Cell4
Cell5
Cell6
8C
16C
Early blastocyst
Mid blastocyst
Late blastocyst
D
TNERTFgfr1 -/-
TNERTFgfr2-/-
TNERTFgf4-/-
TNERTFgfbp1-/-
Flag tag
Knockout
Avidin tag
Exon (Coding)
Poly A
Exon (Non-coding)
E
F
Fold change relative to untreated
G
pERK1/2
ERK1/2
β-ACTIN
Fold change of pERK w.r.t TNERT untreated
H
TNERT+PD

Figure 2.

Figure 2. FGF autocrine signaling pathway components are essential for *Nanog* autoregulation.

A (left) Schematic depiction of Tamoxifen (OHT) inducible TNERT cell line. TNERT and TDiN (Fig EV2A and B) are similar to NERTc3 and 44iN (Navarro *et al*, 2012), where the NANOG function is reinstated by 4-Hydroxytamoxifen (OHT) or Doxycycline, respectively, and endogenous *Nanog* gene activity is reported by GFP. (Middle) FACS profile of TNERT treated with OHT (red) or no OHT (blue). The dotted line represents the FACS profile of unstained E14Tg2a cells used as negative control (−ve c). (Right) *Nanog*:GFP population median of TNERT ($n = 3$).

B FACS profiles of TNERT, and TDiN treated with 2 μM SU5402, with OHT/Doxycycline (red) or no OHT/Doxycycline (blue) ($n = 3$). The dotted line represents the FACS profile of unstained E14Tg2a cells used as negative control (−ve c).

C Heat map representing transcript levels (FPKM) of *Fgfbp1* from 8-cell to blastocyst stage analyzed from the single-cell sequencing data.

D Schematic depiction of TNERTFgfr1$^{-/-}$, TNERTFgfr2$^{-/-}$, TNERTFgf4$^{-/-}$, and TNERTFgfbp1$^{-/-}$ cell lines, which are derivatives of TNERT where *Fgfr1*, *Fgfr2*, *Fgf4*, and *Fgfbp1* are knocked out, respectively.

E FACS profiles of TNERT, TNERTFgfr1$^{-/-}$, TNERTFgfr2$^{-/-}$, TNERTFgf4$^{-/-}$, and TNERTFgfbp1$^{-/-}$ cells, cultured in SL treated with OHT (red) or no OHT (blue) ($n = 3$). The dotted line represents the E14Tg2a FACS profile used as negative control (−ve c).

F RT-qPCR of *Nanog*:GFP transcript from *Nanog* locus with primers complementary to 5′UTR of *Nanog* transcript.

G (left) Western blot of pERK in TNERT, TNERTFgfr1$^{-/-}$, TNERTFgfr2$^{-/-}$, TNERTFgf4$^{-/-}$, and TNERTFgfbp1$^{-/-}$ with or no OHT. (Right) relative pERK levels estimated by densitometry ($n = 4$).

H (top) FACS profiles of TNERT, TNERTFgfr1$^{-/-}$, TNERTFgfr2$^{-/-}$, TNERTFgf4$^{-/-}$, TNERTFgfbp1$^{-/-}$ cultured in SL and TNERT + SLPD ($n = 3$). The dotted line represents the FACS profile of unstained E14Tg2a cells used as negative control (−ve c). (Bottom) *Nanog*:GFP population median of indicated cell lines.

Data information: $n \geq 3$ biological replicates (each dot represents a biological replicate). Data are presented as mean ± SEM in A, F, G, and H. $^{*}P < 0.05$, $^{**}P < 0.01$, $^{***}P < 0.001$, and ns = not significant (paired two-tailed Student's *t*-test).
Source data are available online for this figure.

et al, 2013b), NBR1 at 1.4 kb, and NBR2 at −0.2 kb relative to TSS of *Fgfr2*. NBR1 and NBR2 were deleted in TNERT (Figs 4D and E, and EV4E and F). Autoregulation was operational in TNERTNBR1$^{-/-}$ albeit at reduced strength, whereas it was abolished in TNERTNBR2$^{-/-}$ (Fig 4F), suggesting that NBR2 is essential for the binding of NANOG and activation of *Fgfr2* to trigger autoregulation. Together, our data suggest dose-responsive induction of *Fgf4*, *Fgfbp1*, and *Fgfr2* by NANOG. The *Nanog*-high cells secrete more FGF4 and FGFBP1 and also express higher FGFR2 receptors. The FGF4 in the presence of FGFBP1 binds to FGFR2 to enhance FGF signaling in *Nanog*-high cells to enhance pERK and repress *Nanog*. The *Nanog*-low cells express relatively low FGFR2, resulting in weak FGF signaling and the absence of autoregulation (Fig 4G). We propose that FGFR2 distinguish the *Nanog*-high cells from the low cells to activate ERK-driven autoregulation selectively in *Nanog*-high cells.

ERK interacts and recruits NONO to repress *Nanog* transcription

FGF signaling represses *Nanog* transcription (Hamazaki *et al*, 2006; Santostefano *et al*, 2012) and regulates *Nanog* heterogeneity and monoallelic expression (Nichols *et al*, 2009; Wray *et al*, 2010; Miyanari & Torres-Padilla, 2012). How FGF signaling downstream kinases repress *Nanog* is unclear. ERK can induce Tcf15 to repress *Nanog* (Davies *et al*, 2013*)* or it can interact with NONO to regulate bivalent genes (Ma *et al*, 2016a). We deleted *Tcf15* and *Nono* in TNERT to generate TNERTTcf15$^{-/-}$ and TNERTNono$^{-/-}$ cell lines to examine their function in autoregulation (Figs 5A and EV5A, and B). OHT treatment failed to repress *Nanog*:GFP in TNERTNono$^{-/-}$, unlike in TNERTTcf15$^{-/-}$ indicating an essential role of NONO but not TCF15 (Figs 5B and EV5A and C). NONO has been shown to activate ERK (Ma *et al*, 2016a), and pERK was substantially reduced in TNERTNono$^{-/-}$ despite OHT induction, unlike in TNERT (Figs 5C and EV5D). Endogenous immunoprecipitation showed an interaction between NONO and ERK, the interaction was maintained in the presence or absence of NANOG (Fig 5D). NONO colocalizes with ERK to bivalent developmental genes to maintain poised POL2 (Ma *et al*, 2016a). The ChIP-seq data analysis from Data ref: Ma *et al* (2016b) and Data ref: Tee *et al* (2014b) showed NONO and ERK occupancy on the *Nanog* (Fig EV5E). We induced or repressed the pERK by treatment of E14Tg2a cells with FGF4 or PD (Fig 5E) and analyzed the occupancy of NONO, pERK, POL2, H3K4me3, and H3K27me3. As expected NONO levels were reduced in SLPD and increased in FGF, suggesting its dependency on pERK (Fig 5E) The transcription start site (TSS) and 5 kb upstream region (−5 kb) are the two hubs of transcription factor binding and control of *Nanog* transcription (Loh *et al*, 2006; Chen *et al*, 2008). We performed ChiP-qPCR analysis with multiple primer sets spanning the −5.8 kb to +1.5 kb region relative to TSS (Fig 5F). pERK and NONO binding were detected in immediate downstream regions of the -5 kb, and TSS. Their binding was reduced significantly in PD and enhanced in FGF4 suggesting pERK and NONO binding on *Nanog* is dependent on FGF signaling (Figs 5G and H, and EV5G). pERK was shown to recruit NONO to bivalent genes (Ma *et al*, 2016a). Although *Nanog* is not a bivalent gene, our data suggests pERK recruits NONO to *Nanog*. POL2 occupancy seen in TSS and downstream region was reduced in FGF4 and enhanced in PD treatment suggesting active transcription of *Nanog* in PD and repression in FGF4 (Figs 5I and EV5G). The difference in the enrichment between FGF4 and PD across the locus suggested pERK affected POL2 loading. We further analyzed the enrichment of POL2 on the *Nanog* locus in *Nono*$^{-/-}$ cells. POL2 enrichment was increased across the locus suggesting pERK-NONO affects POL2 loading on the *Nanog* locus (Fig EV5F). This was corroborated with enhanced enrichment of the transcription activating histone mark H3K4me3 in PD (Figs 5J and EV5G) and enrichment of transcription repressive mark H3K27me3 at the -5 kb region of the *Nanog* in FGF4 treatment (Figs 5K and EV5G). pERK phosphorylates NANOG, USP21, and affects NANOG stability and transactivation capability (Brumbaugh *et al*, 2014; Kim *et al*, 2014; Jin *et al*, 2016). In agreement with NANOG destabilization by pERK (Brumbaugh *et al*, 2014; Kim *et al*, 2014; Pokrass *et al*, 2020), the half-life of NANOG was significantly compromised in FGF4 treated cells but enhanced in PD (Figs 5L and EV5H), suggesting that the FGF/ERK represses *Nanog* transcription and also affects NANOG stability. Collectively, these data suggest that FGF signaling activates pERK and its binding onto *Nanog* in a concentration-dependent manner. pERK is essential for the recruitment of NONO to the *Nanog* locus. pERK-NONO is known to poise the POL2 in bivalent genes. In contrast, pERK-NONO affects

 © 2022 The Authors

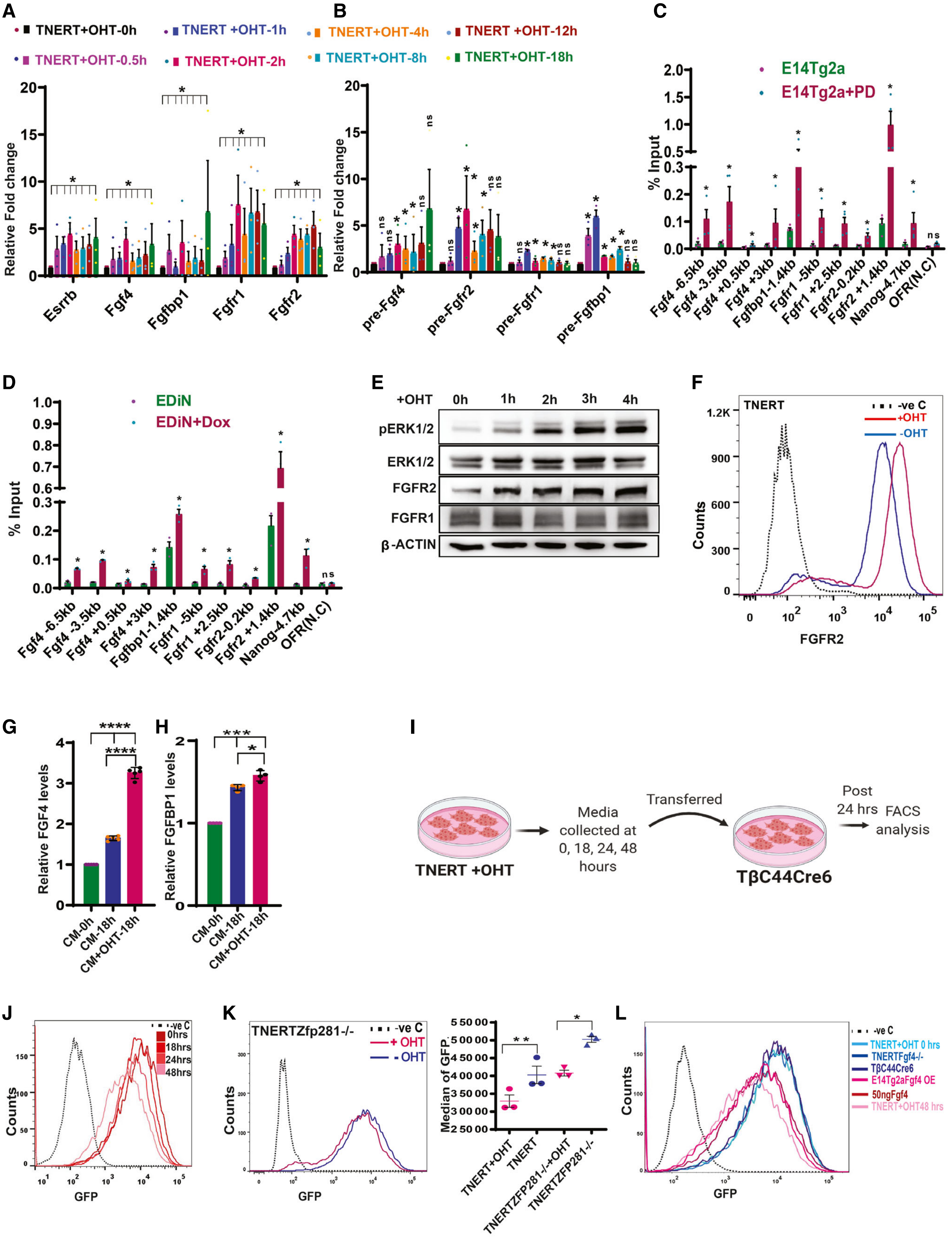
A
TNERT+OHT-0h
TNERT +OHT-1h
TNERT+OHT-0.5h
TNERT+OHT-2h
Relative Fold change
Esrrb
Fgf4
Fgfbp1
Fgfr1
Fgfr2
B
TNERT+OHT-4h
TNERT +OHT-12h
TNERT+OHT-8h
TNERT+OHT-18h
pre-Fgf4
pre-Fgfr2
pre-Fgfr1
pre-Fgfbp1
C
E14Tg2a
E14Tg2a+PD
% Input
D
EDiN
EDiN+Dox
E
+OHT
0h
1h
2h
3h
4h
pERK1/2
ERK1/2
FGFR2
FGFR1
β-ACTIN
F
TNERT
-ve C
+OHT
-OHT
Counts
FGFR2
G
Relative FGF4 levels
CM-0h
CM-18h
CM+OHT-18h
H
Relative FGFBP1 levels
I
Media collected at 0, 18, 24, 48 hours
Transferred
Post 24 hrs
FACS analysis
TNERT +OHT
TβC44Cre6
J
0hrs
18hrs
24hrs
48hrs
Counts
GFP
K
TNERTZfp281-/-
Median of GFP
TNERT+OHT
TNERT
TNERTZFP281-/-+OHT
TNERTZFP281-/-
L
TNERT+OHT 0 hrs
TNERTFgf4-/-
TβC44Cre6
E14Tg2aFgf4 OE
50ngFgf4
TNERT+OHT48 hrs

Figure 3.

◀ **Figure 3. NANOG triggers autoregulation by inducing the expression of FGFR2, FGF4, and FGFBP1.**

A RT-qPCR showing relative transcript levels after 0, 0.5, 1, 2, 4, 8, 12, and 18 h OHT treatment in TNERT (n = 3). *Esrrb*, a known direct target of NANOG was used as the positive control.
B RT-qPCR of relative levels of pre- mRNA at the above indicated time points after OHT treatment in TNERT (n = 3).
C ChIP analysis of NANOG on *Fgf4*, *Fgfbp1*, *Fgfr1*, *Fgfr2*, *Nanog* (validated target) and OFR (non-genic region (negative control)) loci in E14Tg2a cells cultured in SL or SLPD for 48 h (n = 4).
D ChIP analysis of NANOG on promoters of above-indicated loci in EDiN cells cultured in Doxycycline (red) or no Doxycycline (blue) for 48 h (n = 3).
E Western blot of FGFR1, FGFR2, and pERK in TNERT at one-hour intervals at indicated time points after OHT treatment (n > 3).
F FACS analysis of FGFR2 on the cell surface of TNERT treated with (red) or no OHT (blue) (n = 3). The dotted line represents the FACS profile of E14Tg2a cells stained with control IgG and secondary antibody used as negative control (−ve c).
G, H ELISA-based relative quantification of FGF4 (G) and FGFBP1 (H) in conditioned media from TNERT treated with or no OHT (n = 3).
I Schematic of conditioned media experiment.
J FACS analysis of Tβc44Cre6 cell line in conditioned media collected from TNERT treated with OHT after 0, 18, 24, and 48 h. The dotted line represents the FACS profile of unstained E14Tg2a cells used as negative control (−ve c).
K (left) FACS analysis of TNERTZfp281$^{-/-}$ cells treated with (red) or with no OHT (blue) treatment. The dotted line represents the FACS profile of unstained E14Tg2a cells used as negative control (−ve c). (right) *Nanog*:GFP population median of TNERTZfp281$^{-/-}$ (n = 3).
L FACS analysis of Tβc44Cre6 cell line in different conditioned media. Media from TNERT + OHT 0 h, TNERTFGF4$^{-/-}$, Tβc44Cre6 48 h, (shades of blue color) fail to repress *Nanog*:GFP. Media from E14Tg2a- FGF4-OE (overexpression) 48 h, TNERT + OHT 48 h and 50 ng/ml FGF4 (shades of maroon color) repress *Nanog*:GFP. The dotted line represents the FACS profile of unstained E14Tg2a cells used as negative control (−ve c).

Data information: $n \geq 3$ biological replicates (each dot represents a biological replicate). Data are presented as mean ± SEM in A–D, G, H, and K. *$P < 0.05$, **$P < 0.01$, ***$P < 0.001$, ****$P < 0.0001$, and ns = not significant (paired two-tailed Student's t-test).
Source data are available online for this figure.

POL2 loading onto the *Nanog* locus preventing the initiation of transcription. In the absence of active FGF signaling, pERK-NONO occupancy on the *Nanog* is decreased permitting increased POL2 loading and transcription activation of the *Nanog* (Fig 5M).

NANOG regulates ERK signaling dynamics and heterogeneity

ERK signaling regulates *Nanog* expression and heterogeneity in ES cells. Recently pERK expression is reported to be heterogeneous and dynamic in ES cells and preimplantation embryos (Kang *et al*, 2017; Molotkov *et al*, 2017; Deathridge *et al*, 2019). We have shown that FGFR2 exhibits a negatively skewed bimodal expression similar to *Nanog* in ESCs (Fig 3F) and *Fgfr2* is induced in a dosage-dependent manner by NANOG. We asked if NANOG dynamics could regulate ERK signaling dynamics in ES cells through *Fgfr2*. Immunostaining showed heterogeneous expression of NANOG and pERK in WT ESCs (E14Tg2a) with some cells co-expressing both (Fig 6A). Their expression showed a fair correlation (r = 0.36) suggesting a positive association between NANOG and pERK; similar to NANOG and FGFR2. NANOG showed a broad range of expression as represented by a range of relative fluorescence intensity (RFI 0–1,000), and pERK showed a relatively narrow range of expression (RFI 100–600) in ESCs (Fig 6B). The pERK expression in *Nanog* null ESC (TβC44Cre6) was not detectable, suggesting pERK levels are dependent on NANOG. pERK expression was completely lost in PD treatment. NANOG overexpression in WT ESCs (EDiN) enhanced the range of pERK levels relative to WT and broadened the range of pERK levels (RFI 200–800) (Fig 6B) with a fair correlation between NANOG and pERK (r = 0.37) (Fig 6B). Intriguingly, high levels of pERK failed to repress *Nanog* transgene and significantly reduce NANOG in EDiN. This resulted in the coexistence of high pERK and high NANOG in the cells. Despite very high levels of pERK, *Nanog* over-expressing EDiN does not differentiate suggesting that the *Nanog* function in ESC self-renewal is dominant over the pERK function in the differentiation of ESCs. These data suggest that pERK expression levels and dynamic range of expression in ESCs are dependent on the expression level of *Nanog* and its dynamics. To further validate this, we isolated *Nanog*-high subpopulation cells by sorting the highest 10% iRFP expressing NiRPF2A reporter ESCs by FACS. The expression of pERK and NANOG was analyzed in these cells every 4 h during their culture to study the dynamics of NANOG and pERK expression. The sorted cells were collected and cultured in the same media in which the cells were cultured prior to cell sorting. The media was filtered through a 0.22 μm filter to remove any cells. This ensured the secreted factors in the media were retained and the dynamics of expression of NANOG and pERK were analyzed over a time period in their presence. The sorted cells expressed NANOG and pERK. After 4 h of culture in the same media, the NANOG expression increased with a concomitant increase in pERK. The correlation between NANOG and pERK drastically increased within 4 h after culture. At 8 h, both NANOG and pERK expression levels were high with marginal increment in the correlation. NANOG induces pERK, there would be a time lag between the peak of NANOG and pERK. Hence, 8 h may represent the peak expression levels of one of the proteins. At 12 h, pERK levels significantly decrease with a concomitant lesser decrease in NANOG and their correlation and show fair correlation. At 16 h, both pERK and NANOG further decrease with a strong correlation (Fig 6C, Appendix Fig S1). The dynamic correlation between pERK and NANOG at the same time points shown by western blot further substantiated these observations. It suggests that NANOG and pERK follow a dynamic cycle of expression during culture (Fig 6D). These results suggest that ESCs continuously transit between different states of NANOG and pERK expression resulting in heterogeneous and dynamic expression of pERK.

Discussion

We demonstrate that the highest possible expression of *Nanog* could be achieved in SLPD by attaining a consistent low MEK1/2 activity. Wnt signaling can activate MEK1/2 at multiple levels (Yun *et al*, 2005) a relatively lower level of *Nanog* expression in SL2i and 2iL could be attributed to a time-dependent increase in MEK1/2

 © 2022 The Authors

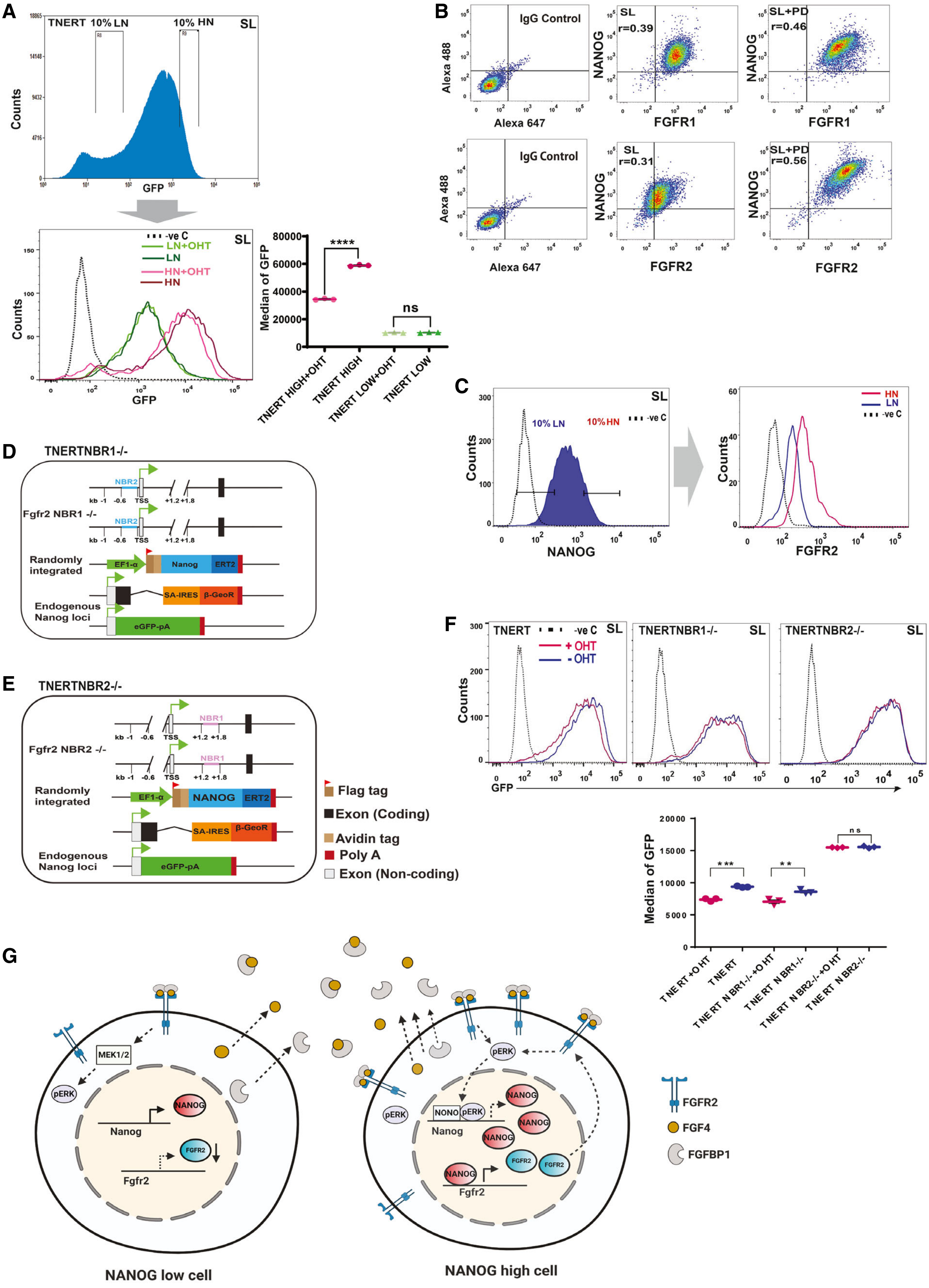
A
TNERT 10% LN
10% HN
SL
Counts
GFP
-ve C
LN+OHT
LN
HN+OHT
HN
Median of GFP
****
ns
TNERT HIGH+OHT
TNERT HIGH
TNERT LOW+OHT
TNERT LOW
B
IgG Control
Alexa 488
Alexa 647
SL
r=0.39
NANOG
FGFR1
SL+PD
r=0.46
r=0.31
FGFR2
r=0.56
C
10% LN
10% HN
NANOG
FGFR2
D
TNERTNBR1-/-
Fgfr2 NBR1 -/-
NBR2
TSS
Randomly integrated
EF1-α
Nanog
ERT2
SA-IRES
β-GeoR
Endogenous Nanog loci
eGFP-pA
E
TNERTNBR2-/-
Fgfr2 NBR2 -/-
NBR1
NANOG
Flag tag
Exon (Coding)
Avidin tag
Poly A
Exon (Non-coding)
F
TNERT
+ OHT
- OHT
TNERTNBR1-/-
TNERTNBR2-/-
***
**
n s
TNERT +OHT
TNERT
TNERT NBR1-/- +OHT
TNERT NBR1-/-
TNERT NBR2-/- +OHT
TNERT NBR2-/-
G
MEK1/2
pERK
Nanog
Fgfr2
NONO
FGFR2
FGF4
FGFBP1
NANOG low cell
NANOG high cell

Figure 4.

Figure 4. NANOG-induced FGFR2 triggers autoregulation predominately in the ES cell population with higher *Nanog* expression.

A (top) To analyze autoregulation in *Nanog*-high and *Nanog*-low cells, we sorted the lowest and the highest 10% population of the TNERT expressing GFP and treated them with OHT. FACS profile of TNERT, the position of the gates indicates the 10% low-*Nanog*:GFP (LN) and 10% high-*Nanog*:GFP (HN) population sorted for culture. (Bottom left) FACS profiles of LN and HN after 18 h culture in SL. LN (dark green), HN (dark maroon) in SL, and LN (light green), HN (light maroon) in SL + OHT. The dotted line represents the FACS profile of unstained E14Tg2a cells used as negative control (−ve c). (Bottom right) *Nanog*:GFP population median of TNERT (n = 3).

B FACS profile of E14Tg2a cultured in SL or SLPD for 48 h and co-immunostained with anti-NANOG and anti-FGFR1 or anti-FGFR2 antibodies. r-values represent the average of three independent experiments (n = 3).

C (left) FACS profile of E14Tg2a immunostained with anti-NANOG and anti-FGFR2 antibody, the gates mark the 10% low-NANOG (LN) and 10% high-NANOG (HN) population. The dotted line represents the FACS profile of E14Tg2a cells stained with control IgG and secondary antibody used as negative control (−ve c). (right) Histogram depicting the FGFR2 expression profiles in the gated LN and HN cell population (n = 3).

D, E Schematic representation of TNERTNBR1$^{-/-}$ (D) and TNERTNBR2$^{-/-}$ (E) cells, in which NANOG binding sequences at +1.4 kb (NBR1) and −0.2 kb (NBR2) are deleted, respectively.

F (top) FACS profiles of TNERT, TNERTNBR1$^{-/-}$, and TNERTNBR2$^{-/-}$ with (red) or no OHT treatment (blue). The dotted line represents the FACS profile of unstained E14Tg2a cells used as negative control (−ve c). (Bottom) *Nanog*:GFP population median of TNERT, TNERTNBR1$^{-/-}$ and TNERTNBR2$^{-/-}$ with or no OHT treatment.

G A cartoon depicting *Nanog* autoregulation in *Nanog*-high cells. The *Nanog*-high cells secrete more FGF4 and FGFBP1. They contain higher levels of FGFR2 on the surface and are hence more sensitive to the FGF ligand triggering a stronger FGF signaling. The increased pERK in these cells recruits NONO to the *Nanog* locus and represses *Nanog* transcription. The *Nanog*-low cells secrete very little FGF4 and FGFBP1 and present fewer FGFR2 on their surface and are less sensitive to FGF signaling. The pERK levels in *Nanog*-low cells are insufficient to execute *Nanog* autoregulation.

Data information: $n \geq 3$ biological replicates (each dot represents a biological replicate). Data are presented as mean ± SEM in (A), and (F). **$P < 0.01$, ***$P < 0.001$, ****$P < 0.0001$ and ns = not significant (paired two-tailed Student's t-test).

activity in the presence of PD and CHIR (Fig 1F and G). The inhibition of MEK1/2 prevents differentiation in 2iL, but a time-dependent increase in MEK1/2 activity is significant and sufficient to facilitate *Nanog* autoregulation. A time-dependent variation in MEK1/2 activity in 2iL opens up the plausibility of other molecular processes regulated by MEK1/2 activity to be functional in a naïve state.

Overexpression of *Nanog* is limited by an autorepression mechanism operating at the transcriptional level to retain the differentiation potential of ESCs (Fidalgo *et al*, 2012; Navarro *et al*, 2012). Among the multiple possible pathways that can regulate *Nanog* (Pereira *et al*, 2006; Santostefano *et al*, 2012; Davies *et al*, 2013; Jin *et al*, 2016), we show that FGF autocrine signaling is recruited for *Nanog* autoregulation. We show that a NANOG dosage-dependent differential induction of *Fgfr2* in *Nanog*-high ESCs triggers autoregulation by activation of ERK. pERK recruits NONO to the *Nanog* locus and affects the loading of POL2 onto the *Nanog* locus reducing *Nanog* transcription (Fig 5M). Other reports (Brumbaugh *et al*, 2014; Kim *et al*, 2014) and our data show that pERK can affect NANOG stability and may contribute to autorepression. However, the inability of pERK to significantly repress NANOG expressed from a transgene and a strong correlation between NANOG-pERK (Fig 6A and B) dismisses the possibility of significant contribution from post-transcriptional mechanisms in autoregulation. Our data suggest that *Nanog* autoregulation is triggered above a threshold of NANOG, thereafter the intensity of repression is dependent on the level of NANOG in the cell.

We show that NANOG activates ERK signaling by inducing *Fgfr2*, *Fgf4*, and *Fgfbp1*. The activated ERK together with NONO represses transcription of *Nanog*, resulting in a NANOG-pERK reciprocal regulatory loop (Fig 6E). The subpopulation of ES cells expressing high NANOG will have higher FGFR2. This induces high ERK activity resulting in a high-NANOG:high-pERK state. The repression of *Nanog* transcription by pERK in these cells reduces NANOG, reducing transcription of *Fgfr2*. The cells traverse through various intermediate levels of NANOG and pERK resulting in a low-NANOG: low-pERK state. Low pERK permits activation of NANOG by other

Figure 5. ERK interacts and recruits NONO to repress *Nanog* transcription.

A Schematic of TNERTNono$^{-/-}$ cell line; a derivative of TNERT in which *Nono* is knocked-out.

B FACS profile of TNERTNono$^{-/-}$ treated with or no OHT (n = 3). The dotted line represents the FACS profile of unstained E14Tg2a cells used as negative control (−ve c).

C Western blot of pERK and ERK in TNERT and TNERTNono$^{-/-}$ cells treated with or no OHT (n = 3).

D Immunoprecipitation analysis showing interactions between ERK and NONO in the presence or absence of *Nanog* induction by Doxycycline in TDiN cells.

E (left) Western blot of pERK and ERK in E14Tg2a cells treated with PD or FGF4. (Middle and right) Relative levels of pERK and NONO in E14Tg2a cells treated with PD or FGF4 ($n > 4$).

F Schematic representation of *Nanog* locus comprising the −6.0 to +2 kb region. The vertical bars represent the relative positions of primer pairs used for ChiP-qPCR analysis. S1–S6 are located upstream of the TSS, the S7 primer pair is located around TSS, and S8 and S9 are located downstream in the first intron.

G–K ChIP-qPCR analysis of pERK (G), NONO (H), Pol2 (I), H3K4me3 (J) and H3K27me3 (K) on *Nanog* 5′ region in E14Tg2a cells (blue), treated with FGF4 (green) and with PD (pink) (n = 3).

L Cycloheximide chase assay of NANOG in SL, SLPD, and SLFGF4 in E14Tg2a cells.

M A cartoon illustrating the repression of *Nanog* by FGF signaling and derepression of *Nanog* in the absence of FGF signaling. The FGF4 activates the FGF signaling cascade, resulting in the phosphorylation of ERK. pERK interacts and recruits NONO to the *Nanog* promoter and represses transcription of *Nanog*. pERK also affects the stability of the NANOG. In the absence of FGF4, the pERK levels decrease resulting in enhanced stability of NANOG and transcription of *Nanog* locus by NANOG and other pluripotency factors resulting in derepression of *Nanog* locus.

Data information: $n \geq 3$ biological replicates (each dot represents a biological replicate). Data are presented as mean ± SEM in E and G-K. *$P < 0.05$, **$P < 0.01$, ****$P < 0.0001$ and ns = not significant (paired two-tailed Student's t-test).
Source data are available online for this figure.

© 2022 The Authors

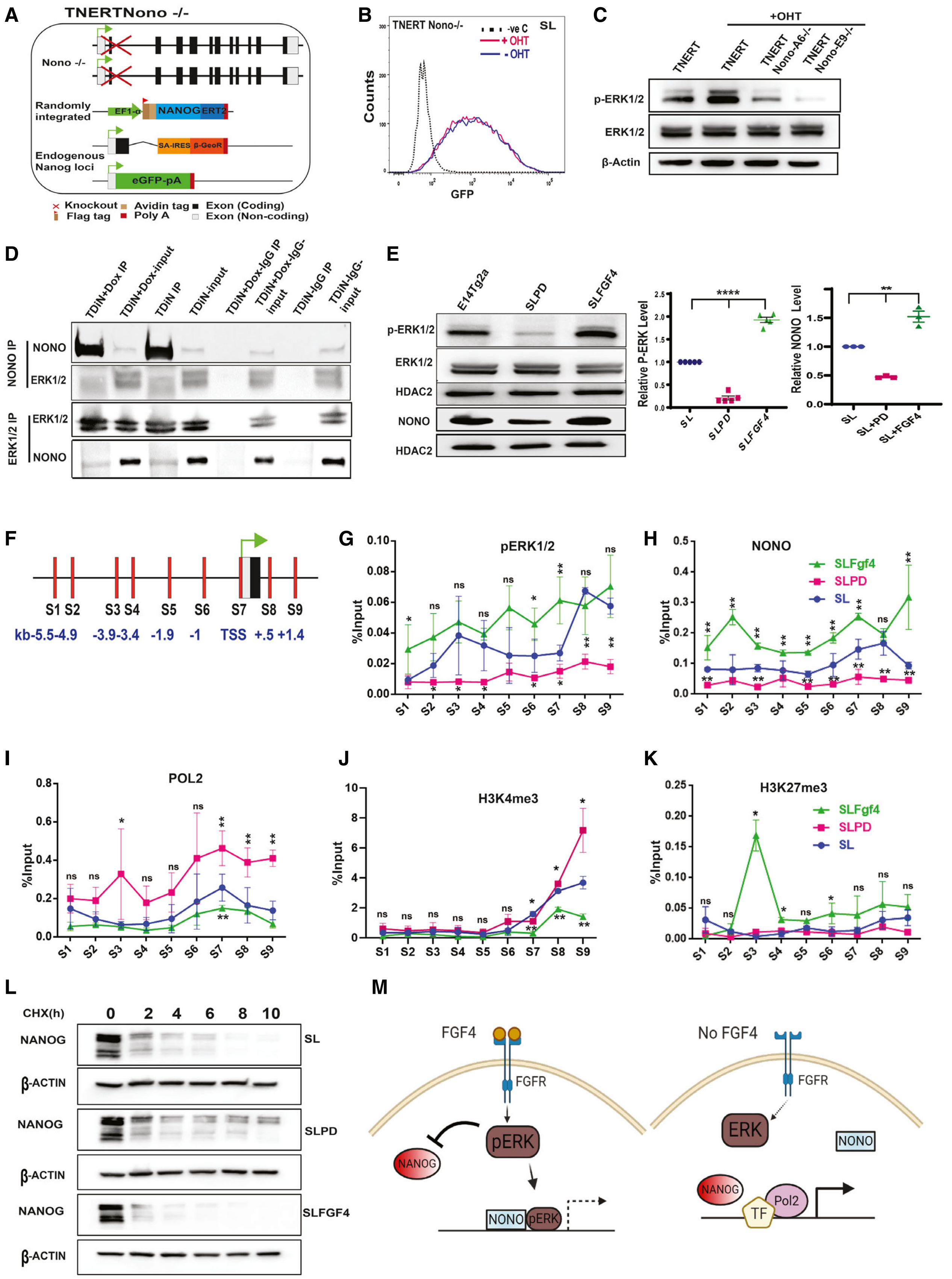
A
TNERTNono -/-
Nono -/-
Randomly integrated
EF1-α
NANOG
ERT2
Endogenous Nanog loci
SA-IRES β-GeoR
eGFP-pA
Knockout
Flag tag
Avidin tag
Poly A
Exon (Coding)
Exon (Non-coding)
B
TNERT Nono-/-
SL
-ve C
+ OHT
- OHT
Counts
GFP
C
+OHT
TNERT
TNERT
TNERT Nono-A6-/-
TNERT Nono-E9-/-
p-ERK1/2
ERK1/2
β-Actin
D
TDiN+Dox IP
TDiN+Dox-input
TDiN IP
TDiN-input
TDiN+Dox-IgG IP
TDiN+Dox-IgG-input
TDiN-IgG IP
TDiN-IgG-input
NONO IP
NONO
ERK1/2
ERK1/2 IP
ERK1/2
NONO
E
E14Tg2a
SLPD
SLFGF4
p-ERK1/2
ERK1/2
HDAC2
NONO
HDAC2
Relative P-ERK Level
****
SL
SLPD
SLFGF4
Relative NONO Level
**
SL
SL+PD
SL+FGF4
F
S1 S2
S3 S4
S5
S6
S7
S8
S9
kb-5.5-4.9
-3.9-3.4
-1.9
-1
TSS
+.5 +1.4
G
pERK1/2
%Input
H
NONO
SLFgf4
SLPD
SL
%Input
I
POL2
%Input
J
H3K4me3
%Input
K
H3K27me3
SLFgf4
SLPD
SL
%Input
L
CHX(h)
0
2
4
6
8
10
NANOG
β-ACTIN
SL
NANOG
β-ACTIN
SLPD
NANOG
β-ACTIN
SLFGF4
M
FGF4
FGFR
pERK
NANOG
NONO
pERK
No FGF4
FGFR
ERK
NONO
NANOG
Pol2
TF

Figure 5.

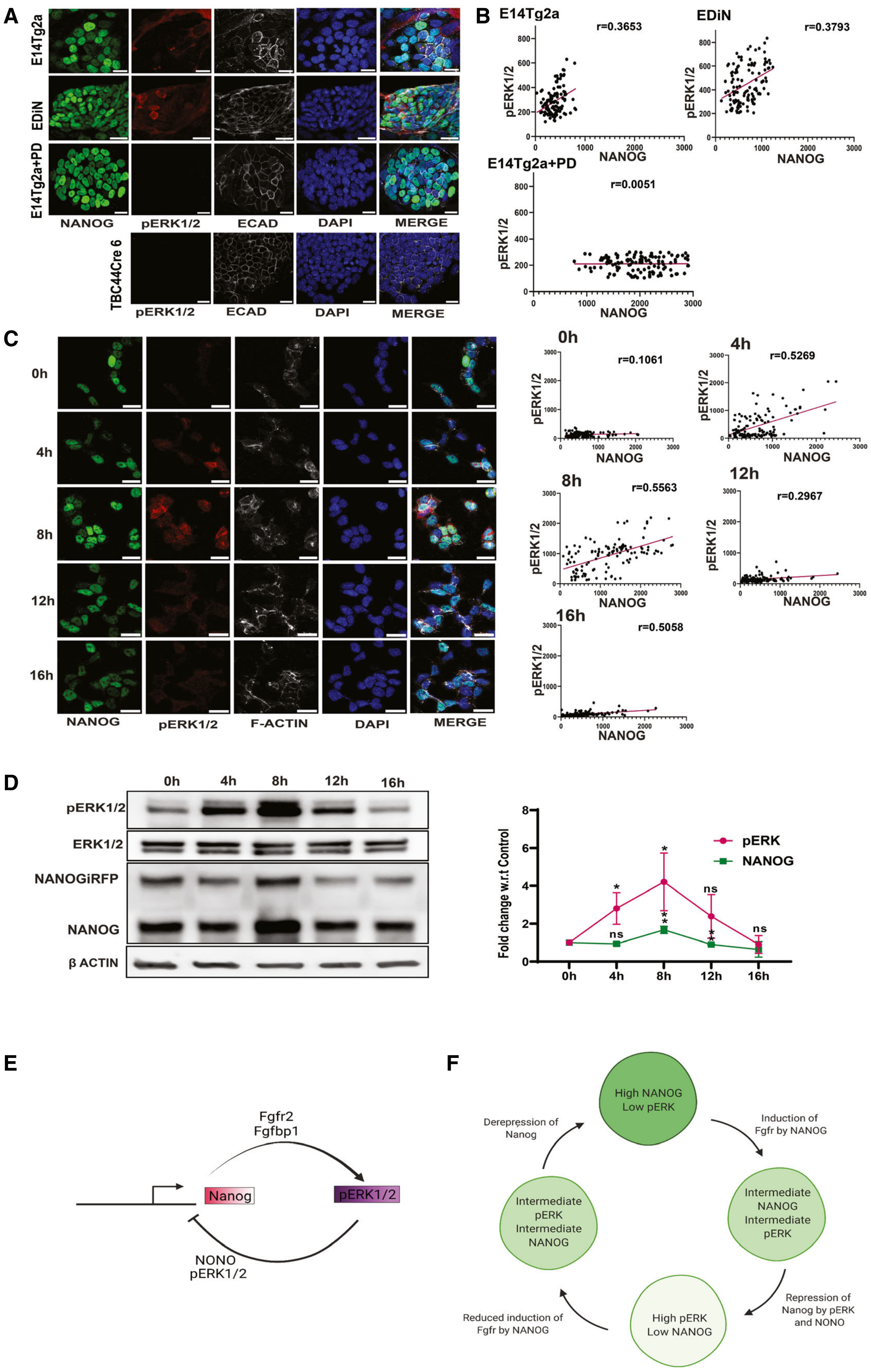

Figure 6.

© 2022 The Authors

Figure 6. NANOG regulates ERK signaling dynamics and heterogeneity.

A Immunofluorescence of pERK (red) and NANOG (green) in the indicated ESCs. Scale bars 25 μM.
B (B)The normalized fluorescence intensity of pERK was plotted against the normalized fluorescent intensity of NANOG.
C (left) Immunofluorescence of pERK and NANOG in 10% NANOG-high NiRFP2A cells at indicated time cultured in the same media collected from cells before FACS. (Right) The normalized fluorescence intensity of pERK was plotted against the normalized fluorescent intensity of NANOG at different time points. Scale bars 25 μM.
D (left) Western blot analysis of pERK, and NANOG in 10% NANOG-high NiRFP2A cells at indicated time cultured in the same media collected from cells before FAC sorting. (Right) Expression levels of NANOG and pERK in 10% NANOG-high NiRFP2A cells relative to 0 h during the time course of culture. The NANOG and pERK expression oscillate between high and low levels in the cells during culture.
E A working model of the NANOG-pERK reciprocal regulatory loop operating in ESCs. NANOG induces *Fgfbp1* and *Fgfr2 to* enhance ERK signaling in *Nanog*-high cells. pERK along with NONO occupy the *Nanog* promoter to repress its transcription. The transcription repression results in reduced NANOG, which prevents induction of *Fgfbp1*and *Fgfr2*. The is reduces ERK activity relieving the repression on the *Nanog* promoter.
F A schematic depicting the progression of cells through different expression states of NANOG and pERK expression in the ESC population. The cells expressing high-NANOG induce *Fgfbp1* and *Fgfr2* to activate pERK by autocrine signaling to give rise to a high-NANOG:high-pERK state. The repression of *Nanog* transcription by pERK leads the cells through different intermediate levels of expression of NANOG and pERK resulting in a low-NANOG:low-pERK state. The low pERK permits transcription of *Nanog* and gradual induction of *Fgfbp1* and *Fgfr2* by NANOG culminating in a high-NANOG:high-pERK state. The cells will cycle through different levels of pERK and NANOG levels generating a heterogeneous population with a strong correlation between pERK and NANOG in an ESC cells culture.

Data information: $n \geq 3$ biological replicates (each dot represents a biological replicate). Data are presented as mean ± SEM in D. *$P < 0.05$, **$P < 0.01$ and ns = not significant (paired two-tailed Student's *t*-test).
Source data are available online for this figure.

pluripotency factors gradually increasing NANOG in these cells. The increased NANOG activates *Fgfr2*, *Fgfbp1*, and *Fgf4* to induce ERK activity leading to various intermediate levels of NANOG and pERK culminating in a high-NANOG:high-pERK state. This induces a self-perpetuating cycle of activation of ERK signaling by NANOG and repression of *Nanog* by pERK leading to dynamic expression levels of NANOG and pERK in the ESC population (Fig 6F). Our cyclic NANOG-pERK circuit is in agreement with experiments of Kalmar *et al* (2009) in which, either the *Nanog* high or low cells sorted from *Nanog*-GFP reporter cells reestablish a bimodal distribution of GFP traversing through intermediate stages. This also suggests that the heterogeneous expression of *Nanog* in ESC could result from asynchronous cycling of cells through the NANOG-pERK reciprocal regulatory circuit.

pERK heterogeneity is suggested to be a vital determinant of fate choice in ICM and ES cells (Bessonnard *et al*, 2014; Kang *et al*, 2017; Molotkov *et al*, 2017; Pokrass *et al*, 2020; Simon *et al*, 2020). The mechanism generating pERK heterogeneity is unclear. pERK heterogeneity may originate due to differential local concentrations of FGF4 or FGFBP1 or heterogeneous expression of receptors FGFRs or by negative feedback regulators (ETV5, DUSP1/6). *Nanog* is considered to induce FGF paracrine signaling through FGF4 secretion and specify primitive endoderm by non-cell autonomous mechanisms (Messerschmidt & Kemler, 2010; Frankenberg *et al*, 2011). Although FGF4 is essential for *Nanog* autoregulation, it is a secreted protein. Its induction by NANOG can neither explain the functioning of autoregulation exclusively in *Nanog*-high cells nor the heterogenous pERK activation in ESCs or ICM. Among the FGF signaling components, FGFRs can be differentially distributed in the subpopulation of ESCs as they are located on the cells. They may act as discriminatory molecules to differentially sensitize the sub-populations to FGF4. FGFR1 and FGFR2 are predominate FGFRs expressed in preimplantation embryos and pluripotent cells. FGFR1 plays a primary role in the linage segregation of the ICM cells (Molotkov *et al*, 2017). Our data suggest that FGFR1 is not the predominant FGFR involved in *Nanog* autorepression in ESCs. This might be attributed to the relatively uniform expression of FGFR1 in all cell types of the blastocyst. FGFR2 is mostly restricted to extraembryonic cells in blastocyst (Kang *et al*, 2017; Molotkov *et al*, 2017). Although FGFR2 was not detected in the epiblast (Kang *et al*, 2017; Molotkov *et al*, 2017), our data shows the expression of FGFR2 in ESC. Further, *Fgfr2* is induced by NANOG and correlates with NANOG expression. We suggest a dosage-dependent induction of *Fgfr2* by NANOG and its accumulation on the surface of NANOG high cells can potentiate the cells to differentially respond to FGF4. Our data establish that the dosage-dependent induction of *Fgfr2* is the basis for differential activation of ERK in subpopulations of ESCs resulting in pERK heterogeneity. The carrier protein FGFBP1 may also locally enhance FGF signaling further contributing to pERK heterogeneity similar to heparan sulfate proteoglycans (Galanternik *et al*, 2015).

We propose the reciprocal regulation of *Nanog* by ERK signaling and ERK signaling by NANOG as the basis for both NANOG and pERK heterogeneity. We suggest that the NANOG-pERK axis may not merely be viewed as a mechanism of regulation of *Nanog* expression by ERK signaling, but rather as a cyclic circuit where *Nanog* heterogeneity and expression dynamics lead to ERK signaling dynamics and vice versa. *Nanog* and ERK signaling are induced in multiple cancers (Song *et al*, 2017; Huang *et al*, 2020). The significance of the NANOG-pERK reciprocal regulatory loop in establishing heterogeneity and ERK signaling dynamics may not be limited to pluripotent cells but could be relevant in cancer stem cells and tumor heterogeneity.

Materials and Methods

Cell culture

The cell lines used in this study and their origin is depicted in Appendix Fig S2. All the cells used in this study are derivatives of E14Tg2a ESC. The cells were cultured as described earlier (Festuccia *et al*, 2012). 4-Hydroxytamoxifen (4-OHT), Doxycycline, and Cycloheximide were used at a concentration of 1 μg/ml, 1 μg/ml, and 100 μg/ml, respectively. The TNERT and its derivative cell lines were treated with 4-OHT for 18 h except when indicated. TDiN and EDiN were treated with Doxycycline for 48 h unless indicated. CHIR99021 (CHIR), PD0325901 (PD), and SU5402 were used at

© 2022 The Authors

3 μM, 1 μM, and 2 μM, respectively, except when indicated. FGF4 and FGFBP1 were used at 50 ng/ml concentration. The cells were cultured in Serum + LIF (SL), SL + PD (SLPD), SL + CHIR (SLCHIR), SL + SU5402 (SLSU5402), SL + PD + CHIR (SL2i), and N2B27 + LIF + PD + CHIR (2iL) for at least 2 passages before treating with either 4-OHT or Doxycycline.

The cells were cultured on cell culture dishes coated with 0.1% gelatin for all experiments. The conditioned media from the cells was collected after the specific treatments or indicated time points. The conditioned media was passed through a 0.22 μM filter and added to Tβc44Cre6 or TNERT cells. The cells were cultured in the conditioned media for 24 h before FACS analysis.

Generation of Knock-out cell lines using paired CRISPR constructs

pU6-iRFP (pU6-Cas9-T2A-iRFP-2A-PuroR) construct was engineered by replacing mCherry coding sequence with iRFP670-2A-PuroR cassette in pU6-(BbsI)-CBh-Cas9-T2A-mCherry plasmid (Addgene 64324) by Gibson assembly. For generating knock-out of a gene, two sgRNAs were designed with the expected cutting sites at least 30 bps apart to achieve deletion of at least 30 bps or more. For genotyping of the deletions, a set of genotyping primers was designed outside the deletion region flanking the sgRNA pair. The sgRNAs were designed using the UCSC genome browser and Deskgen or Benchling. The sequences of the sgRNAs and the genotyping primers are detailed in Appendix Table S1. All sgRNAs were cloned into pU6-Cas9-T2A-iRFP-2A-PuroR plasmids. To generate a paired sgRNA construct, the U6-SgRNA cassette from one plasmid containing the sgRNA was amplified and Gibson assembled into the XbaI site of the plasmid containing the other sgRNA of the pair. Approximately 1 μg of paired sgRNA CRISPR plasmid was nucleofected in 1 million cells. The transfected cells were sorted by FACS for iRFP expression and cultured to obtain clones. The clones were genotyped by PCR using respective primer sets to identify the heterozygous and homozygous clones. The sequence of the derivation of cell lines is described in Appendix Fig S2.

Generation of Knock-in cell lines

A sgRNA encompassing the stop codon of *Nanog* was cloned into pU6-iRFP and co-transfected with the targeting vectors. The 2A-mCherry cassette was replaced with iRFP sequences by Gibson assembly in the Nanog-2A-mCherry targeting vector (Addgene 59995) to generate Nanog iRFP670 fusion targeting vector. Approximately 3 μg plasmid (targeting vector and CRISPR plasmid) were nucleofected in 3 million E14Tg2a cells. The cells were selected against G418. The derivation of cell lines is described in Appendix Fig S2. The cell lines used in this study are free of mycoplasma (Appendix Fig S3). Cell line identity was ascertained by STR analysis as shown in Appendix Fig S4 and Appendix Table S3.

Western blot analysis

The cells were harvested by using RIPA buffer with 25 mM Tris–HCl (pH 8.0), 150 mM NaCl, 1%NP-40, 0.5% Sodium deoxycholate, 0.1% SDS and Complete Protease Inhibitor Cocktail Tablets (Roche). The protein samples were resolved by 4–20% gradient SDS-PAGE and electroblotted onto a polyvinylidene difluoride (PVDF) membrane. The blot was blocked with 3% Blotto for an hour and incubated overnight with a primary antibody at 4°C. Blots were washed thrice with TBST and hybridized with secondary antibodies and the blots were visualized using enhanced chemiluminescence (ECL)detection kit. Western blot quantifications were performed using Image lab (Bio-rad).

Real-time PCR analysis

The RNA was extracted with TRIZOL reagent and quantified using a Nanodrop2000 spectrophotometer (Thermo Fisher Scientific). One microgram of total RNA was reversed transcribed into cDNA by using superscript III. All real-time PCR was carried out with Power SYBR Green PCR master mix on the ABI prism 7900 HT sequence detection system (ABI) as per the manufacturer's instructions. GAPDH was used as an internal control or normalizer. The data was analyzed by SDS 2.2 software provided with the instrument. The primers used for real-time PCR are given in Appendix Table S1.

Chromatin immunoprecipitation (ChIP)

Cells were fixed by adding 270 μl of 37% formaldehyde into 10 ml of media and incubated for 10 min at 37°C to crosslink the chromatin. Cells were washed twice with cold PBS containing protease inhibitors. Cells were scraped and harvested by centrifugation. The cell pellet was dissolved in 200 μl of SDS Lysis Buffer (1% SDS, 10 mM EDTA, and 50 mM Tris, pH 8.0) containing protease inhibitors (per 10^6 cells) and incubated on ice for 10 min. The 25 cycles of sonication were used to shear DNA between 200 to 1,000 base pairs. The sample was centrifuged at 13,000 rpm for 10 min (at 4°C). The supernatant was diluted by adding 1,800 μl ChIP Dilution Buffer (1.1% Triton X- 100, 1.2 mM EDTA, 16.7 mM Tris–HCl, pH 8.0, 167 mM NaCl with protease inhibitors). The 1% input was aliquoted from the supernatant. To reduce nonspecific background, diluted cell supernatant was preabsorbed for 1 h at 4°C with protein A/G magnetic beads (Invitrogen). The supernatant fraction was incubated overnight at 4°C with an appropriate antibody and protein A/G magnetic beads were blocked with 4% BSA, and 2 μg salmon sperm DNA. The next day, preblocked beads were mixed with the sample and incubated for 1 h to capture the antibodies. The supernatant was discarded and washed in the given order with 1 ml of each of the buffers—Low Salt Wash Buffer (0.1% SDS, 1% Triton X-100, 2 mM EDTA, 20 mM Tris–HCl, pH 8.0, 150 mM NaCl), High Salt Wash Buffer (0.1% SDS, 1% Triton X-100, 2 mM EDTA, 20 mM Tris–HCl, pH 8.0, 500 mM NaCl), LiCl Wash Buffer (0.25 M LiCl, 1% IGEPAL-CA630, 1% Deoxycholic acid, 1 mM EDTA, 10 mM Tris, pH 8.0.), and TE buffer. DNA was eluted with elution buffer (1%SDS, 0.1 M NaHCO3). The sample input and the ChIP chromatin were reverse crosslinked with 20 μl of 5 M NaCl by heating at 65°C for 4 h. Followed by one hour at 45°C with 10 μl of 0.5 M EDTA, 20 μl 1 M Tris–HCl, pH 6.5, and 2 μl of 10 mg/ml Proteinase K. Finally, DNA was eluted in 50 μl water using a minElute PCR purification kit. Then 1 μl of sample and input was used for qPCR analysis. The primers used for qPCR analysis are listed in Appendix Table S1.

 © 2022 The Authors

Co-Immunoprecipitation in ES cells

10–12 million ES cells were harvested by trypsinization, washed twice with cold PBS, and resuspended in 800 μl of CoIP Lysis Buffer (50 mM Tris–HCl, pH 67.5; 350 mM NaCl, 0.7% NP40, EDTA 0.1 mM, 20% (v/v) glycerol, and protease inhibitor cocktail). The cell lysate was mixed with protein A/G magnetic beads for 1 h at 4°C for pre-clearing the background. Then 5% input was aliquoted and the remaining supernatant was incubated overnight with the appropriate primary antibody. The protein A/G magnetic beads were blocked overnight at 4°C with 200 μl of CoIP Lysis buffer containing 4% BSA. The next day, the beads were transferred to the primary antibody incubated tubes and incubated for 1 h at 4°C. The bead was washed three times with ice-cold TBS150 (50 mM Tris, 150 mM NaCl) and the protein was eluted with 2 sample buffer (125 mM Tris–HCl, pH 6.8, 4% SDS, 20% (v/v) glycerol, 0.004% bromophenol blue), by boiling for 5 min. The western was done for sample and input and the interaction was analyzed.

Immunocytochemistry

The cells were cultured in 24-well dishes and fixed in 3.7% formaldehyde diluted in PBS for 15 min at RT. After 3 washes with PBS, the cells were permeabilized and blocked with PBS containing 0.5% BSA and 0.3% Triton-X100 for 1 h at room temperature. The cells were hybridized with primary antibody (1:100 dilution) in PBS containing 0.5% BSA at 4°C overnight in a humidified chamber. The cells were washed three times with PBS and hybridized to an appropriate secondary antibody at 1:1,000 dilution room temperature for 1 h. The nuclei were stained with DAPI in 1× PBS for 20 min at room temperature. The cells were washed thrice with PBS. The cells were layered with 100 μl of the mixture of PBS and Glycerol (1:1) and the images were acquired on the ZEISS Axio observer microscope, Olympus FV3000 confocal microscope, and analyzed using ImageJ software. E-Cadherin or phalloidin staining was utilized to mark the boundaries of the cells. The cells in the colonies were segmented manually using E-Cadherin or phalloidin staining in imageJ software. The mean fluorescence intensity (MFI) was estimated for around 200 cells and correlation plots were plotted.

ELISA assay

The condition media from the cell lines was collected at the respective time points. 100 μl of the media was coated per well of 96 wells of ELISA plate by incubating overnight at 4°C. The wells were washed thrice with PBS containing 0.05% Tween-20 and blocked with PBS containing 2% BSA for 1 h at room temperature. The wells were washed once with PBS and incubated with the appropriate primary antibody (1:100) for 1 h. Washed thrice with PBST, an appropriate HRP-labeled secondary antibody was hybridized for 1 h at room temperature. The wells were washed thrice with PBST and incubated in substrate solution OPD (o-phenylenediamine dihydrochloride) 3 mg/ml with 6 μl/ml (H_2O_2) for 30 min in dark. The reaction was stopped by using 2N H_2SO_4. The absorbance was measured at 492 nm in Power wave XS2 (Bio Tek instruments).

FACS analysis

Reporter cells

Cells were trypsinized and collected by spinning at 800 rpm for 5 min. The media was removed and cells were resuspended in 300 μl of PBS containing 2% FBS at 10^6 cells/ml. The samples were analyzed in the Gallios flow cytometer (Beckman Coulter) or Fortessa flow cytometer (BD Biosciences). Sorting was performed on a MoFlo-XDP cell sorter (Beckman Coulter).

Immunostained cells

Cells were harvested by treatment with 0.5 mM EDTA and resuspended into single cells. The cells were fixed in PBS with 4% paraformaldehyde (PFA) for 20 min at room temperature. Cells were washed twice with cold PBS and incubated with methanol for 30 min for permeabilization. In the case of experiments involving the analysis of FGFRs on the cell surface, the permeabilization step was excluded. Then cells were blocked with PBS containing 0.5% BSA for 60 min at room temperature. The cells were washed and hybridized to the appropriate primary antibody at 4°C overnight. The cells were washed thrice with PBS and hybridized to the appropriate secondary antibody in PBS containing 0.5% BSA at 1:1,000 dilution for 1 h at room temperature. The cells were washed thrice with PBS and the fluorescence profiles were acquired in the Gallios FACS analyzer (Beckman Coulter). All the FACS data were analyzed using FlowJo software (BD Biosciences).

Statistical analysis and reproducibility

Statistical analysis was done by using a two-tailed paired or unpaired student *t*-test. The representation of data is in the form of mean ± SEM. The mean was calculated for $n \geq 3$ biological replicates. *$P \leq 0.05$, **$P \leq 0.001$, ***$P \leq 0.0001$, ****$P \leq 0.00001$, and ns = not significant.

Data availability

No primary data sets have been generated and deposited.

Expanded View for this article is available online.

Acknowledgements

HTK was supported by a fellowship from ICMR (India), DJ, VVV, and MS was supported by a fellowship from UGC (India). RSR was supported by DBT grant No: BT/PR14064/GET/119/16/2015. PCS was supported by WT/DBT IA grant No: 500053/Z/09/Z. We thank the Microscopy, and FACS core facilities of CCMB for the support extended to carry out this work. We thank Ian chambers for his constructive comments on the manuscript. Illustrations created with biorender.com.

Author contributions

Hanuman T Kale: Conceptualization; resources; data curation; formal analysis; validation; investigation; visualization; methodology; writing – original draft; writing – review and editing. **Rajendra Singh Rajpurohit:** Resources; validation; investigation. **Debabrata Jana:** Resources; validation; visualization; writing – review and editing. **Vijay V Vishnu:** Resources; data curation; investigation. **Mansi Srivastava:** Resources. **Preeti R Mourya:** Resources. **Gunda**

© 2022 The Authors

Srinivas: Investigation. **P Chandra Shekar:** Conceptualization; supervision; funding acquisition; methodology; writing – original draft; project administration; writing – review and editing.

Disclosure and competing interests statement

The authors declare that they have no conflict of interest.

References

Auciello G, Cunningham DL, Tatar T, Heath JK, Rappoport JZ (2013) Regulation of fibroblast growth factor receptor signalling and trafficking by Src and Eps8. *J Cell Sci* 126: 613 – 624

Bessonnard S, De Mot L, Gonze D, Barriol M, Dennis C, Goldbeter A, Dupont G, Chazaud C (2014) Gata6, Nanog and Erk signaling control cell fate in the inner cell mass through a tristable regulatory network. *Development* 141: 3637 – 3648

Brumbaugh J, Russell JD, Yu P, Westphall MS, Coon JJ, Thomson JA (2014) NANOG is multiply phosphorylated and directly modified by ERK2 and CDK1 in vitro. *Stem Cell Rep* 2: 18 – 25

Chambers I, Colby D, Robertson M, Nichols J, Lee S, Tweedie S, Smith A (2003) Functional expression cloning of Nanog, a pluripotency sustaining factor in embryonic stem cells. *Cell* 113: 643 – 655

Chambers I, Silva J, Colby D, Nichols J, Nijmeijer B, Robertson M, Vrana J, Jones K, Grotewold L, Smith A (2007) Nanog safeguards pluripotency and mediates germline development. *Nature* 450: 1230 – 1234

Chen X, Xu H, Yuan P, Fang F, Huss M, Vega VB, Wong E, Orlov YL, Zhang W, Jiang J (2008) Integration of external signaling pathways with the core transcriptional network in embryonic stem cells. *Cell* 133: 1106 – 1117

Davies OR, Lin C-Y, Radzisheuskaya A, Zhou X, Taube J, Blin G, Waterhouse A, Smith AJ, Lowell S (2013) Tcf15 primes pluripotent cells for differentiation. *Cell Rep* 3: 472 – 484

Deathridge J, Antolović V, Parsons M, Chubb JR (2019) Live imaging of ERK signalling dynamics in differentiating mouse embryonic stem cells. *Development* 146: dev172940

Faddah DA, Wang H, Cheng AW, Katz Y, Buganim Y, Jaenisch R (2013) Single-cell analysis reveals that expression of nanog is biallelic and equally variable as that of other pluripotency factors in mouse ESCs. *Cell Stem Cell* 13: 23 – 29

Festuccia N, Osorno R, Halbritter F, Karwacki-Neisius V, Navarro P, Colby D, Wong F, Yates A, Tomlinson SR, Chambers I (2012) Esrrb is a direct Nanog target gene that can substitute for Nanog function in pluripotent cells. *Cell Stem Cell* 11: 477 – 490

Fidalgo M, Faiola F, Pereira C-F, Ding J, Saunders A, Gingold J, Schaniel C, Lemischka IR, Silva JC, Wang J (2012) Zfp281 mediates Nanog autorepression through recruitment of the NuRD complex and inhibits somatic cell reprogramming. *Proc Natl Acad Sci U S A* 109: 16202 – 16207

Fidalgo M, Shekar PC, Ang YS, Fujiwara Y, Orkin SH, Wang J (2011) Zfp281 functions as a transcriptional repressor for pluripotency of mouse embryonic stem cells. *Stem Cells* 29: 1705 – 1716

Filipczyk A, Gkatzis K, Fu J, Hoppe PS, Lickert H, Anastassiadis K, Schroeder T (2013) Biallelic expression of nanog protein in mouse embryonic stem cells. *Cell Stem Cell* 13: 12 – 13

Francavilla C, Rigbolt KT, Emdal KB, Carraro G, Vernet E, Bekker-Jensen DB, Streicher W, Wikström M, Sundström M, Bellusci S (2013) Functional proteomics defines the molecular switch underlying FGF receptor trafficking and cellular outputs. *Mol Cell* 51: 707 – 722

Frankenberg S, Gerbe F, Bessonnard S, Belville C, Pouchin P, Bardot O, Chazaud C (2011) Primitive endoderm differentiates via a three-step mechanism involving Nanog and RTK signaling. *Dev Cell* 21: 1005 – 1013

Galanternik MV, Kramer KL, Piotrowski T (2015) Heparan sulfate proteoglycans regulate Fgf signaling and cell polarity during collective cell migration. *Cell Rep* 10: 414 – 428

Hamazaki T, Kehoe SM, Nakano T, Terada N (2006) The Grb2/Mek pathway represses Nanog in murine embryonic stem cells. *Mol Cell Biol* 26: 7539 – 7549

Hayashi K, de Sousa Lopes SMC, Tang F, Surani MA (2008) Dynamic equilibrium and heterogeneity of mouse pluripotent stem cells with distinct functional and epigenetic states. *Cell Stem Cell* 3: 391 – 401

Huang C, Yoon C, Zhou X-H, Zhou Y-C, Zhou W-W, Liu H, Yang X, Lu J, Lee SY, Huang K (2020) ERK1/2-Nanog signaling pathway enhances CD44 (+) cancer stem-like cell phenotypes and epithelial-to-mesenchymal transition in head and neck squamous cell carcinomas. *Cell Death Dis* 11: 1 – 14

Jin J, Liu J, Chen C, Liu Z, Jiang C, Chu H, Pan W, Wang X, Zhang L, Li B (2016) The deubiquitinase USP21 maintains the stemness of mouse embryonic stem cells via stabilization of Nanog. *Nat Commun* 7: 1 – 15

Kalmar T, Lim C, Hayward P, Munoz-Descalzo S, Nichols J, Garcia-Ojalvo J, Arias AM (2009) Regulated fluctuations in nanog expression mediate cell fate decisions in embryonic stem cells. *PLoS Biol* 7: e1000149

Kang M, Garg V, Hadjantonakis A-K (2017) Lineage establishment and progression within the inner cell mass of the mouse blastocyst requires FGFR1 and FGFR2. *Dev Cell* 41: 496 – 510

Karwacki-Neisius V, Göke J, Osorno R, Halbritter F, Ng JH, Weiße AY, Wong FC, Gagliardi A, Mullin NP, Festuccia N (2013a) Reduced Oct4 expression directs a robust pluripotent state with distinct signaling activity and increased enhancer occupancy by Oct4 and Nanog. *Cell Stem Cell* 12: 531 – 545

Karwacki-Neisius V, Göke J, Osorno R, Halbritter F, Ng JH, Weiße AY, Wong FC, Gagliardi A, Mullin NP, Festuccia N (2013b) European Nucleotide Archive PRJEB1833 (https://www.ebi.ac.uk/ena/browser/view/PRJEB1833?show=reads). [DATASET]

Kim S-H, Kim MO, Cho Y-Y, Yao K, Kim DJ, Jeong C-H, Yu DH, Bae KB, Cho EJ, Jung SK (2014) ERK1 phosphorylates Nanog to regulate protein stability and stem cell self-renewal. *Stem Cell Res* 13: 1 – 11

Kunath T, Saba-El-Leil MK, Almousailleakh M, Wray J, Meloche S, Smith A (2007) FGF stimulation of the Erk1/2 signalling cascade triggers transition of pluripotent embryonic stem cells from self-renewal to lineage commitment. *Development* 134: 2895 – 2902

Lanner F, Rossant J (2010) The role of FGF/Erk signaling in pluripotent cells. *Development* 137: 3351 – 3360

Loh Y-H, Wu Q, Chew J-L, Vega VB, Zhang W, Chen X, Bourque G, George J, Leong B, Liu J (2006) The Oct4 and Nanog transcription network regulates pluripotency in mouse embryonic stem cells. *Nat Genet* 38: 431 – 440

Ma C, Karwacki-Neisius V, Tang H, Li W, Shi Z, Hu H, Xu W, Wang Z, Kong L, Lv R (2016a) Nono, a bivalent domain factor, regulates Erk signaling and mouse embryonic stem cell pluripotency. *Cell Rep* 17: 997 – 1007

Ma C, Karwacki-Neisius V, Tang H, Li W, Shi Z, Hu H, Xu W, Wang Z, Kong L, Lv R (2016b) Gene Expression Omnibus GSE73426 (https://www.ncbi.nlm.nih.gov/geo/query/acc.cgi?acc=GSE73426). [DATASET]

Messerschmidt DM, Kemler R (2010) Nanog is required for primitive endoderm formation through a non-cell autonomous mechanism. *Dev Biol* 344: 129 – 137

Mitsui K, Tokuzawa Y, Itoh H, Segawa K, Murakami M, Takahashi K, Maruyama M, Maeda M, Yamanaka S (2003) The homeoprotein Nanog is

 © 2022 The Authors

required for maintenance of pluripotency in mouse epiblast and ES cells. *Cell* 113: 631 – 642

Miyanari Y, Torres-Padilla M-E (2012) Control of ground-state pluripotency by allelic regulation of Nanog. *Nature* 483: 470 – 473

Molotkov A, Mazot P, Brewer JR, Cinalli RM, Soriano P (2017) Distinct requirements for FGFR1 and FGFR2 in primitive endoderm development and exit from pluripotency. *Dev Cell* 41: 511 – 526

Mora-Castilla S, Tejedo J, Hmadcha A, Cahuana GM, Martín F, Soria B, Bedoya F (2010) Nitric oxide repression of Nanog promotes mouse embryonic stem cell differentiation. *Cell Death Differ* 17: 1025 – 1033

Navarro P, Festuccia N, Colby D, Gagliardi A, Mullin NP, Zhang W, Karwacki-Neisius V, Osorno R, Kelly D, Robertson M (2012) OCT4/SOX2-independent Nanog autorepression modulates heterogeneous Nanog gene expression in mouse ES cells. *EMBO J* 31: 4547 – 4562

Nichols J, Silva J, Roode M, Smith A (2009) Suppression of Erk signalling promotes ground state pluripotency in the mouse embryo. *Development* 136: 3215 – 3222

Niwa H, Ogawa K, Shimosato D, Adachi K (2009) A parallel circuit of LIF signalling pathways maintains pluripotency of mouse ES cells. *Nature* 460: 118 – 122

Ornitz DM, Xu J, Colvin JS, McEwen DG, MacArthur CA, Coulier F, Gao G, Goldfarb M (1996) Receptor specificity of the fibroblast growth factor family. *J Biol Chem* 271: 15292 – 15297

Park S-J, Shirahige K, Ohsugi M, Nakai K (2015) DBTMEE: a database of transcriptome in mouse early embryos. *Nucleic Acids Res* 43: D771 – D776

Pereira L, Yi F, Merrill BJ (2006) Repression of Nanog gene transcription by Tcf3 limits embryonic stem cell self-renewal. *Mol Cell Biol* 26: 7479 – 7491

Pokrass MJ, Ryan KA, Xin T, Pielstick B, Timp W, Greco V, Regot S (2020) Cell-cycle-dependent ERK signaling dynamics direct fate specification in the mammalian preimplantation embryo. *Dev Cell* 55: 328 – 340

Santostefano KE, Hamazaki T, Pardo CE, Kladde MP, Terada N (2012) Fibroblast growth factor receptor 2 homodimerization rapidly reduces transcription of the pluripotency gene Nanog without dissociation of activating transcription factors. *J Biol Chem* 287: 30507 – 30517

Simon CS, Rahman S, Raina D, Schröter C, Hadjantonakis A-K (2020) Live visualization of ERK activity in the mouse blastocyst reveals lineage-specific signaling dynamics. *Dev Cell* 55: 341 – 353

Song K, Cho H, Kim S, Lee H, Oh S, Woo S, Hong S, Jang H, Noh KH, Choi C (2017) API5 confers cancer stem cell-like properties through the FGF2-NANOG axis. *Oncogene* 6: e285

Tassi E, Al-Attar A, Aigner A, Swift MR, McDonnell K, Karavanov A, Wellstein A (2001) Enhancement of fibroblast growth factor (FGF) activity by an FGF-binding protein. *J Biol Chem* 276: 40247 – 40253

Tee W-W, Shen SS, Oksuz O, Narendra V, Reinberg D (2014a) Erk1/2 activity promotes chromatin features and RNAPII phosphorylation at developmental promoters in mouse ESCs. *Cell* 156: 678 – 690

Tee W-W, Shen SS, Oksuz O, Narendra V, Reinberg D (2014b) European Nucleotide Archive PRJNA213296 (https://www.ebi.ac.uk/ena/browser/view/PRJNA213296?show=reads). [DATASET]

Torres-Padilla M-E, Chambers I (2014) Transcription factor heterogeneity in pluripotent stem cells: a stochastic advantage. *Development* 141: 2173 – 2181

Toyooka Y, Shimosato D, Murakami K, Takahashi K, Niwa H (2008) Identification and characterization of subpopulations in undifferentiated ES cell culture. *Development* 135: 909 – 918

van den Berg DL, Zhang W, Yates A, Engelen E, Takacs K, Bezstarosti K, Demmers J, Chambers I, Poot RA (2008) Estrogen-related receptor beta interacts with Oct4 to positively regulate Nanog gene expression. *Mol Cell Biol* 28: 5986 – 5995

Wray J, Kalkan T, Smith AG (2010) The ground state of pluripotency. *Biochem Soc Trans* 38: 1027 – 1032

Yun M-S, Kim S-E, Jeon SH, Lee J-S, Choi K-Y (2005) Both ERK and Wnt/β-catenin pathways are involved in Wnt3a-induced proliferation. *J Cell Sci* 118: 313 – 322

Zhang X, Ibrahimi OA, Olsen SK, Umemori H, Mohammadi M, Ornitz DM (2006) Receptor specificity of the fibroblast growth factor family: the complete mammalian FGF family. *J Biol Chem* 281: 15694 – 15700

License: This is an open access article under the terms of the Creative Commons Attribution License, which permits use, distribution and reproduction in any medium, provided the original work is properly cited.

Expanded View Figures

Figure EV1. Residual MEK1/2 activity in the ground state prevents complete derepression of *Nanog*.

A (top) Schematic depiction of NiRFP2A with both alleles of *Nanog* fused to iRFP coding sequences in frame with last coding sequence. (Middle) CRISPR mediated knock-in strategy of the iRFP-loxP-IRES-NeoR-loxP cassette into *Nanog* locus. The sgRNA includes the stop codon of the *Nanog* gene. The location of the genotyping primers (FP/RP) for the knock-in is marked by the arrows. (Bottom) Genotyping of the NiRFP2A clones, a 2.9 kb band is amplified only in the knock-in clones as one of the primers is complementary to a sequence outside the left homology arm and the other primer is complementary to the iRFP sequence.

B Relative quantification of OCT4 and SOX2. The expression is normalized relative to HDAC2 levels and expression levels of OCT4 and SOX2 in SL ($n \geq 3$).

C, D Relative pERK expression levels in indicated time points and treatments ($n = 3$).

E Western blot of pERK and ERK in 1 μM PD and increasing concentrations of CHIR in SL media.

F Western blot of pERK and ERK in 3 μM CHIR and increasing concentrations of PD in SL media. G, H Relative pERK expression levels in indicated concentrations of CHIR and PD, respectively ($n \geq 3$).

G, H Relative pERK expression levels in indicated concentrations of CHIR and PD, respectively ($n \geq 3$).

Data information: $n \geq 3$ biological replicates (each dot represents a biological replicate). Data are presented as mean ± SEM in B–D and G–H. *$P < 0.05$, **$P < 0.01$, ***$P < 0.001$, ****$P < 0.0001$ and ns = not significant (paired two-tailed Student's t-test).
Source data are available online for this figure.

 © 2022 The Authors

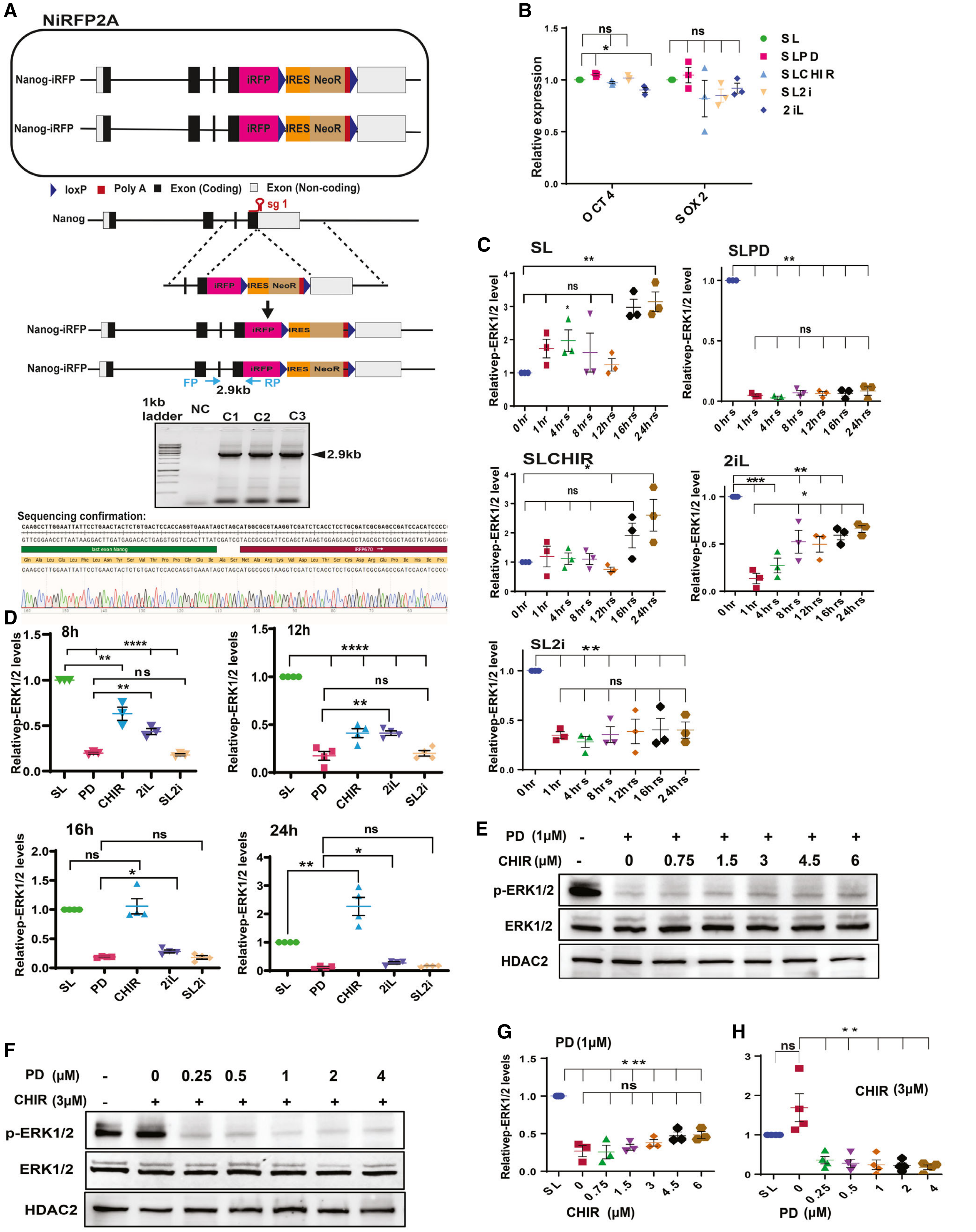
A
NiRFP2A
Nanog-iRFP
Nanog-iRFP
loxP Poly A Exon (Coding) Exon (Non-coding)
sg 1
Nanog
FP 2.9kb RP
1kb ladder NC C1 C2 C3
2.9kb
Sequencing confirmation:
B
Relative expression
OCT4
SOX2
SL
SLPD
SLCHIR
SL2i
2iL
C
SL
SLPD
SLCHIR
2iL
SL2i
Relativep-ERK1/2 level
D
8h
12h
16h
24h
Relativep-ERK1/2 levels
SL PD CHIR 2iL SL2i
E
PD (1µM)
CHIR (µM)
p-ERK1/2
ERK1/2
HDAC2
F
PD (µM)
CHIR (3µM)
G
PD (1µM)
CHIR (µM)
H
CHIR (3µM)
PD (µM)

Figure EV1.

Figure EV2. FGF autocrine signaling pathway components are essential for *Nanog* autoregulation.

A Immunofluorescence of NANOG in TNERT cells after 30 min treatment with or no OHT. Scale bars 5 μM.
B (left) Schematic of Doxycycline inducible TDiN cell line, generated by the introduction of a Tetracycline inducible Flag-Avi-NANOG transgene in Tβc44Cre6 cell line. (Right) Western blot of NANOG in TDiN cells after 48 h treatment with or no Doxycycline.
C FACS profiles of TNERT treatment with or no OHT in SLCHIR and SL2i. The dotted line represents the FACS profile of unstained E14Tg2a cells used as negative control (−ve c).
D FACS profiles of TDiN cell line in SL, SLCHIR, 2iL, SL2i, and SLPD. The dotted line represents the FACS profile of unstained E14Tg2a cells used as negative control (−ve c). (Bottom right) *Nanog*:GFP population median of TDiN in indicated treatments ($n = 3$).
E *Nanog*:GFP population median of TNERT, and TDiN treated with SU5402, with OHT/Doxycycline or no OHT/Doxycycline.
F (top) CRISPR-based knock-out strategy using paired sgRNA to knock-out *Fgf4* in TNERT. The sgRNAs are positioned at the beginning and the near end of exon II. The deletion results in the loss of the start codon and a part of the coding region in exon II. The dotted line represents the deleted region of the gene. FP and RP represent the relative positions of the genotyping primers. (Middle left) genotyping PCR of TNERTFgf4$^{-/-}$ clones. The WT allele gives an amplicon of 332 bp and the knock-out allele has a smaller amplicon by 32 bps or more. (Middle right) The relative abundance of FGF4 in media of TNERT and TNERTFgf4$^{-/-}$ clones 48 h after OHT treatment. (Bottom) Chromatogram of the TNERTFgf4$^{-/-}$ clones showing the sequences at the junction of the deletion.
G (top) Schematic of the gene structure of *Fgfbp1* and the relative positions of the two sgRNAs used for paired sgRNA knock-out strategy. One sgRNA is complimentary to 5′UTR and the other to the 3′ end of the coding region of the only exon. (Middle left) Genotyping PCR showing a WT amplicon of 1,220 bps and an amplicon around 400 bps in case of deletion. (Middle right) The relative abundance of FGFBP1 in media of TNERT and TNERTFgfbp1$^{-/-}$ clones 48 h after OHT treatment. (Bottom) Chromatogram of the TNERTFgfbp1$^{-/-}$ clone showing the sequences at the junction of the deletion.
H Strategy for knock-out of *Fgfr1* in TNERT cells. The schematic depicts the gene structure of *Fgfr1* with the relative positions of the two sgRNAs. One sgRNA targets the 3′end of Intron 8 and the other exon10. (Middle left) Genotyping PCR shows a WT allele amplicon at 494 bp and a knock-out allele with smaller amplicons around 150 bp. (Middle right) Western blot analysis of FGFR1 in TNERT and TNERTFgfr1$^{-/-}$ clones. (Bottom) chromatogram showing the sequence of the deleted region.
I A paired sgRNA strategy to knock-out *Fgfr2* in TNERT. (Top) The schematic represents the *Fgfr2* gene structure, with relative positions of the sgRNAs. One sgRNA target exon2 and the other sgRNA targets the coding region of the last exon approximately 100 kb apart. The dotted line represents the region of deletion in the *Fgfr2* gene. (Middle left) PCR genotyping shows a 665 bp amplicon when at least one allele of *Fgfr2* is deleted. This genotyping strategy cannot distinguish between +/− and −/− genotypes. (Middle right) Western blot analysis of FGFR2 protein in the *Fgfr2* targeted clones distinguishing the +/− and −/− clones. (Bottom) chromatogram represents the sequence of the genotyping amplicon indicating the exact sites of deletion.

Data information: $n \geq 3$ biological replicates (each dot represents a biological replicate). Data are presented as mean ± SEM in D and E. $^{**}P < 0.01$, $^{***}P < 0.001$, $^{****}P < 0.0001$ and ns = not significant (paired two-tailed Student's *t*-test).
Source data are available online for this figure.

 © 2022 The Authors

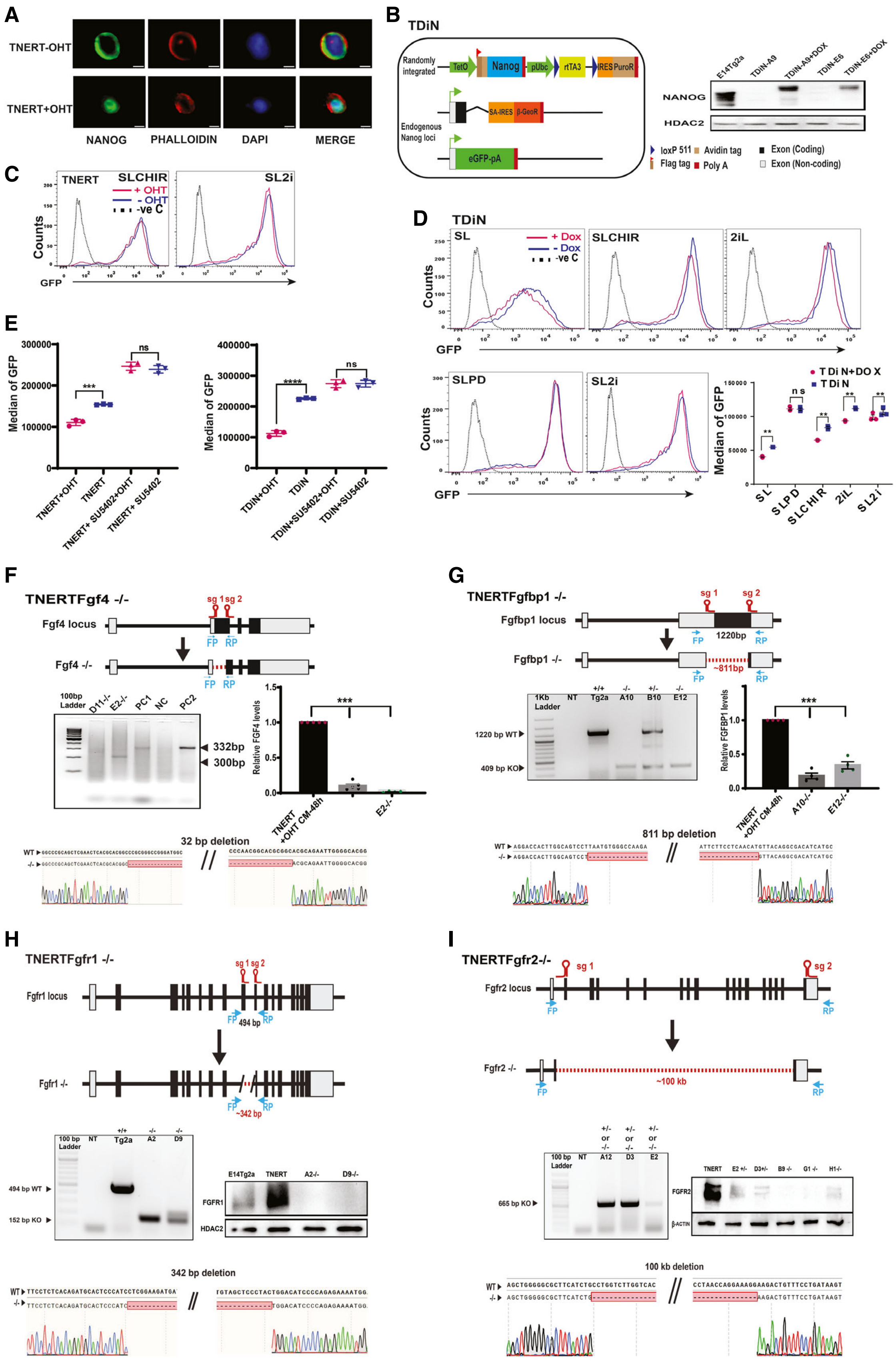
A
TNERT-OHT
TNERT+OHT
NANOG
PHALLOIDIN
DAPI
MERGE
B
TDiN
Randomly integrated
TetO
Nanog
pUbc
rtTA3
IRES PuroR
Endogenous Nanog loci
SA-IRES
β-GeoR
eGFP-pA
E14Tg2a
TDIN-A9
TDIN-A9+DOX
TDIN-E6
TDIN-E6+DOX
NANOG
HDAC2
loxP 511
Avidin tag
Exon (Coding)
Flag tag
Poly A
Exon (Non-coding)
C
TNERT
SLCHIR
+ OHT
- OHT
-ve C
SL2i
Counts
GFP
D
TDiN
SL
+ Dox
- Dox
-ve C
SLCHIR
2iL
SLPD
SL2i
TDiN+DOX
TDiN
Median of GFP
E
Median of GFP
TNERT+OHT
TNERT
TNERT+ SU5402+OHT
TNERT+ SU5402
TDiN+OHT
TDiN
TDiN+SU5402+OHT
TDiN+SU5402
ns
F
TNERTFgf4 -/-
Fgf4 locus
Fgf4 -/-
sg 1
sg 2
FP
RP
100bp Ladder
D11-/-
E2-/-
PC1
NC
PC2
332bp
300bp
Relative FGF4 levels
TNERT +OHT CM-48h
32 bp deletion
G
TNERTFgfbp1 -/-
Fgfbp1 locus
Fgfbp1 -/-
1220bp
~811bp
1Kb Ladder
NT
Tg2a
A10
B10
E12
1220 bp WT
409 bp KO
Relative FGFBP1 levels
A10-/-
E12-/-
811 bp deletion
H
TNERTFgfr1 -/-
Fgfr1 locus
Fgfr1 -/-
494 bp
~342 bp
100 bp Ladder
A2
D9
494 bp WT
152 bp KO
E14Tg2a
TNERT
A2-/-
D9-/-
FGFR1
HDAC2
342 bp deletion
I
TNERTFgfr2-/-
Fgfr2 locus
Fgfr2 -/-
~100 kb
665 bp KO
A12
D3
E2
FGFR2
β-ACTIN
100 kb deletion

Figure EV2.

Figure EV3. *Nanog* enhances the expression of FGF autocrine signaling pathway components.

A Browser tracks of NANOG enrichment in Fragment Per Kilobase of transcripts per Million (FPKM) in Oct4$^{+/+}$ cells (normal NANOG levels) and Oct4$^{+/-}$ cells (higher NANOG levels at *Fgf4*, *Fgfbp1*, *Fgfr1*, and *Fgfr2* loci) (Data ref: Karwacki-Neisius *et al*, 2013b).

B Histogram of FGFR1 expression on the cell surface analyzed by immunostaining and FACS of fixed but unpermeabilized TNERT cells treated with (red) or no OHT (blue). The dotted line represents the FACS profile of E14Tg2a cells stained with control IgG and secondary antibody used as negative control (−ve c).

C Relative expression levels of pERK, FGFR2, and FGFR1 at indicated time points after OHT treatment in TNERT cells ($n > 3$).

D ELISA-based relative quantification of FGF4 and FGFBP1in media from EDiN cells cultured with or no Doxycycline ($n = 3$). EDiN cell was generated by introducing a Doxycycline inducible Flag-Avi-NANOG transgene in E14Tg2a cells.

E *Nanog*:GFP population median of Tβc44Cre6 treated with OHT-induced conditioned media collected after different time points ($n = 3$).

F (top) Schematic of TNERTZfp281$^{-/-}$ cells, (upper-middle) CRISPR-based paired guide knock-out strategy indicating the relative position of the sgRNAs, FP and RP indicate the genotyping primers. (Lower middle) Genotyping PCR indicating +/− and −/− clones. (Bottom) The sequencing chromatogram of the deleted region confirms the exact site of deletion, followed by RT-qPCR analysis of the *Zfp281* transcripts.

G *Nanog*:GFP population median of Tβc44Cre6 treated with conditioned media from TNERT + OHT 0 h, TNERTFGF4$^{-/-}$ + OHT 48 h, Tβc44Cre6 48 h, E14Tg2a-FGF4-OE (overexpression) 48 h, TNERT + OHT 48 h and 50 ng/ml FGF4 ($n = 3$).

H, I ELISA-based relative quantities of FGF4 and FGFBP1 in media from TNERT after 18, 24, and 48 h of OHT treatment ($n = 3$).

J ELISA-based relative quantities of FGF4 in conditioned media from cell lines—TNERT + OHT 0 h, TNERTFGF4$^{-/-}$ + OHT 48 h, E14Tg2a-FGF4-OE 48 h (overexpression), TNERT−/+OHT 48 h, and 50 ng/ml FGF4 ($n = 3$).

K ELISA-based relative quantities of FGFBP1 in conditioned media from various cell lines—TNERT + OHT 0 h, TNERT-Fgfbp1$^{-/-}$ 48 h + OHT, TNERT 48 h −/+OHT, and 50 ng/ml FGBP1 ($n = 3$).

Data information: $n \geq 3$ biological replicates (each dot represents a biological replicate). Data are presented as mean ± SEM in C and D–K. *$P < 0.05$, **$P < 0.01$, ***$P < 0.001$, ****$P < 0.0001$ and ns = not significant (paired two-tailed Student's *t*-test).

 © 2022 The Authors

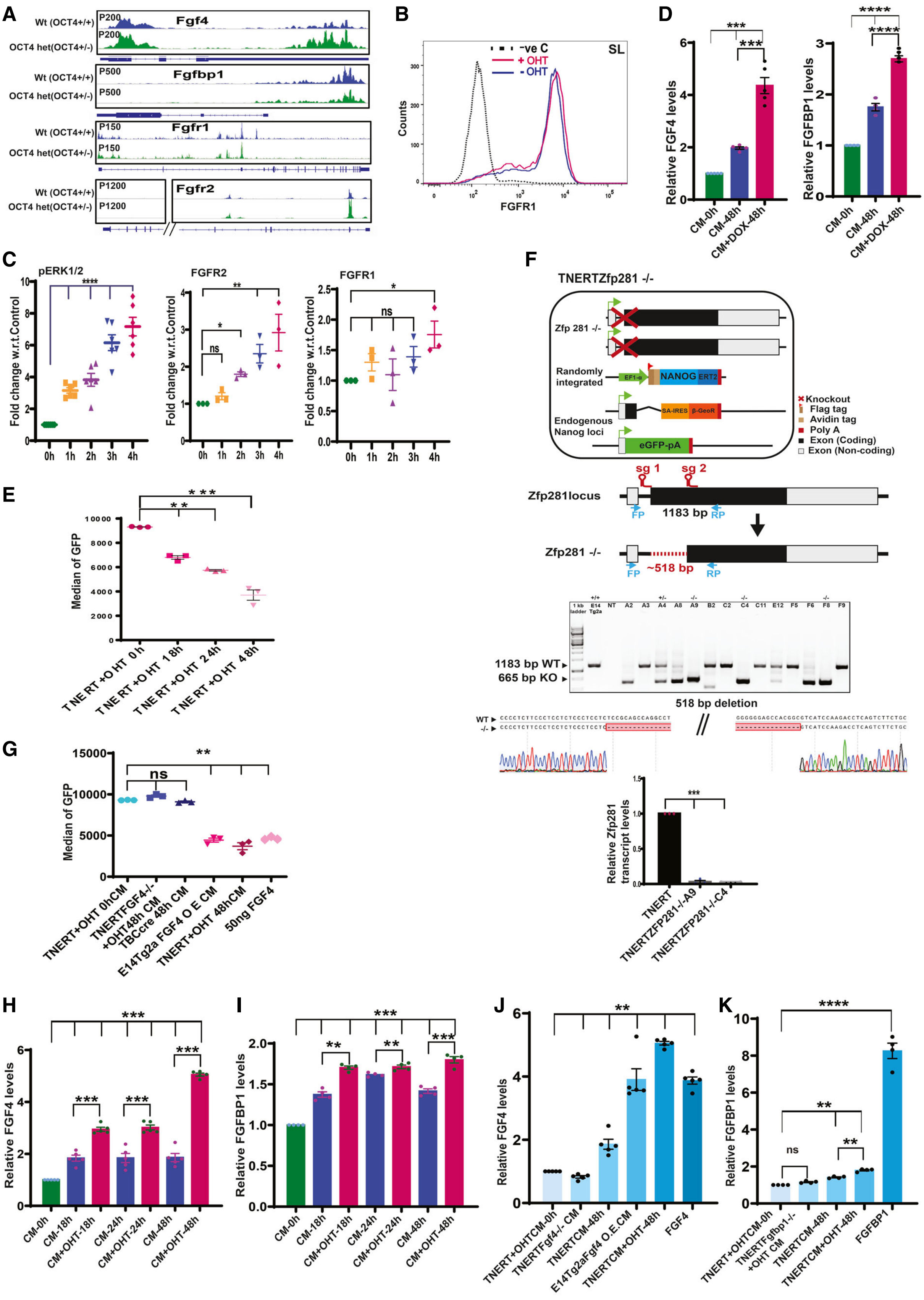
A
Fgf4
Wt (OCT4+/+)
OCT4 het(OCT4+/-)
Fgfbp1
Fgfr1
Fgfr2
B
-ve C
+ OHT
- OHT
SL
Counts
FGFR1
C
pERK1/2
FGFR2
FGFR1
Fold change w.r.t.Control
D
Relative FGF4 levels
Relative FGFBP1 levels
CM-0h
CM-48h
CM+DOX-48h
E
Median of GFP
TNERT+OHT 0h
TNERT+OHT 18h
TNERT+OHT 24h
TNERT+OHT 48h
F
TNERTZfp281 -/-
Zfp 281 -/-
Randomly integrated
NANOG
Endogenous Nanog loci
eGFP-pA
Knockout
Flag tag
Avidin tag
Poly A
Exon (Coding)
Exon (Non-coding)
Zfp281locus
Zfp281 -/-
1183 bp
~518 bp
1183 bp WT
665 bp KO
518 bp deletion
Relative Zfp281 transcript levels
TNERT
TNERTZFP281-/-A9
TNERTZFP281-/-C4
G
Median of GFP
TNERT+OHT 0hCM
TNERTFGF4-/- +OHT48h CM
TBCcre 48h CM
E14Tg2a FGF4 O E CM
TNERT+OHT 48hCM
50ng FGF4
H
Relative FGF4 levels
I
Relative FGFBP1 levels
J
Relative FGF4 levels
K
Relative FGFBP1 levels

Figure EV3.

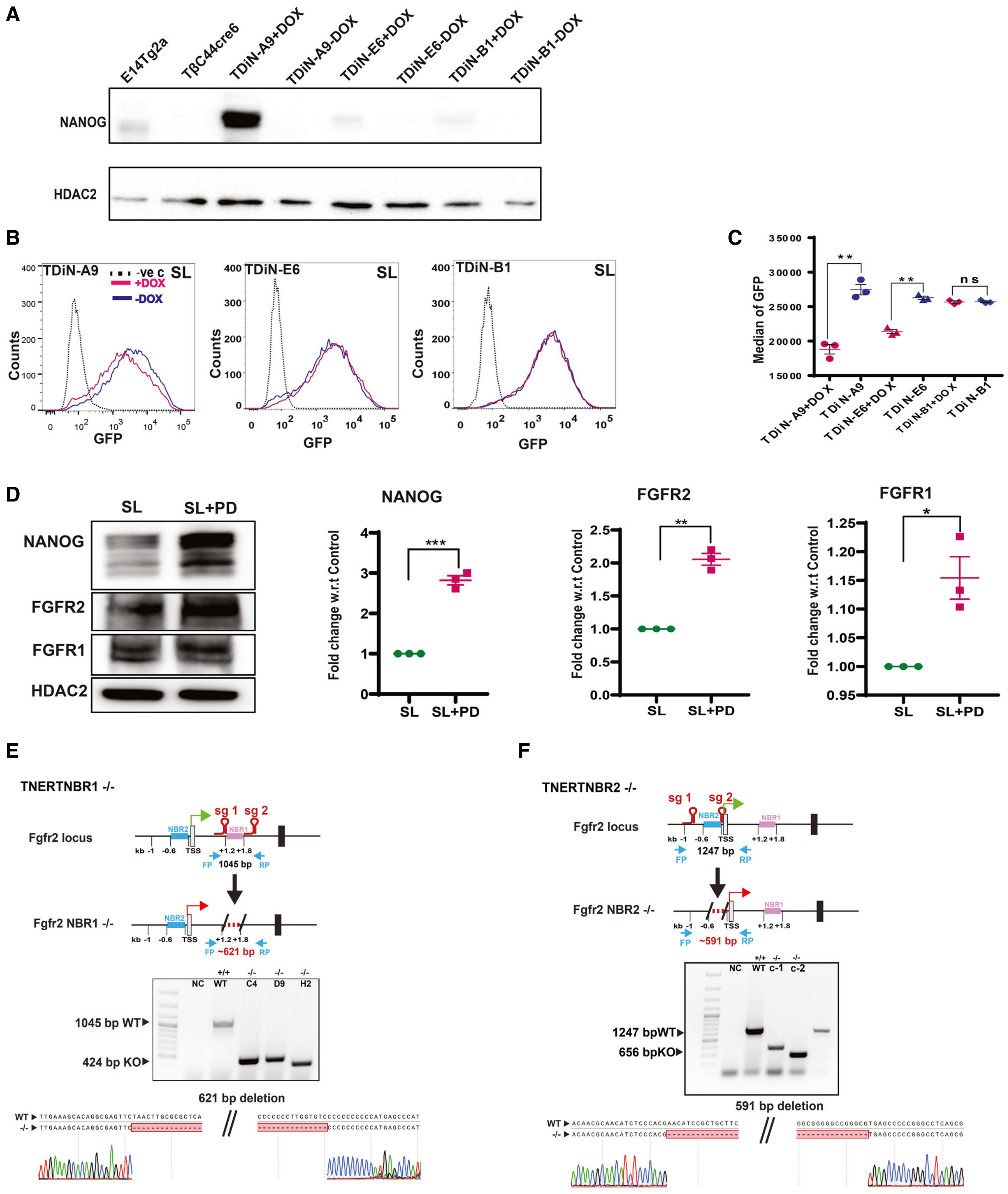

Figure EV4.

© 2022 The Authors

Figure EV4. NANOG-induced FGFR2 triggers autoregulation predominately in the ES cell population with higher *Nanog* expression.

A Western blot analysis of Flag-Avi-NANOG in different clones of TDiN treated with or no Doxycycline showing different levels of NANOG expression relative to E14Tg2a. The clones show different levels of expression Flag- Avi-NANOG upon Doxycycline treatment.
B FACS profiles of *Nanog*:GFP in TDiN clones treated with or no Doxycycline. The dotted line represents the FACS profile of unstained E14Tg2a cells used as negative control (−ve c).
C *Nanog*:GFP population median of TDiN clones ($n = 3$).
D (left) Western blot of NANOG, FGFR2, and FGFR1 in E14Tg2a cultured in SLPD for 48 h. (Right) Fold change in expression of NANOG, FGFR2, and FGFR1 in E14Tg2a cultured in SLPD relative to SL ($n = 3$).
E (top) Schematic of strategy for deletion of NANOG Binding Region 1 (NBR1) in TNERT indicating the position of the NBR1 and the relative position of the sgRNA pair. The sgRNAs are complementary to sequences around 1.2 kb and 1.8 kb downstream of TSS. FP and RP indicate the relative position of the primers for genotyping. (Middle) Genotyping of TNERT NBR1 knock-out clones. The WT shows an amplicon of 1,045 bp, upon deletion around 600 bp sequence comprising multiple NANOG binding sites is deleted. (Bottom) Sequence and chromatogram of the genotype PCR amplicon indicating the exact sequence of the junction of deletion in TNERTNBR1$^{-/-}$ clone.
F Schematic of strategy for deletion of NANOG Binding Region 2 (NBR2) in TNERT indicating the position of NBR2 and relative position of the sgRNA pair. The sgRNAs are complementary to sequences around TSS and 0.6 kb upstream of TSS of *Fgfr2*. (Middle) Genotyping of the TNERTNBR2 knock-out clones. The WT shows an amplicon of 1,247 bp. The knock-out would lead to deletion of around 690 bps and a smaller amplicon of around 650 bps. (Bottom) Sequence and chromatogram of the PCR amplicon from TNERT knock-out clones showing the exact site of deletion in TNERT NBR2$^{-/-}$ clone.

Data information: $n \geq 3$ biological replicates (each dot represents a biological replicate). Data are presented as mean ± SEM in C and D. *$P < 0.05$, **$P < 0.01$, ***$P < 0.001$ and ns = not significant (paired two-tailed Student's *t*-test).
Source data are available online for this figure.

Figure EV5. ERK interacts and recruits NONO to repress *Nanog* transcription.

A (left) FACS profile of TNERTTcf15$^{-/-}$ treated with or no OHT ($n = 3$). The dotted line represents the FACS profile of unstained E14Tg2a cells used as negative control (−ve c). (Right) *Nanog*:GFP population median of TNERT and TNERTTcf15$^{-/-}$ treated with or no OHT ($n = 3$).
B A CRISPR-based knock-out strategy using paired sgRNA, to knock-out of *Nono* in TNERT cells. (Top) The schematic represents the mouse *Nono* gene structure with relative positions of the two sgRNAs flanking the second coding exon of *Nono*. FP and RP indicate the relative position of genotyping primers. The dotted line indicates the region of deletion in the *Nono* gene. (Middle) Genotyping PCR of the *Nono*$^{-/-}$ deletion in TNERT. The WT allele gave an amplicon of 711 bp and the deleted allele shows a smaller amplicon of 330 bp; followed by sequence and chromatogram indicating the deletion site (bottom) Western blot analysis of NONO protein in TNERT and TNERTNono$^{-/-}$ clones.
C (C)*Nanog*:GFP population median of TNERT and TNERTNono$^{-/-}$ treated with or no OHT ($n = 3$).
D The relative abundance of pERK in TNERT treated with or no OHT and TNERTNono$^{-/-}$ with OHT ($n = 4$).
E Browser tracks of pERK, NONO, POL2, H3K4me3, H3K27me3 enrichment in Fragment Per Kilobase of transcripts per Million (FPKM) on *Nanog* gene (Data ref: Ma *et al*, 2016b; Data ref: Tee *et al*, 2014b).
F ChIP-qPCR analysis of POL2 enrichment on Nanog locus in E14Tg2a (WT) and Nono$^{-/-}$ cell lines. Refer to the schematic in Fig 5F for the coordinates of S1–S9.
G ChIP-qPCR analysis of pERK, NONO, H3K27, H3K4, and POL2 enrichment on a non-genic region and a known validated target locus. The non-genic region named ORF Free Region (OFR) corresponds to Chr6:43023477 + 43023592 (mm10). *HoxC11* and *Oct4* were used as a known validated target genes for ChIP-qPCR.
H The relative abundance of NANOG after 0, 2, 4, 6, and 8 h of Cycloheximide (CHX) chase cultured in SL, PD, and FGF4 ($n = 3$).

Data information: $n \geq 3$ biological replicates (each dot represents a biological replicate). Data are presented as mean ± SEM in A, C, D, and F–H. *$P < 0.05$, **$P < 0.01$, ***$P < 0.001$, ****$P < 0.0001$ and ns = not significant (paired two-tailed Student's *t*-test).
Source data are available online for this figure.

© 2022 The Authors

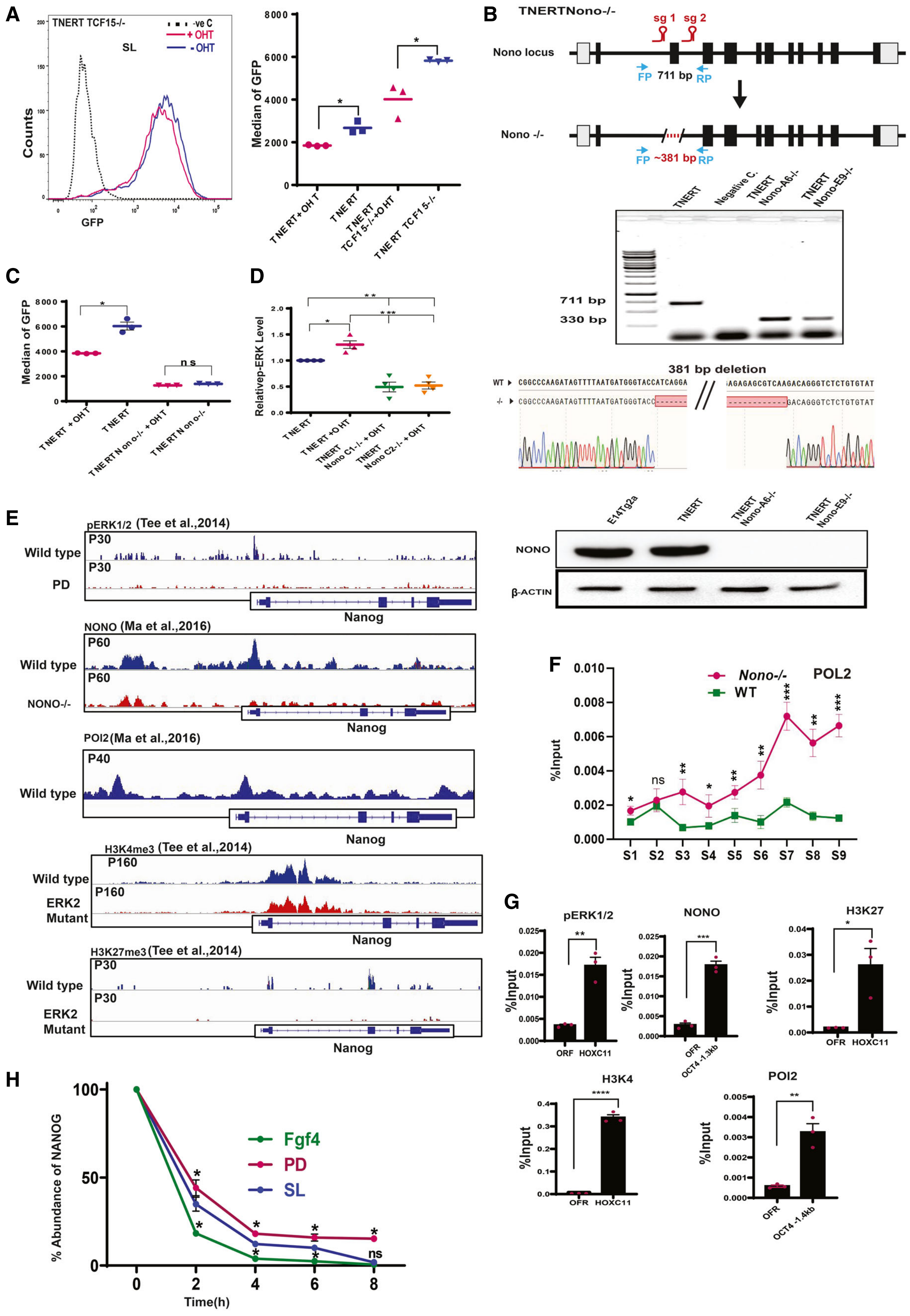
A
TNERT TCF15-/-
SL
-ve C
+ OHT
- OHT
Counts
GFP
Median of GFP
B
TNERTNono-/-
sg 1 sg 2
Nono locus
FP 711 bp RP
Nono -/-
FP ~381 bp RP
711 bp
330 bp
381 bp deletion
C
D
Relativep-ERK Level
E
pERK1/2 (Tee et al.,2014)
Wild type
PD
Nanog
NONO (Ma et al.,2016)
NONO-/-
POl2 (Ma et al.,2016)
H3K4me3 (Tee et al.,2014)
ERK2 Mutant
H3K27me3 (Tee et al.,2014)
NONO
β-ACTIN
F
Nono-/-
WT
POL2
%Input
G
pERK1/2
NONO
H3K27
H3K4
POl2
H
% Abundance of NANOG
Fgf4
PD
SL
Time(h)

Figure EV5.

A NANOG-pERK reciprocal regulatory circuit mediates *Nanog* autoregulation and ERK signaling dynamics.

Hanuman T. Kale, Rajendra Singh Rajpurohit, Debabrata Jana, Vijay V. Vishnu, Mansi Srivastava, Preeti R. Mourya, Gunda Srinivas, P. Chandra Shekar*

Table of Contents

Appendix Figure S1 – page 2

Appendix Figure S2 – page 3

Appendix Table S1. Oligonucleotide sequences used in this study – page4

Appendix Table S2. Reagents used in this study- page 7

Appendix Figure S3 -Myoplasma analysis- page 10

Appendix Figure S4 - STR analysis- page 11

Appendix Table S3. Oligonucleotide sequences used for STR analysis- page 12

Appendix references -page 13

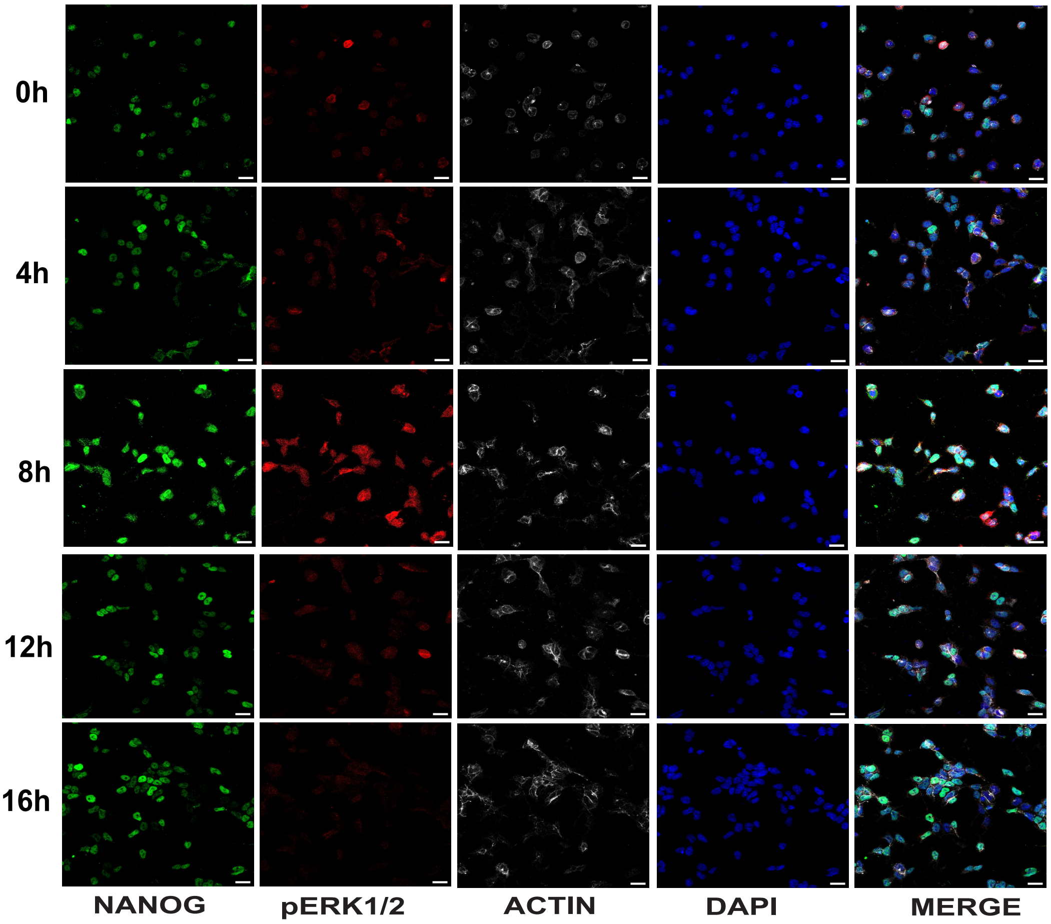

Appendix Figure S1. Data related to Fig 6.

NANOG regulates ERK signaling dynamics and heterogeneity.

Immunocytochemistry at different time intervals of culture of NiRFP2A ESCs after sorting of 10% of Nanog-high cells. pERK (red), NANOG (green) and nuclear stain (blue). pERK and NANOG were detected in 10% Nanog-high cells immediately after sorting. Most of the cells expressed NANOG and few of these cells expressed pERK also. pERK showed very high heterogeneity at 0 hrs. pERK expression increased gradually with further increase in NANOG until 8 hrs. pERK1//2 was highest at 8 hrs with concomitant high expression of NANOG. Most of the cells expressed pERK by 8 hrs. pERK expression decrease after 8 hrs coinciding with decrease of NANOG. few cells expressed pERK by 12 hrs. The pERK expression further decreased by 16hrs followed by decrease in NANOG expressing cells. Scale bar 25 µM.

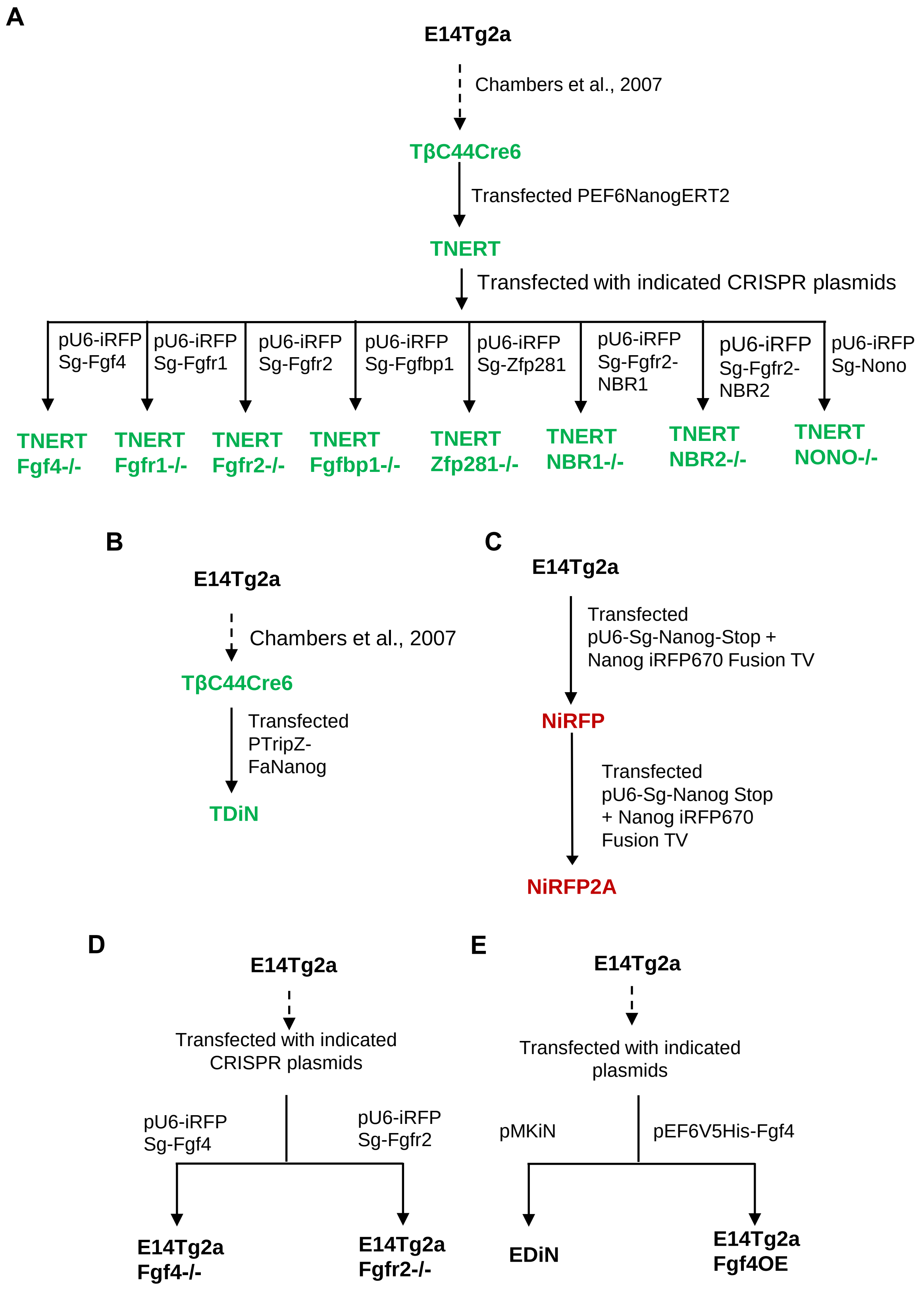

Appendix Figure S2: Information related to methods. A pedigree chart of cell lines used in this study

(A) Flow chart illustrating the lineage and process of generation of TNERT and generation of knock out cells lines in TNERT background. (B) A flow chart describing derivation of TDiN. (C) A flow chart depicting derivation of NiRFP2A from E14Tg2a. (D) A flow chart depicting derivation of Fgf4-/- and Fgfr2-/- ES cell lines from E14Tg2a. (E) A flowchart depicting derivation of EDiN and Fgf4OE (over expression) ES cell lines from E14Tg2a.

Appendix Table S1. Oligonucleotide sequences used in this study.

Name of the Oligo	Sequence
**Oligos for paired sgRNA Knock-out**	
Fgf4 sgRNA1	CACCGCCGTGCGTGAGTTCGAGCTG
	AAACCAGCTCGAACTCACGCACGGG
Fgf4 sgRNA2	CACCGCGAAACGCGGGCCGACCAC
	AAACGTGGTCGGCCCGCGTTTCGC
Fgfr1 sgRNA1	CACCGCGTCATCATCTTCCGAGGAT
	AAACATCCTCGGAAGATGATGACGC
Fgfr1 sgRNA2	CACCGTTCTCTGGGGATGTCCAGTA
	AAACTACTGGACATCCCCAGAGAAC
Fgfr2 sgRNA1	CACCGCTGGGGGCGCTTCATCTGCC
	AAACGGCAGATGAAGCGCCCCCAGG
Fgfr2 sgRNA2	CACCGCTCAGTGTAAGTAGGTTCC
	AAACGGAACCTACTTACACTGAGC
Fgfbp1 sgRNA1	CACCGCACAGTCTTGGCCCACATTA
	AAACTAATGTGGGCCAAGACTGTGC
Fgfbp1 sgRNA2	CACCGATGTCGCCTGTAACATGTTG
	AAACCAACATGTTACAGGCGACATC
Fgfr2 NBR1 sgRNA1	CACCGTCGGAGCAGCTAGGCGAACT
	AAACAGTTCGCCTAGCTGCTCCGAC
Fgfr2 NBR1 sgRNA2	CACCGTTGTGTTTAGGGCCCCCCCT
	AAACAGGGGGGGCCCTAAACACAAC
Fgfr2 NBR2 sgRNA1	CACCGTGGAAGCAGCGGATGTTCGT
	AAACACGAACATCCGCTGCTTCCAC
Fgfr2 NBR2 sgRNA2	CACCGACCGGAGCTGCTCTCGGATC
	AAACGATCCGAGAGCAGCTCCGGTC
Nanog -stop codon sgRNA	CACCGTATGAGACTTACGCAACATC
	AAACGATGTTGCGTAAGTCTCATAG
Zfp281 sgRNA1	CACCGAGGCCTGGCTGCGGAGAGG
	AAACCCTCTCCGCAGCCAGGCCTC
Zfp281 sgRNA1	CACCGCGGGGTATGAAAATCGGCAG
	AAACCTGCCGATTTTCATACCCCGG
Nono sgRNA1	CACCGTTTTAATGATGGGTACCATC
	AAACGATGGTACCCATCATTAAAAC
Nono sgRNA1	CACCGTTCCGAGAGAGCGTCAAGAC
	AAACGTCTTGACGCTCTCTCGGAAC
**Genotyping primers**	
Fgf4 knock-out	CGCAGCACTCACCGAACTCA
	TGCCCACGTTGCAGTAGAGC
Fgfr1 knock-out	GTCTAGACAGGGCGAATGCTGTTT
	ACTTGAACTTCACCGTCTTGGCAG
Fgfr2 knock-out	TCTGGGTTTAAGCAAGTTGGCACT
	CAGCATACATGGTGGGTCAGAGAG
Fgfbp1 knock-out	GAAAGTGAGAAGCTGAGTGAATGG
	TATATGCACCTAGGTTTGTGGTCC
Fgfr2 NBR1 knock-out	GAAGAAACTGCTGGAGTGTGGTCA
	AGGGTAGTTCCAGGATACCTCAGC
Fgfr2 NBR2 knock-out	AGAGGCTTTGGATGACTCTGCAAC
	GCGATGATCTCGGAGGAAAACTCC

Zfp281 knock-out	TGTGGAGAGGACGGCGTTATTTTT
	TGAAACCCATACTACACTGGCTGC
Nono Knock out	AGGTTTCCCTGCTTGTCTTTGTCT
	GCAGCAACGCCCTTAATTTCAACA
Nanog-iRFP670	ACCCAGGGGTGACAAAGTATTCCAA
	GCATTTTCCGTAATGCGCGTGATCC
Nanog-sfGFP	ACCCAGGGGTGACAAAGTATTCCAA
	CGTTTGTAGCATCACCTTCACCCTC
Nanog-T2AmCherry	ACCCAGGGGTGACAAAGTATTCCAA
	TCGCCCTTGCTCACCATTGGCCCGGGATTCTCTTC
**q-RT-PCR primers**	
	CTGCCATTAATGTGGCCATCCC
Dusp6	GTGTTCTCATTCCAGTCGCTGC
	TGGTCCCCACAGTTTGCCTAGTTC
Nanog	CAGGTCTTCAGAGGAAGGGCGA
	GTGGAGGAAGCCGACAACAATGA
Oct4	CAAGCTGATTGGCGATGTGAG
	TTTTCTAGTCGGCATCACCG
Sox2	ACAAGAGAATTGGGAGGGGT
	GTGCAGCTTGCAGCAGTAAC
Klf4	AGCGAGTTGGAAAGGATAAAGTC
	CAGTCCAGAATACCAGAGTGGAA
Rex1	ACTCTAGGTATCCGTCAGGGAAG
	CCCTGCGGAGACAGGTAACAG
Fgfr2	AGCGTCAGCTTATCTCTGGGGA
	ACCCTGTAGCTCCCTACTGGAC
Fgfr1	TGGCATAGCGAACCTTGTAGCC
	ACACGAGGGACAGTCTTCTGGA
Fgf4	TAGGCGTTGTAGTTGTTGGGCA
	CTAACCTCAAGCTGGTGAACCCC
Fgfbp1	TCTCTAATGGCCATGGTCTGGGT
	CAACGGCACAGTCAAGGCCGA
Gapdh	CCCTTCAAGTGGGCCCCGG
	CCACCGATTGCTTGGAAGTTGG
Spry2	AGGTCTTGGCAGTGTGTTCACC
	AGCCCAGTACTCAGGCTTGT
Pre-*Nanog-1*	AGCATCACAACACGCACCT
	GCCAGCAGATGGCATAATTT
Pre-*Nanog-2*	TGATGGCAATGCTGAGGTTA
	GTCCCAGCTGGTGTGACTCT
Pre-*Oct4*	TCTTCTGCTTCAGCAGCTTG
	GACTACCTGCTGGGCCTCAAAA
Pre-*Fgf4*	TACTCAGCCCCCGAGACTACTAC
	CCCGTCCCTTCCCAAATCTGATA
Pre-*Fgfr2*	TCACCTTGGGTCAGGATAACAAG
	GACTACCTGCTGGGCCTCAAAA
Pre-*Fgfbp1*	TACTCAGCCCCCGAGACTACTAC
Pre-*Fgfr1*	CCCGTCCCTTCCCAAATCTGATA
	TCACCTTGGGTCAGGATAACAAG

5'UTR Of Nanog	GGTGATACGTTGGCCTTCTAGT
	TTCTCAAATACACACAAGAGCCTTA
**q-ChIP-PCR primers**	
Nanog 5.5 kb	GTGGGTGCACACAGAGAACAAC
	CTGAGAGCTCAGGCCCACAAAG
Nanog -4.9 kb	AACATTCCTTTCCCCACCCACA
	AAGAGGTGGCTGGTAGCCAAAA
Nanog -4.7 kb	TGGGGTAAACTTAAGGCTATGG
	AGCTCTAAGCCGGTTCTCATTT
Nanog -3.9 kb	CCCTACCTCTCCTGAGGTGTGA
	CATGCCTGAGGAAGTCAGAGGA
Nanog -3.4 kb	TGTAGCCCTTGGTTAGTCCGAG
	GGCAGGCATCACCAAAGTCATT
Nanog -1.9 kb	GGTTCAGTCAGGCTGGGCAAT
	CTGCTGCCACACTATCACTGTC
Nanog -1.0 kb	AGCCGACTTAAGCTGGGTTAGA
	TGCTCTAGCTGGTCCCAAACTC
Nanog -0.05 kb	TAGGGTAGGAGGCTTGAGGGG
	AAGTCAGAAGGAAGTGAGCCGC
Nanog +.5 kb	CCGGTGATACGTTGGCCTTCTA
	ACTGCCCCCGAACATATTCCAA
Nanog +1.4 kb	GTTAGGAATGAACGGGTGGGGA
	AGTAGACAGCCCTGAAAGCAGC
Fgf4 +3.5 kb	GCCCAGAACCCAATTTTTATGCAC
	CAAAGTCCCAGAGCCATTCCCTT
Fgf4 -3.0 kb	TTAGCTCGCTTCAGGGAATGCTT
	TTGCTGTCTGTAGCCTCCCATAA
Fgf4 -6.5 kb	CACAAAGGTCGCTTAAGTGGTGG
	ACACGATTTCCAGACTCCTCCAG
Fgf4 -6.9 kb	TTAGGCACCCAAAGGCAGAATTG
	GTCCTGTTATTCATGGCAGGGGA
Fgfr1-5kb	TGGCCTTGGATGAATTGTTGGC
	TTCCACCTCCCTTCAGGACACT
Fgfr1+2.5 kb	TGGGGTGGTGTCTCTTCCTTTCAG
	CAAGCCATTAGGGAGGGAGGCAA
Fgfbp1-1.4 kb	TCCAGTGTGTGTGGTAAACAGGT
	AACACTGCCTCTGGATGGTCTAC
Fgfr2 -2.5 kb	TTTTGTCCCACTTCTTGGGGC
	AATCTTCCACCAGCCTGGACTC
Fgfr2 -4.4 kb	AAACAACGTAACGCATCCACTGT
	TGCACAGATGACCTCTCGGAAC
Oct4-0.3kb	TCTCCAGAGGATGGCTGAGTG
	CCAGGAGGCCTTCATTTTCAAC
HoxC11+0.13kb	CACCGTCTCTTCCTTCCTACCC
	GACGAGTAGCTGTTCCGATGGT
OFR-Chr6(43023477..43023592)	TGTGTGTTTGTGCACCGTGTGTGA
	TAGGTCCAATTCCCACCACCAGCAT

Appendix Table S2: Reagents used in this study

REAGENT or RESOURCE	SOURCE	IDENTIFIER
Antibodies		
Anti-NANOG	Thermo Fisher Scientific	Cat# 14-5761-80, RRID: AB_763613
Anti-NANOG	Cell signaling Technology	Cat# 8822, RRID: AB_11217637
Nanog Polyclonal Antibody	Thermo Fisher Scientific	Cat# PA5-47376, RRID: AB_2607022
Anti-OCT3/4	Cell signaling Technology	Cat# 83932, RRID: AB_2721046
Anti-OCT3/4	Thermo Fisher Scientific	Cat# 14-5841-82, RRID: AB_914301
Anti-SOX 2	Cell signaling Technology	Cat# AMAb91307, RRID: AB_2665892
Anti-FGFR2	Thermo Fisher Scientific	Cat# PA1-24763, RRID: AB_780623
Anti-FGFR2	R&D Systems	Cat# MAB6843, RRID: AB_2103395
Anti-FGFR1	Cell signaling Technology	Cat# 9740, RRID: AB_11178519
FGFBP1 Polyclonal Antibody	Thermo Fisher Scientific	Cat# PA5-77220, RRID: AB_2720947
Anti-FGF4	Thermo Fisher Scientific	Cat# PA5-20483, RRID: AB_11152903
Anti-ERK	Cell signaling Technology	Cat# 9102, RRID: AB_330744
Anti-P-ERK	Cell signaling Technology	Cat# 4370, RRID: AB_2315112
Anti-P-ERK	Cell signaling Technology	Cat#9101, RRID: AB_331646
Anti-Trimethyl Histone H3(Lys4) (C42D8)	Cell signaling Technology	Cat# 9751, RRID: AB_2616028)
Anti-Trimethyl Histone H3(Lys27)	Merck Millipore	Cat# 07-449, RRID: AB_310624
Anti- β-ACTIN	Sigma-Aldrich	Cat# A2228, RRID: AB_476697
Anti-HDAC2	Thermo Fisher Scientific	Cat# 51-5100; RRID: AB_2533908
Anti-RNA polymerase II Antibody, clone CTD4H8	Merck Millipore	Cat# 05-623, RRID: AB_309852
Nanog (eBioMLC-51)	Thermo Fisher Scientific	Cat#14-5761-80 RRID:AB_763613
Oct4	Thermo Fisher Scientific	Cat#14-5841-37RRID AB_2865147
Chemicals, Peptides, and Recombinant Proteins		
rhFGF4	R&D Systems	Cat# 7460-F4-025
rhFGFBP1	R&D Systems	Cat# 1593-FB-025
Human FGF4 recombinant protein	Thermo Fisher Scientific	Cat# PHG0154
Human BMP4 recombinant protein	Thermo Fisher Scientific	Cat# PHC9534
Human EGF recombinant protein	Thermo Fisher Scientific	Cat# 01-107

Human Insulin recombinant protein	Thermo Fisher Scientific	Cat# RP-10908
Human bFGF recombinant protein	Thermo Fisher Scientific	Cat# RP-8628
G418 disulfate salt	Sigma-Aldrich	Cat# A1720
Doxycycline Hyclate	Sigma-Aldrich	Cat# D9891
SU5402	Sigma-Aldrich	Cat# SML0443
PD0325901	Sigma-Aldrich	Cat# PZ0162
CHIR99021	Sigma-Aldrich	Cat# SML1046
(Z)-4-Hydroxytamoxifen	Sigma-Aldrich	Cat# H7904
LIF	Made in-house	
Heparan sulfate sodium salt	Sigma-Aldrich	Cat# H7640
o-Phenylenediamine dihydrochloride	Sigma-Aldrich	Cat# P8287
Deposited Data		
Experimental Models: Cell Lines		
E14Tg2a		Chambers et al., 2007
TNGA		Chambers et al., 2007
TβC44Cre6		Chambers et al., 2007
TNERT	This Study	
NiRFP2A	This Study	
TDiN	This Study	
OGNM	This Study	
EDiN	This Study	
NisGFPDiN	This Study	
NsGiR	This Study	
TNERTFgfr2-/-	This Study	
TNERTFgfr1-/-	This Study	
TNERTFgf4-/-	This Study	
TNERTFgfbp1-/-	This Study	
TNERTNBR1-/-	This Study	
TNERTNBR2-/-	This Study	
TNERTNono-/-	This Study	
E14Tg2aFgf4-/-	This Study	
E14Tg2aFgfr2-/-	This Study	
E14Tg2aFgf4OE	This Study	
Oligonucleotides		
Oligos used for sgRNA cloning, genotyping, qPCR – RTPCR and ChIP-PCR	Supplemental Table 1	
Recombinant DNA		
pU6-(BbsI)-CBh-Cas9-T2A-mCherry	Addgene 64324	(Weber et al., 2015)
Mouse Oct4-GFP GOF18 transgenic reporter	Addgene 60527	(Gafni et al., 2013)
Nanog iRFP670 Fusion Targeting vector	This Study	
Nanog sfGFP Fusion Targeting vector	This Study	
Nanog-2A-mCherry	Addgene 59995	(Faddah et al., 2013)
pEF6V5His-Fgf4	This Study	

PEF6NanogERT2	This Study	
PTripZ-FaNanog	This Study	
pU6-iRFP	This Study	
pU6-iRFP Sg-Fgf4	This Study	
pU6-iRFP Sg-Fgfr1	This Study	
pU6-iRFP Sg-Fgfr2	This Study	
pU6-iRFP Sg-Fgfbp1	This Study	
pU6-iRFP Sg-Zfp281	This Study	
pU6-iRFP Sg-Nono	This Study	
pU6-iRFP Sg-Fgfr2-NBR1	This Study	
pU6-iRFP Sg-Fgfr2-NBR2	This Study	
pU6-iRFP Sg-Nanog-Stop	This Study	
pMKiN	This Study	
Software and Algorithms		
ImageJ	ImageJ	RRID: SCR_003070
FlowJo	BD Bioscience	RRID: SCR_008520
Integrative genomics viewer	Broad Institute	RRID: SCR_011793
SDS	Applied Biosystems	RRID: SCR_015806
Image Lab	Bio-rad	RRID: SCR_014210
GraphPad Prism	GraphPad	RRID: SCR_002798
Other		
Pierce Protein A/G Magnetic beads	Thermo Fisher Scientific	Cat# 88803
BD LSR Fortessa	BD Bioscience	N/A
MoFLo XDP	Beckman Coulter	N/A
Gallios Flowcytometer	Beckman Coulter	N/A
Chemidoc MP imaging system	Bio-rad	
Zeiss Axio Observer	Zeiss	N/A

A

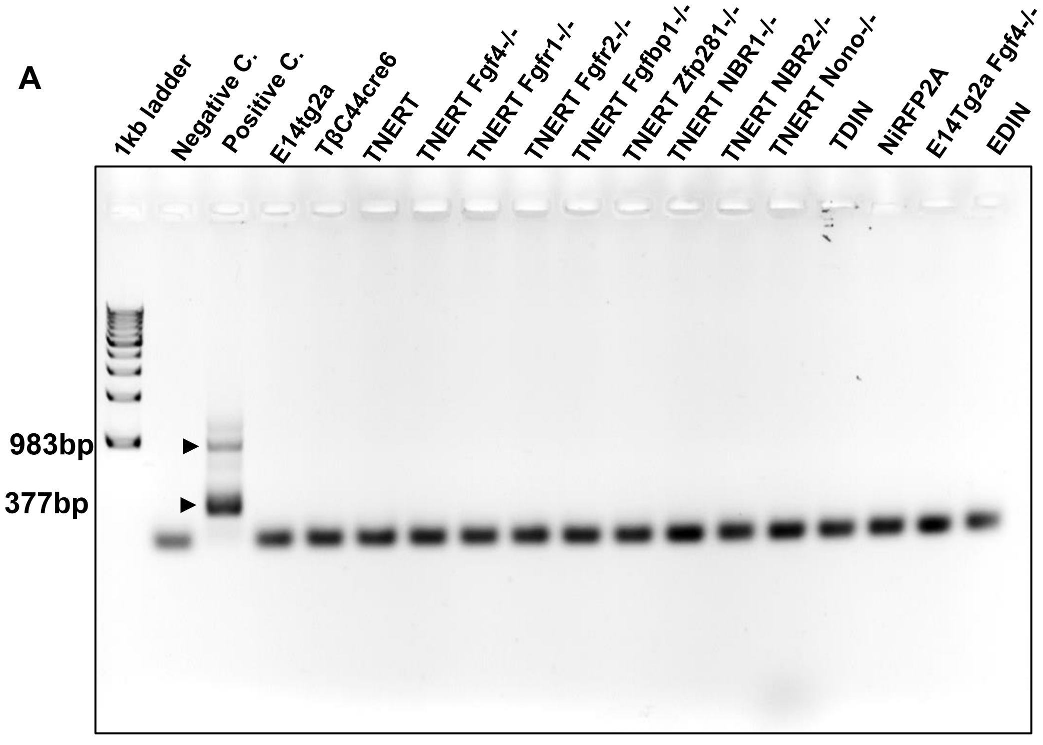

B

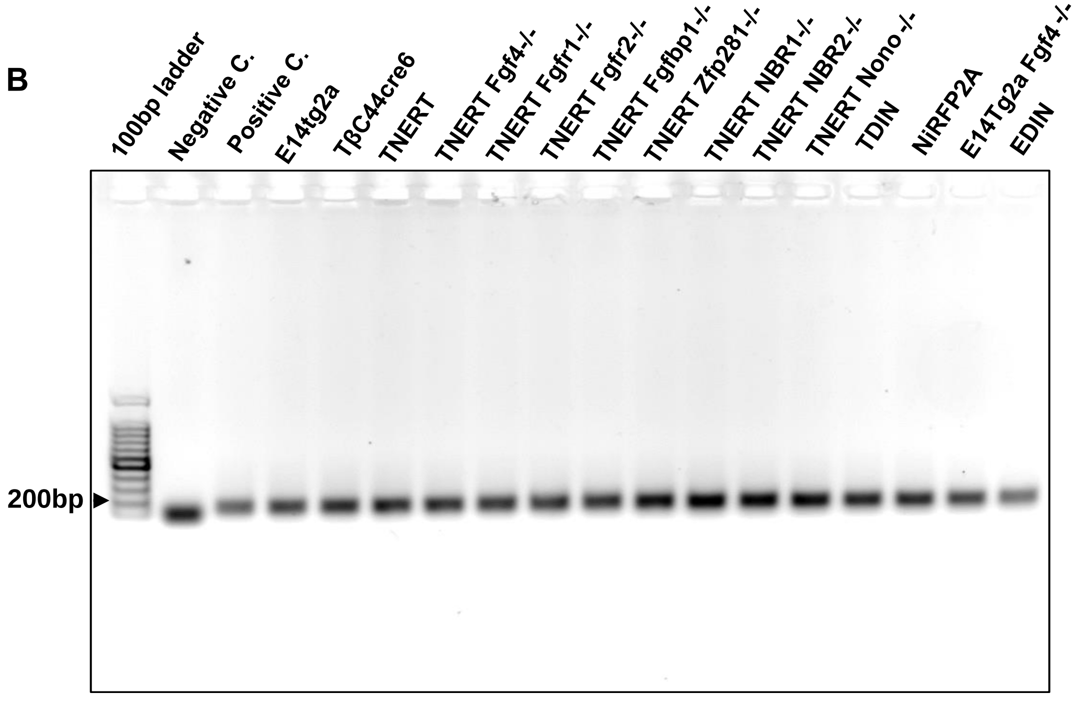

Appendix Figure S3: Mycoplasma contamination analysis of the cell lines using PCR detection.

(A) Agarose gel electrophoresis image of PCR performed to detect the presence of mycoplasma genome in genomic DNA isolated from the mentioned cell lines using lookOut mycoplasma PCR detection kit. Negative C. does not contain any template. Positive C. contains DNA template coding for 16sRNA of mycoplasma. (B) Agarose gel electrophoresis image of PCR performed to detect the presence of mouse genomic DNA and absence of PCR inhibitors in the DNA preparation used for mycoplasma detection using a primer set specific to mouse genomic DNA.

A

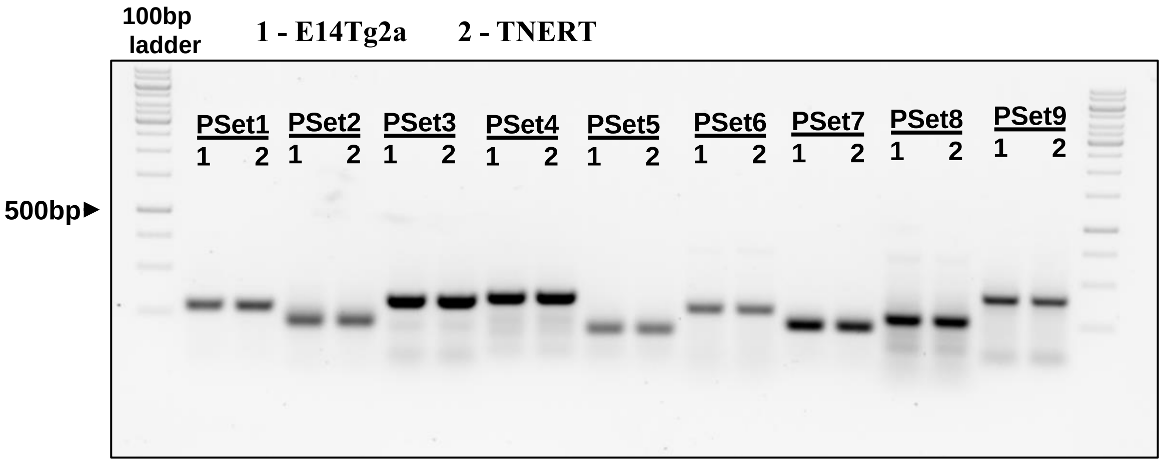

B

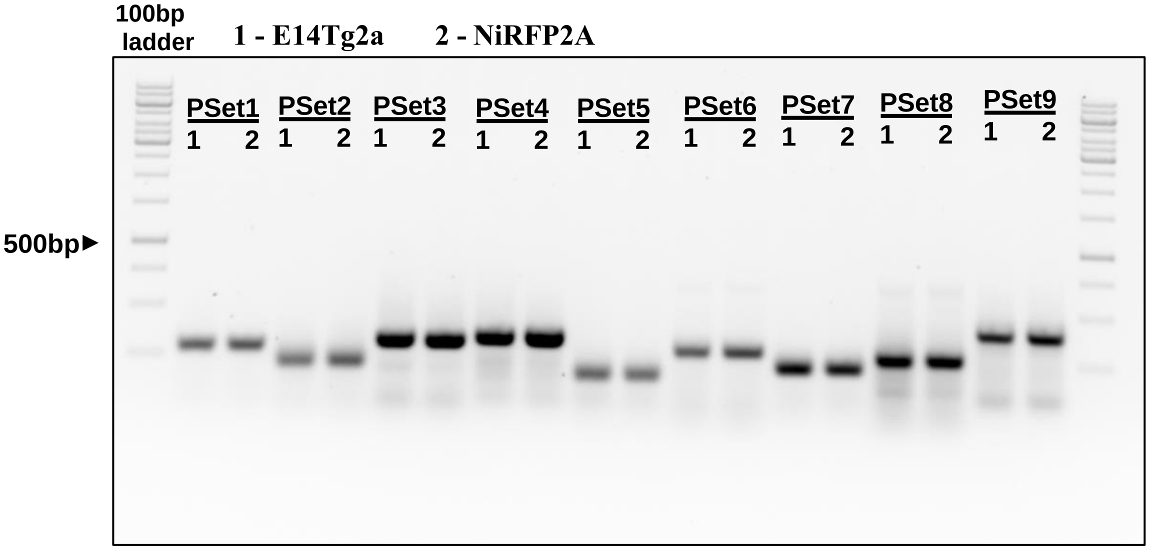

Appendix Figure S4: STR analysis to authenticate the identity of the cell lines

(A) Agarose gel electrophoresis image of PCR performed using primers specific to STR regions of mice. TNERT cell line showed identical STR profile to its parent cell line E14Tg2a. (B) Agarose gel electrophoresis image of PCR performed using primers specific to STR regions of mice. NiRFP2A cell line showed identical STR profile to its parent cell line E14Tg2a. The STR regions and sequence of the primers is given in Appendix table S3.

Appendix table S3: PSet- Primer set used for STR analysis

Primer Set (PSet)	Primer sequence	STR regions	Expected size
PSet 1	TCTTTCTCCTTTTGTGTCATGC	STR -18-3 F	281–313 bp
	TCAAAGTTGGGGTTACAGAATG	STR -18-3 F	
PSet 2	AAGCTTCTCTGGCCATTTGA	4-2 F	217–248bp
	TTCATAAACTTCAAGCAATGACA	4-2 R	
PSet 3	AGTCCACCCAGTGCATTCTC	6-7 F	333–515 bp
	CATGTGGCTGGTATGCTGTT	6-7 R	
PSet 4	GGCTCTCTCACACCTCATCC	9-2 F	318–360 bp
	TCCATGAATCCAGACATTCC	9-2 R	
PSet 5	TCTGGGCGTGTCTGTCATAA	15-3 F	157–222 bp
	TTCTCAGGGAGGAGTGTGCT	15-3 R	
PSet 6	TTTGCAACAGCTCAGTTTCC	6-4 F	276–311 bp
	AATCGCTGGCAGATCTTAGG	6-4 R	
PSet 7	CAAAATTGTCATTGAACACATGTAA	12-1 F	222–259 bp
	CAATGGTCAAGAAATACTGAAGTACAA	12-1 R	
PSet 8	CGTTTTACCTGGCTGACACA	5-5 F	258–298 bp
	GATGCTTGCCTGTTCCTAGC	5-5 R	
PSet 9	GGATGGATGGATGGATGAAA	X-1 F	357–442 bp
	AAGGTATATATCAAGATGGCATTATCA	X-1 R	

Appendix References

1. I. Chambers *et al.*, Nanog safeguards pluripotency and mediates germline development. **450**, 1230-1234 (2007).
2. D. A. Faddah *et al.*, Single-cell analysis reveals that expression of nanog is biallelic and equally variable as that of other pluripotency factors in mouse ESCs. **13**, 23-29 (2013).
3. T. Weber *et al.*, Increasing the efficiency of homology-directed repair for CRISPR-Cas9-induced precise gene editing in mammalian cells. *Nature biotechnology* **33**, 543-548 (2015).
4. O. Gafni *et al.*, Derivation of novel human ground state naive pluripotent stem cells. *Nature* **504**, 282-286 (2013).
5. N. Festuccia *et al.*, Esrrb is a direct Nanog target gene that can substitute for Nanog function in pluripotent cells. **11**, 477-490 (2012).
6. V. Karwacki-Neisius *et al.*, Reduced Oct4 expression directs a robust pluripotent state with distinct signaling activity and increased enhancer occupancy by Oct4 and Nanog. **12**, 531-545 (2013).
7. W.-W. Tee, S. S. Shen, O. Oksuz, V. Narendra, D. J. C. Reinberg, ERK activity promotes chromatin features and RNAPII phosphorylation at developmental promoters in mouse ESCs. **156**, 678-690 (2014).
8. C. Ma *et al.*, Nono, a bivalent domain factor, regulates Erk signaling and mouse embryonic stem cell pluripotency. **17**, 997-1007 (2016).
