# Supplementary material for: A NANOG‐pERK reciprocal regulatory circuit regulates Nanog autoregulation and ERK signaling dynamics
Source: EMBO Rep. 2022 Sep 6;23(11):e54421. doi: 10.15252/embr.202154421 (PMC9638859; doi:10.15252/embr.202154421)
Supplement: Supplementary file 10 [file EMBR-23-e54421-s002.pdf]

EMBO reports

A NANOG-pERK reciprocal regulatory circuit regulates Nanog autoregulation and ERK signaling dynamics

Hanuman Kale, Rajendra Rajpurohit, Debabrata Jana, Vishnu Vijay, Mansi Srivastava, Preeti Mourya, Gunda Srinivas, and P Chandra Shekar
DOI: 10.15252/embr.202154421

Corresponding author(s): P Chandra Shekar (csp@ccmb.res.in)

Review Timeline:

Submission Date:	1st Dec 21
Editorial Decision:	11th Jan 22
Revision Received:	30th May 22
Editorial Decision:	21st Jun 22
Revision Received:	9th Jul 22
Editorial Decision:	22nd Jul 22
Revision Received:	9th Aug 22
Accepted:	17th Aug 22

Editor: Achim Breiling

Transaction Report:

(Note: With the exception of the correction of typographical or spelling errors that could be a source of ambiguity, letters and reports are not edited. Depending on transfer agreements, referee reports obtained elsewhere may or may not be included in this compilation. Referee reports are anonymous unless the Referee chooses to sign their reports.)

Dear Dr. Shekar,

Thank you for the submission of your research manuscript to EMBO reports. We have now received the full set of referee reports that are copied below.

As you will see, the referees feel that the study is of interest (although referee #3 indicates novelty issues). However, all three referees have also several major concerns, indicating that the many effects reported are rather subtle and that the mechanism proposed has not been rigorously established. Moreover, they note technical and experimental shortcomings and also pointed to missing controls, discrepancies between datasets or doubts about reagent/assay specificity that question further the validity the data. As the reports are below, I will not further detail them here.

Given these comments and considering the amount of work required to address them, we cannot offer to publish your manuscript. However, in case you feel that you can address the referee concerns in a timely and thorough manner and can obtain data that would considerably strengthen the study (as outlined in the reports), we would have no objections to consider a new manuscript on the same topic in the future.

Please note that if you were to send a new manuscript this would be assessed again with respect to the literature and the novelty of your findings at the time of resubmission.

Yours sincerely,

Achim Breiling
Senior Editor
EMBO Reports

Referee #1:

Kale and colleagues address the question how negative autoregulation of Nanog expression pluripotent cells is mediated mechanistically. They show that Fgf4, Fgfbp1 and Fgfr2 expression is positively regulated by NANOG. Furthermore, they provide evidence that negative autoregulation of Nanog expression in inducible cell lines carrying Nanog-reporter constructs is dependent on intact Fgf4, Fgfbp1 and Fgfr2 genes. Together, these findings lead them to propose the existence of a cell-extrinsic negative feedback circuit for Nanog autoregulation. The authors provide further evidence for this loop by substantiating a previously identified mechanism of Nanog repression by ERK through NONO, and by following the dynamics of ERK and NANOG expression in sorted cell populations. In addition, the paper reports some interesting observations on ERK activation by the GSK3 inhibitor Chiron (Fig. 1), which in my view however do not contribute significantly to the main message of the paper.

The authors use an extensive set of appropriately validated transgenic and mutant cell lines to come up with a novel mechanism for an important question in the stem cell field - how the expression of pluripotency factors is controlled to enable both maintenance of pluripotency as well as differentiation. While these ingredients make the paper a potential candidate for publication, I note that the magnitude of some of the effects reported here is comparatively low, raising the question how relevant the author's new mechanism is in comparison to previously proposed mechanisms. I also have issues with some of the experimental modalities that the authors use. These shortcomings could be addressed by a set of additional experiments, as well as by toning down some of the conclusions.

Major points
1. Fig. 2B: The magnitude of the effects reported here is very small. This experiment needs to be repeated to ascertain reproducibility and convey a clearer message about this magnitude.

2. Fig. 2E and following: The authors need to state more clearly in which medium these experiments were carried out. This is not obvious from the current description in the text or figure legends, and impinges on the interpretation of the results.

3. Fig. 3B, Fig. EV1A: The positive regulation of Fgf4, Fgfbp1 and Fgfr2 and the resulting upregulation of pERK is one of the most important parts of the new mechanism proposed by the authors. However, I feel that to establish this link beyond doubt, the paper would benefit from additional data that can be generated with existing reagents, as well as from presenting the current data differently.
For example, why is the qPCR data in EV1A? I think this should be in the main figure. The same is true for the pERK blot in Fig. EV1E - the increase in pERK upon induction of NANOG provides good evidence for the author's model and should also be included in the main figure. Furthermore, a strong prediction is that this increase in pERK should be absent in Fgf4, Fgfbp1 and Fgfr2 mutant lines, something that the authors can easily test with the existing cell lines.
In contrast, I am skeptical of the correlation between NANOG and FGFR2 expression inferred from the FACS analysis in Fig. 3B. This kind of assay is prone to artifacts. For example, can the axes be compared between the four panels? If yes, why does

this analysis not show the increase in NANOG expression in SLPD compared to SL? For the analysis shown in Fig. 3C, have the authors controlled for confounding variables, such as cell size?
A more convincing way to establish the dependence between NANOG, FGFR and pERK expression would be to use the NANOG inducible cell lines that the authors have, and to blot for FGFR and pERK after different induction times (I notice that the authors have working antibodies for both FGF receptors). Adding these additional experiments would put more emphasis on the papers main message and significantly strengthen the work.

4. Page 4: "... and discounts that direct role of NANOG..." This statement is too strong and should be reworded. The cell-extrinsic mechanism could well work in addition to the previously described cell-intrinsic ones.

5. Page 4: "...suggests that ZFP281 is dispensable..." I disagree with this statement, particularly because EV1K shows higher NANOG reporter expression in Zfp281 mutant cells compared to the wild type. If I a missing something here please clarify.

6. Fig. 5A, B: This data should be shown at higher resolution, using a smaller field of view. As it is presented right now, it does not support the authors point. Furthermore, the authors need to describe how the data in 5B have been generated from the data in 5A. Have they segmented the cells, and if so, how? How did they assign cytoplasmic pERK to nuclear NANOG?

7. Fig. 5D: Are these measurements derived from FACS data? As pointed out above, using flow cytometry to make these measurements is prone to artifacts. I recommend the authors repeat this experiment, but use western blotting to measure NANOG and pERK levels. This is an doable experiment that could substantiate the link between NANOG and pERK dynamcis.

Minor points
1. Fig. 1G: ppERK levels in SL2i seem to be similar to those in SLPD, in contrast to the proposed role of the GSK3 inhibitor in activating MEK. Can the authors comment on this observation?

2. Fig. EV1L: The different conditions are difficult to distinguish in this plot. Consider a different representation.

3. Discussion section: "...specify primitive endoderm by cell-autonomous mechanisms ..." should read. "...non-cell-autonomous..."

4. Discussion section: The authors could more clearly spell out the possibility that NANOG heterogeneity is a consequence of de-synchronized cycling of cells through the NANOG-pERK feedback loop. They can also cite experiments with sorted Nanog-reporter cells (e.g. PMID: 19582141).

5. Molotkov et al. (2017, PMID: 28552557) have demonstrated a central role for FGFR1 and not FGFR2 for pluripotentcy and differentiation. The authors need to discuss their results in the light of these previous findings.

6. Fig. S6: The NANOG staining looks blurred. Please provide higher resolution / smaller field of view representations of this data.

Overall I found the paper hard to review. Page and line numbers as well as a clearer labelling of the figures would have been helpful here. It would also have been helpful to include journal names in the reference list.

Referee #2:

In this manuscript, Kale and colleagues present evidence of a Nanog-pERK reciprocal regulatory circuit. The focus of the paper is on Nanog autorepression, Nanog induction of components of the FGF/MEK/ERK pathway, MEK/ERK regulation of Nanog transcript and really nice analysis of Nanog/pERK dynamics. The manuscript presents some interesting findings and would be of interest to the community. The main issue in my view is the focus on the MEK/ERK having a role in Nanog AUTOrepression rather than just 'repression'. There are a number of observations that I struggle to see how they fit the narrative presented in the paper, but that hopefully the authors can clarify.

Major points:
- "[...] (Figure 2A, Appendix Fig S2C and D). The data from distinct induction systems conclusively establish and essential role of MEK1/2 in Nanog autoregulation". I struggle to interpret these results because the phenotypes associated with Nanog expression on a null-background are very subtle compared to the effect of adding PD. To put it in another way, if we look at figure 2A, the median GFP level in conditions that contain PD ~3x higher than SL alone in the absence of OHT. Since TNERT cells -OHT are Nanog-null, it suggests whatever PD is doing to the Nanog locus is independent of Nanog. Addition of Nanog seems to have a very minor effect. Similar to above, deleting elements NBR1 and NBR2 also changes the expression of Nanog:GFP independently of Nanog expression (Figure 3F).

- "Nanog induces and enhances FGF autocrine signalling through FGFR2 to execute Nanog autoregulation" Can the authors

also confirm that mutants of Figure 2D-E show altered levels of pERK? Do these mutants in -OHT conditions also show elevated Nanog:GFP expression compared to control lines, similar to addition of PD? Similarly, to justify the previous statements, the authors would need to show that Nanog induction in the mutant lines does not alter ERK levels.

- Could the authors please explain some inconsistency of results? In figure EV1E, +OHT addition causes very high levels of pERK. However, in figure 4C, +OHT has no effect of pERK levels. The ability of Nanog expression to induce pERK is a central aspect so it's a bit worrying to see this discrepancy.
- on a related note, it is also unclear what is happening in the Nono-null cells. These cells overall show much lower levels of Nanog:GFP compared to untargeted controls (Figrue Apendix 5C). Since Figure 4C shows pERK is nearly absent in Nono-null cells, I would expect these cells phenocopy to some extent what happens in PD conditions and show much higher Nanog:GFP.

- Nono and pERK occupancy on Nanog locus. How does FGF and PD treatment affect Nono expression? The changes in pERK binding at Nanog locus are unsurprising given that pERK levels are dictated by the culture treatment. Is the same for Nono? This is important since the authors claim that "pERK is essential for the recruitment of NONO to the Nanog locus".

- all chip-seq and chip-PCR data of the paper. Can the authors please also include a control region for a known/validated target of pERK and Nono to show that the levels of binding at the Nanog locus are biologically relevant? Since pERK levels vary greatly with the treatment, and the binding does not appear enriched in any particular region, it would help to show that the chip signal observed are not just the result of higher background.

- Interpretation of the Nono/pERK/Pol2 binding results. Also here I find the interpretation a bit confusing. The authors say "pERK-NONO affects POL2 loading onto the Nanog locus preventing the initiation of transcription" but there is no evidence that the process is at all dependent on Nono. If this was the case, the Nono-null cells would have higher Nanog:GFP expression, but the opposite appears to be the case.

Other points:
- introduction: The introduction is very short, and readers would benefit from key topics being included. For example, there is no mention of the literature on the role of ERK; 2iLIF culture is not introduced but CH and PD inhibitors are used regularly; and no description of provided as to what NONO is. The literature is also not always accurately cited (e.g. {Faddah:2013ed, Filipczyk:2013kp} convincingly disproved the earlier report of {Miyanari:2012ef} about moni-allelic regulation of Nanog).

- First results section (Figure 1). The authors refer to any changes in Nanog in terms of the hypothesised autorepression ("Nanog expression was derepressed above basal levels", "Nanog expression in SPDL indicates inefficient repression", etc). This is not a very helpful description, since the authors present very little evidence at this point that autorepression is involved. I suggest describing the results without assigning a mechanism (e.g. "results in increased expression".

- Please provide some information on the levels, heterogeneity and timing of induction for both TNERT and inducible Nanog lines.

- Figure 2D-E. Can this be confirmed through an alternative method? Maybe RT-qPCR on Nanog UTR, etc? again, the phenotypes are very subtle.

- please indicate clearly for all figures what the statistics are.

- Beginning of result section 2: lots of acronyms are given to different lines, and the inserts within lines (e.g. NANOGER vs TNERT, etc). This creates more confusion than necessary. I suggest consolidating the nomenclature.

- please provide some info on the figure as to what Figure 2C shows

- in the literature it's more common to find non cell-autonomous than "cell non-autonomous" as the authors describe.

Referee #3:

Kale et al explore mechanisms underpinning Nanog autoregulation in ES cells using a series of inducible reporters and cell lines. They conclude that Nanog regulates expression of FGF signalling components such as FGFBP1 and FGFR2 to control FGF-mediated ERK1/2 activation, which in turn leads to high levels of ERK activity in cells with high Nanog expression. This leads to recruitment of NONO to the Nanog locus, which suppresses PolII loading and Nanog transcription to establish autoregulation.

In this area, it is well established that ERK1/2 signalling drives Nanog heterogeneity in ES cells, although this is not discussed or referenced at all in the introduction. Therefore, it does not take much of a leap to consider that because ERK1/2 signalling drives Nanog heterogeneity, it might also underpin Nanog autoregulation. As a result, in my view the conceptual advance presented

here is somewhat limited.

Furthermore, Nanog has been previously proposed to modulate FGF signalling, but by a different mechanism in which Nanog drives expression of FGF4 and suppression of FGFR2 in Nanog high cells. This enables segregation of Nanog high and low populations based on distinct FGF-ERK1/2 signalling responses (reviewed in Lanner and Rossant 2010). However, this study proposes the opposite, that Nanog expression leads to ERK1/2 activation in Nanog high cells. However, from what I can see, the data presented do not fully support this - in Fig5D Nanog and pERK1/2 staining show an inverse correlation, which is opposite to what the authors are proposing. My concern is that in such a dynamic system, it is difficult to confidently distinguish signalling and transcriptional responses in distinct populations of Nanog low and Nanog high cells. Thus, there is a risk that what is being reported here simply reflects what is already known - i.e. that Nanog is regulated by and regulates ERK1/2 signalling.

The area that may be of most interest and novelty is the mechanistic interplay between ERK1/2 signalling and Nanog at the transcriptional level. Is pERK1/2 staining also heterogeneous in regular mouse ES cells cultured in LIF/FCS, which exhibit dynamic Nanog heterogeneity? This would provide evidence that ERK1/2 signalling is not simply influenced by the distinct domed morphology observed in Nanog high EDiN colonies. And is pERK1/2 staining in Fig 5A specific? This can be controlled using MEK1/2 inhibitor PD0325901. Finally, is pERK1/2 recruitment measured by ChIP in Fig 4G specific and inducible - what are the controls for non-specific background signal?

** As a service to authors, EMBO Press provides authors with the ability to transfer a manuscript that one journal cannot offer to publish to another journal, without the author having to upload the manuscript data again. To transfer your manuscript to another EMBO Press journal using this service, please click on
Link Not Available

Referee #1:

Kale and colleagues address the question how negative autoregulation of Nanog expression pluripotent cells is mediated mechanistically. They show that Fgf4, Fgfbp1 and Fgfr2 expression is positively regulated by NANOG. Furthermore, they provide evidence that negative autoregulation of Nanog expression in inducible cell lines carrying Nanog-reporter constructs is dependent on intact Fgf4, Fgfbp1 and Fgfr2 genes. Together, these findings lead them to propose the existence of a cell-extrinsic negative feedback circuit for Nanog autoregulation. The authors provide further evidence for this loop by substantiating a previously identified mechanism of Nanog repression by ERK through NONO, and by following the dynamics of ERK and NANOG expression in sorted cell populations. In addition, the paper reports some interesting observations on ERK activation by the GSK3 inhibitor Chiron (Fig. 1), which in my view however do not contribute significantly to the main message of the paper.

The authors use an extensive set of appropriately validated transgenic and mutant cell lines to come up with a novel mechanism for an important question in the stem cell field - how the expression of pluripotency factors is controlled to enable both maintenance of pluripotency as well as differentiation. While these ingredients make the paper a potential candidate for publication, I note that the magnitude of some of the effects reported here is comparatively low, raising the question how relevant the author's new mechanism is in comparison to previously proposed mechanisms. I also have issues with some of the experimental modalities that the authors use. These shortcomings could be addressed by a set of additional experiments, as well as by toning down some of the conclusions.

We thank the referee for reading our manuscript thoroughly and appreciating the novel mechanisms for an important question elucidated in the manuscript. We have improved the manuscript by performing multiple experiments based on the constructive comments of the referees.

We thank the referee for pointing out our interesting observations on ERK activation by the GSK3 inhibitor Chiron (Fig 1). We do realize, that we have not been able to convey clearly, how the observations in Fig 1 led to the main work of this manuscript? We apologize for an inadvertent editing error. We had deleted introductory sentences describing, how Fig 1 and other published data prompted us to investigate a possible role of FGF autocrine signaling in *Nanog* autorepression.

Navarro et. al. had shown that the *Nanog* autorepression is functional in 2iL (Navarro *et al*, 2012). Also suggested that *Nanog* autoregulation is independent of MEK/GSK signaling. In Fig1 we show that MEK activity is not completely and continuously inhibited in 2iL (in presence of CHIR) unlike in SLPD. The presence of residual MEK activity in 2iL from Fig1 and the functioning of *Nanog* autorepression in 2iL together opened a possibility

for the role of MEK signaling in *Nanog* autorepression in contrast to a previous report (Navarro *et al.*, 2012).

We have added these introductory sentences in line # 98 " *Nanog autorepression was shown to function in 2iL (Navarro et al., 2012).* We *show residual MEK activity persist in 2iL and Nanog expression is lower in 2iL than SLPD.*" This will put the observations in Fig 1 in the context of the main work and suggest these observations as the basis for investigating the possible role of FGF autocrine signaling in *Nanog* autorepression.

Major points

1. Fig. 2B: The magnitude of the effects reported here is very small. This experiment needs to be repeated to ascertain reproducibility and convey a clearer message about this magnitude.

We have again repeated the experiments at least 4 times. We find the results are consistently reproducible. Please find the data from multiple experiments for your consideration (given below). *Nanog*:GFP is repressed in TNERT+OHT, addition of FGFR inhibitor - SU5402 inhibits *Nanog* autoregulation, suggesting FGFRs are essential for *Nanog* autoregulation. We would like to bring forth an important observation about the magnitude of the effects of our old and new experiments. We have found that the magnitude of the effect is more pronounced when early passage cells of TNERT or TDiN clones are used. The magnitude of the effects tends to become smaller beyond 15 passages. The older data was obtained from late passage cells. The new experiments were carried out using early passage cells of the same clones. We have replaced the previous representative images of Fig. 2B with representative images from the new experiments. The corresponding quantification data in Fig EV2E is also replaced.

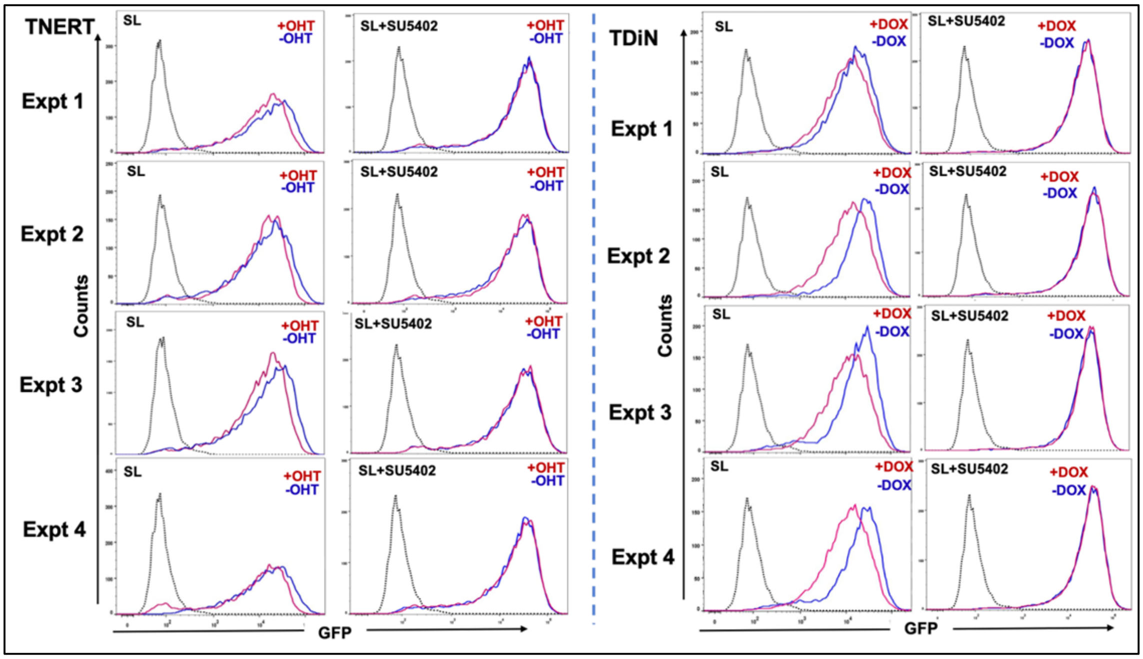

2. Fig. 2E and the following: The authors need to state more clearly in which medium these experiments were carried out. This is not obvious from the current description in the text or figure legends, and impinges on the interpretation of the results.

Thanks for pointing out this oversight. The experiments in Fig. 2E were carried out in SL media. We have added the media conditions into the panels of Fig. 2E to facilitate the interpretation of the results. The culture condition is now described in the figure legend as well.

3. (i) Fig. 3B, Fig. EV1A: The positive regulation of Fgf4, Fgfbp1 and Fgfr2 and the resulting upregulation of pERK is one of the most important parts of the new mechanism proposed by the authors. However, I feel that to establish this link beyond doubt, the paper would benefit from additional data that can be generated with existing reagents, as well as from presenting the current data differently.

We agree additional data and a better representation of the current data can strengthen the proposed mechanism. We have performed additional experiments and also improved the representation of the data to strengthen this part of the manuscript. Since multiple issues are raised in this part of the comments. We have split this part of referee comments into subsections to address all the issues comprehensively.

(ii) For example, why is the qPCR data in EV1A? I think this should be in the main figure. The same is true for the pERK blot in Fig. EV1E - the increase in pERK upon induction of NANOG provides good evidence for the author's model and should also be included in the main figure.

Thanks for this suggestion, now we have included Fig EV1 among the main figures. It is now included as main Fig 3 in the revised manuscript. Fig 3, 4, and 5 are henceforth referred to as Fig 4, 5, and 6 respectively in the revised manuscript.

(iii) Furthermore, a strong prediction is that this increase in pERK should be absent in Fgf4, Fgfbp1 and Fgfr2 mutant lines, something that the authors can easily test with the existing cell lines.

We have performed western blot analysis of pERK and ERK in the mutant lines. We could still detect pERK in all the mutant cell lines. However, the pERK levels were significantly lower in all mutant lines relative to the TNERT line. Further, the treatment with OHT did not significantly induce pERK levels in these mutant cell lines. We did not observe significant changes in pERK levels in the Fgfr1-/- mutant line relative to TNERT.

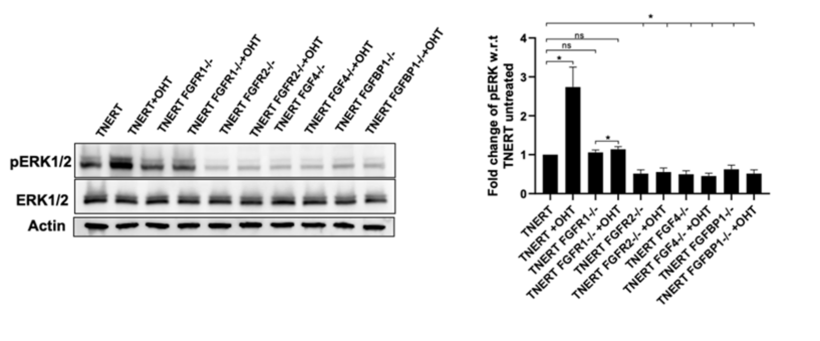

(iv) In contrast, I am skeptical of the correlation between NANOG and FGFR2 expression inferred from the FACS analysis in Fig. 3B. This kind of assay is prone to artifacts. For example, can the axes be compared between the four panels? If yes, why does this analysis not show the increase in NANOG expression in SLPD compared to SL?

We acknowledge the concerns and skepticism of the referees. We have performed experiments suggested by the referees and repeated the FACS analysis experiments with a new set of antibodies and acquisition voltage settings to strengthen the claims.

The axes of the four panels cannot be compared. Although, the NANOG antibody is common for all four panels. The FGFR1 and FGFR2 antibodies can have different affinities for their antigens. Hence, the fluorescence intensities cannot be compared between FGFFR1 and FGFR2 samples. Further, the acquisition voltages for FACS were set based on control cells, stained with primary antibody isotype control and secondary fluorescent antibody. The background fluorescence for the FGFR1 and FGFR2 will be different for these as well. However, within FGFR1 or FGFR2 stained samples, the axes can be compared for SL and SLPD samples.

We agree with the referee that the NANOG expression is expected to show an increase in SLPD. We performed the quantifications for the median fluorescence of NANOG in SL and SLPD samples of FGFR1 and FGFR2 panels. We find a significant increase in NANOG expression in SLPD samples relative to SL in both FGFR1 and FGFR2 stained samples (on the right). However, the difference is not as drastic as seen in the TbC44Cre6 or TNERT cell lines. This could be due to two reasons.

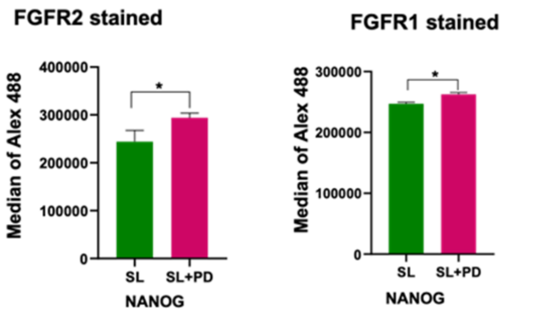

Estimation of Median Alexa488 from previous data which was acquire at high voltage setting and different set of antibodies

(a) The TβC44Cre6 or TNERT have internal fluorescence of GFP from the *Nanog* locus, whereas in this experiment the fluorescence measures the endogenous NANOG protein by immunofluorescence.

(b) Further, most of the data points in the FACS data are seen between 10^5 and 10^6 decades, which is at the end of the scale in the graph. This was due to the high voltage setting during

FACS data acquisition. Although, this would not significantly affect the calculation of the correlation coefficient. It does not represent the data points beyond 10^6 decades, hence, can impact the estimation of expression levels. So, we have repeated the experiments with lower voltage settings to reliably estimate both the correlation coefficient and the expression levels.

The antibodies and double staining can sometimes be a source of artifacts in FACS. To minimize the possibilities of artifacts, we have performed additional FACS experiments using a different set of primary antibodies against the antigens. We have acquired the data by setting the acquisition voltages with cells stained with primary antibody isotype control and secondary fluorescent antibody. All the data points in the FACS are within the 10^5 decade scale. We have analyzed the correlation coefficient as well as the expression levels of NANOG, FGFR1, and FGFR2 (on the right). The expression is significantly increased in SLPD. The data has been replaced with the new data from these experiments in Fig. 4B (Fig. 3B of the previous version of the manuscript).

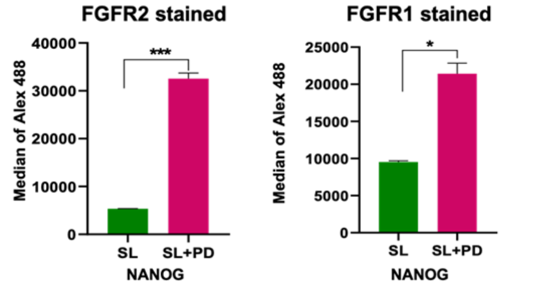

Estimation of Median NANOG stained wit ALex488 for the data presented in Fig. 4B acquired at lower voltage with different set of antibodies

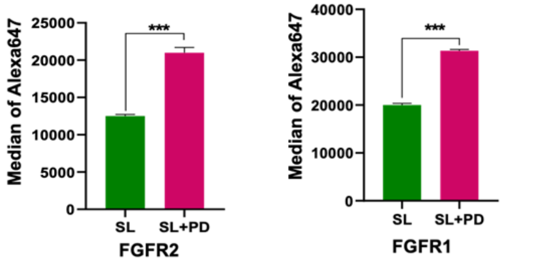

Estimation of Median FGFR2 and FGFR1 stained wit Alex647 for the data presented in Fig. 4B acquired at lower voltage with different set of antibodies

For the analysis shown in Fig. 3C, have the authors controlled for confounding variables, such as cell size?

Yes, the confounding variable like cell size had been controlled by plotting FSC/SSC and gating a narrow range of FSC. Even the cell doublets had been excluded by plotting the FSC height vs FSC area.

A more convincing way to establish the dependence between NANOG, FGFR and pERK expression would be to use the NANOG inducible cell lines that the authors have, and to blot for FGFR and pERK after different induction times (I notice that the authors have working antibodies for both FGF receptors). Adding these additional experiments would put more emphasis on the papers main message and significantly strengthen the work.

We cannot agree more with the referees. We have performed western blot of FGFRs and pERK in the TNERT cell line. The OHT treatment can induce translocation of NANOG into the nucleus in 5-10 minutes. We have analyzed the expression of FGFRs and pERK at one-hour time intervals after OHT treatment. FGFRs were induced in OHT treated samples which increased gradually over time. The induction was more pronounced in FGFR2. The pERK levels also increase gradually over time, suggesting NANOG induces FGFRs and

pERK in ESCs. The data is now added to the main figure as Fig 3E. We have also made appropriate edits to the text of the manuscript.

4. Page 4: "... and discounts that direct role of NANOG..." This statement is too strong and should be reworded. The cell-extrinsic mechanism could well work in addition to the previously described cell-intrinsic ones.

We have reworded the statement to “suggesting that autoregulation predominately operates via non-cell autonomous mechanisms besides the mechanism proposed earlier (Fidalgo *et al*, 2012).”

5. Page 4: "...suggests that ZFP281 is dispensable..." I disagree with this statement, particularly because EV1K shows higher NANOG reporter expression in Zfp281 mutant cells compared to the wild type. If I a missing something here please clarify.

We apologize for creating confusion. This could be due to improper wording used by us to describe this result. Now we have reworded the statement to convey the meaning of the result.

We confer with observations of the referees that *Nanog* reporter expression in the TNERT Zfp281 mutant line is higher than in TNERT cells. This suggests that Zfp281 is essential for the repression of expression in agreement with a previous report (Fidalgo *et al*, 2011).

The induction of NANOG in the TNERTZfp281 mutant line with OHT was able to repress the Nanog:GFP (similar to TNERT + OHT) suggesting that the *Nanog* autorepression is still functional in TNERTZfp281-/- cells. If ZFP281 was essential for autoregulation; OHT induction in TNERTZfp281-/- should not have repressed Nanog:GFP. The results should have resembled the TNERT FGF4-/- or FGFR2-/- cells. So our results show that ZFP281 is essential for *Nanog* repression, but does not contribute to *Nanog* autorepression.

6. Fig. 5A, B: This data should be shown at higher resolution, using a smaller field of view. As it is presented right now, it does not support the authors point. Furthermore, the authors need to describe how the data in 5B have been generated from the data in 5A. Have they segmented the cells, and if so, how? How did they assign cytoplasmic pERK to nuclear NANOG?

We have repeated the experiments and acquired high-resolution images by confocal microscopy. We have replaced the low-resolution images with higher resolution images.

For immunostaining, along with NANOG and pERK antibodies, we have utilized the E-Cadherin antibody to mark the boundaries of the cells. The cells in the colonies were segmented manually using E-Cadherin or phalloidin staining in imageJ software. The Mean

fluorescence intensity (MFI) was estimated within the marked boundary in imageJ software. MFI from around 200 cells and correlation plots were plotted.

7. Fig. 5D: Are these measurements derived from FACS data? As pointed out above, using flow cytometry to make these measurements is prone to artifacts. I recommend the authors repeat this experiment, but use western blotting to measure NANOG and pERK levels. This is an doable experiment that could substantiate the link between NANOG and pERK dynamcis.

Allaying the skepticism of the referees with possible artifacts from FACS measurements for this experiment, we have repeated the experiments. We have analyzed the NANOG and pERK expression and correlation using confocal microscopy and western blots. In previous FACS experiments, we had cultured the cells in fresh media after sorting. This would remove the impact of the factors (FGF4 and FGFBP1) secreted by the cells into the media. This would affect the pERK levels in the sorted cells. Hence, an inverse correlation was observed between NANOG and pERK in previous FACS experiments in Fig. 5C, D, as pointed out by referee #3. To circumvent this possibility, in the new experiments, we collected the media from the cells before cell sorting and filtered it through 0.22 um filters. The same media was used for culturing the sorted cells. Microscopy and western blot analyses were performed on cells at 4 hr time intervals till 16 hrs. we observe a correlation between the pERK and NANOG western blot and immunostaining. The previous FACS has been replaced with the new one in Fig. 6C, D and the results section has been modified accordingly.

Minor points

1. Fig. 1G: ppERK levels in SL2i seem to be similar to those in SLPD, in contrast to the proposed role of the GSK3 inhibitor in activating MEK. Can the authors comment on this observation?

This is an interesting and keen observation made by the referee. Multiple experiments in our lab which do not directly pertain to the main work of this manuscript have shown that dynamics and levels of pERK in SL2i are intermediate to SLPD and 2iL.

The MEK activity in 2iL increase over a period of time and by 8 hours a significant increase in MEK activity can be observed. In SL2i the MEK activity increase is not as much as 2iL after 24 hrs (Fig EV1C).

The serum contains growth factors, cytokines, and hormones like BMP4, FGF ligands, Insulin, etc. these factors may activate MEK and enhance pERK. In serum-free media like N2B27 (used in 2iL) these factors are missing, hence we expect consistent repression of MEK activity. However, our observations of an increase in MEK activity in 2iL are paradoxical to this logic. We do not have a definitive answer to this paradox. We think some confounding factors present in the serum might be counteracting the role of the GSK3 inhibitor role in activating MEK. These factors are absent in serum-free (2iL) media resulting in a significant increase in MEK activity in 2iL.

2. Fig. EV1L: The different conditions are difficult to distinguish in this plot. Consider a different representation.

We have changed the colors of the plots. All conditioned media where Nanog:GFP is not repressed (TNERT+OHT 0hrs, TNERTFgf4-/-, and TbC44Cre6) are plotted in shades of blue. The conditioned media where Nanog:GFP is repressed (TNERT+OHT 48hrs, E14Tg2aFgf4 OE, and 50ngFgf4) are plotted in shades of maroon. Changes have been made in the description of the results to facilitate the interpretation of the data. We hope it would improve the clarity of these results. Now, this is Fig 3L in the revised manuscript.

3. Discussion section: "...specify primitive endoderm by cell-autonomous mechanisms ..." should read. "...non-cell-autonomous..."

Thank you very much for pointing out this error, we have edited the text to insert the correction.

4. Discussion section: The authors could more clearly spell out the possibility that NANOG heterogeneity is a consequence of de-synchronized cycling of cells through the NANOG-pERK feedback loop. They can also cite experiments with sorted Nanog-reporter cells (e.g. PMID: 19582141).

Thanks for the suggestion, we added this possibility into the discussion section.

5. Molotkov et al. (2017, PMID: 28552557) have demonstrated a central role for FGFR1 and not FGFR2 for pluripotentcy and differentiation. The authors need to discuss their results in the light of these previous findings.

We have addressed this aspect in the discussion section.

6. Fig. S6: The NANOG staining looks blurred. Please provide higher resolution / smaller field of view representations of this data.

We have replaced the images with higher resolution and a larger field of view in Appendix Figure S1. We have added high-resolution smaller field of view images to Fig 6E.

Overall I found the paper hard to review. Page and line numbers as well as a clearer labelling of the figures would have been helpful here. It would also have been helpful to include journal names in the reference list.

We apologize for the inconvenience. We have added the page numbers, and line numbers improved the labelling of the figures and reformatted the reference list to include the journal names. We hope the revised manuscript reads better and clearer.

Referee #2:

In this manuscript, Kale and colleagues present evidence of a Nanog-pERK reciprocal regulatory circuit. The focus of the paper is on Nanog autorepression, Nanog induction of components of the FGF/MEK/ERK pathway, MEK/ERK regulation of Nanog transcript and really nice analysis of Nanog/pERK dynamics. The manuscript presents some interesting findings and would be of interest to the community. The main issue in my view is the focus on the MEK/ERK having a role in Nanog AUTOrepression rather than just 'repression'. There are a number of observations that I struggle to see how they fit the narrative presented in the paper, but that hopefully the authors can clarify.

We thank the referee for considering the findings interesting and of interest to the community. We relate to the importance of the issue raised by the referees about "the focus on MEK/ERK having a role in Nanog AUTOrepression rather than just 'repression' . Thanks for highlighting the clear distinction between the *Nanog* AUTOrepression and just "repression".

MEK/ERK and FGF4's role in Nanog repression is well established. Similarly, TCF3, GATA6, GATA4, TCF15, OTX2, ZFP281, SNAI1, and AMPK also have been reported to repress Nanog expression (Chae & Broxmeyer, 2009; Davies *et al*, 2013; Di Giovannantonio *et al*, 2021; Fidalgo *et al.*, 2011; Galvagni & Neri, 2015; Pereira *et al*, 2006; Schröter *et al*, 2015; Singh *et al*, 2007). These molecules may be induced in different contexts and by different mechanisms leading to the repression of *Nanog*. Whereas, *Nanog* autorepression is a distinct context where increased levels of NANOG, induce a negative feedback regulatory mechanism to limit *Nanog* expression levels within those cells. It seemed logical to us, to the hypothesis that *Nanog* might activate and recruit one or more of the repressive mechanisms for its feedback autorepression, rather than evolve a distinct repressive mechanism for autorepression. Among these repressive mechanisms, ERK signaling was found to be essential for autorepression in our initial experiments. We investigated the possible role of other repressive mechanisms in auto repression -TCF15 (Fig EV5A), ZFP281 (Fig 3K), and TCF3 (given below). Although these factors are essential for *Nanog* repression they do not seem to be critical for *Nanog* autorepression. Along with the essential role of ERK signaling in *Nanog* repression, our work suggests that ERK signaling is activated by *Nanog* to execute its own feedback autorepression.

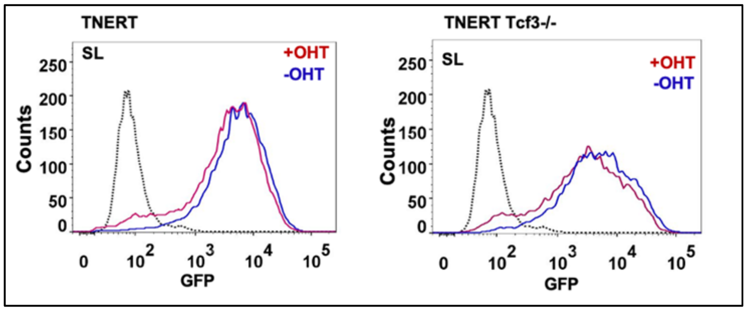

The essential role of ERK signaling in Nanog repression is demonstrated by earlier reports, here we have focused predominately on the role of ERK signaling in *Nanog* AUTOrepression. We have performed additional experiments and analyses to clarify all the comments of the referees.

Major points:

- "[...] (Figure 2A, Appendix Fig S2C and D). The data from distinct induction systems conclusively establish and essential role of MEK1/2 in Nanog autoregulation". I struggle to interpret these results because the phenotypes associated with Nanog expression on a null-background are very subtle compared to the effect of adding PD. To put it in another way, if we look at figure 2A, the median GFP level in conditions that contain PD ~3x higher than SL alone in the absence of OHT. Since TNERT cells -OHT are Nanog-null, it suggests whatever PD is doing to the Nanog locus is independent of Nanog. Addition of Nanog seems to have a very minor effect. Similar to above, deleting elements NBR1 and NBR2 also changes the expression of Nanog:GFP independently of Nanog expression (Figure 3F).

We agree with the observation of the referees that the median GFP level in SLPD is multiple folds higher relative to SL in TNERT in Fig. 2A. We also agree with the conclusion drawn by the referees that "it suggests whatever PD is doing to the Nanog locus is independent of Nanog". This conclusion is true when comparing partial data, about only -OHT samples of SL and SLPD. This conclusion is incomplete and pertains to repression (just repression) of *Nanog*, but not autorepression. FGF, INSULIN, and other ligands present in the serum can activate basal levels of ERK signaling in TNERT cultured in SL. These moderate ERK levels can induce repression of the Nanog locus as seen by the lower median GFP levels in TNERT in SL relative to SLPD. This suggests that *Nanog* repression (just repression) is independent of NANOG but dependent on ERK activation mediated by factors. Hence the median GFP levels in SL are multiple folds less than SLPD where ERK signaling is completely abrogated. When NANOG is induced in TNERT by adding OHT (TNERT+OHT) the Nanog locus is further repressed. This repression is due to induction of *Nanog* in the cells and hence this decrease in median GFP levels relative to -OHT represents *Nanog* Autorepression. However, in the presence of PD where pERK is absent, OHT induction of NANOG fails to repress the *Nanog* locus and the media GFP levels remain unchanged. Repression is dependent on ERK signaling, Autorepression is dependent on both Nanog and ERK signaling. These data suggest that ERK is essential for just repression and as well as autorepression of *Nanog*.

Addition of Nanog seems to have a very minor effect.

We also agree that the addition of *Nanog* seems to have a minor effect on *Nanog* repression compared to the difference seen between the SL and SLPD. However, this difference is significant and reproducible. Multiple serum factors in SL, feed into ERK signaling continuously to repress *Nanog*. This results in a consistent and high magnitude of

repression of *Nanog* in SL relative to SLPD. The addition of Nanog in SL enhances ERK signaling to repress Nanog further. Nanog autorepression is one more layer of repression that acts in addition to the regular repression. This notion is also shared by referee #1. The reasons for the minor effect could be (a) The base level of Nanog expression used for comparison of Nanog autorepression is already too low in TNERT-OHT cultured in SL. It might not be appropriate to compare the difference between SL and SLPD using SLPD as the base level; with +OHT and -OHT in SL using -OHT as the base level. These two contexts are different - one is repression the other is autorepression. (b) Nanog autorepression is a feedback repression mechanism to reduce the expression levels of Nanog in the cell when it exceeds a threshold limit. Logically it should limit the expression of Nanog only to the extent of the excessive expression beyond the threshold limit. This is also clear from our data (figure EV4 A-C), where mere expression of Nanog does not induce autorepression, but it is induced only when Nanog is overexpressed. The magnitude of the difference in repression also appears to be dependent on the over-expression levels of Nanog. (c) Further, the TNERT cell line is similar to the Nanog autorepression experimental system (NERTC3) used by Navarro et al. The magnitude of Nanog autorepression observed by us is similar to and consistent with the data of Navarro et al. 2012.

Similar to above, deleting elements NBR1 and NBR2 also changes the expression of Nanog:GFP independently of Nanog expression (Figure 3F).

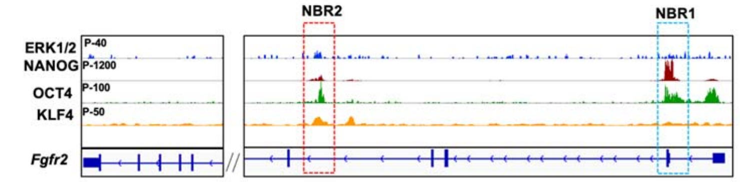

Browser track of transcription factors enrichment on NBR1 And NBR2 sites of *Fgfr2*

The NBR1 and NBR2 regions encompass consensus sequences for NANOG binding as well as for multiple other factors (Browser track of CHIP data given below). NANOG is one of the many factors that binds to these regions and activate FGFR2. Deletion of NBR1 and NBR2 is likely to change the expression of *Nanog*:GFP independent of Nanog. Our data do not suggest/claim that NBR1 and NBR2 are exclusively dependent on Nanog expression to change *Nanog*:GFP. Instead, our data suggest that NBR2 is essential for NANOG to repress *Nanog*:GFP. The possibility of NBR1 and NBR2 regions being regulated by other factors leading to Nanog repression in a different context cannot be ruled out.

- "Nanog induces and enhances FGF autocrine signaling through FGFR2 to execute Nanog autoregulation" Can the authors also confirm that mutants of Figure 2D-E show altered levels of pERK? Do these mutants in -OHT conditions also show elevated Nanog:GFP expression compared to control lines, similar to addition of PD? Similarly, to justify the previous statements, the authors would need to show that Nanog induction in the mutant lines does not alter ERK levels.

We have analyzed the pERK levels in all mutant lines- Fgfr2-/-, Fgfbp1-/-, Fgf4-/-, and Fgfr1-/-. We observed an overall reduction in the pERK levels in all mutants relative to TNERT. Fgfr1-/- did not show very significant changes in the pERK levels. Further, the

induction of Nanog by the addition of OHT did not significantly enhance the pERK activity in these mutants. The data is provided in part 3 (iii) of the response to Referee #1.

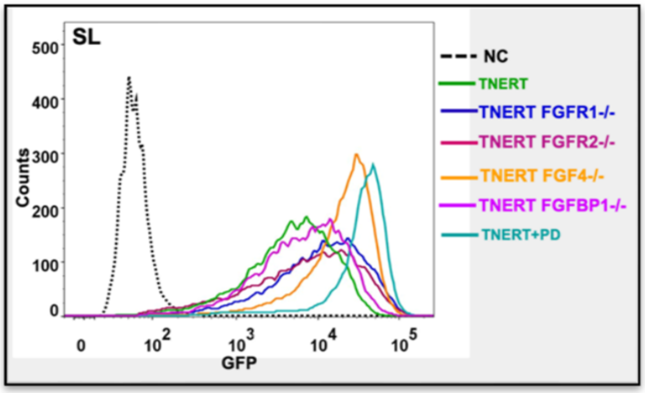

We have performed experiments to compare the expression of Nanog:GFP in -OHT condition of all the mutants with the control TNERT line. We do observe an increase in the Nanog:GFP expression in all the mutant lines. The magnitude of increase in Nanog:GFP is different in all mutant lines. The highest increase was seen in the ligand mutant (Fgf4-/-) which was relatively close to PD. None of the mutants showed Nanog:GFP expression equivalent to PD treatment (on the right). This suggests pERK might also be activated by other growth factor pathways to some extent in ESCs.

- Could the authors please explain some inconsistency of results? In figure EV1E, +OHT addition causes very high levels of pERK. However, in figure 4C, +OHT has no effect of pERK levels. The ability of Nanog expression to induce pERK is a central aspect so it's a bit worrying to see this discrepancy.

We thank the referees for pointing out seemingly inconsistent results. The increase in pERK levels in +OHT is not obvious in the representative western blot images due to a smudge in the TNERT+OHT samples in Fig 4C (now Fig. 5C). However, The western blot quantification data corresponding to Fig 4C which was provided in Fig. S5D clearly showed a significant increase in pERK levels from four independent experiments. We are sorry for the confusion arising due to poor quality representative blots in Fig 4C. We have replaced the blots with another set of representative blots without smudges to represent the results clearly in the revised manuscript in Fig 5C (previously Fig 4C). The quantification data remains the same in Fig EV5D (previous Fig. S5D)

- on a related note, it is also unclear what is happening in the Nono-null cells. These cells overall show much lower levels of Nanog:GFP compared to untargeted controls (Figrue Apendix 5C). Since Figure 4C shows pERK is nearly absent in Nono-null cells, I would expect these cells phenocopy to some extent what happens in PD conditions and show much higher Nanog:GFP.

We were also puzzled by the lower levels of Nanog:GFP in TNERTNono-/- cells relative to TNERT. We have analyzed multiple independent clones of TNERTNono-/-. The Nanog:GFP level was found to be low in all the clones. We do not understand, why the Nanog:GFP levels are low in TNERTNono-/- cells contrary to the expected increase in GFP expression? At this juncture, we can only speculate on possible reasons. TNERTNono-/- lines carry null mutations of both *Nanog* and *Nono* genes. The anomalous behavior of Nanog:GFP could be a result of epistatic interactions between these two mutations. We do not have any evidence to confirm this. Alternately, we have deleted the *Nono* gene in E14Tg2a ESCs to understand its

effect on Nanog expression without interference with any other mutations. We have performed western blot and immunostaining to assess the impact of the loss of NONO on pERK and NANOG. The Nono-/- cells showed low levels of PERK and enhanced expression of *Nanog* as expected, suggesting that Nono is essential for sustaining pERK levels and repression of *Nanog* (data given below). The anomalous behavior of *Nanog*:GFP in TNERTNono-/- cells is interesting but does not in any way impact the central findings and conclusions of the manuscript. We have not included any discussion about this aspect in the manuscript.

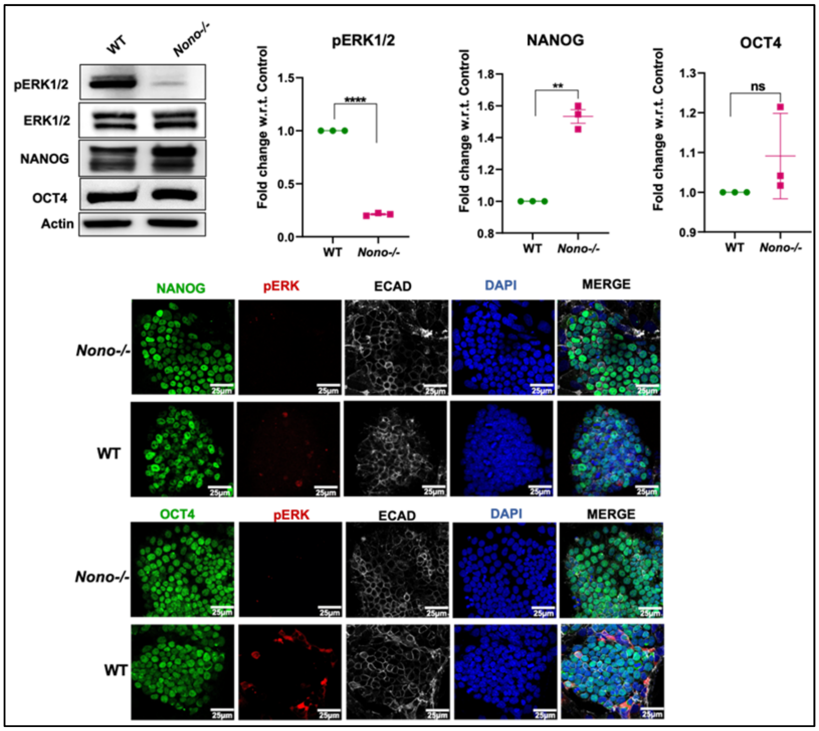

- Nono and pERK occupancy on Nanog locus. How does FGF and PD treatment affect Nono expression? The changes in pERK binding at Nanog locus are unsurprising given that pERK levels are dictated by the culture treatment. Is the same for Nono? This is important since the authors claim that "pERK is essential

for the recruitment of NONO to the Nanog locus".

- We have performed additional western blot analysis for NONO expression in FGF4 and PD treatment. The result shows that the expression of NONO correlates with pERK expression. The NONO expression was increased in FGF4 and reduced in PD treatment. The related data is added to Fig 5E.
- Yes, the changes in NONO binding on the *Nanog* locus are mostly similar to pERK. The occupancy of NONO was generally increased in FGF4 and reduced in PD treatment. This data was already provided in Fig 4H (Fig 5H in the revised manuscript) of the manuscript.

- all chip-seq and chip-PCR data of the paper. Can the authors please also include a control region for a known/validated target of pERK and Nono to show that the levels of binding at the Nanog locus are biologically relevant? Since pERK levels vary greatly with the treatment, and the binding does not appear enriched in any particular region, it would help to show that the chip signal observed are not just the result of higher background.

We agree with the referees. We had already analyzed the enrichment of pERK, NONO, H3K27, H3K4, and Pol2 in known validated targets (positive control) and a non-genic region (negative control). Now, we provided this data in Fig EV5G.

- Interpretation of the Nono/pERK/Pol2 binding results. Also here I find the interpretation a bit confusing. The authors say "pERK-NONO affects POL2 loading onto the Nanog locus preventing the initiation of transcription" but there is no evidence that the process is at all dependent on Nono. If this was the case, the Nono-null cells would have higher Nanog:GFPexpression, but the opposite appears to be the case.

We had provided evidence for the dependence of this process on pERK in Fig 4I (Now Fig 5I). We have carried out ChIP-qPCR to evaluate POL2 occupancy on the Nanog locus in *Nono*-/- ESCs (*the Nono-/- cell line* is *described in the above section on the previous page)*. The occupancy of POL2 on the *Nanog* locus was significantly increased in the *Nono*-/- cell relative to wildtype. The data is now added in Fig EV5F. Our data shows that either loss of pERK or NONO can affect the other, eventually affecting POL2 loading onto the *Nanog* locus.

Other points:
- introduction: The introduction is very short, and readers would benefit from key topics being included. For example, there is no mention of the literature on the role of ERK; 2iLIF culture is not introduced but CH and PD inhibitors are used regularly; and no description of provided as to what NONO is. The literature is

also not always accurately cited (e.g. {Faddah:2013ed, Filipczyk:2013kp} convincingly disproved the earlier report of {Miyanari:2012ef} about moni-allelic regulation of Nanog).

Thanks for the suggestions, we have made changes in the introduction section to address these points.

- First results section (Figure 1). The authors refer to any changes in Nanog in terms of the hypothesised autorepression ("Nanog expression was derepressed above basal levels", "Nanog expression in SPDL indicates inefficient repression", etc). This is not a very helpful description, since the authors present very little evidence at this point that autorepression is involved. I suggest describing the results without assigning a mechanism (e.g. "results in increased expression".

Our intention of using the terms "derepressed" and "repression" in the first results section was in the context of the well-known *Nanog* repression phenomenon and not the hypothesized autorepression. We agree with the referee that describing the result without ascribing to any mechanisms would be appropriate at this stage of the manuscript. We have made necessary edits accordingly in the manuscript text.

- Please provide some information on the levels, heterogeneity, and timing of induction for both TNERT and inducible Nanog lines.

- The TNERT and TDiN cell lines clones with a similar or higher expression level of Nanog compared to the WT ESC were used in this study. The clones which expressed lower levels of Nanog did not show Nanog autorepression and they were not used for any experiments.
- None of these clones showed heterogeneity of NANOG expression.
- The timing of induction for TNERT and inducible Nanog lines was already described in the Methods section – "The cells were cultured as described earlier (Festuccia *et al*, 2012). 4-Hydroxytamoxifen (4-OHT), Doxycycline, and Cycloheximide were used at a concentration of 1 µg/ml, 1 µg/ml, and 100 µg/ml respectively. The TNERT and its derivative cell lines were treated with 4-OHT for 18 hrs except when indicated. TDiN and EDiN were treated with Doxycycline for 48 hrs unless indicated."

- Figure 2D-E. Can this be confirmed through an alternative method? Maybe RT-qPCR on Nanog UTR, etc? again, the phenotypes are very subtle.

We have performed RT-qPCR analysis to assess Nanog:GFP transcript using a primer set corresponding to the 5'UTR region of Nanog mRNA. A significant reduction was observed in transcript levels after OHT treatment in TNERT and TNERTFgfr1-/- cells. No significant change was observed in *Fgfr2, Fgf4*, and *Fgfbp1* mutant lines upon OHT treatment. It is tempting to cross-compare the magnitude of differences between the FACS and qPCR data. However, in FACS, we are analyzing the GFP levels which will depend on the protein stability, and in qPCR, we are comparing the transcript which would follow entirely different dynamics. However, both the data suggest that the Nanog locus is repressed in TNERT and TNERTFgfr1-/- cells upon induction of NANOG by OHT. The data is now presented in Fig 2F. We have also analyzed the Nanog:GFP in all the mutants relative to TNERT, we observe that the transcript levels are increased in all the mutants relative to TNERT (data given on the right).

- please indicate clearly for all figures what the statistics are.

The detailed statistics information was provided in the methods section. Now we have also included it in all the figure legends.

- Beginning of result section 2: lots of acronyms are given to different lines, and the inserts within lines (e.g. NANOGER vs TNERT, etc). This creates more confusion than necessary. I suggest consolidating the nomenclature.

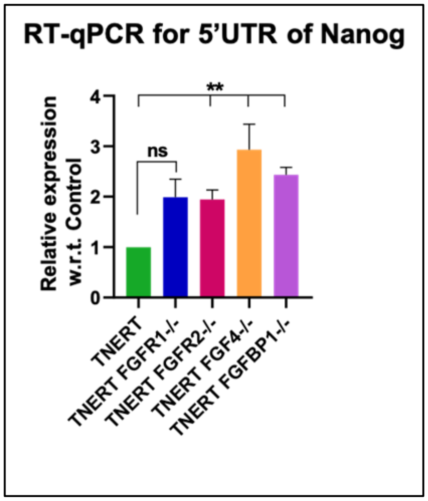

We have cut down on the acronyms for the inserts within cell lines to reduce the acronyms and limited the nomenclature to the names of different cell lines.

- please provide some info on the figure as to what Figure 2C shows

We have described more detailed information about Figure 2C in the result section.

- in the literature it's more common to find non cell-autonomous than "cell non-autonomous" as the authors describe.

Thanks for pointing out this error. We have edited the text appropriately as suggested.

Referee #3:

Kale et al explore mechanisms underpinning Nanog autoregulation in ES cells using a series of inducible reporters and cell lines. They conclude that Nanog regulates expression of FGF signalling components such as FGFBP1 and FGFR2 to control FGF-mediated ERK1/2 activation, which in turn leads to high levels of ERK activity in cells with high Nanog expression. This leads to recruitment of NONO to the Nanog locus, which suppresses PolII loading and Nanog transcription to establish autoregulation.

In this area, it is well established that ERK1/2 signalling drives Nanog heterogeneity in ES cells, although this is not discussed or referenced at all in the introduction. Therefore, it does not take much of a leap to consider that because ERK1/2 signalling drives Nanog heterogeneity, it might also underpin Nanog autoregulation. As a result, in my view the conceptual advance presented here is somewhat limited.

We thank the referee for the critical review and suggestions which helped us to improve the quality of the manuscript. We have addressed all the comments of the referee. We beg to differ with the view of the referee about the conceptual advance presented in this manuscript. We would like to present our case with more information and our point of view to persuade the referee to reconsider their opinion on the conceptual advances presented in this manuscript.

- We have discussed and referenced the essential function of ERK1/2 signalling in *Nanog* heterogeneity in the relevant context in the results and discussion section. We agree it would have been more appropriate to discuss and reference it in the introduction. Now, we have included the discussion of the function of ERK1/2 signalling in *Nanog* heterogeneity in the introduction of the revised manuscript.
- The referee's suggestion "that because ERK1/2 signalling drives Nanog heterogeneity, it might also underpin Nanog autoregulation" sounds like an obvious possibility. However, there is no literature that suggests anywhere in this direction. The available literature indicates the contrary.
  (a) Among the two studies which reported Nanog autorepression (Fidalgo et al. 2012 and Navarro et al. 2012), Navarro et al. suggested that ERK signaling is dispensable for Nanog autorepression. Navarro et al. state "Overall, these results suggest that Nanog autorepression persists in '2i + LIF' and is therefore independent of the MEK/GSK3 signaling pathways."
  (b) ERK signaling is essential for heterogeneous expression of pluripotency factors Rex1, KLf4, and Nanog (Marks *et al*, 2012; Wray *et al*, 2010). If ERK signalling can underpin Nanog autoregulation then it might also underpin autorepression of Rex1 and Klf4. However, there is no report of the autorepression phenomenon of Rex1 or Klf4 in ESCs. Klf4 autorepression is reported in breast cancer cells by an entirely different mechanism involving miRNA (Lin *et al*, 2011).

Furthermore, Nanog has been previously proposed to modulate FGF signalling, but by a different mechanism in which Nanog drives expression of FGF4 and

suppression of FGFR2 in Nanog high cells. This enables segregation of Nanog high and low populations based on distinct FGF-ERK1/2 signalling responses (reviewed in Lanner and Rossant 2010). However, this study proposes the opposite, that Nanog expression leads to ERK1/2 activation in Nanog high cells.

We have carefully studied Lanner and Rossant's review of 2010. We did not find any information suggesting Nanog drives expression of FGF4 and suppression of FGFR2. On the contrary, they have proposed a model (Fig 3, Lanner and Rossant 2010) where FGF/ERK is suggested to function upstream of Nanog and Gata6. They cite Guo et al. 2010 -"detected a stronger and earlier inverse correlation between *Fgf4* and *Fgfr2* transcripts in ICM cells than between *Nanog* and *Gata6*, supporting a model in which FGF/Erk signaling variation is upstream of Nanog and Gata6 (Fig. 3)" to support their model. This review does not describe Nanog high and Nanog low cells, they describe Nanog and Gata6 expressing cells in the context of segregation of ICM cells into primitive endoderm and epiblast. We do not think Nanog low cells can be equated to Gata6 positive cells in ICM. Cells expressing Gata6 would have mostly committed to primitive endoderm, whereas most of the Nanog low cells can rise to Nanog high cells (Chambers *et al*, 2007).

We agree with the referee that Nanog was shown to function upstream of FGF4. But, this was reported later by Frankenberg et al. in 2011(Frankenberg *et al*, 2011). We have not been able to find any report suggesting *Nanog* suppresses Fgfr2, so we would like to politely disagree with the referee on this part of the comment. Our data shows that Fgfr2 is induced by Nanog and Fgfr2 is essential for autorepression. Although Nanog is shown to be essential for Fgf4 induction, it is a secreted protein. It cannot distinguish between Nanog high and low cells. Whereas FGFR2 is induced by Nanog and retained on the membrane of Nanog high cells. This can help the FGF4 ligand to distinguish between the Nanog high and low cells and induce stronger ERK signalling in Nanog high cells. This can lead to induction of Nang autorepression predominately in the Nanog high cells. Our data (Fig 4A) and the logic of Nanog autorepression proposed by Fidalgo et al. and Navarro et al.(Fidalgo *et al.*, 2012; Navarro *et al.*, 2012) suggest that Nanog autorepression operates in Nanog high cells to prevent Nanog levels from crossing levels which can inhibit differentiation of ESC.

However, from what I can see, the data presented do not fully support this - in Fig5D Nanog and pERK1/2 staining show an inverse correlation, which is opposite to what the authors are proposing. My concern is that in such a dynamic system, it is difficult to confidently distinguish signalling and transcriptional responses in distinct populations of Nanog low and Nanog high cells. Thus, there is a risk that what is being reported here simply reflects what is already known - i.e. that Nanog is regulated by and regulates ERK1/2 signalling.

We agree with the referee, that the data presented by us in Fig 5D showed an inverse correlation between Nanog and pERK. This may not necessarily mean the opposite of what we are proposing. Since Nanog induces the Fgfr, Fgf4, and Fgfbp1 to activate pERK. There will be a time lag between the highest level of Nanog and the highest levels of pERK, with intermediate levels. However, this may not generate a complete inverse correlation as observed in Fig 5D.

After careful examination of the data and the experimental design, we recognized a flaw in our experimental design. We apologize for this mistake. Nanog autorepression functions only in Nanog high population. So, we sorted the top 10% Nanog high cells and cultured them to follow the dynamics of NANOG and pERK during the course of culture. In our experiments, we used fresh media to culture the cells. This was a mistake, by using fresh media we have effectively removed the impact of the factors secreted from the cells on the dynamics of NANOG and pERK. The right experiment would have been to culture the cells in the media collected from the cells before sorting Nanog high cells. This would retain all the secreted factors and allow further progression of the trajectory of NANOG and pERK. We think the culture of cells in fresh media removed the induction of FGF signaling by the secreted proteins and le dot reduction in pERK resulting in an inverse correlation with NANOG.

We have repeated the experiments considering all these factors with changes in the experimental design. The media from the cells was collected before cell sorting and filtered through a 0.22 µM filter. This media was used for culturing the cells to ensure the autocrine signaling factors secreted by the cells were retained. We analyzed NANOG and pERK expression every 4 hr intervals. Referee #1 had expressed reservation about FACS artifacts. Hence, we performed immunofluorescence and western blot analysis of these cells. Both data show a positive correlation between NANOG and pERK. They follow a similar pattern over a period of 16 hrs, with high expression at 8 hrs and low expression by 16 hrs. This behavior of NANOG and pERK expression is consistent with our suggestion that NANOG and pERK feed into each other and cycle through different intermediate levels of NANOG and pERK in culture.

The area that may be of most interest and novelty is the mechanistic interplay between ERK1/2 signalling and Nanog at the transcriptional level. Is pERK1/2 staining also heterogeneous in regular mouse ES cells cultured in LIF/FCS, which exhibit dynamic Nanog heterogeneity? This would provide evidence that ERK1/2 signalling is not simply influenced by the distinct domed morphology observed in Nanog high EDiN colonies. And is pERK1/2 staining in Fig 5A specific? This can be controlled using MEK1/2 inhibitor PD0325901. Finally, is pERK1/2 recruitment measured by ChIP in Fig 4G specific and inducible - what are the controls for non-specific background signals?

We have performed additional experiments to address all the questions.

- Yes, pERK1/2 was also heterogeneous in regular ESC cultured in SL, which exhibits dynamic Nanog heterogeneity. This data was already provided in Fig. 5 A. The results might not have been very clear due to the low resolution of the images. Now we have provided high resolution and high magnification images in Fig 6A, which clearly show heterogeneous expression of pERK.
- Yes, pERK staining is very specific. pERK can be seen as two puncta in mitotic cells (pERK has been shown to localize to spindle poles in mitosis (Shapiro *et al*, 1998). As suggested, we have also used PD treatment where pERK staining is completely lost (Fig 6A).
- Yes, the pERK1/2 recruitment measured by ChiP in Fig 4G (revised as Fig 5G) is specific and inducible. This is true for NONO as well. The inducibility can be seen in

the samples treated with FGF4 and PD in all the ChIP-qPCRs. We have analyzed the recruitment of pERK and other proteins shown in Fig 4G-K (revised a Fig 5G-K) to a non-genic region (negative control) and known validated target locus for each of these proteins. We do not observe significant enrichment of these proteins in the non-genic region but see significant enrichment in the known validated targets in the genome. The data is provided in Fig EV5G.

References:

Chae H-D, Broxmeyer HE, 2009. AMPK regulates Nanog Gene Expression Via p53 in Mouse Embryonic Stem Cells. American Society of Hematology.

Chambers I, Silva J, Colby D, Nichols J, Nijmeijer B, Robertson M, Vrana J, Jones K, Grotewold L, Smith A (2007) Nanog safeguards pluripotency and mediates germline development. *Nature* 450: 1230-1234

Davies OR, Lin C-Y, Radzisheuskaya A, Zhou X, Taube J, Blin G, Waterhouse A, Smith AJ, Lowell S (2013) Tcf15 primes pluripotent cells for differentiation. *Cell reports* 3: 472-484

Di Giovannantonio LG, Acampora D, Omodei D, Nigro V, Barba P, Barbieri E, Chambers I, Simeone A (2021) Direct repression of Nanog and Oct4 by OTX2 modulates the contribution of epiblast-derived cells to germline and somatic lineage. *Development* 148: dev199166

Festuccia N, Osorno R, Halbritter F, Karwacki-Neisius V, Navarro P, Colby D, Wong F, Yates A, Tomlinson SR, Chambers IJCsc (2012) Esrrb is a direct Nanog target gene that can substitute for Nanog function in pluripotent cells. 11: 477-490

Fidalgo M, Faiola F, Pereira C-F, Ding J, Saunders A, Gingold J, Schaniel C, Lemischka IR, Silva JC, Wang JJPotNAoS (2012) Zfp281 mediates Nanog autorepression through recruitment of the NuRD complex and inhibits somatic cell reprogramming. 109: 16202-16207

Fidalgo M, Shekar PC, Ang YS, Fujiwara Y, Orkin SH, Wang J (2011) Zfp281 functions as a transcriptional repressor for pluripotency of mouse embryonic stem cells. *Stem cells* 29: 1705-1716

Frankenberg S, Gerbe F, Bessonnard S, Belville C, Pouchin P, Bardot O, Chazaud C (2011) Primitive endoderm differentiates via a three-step mechanism involving Nanog and RTK signaling. *Developmental cell* 21: 1005-1013

Galvagni F, Neri F (2015) Snai1 represses Nanog to promote embryonic stem cell differentiation. *Genomics Data* 4: 82-83

Lin C-C, Liu L-Z, Addison JB, Wonderlin WF, Ivanov AV, Ruppert JM (2011) A KLF4–miRNA-206 autoregulatory feedback loop can promote or inhibit protein translation depending upon cell context. *Molecular and cellular biology* 31: 2513-2527

Marks H, Kalkan T, Menafra R, Denissov S, Jones K, Hofemeister H, Nichols J, Kranz A, Stewart AF, Smith A (2012) The transcriptional and epigenomic foundations of ground state pluripotency. *Cell* 149: 590-604

Navarro P, Festuccia N, Colby D, Gagliardi A, Mullin NP, Zhang W, Karwacki-Neisius V, Osorno R, Kelly D, Robertson M (2012) OCT4/SOX2-independent Nanog autorepression modulates heterogeneous Nanog gene expression in mouse ES cells. *The EMBO journal* 31: 4547-4562

Pereira L, Yi F, Merrill BJ (2006) Repression of Nanog gene transcription by Tcf3 limits embryonic stem cell self-renewal. *Molecular and cellular biology* 26: 7479-7491

Schröter C, Rué P, Mackenzie JP, Martinez Arias A (2015) FGF/MAPK signaling sets the switching threshold of a bistable circuit controlling cell fate decisions in embryonic stem cells. *Development* 142: 4205-4216

Shapiro PS, Vaisberg E, Hunt AJ, Tolwinski NS, Whalen AM, McIntosh JR, Ahn NG (1998) Activation of the MKK/ERK pathway during somatic cell mitosis: direct interactions of active ERK with kinetochores and regulation of the mitotic 3F3/2 phosphoantigen. *The Journal of cell biology* 142: 1533-1545

Singh AM, Hamazaki T, Hankowski KE, Terada N (2007) A heterogeneous expression pattern for Nanog in embryonic stem cells. *Stem cells* 25: 2534-2542

Wray J, Kalkan T, Smith AG (2010) The ground state of pluripotency. *Biochemical Society Transactions* 38: 1027-1032

1st Revision - Editorial Decision 21st Jun 2022

Dear Dr. Shekar

Thank you for the re-submission of your revised manuscript to our editorial offices. I have now received the reports from three of the referees that have re-evaluated, you will find below. As you will see, the two referees now fully support publication of your study in EMBO reports. Both have some final points and suggestions to improve the manuscript.

Referee #3 was more critical, indicating novelty issues and conflicts to previously published data. During cross-commenting with the other referees indicated that they disagree with this assessment. They state:

- 'Nanog-lo ESCs will almost never give rise to primitive endoderm in culture, in contrast to the situation in the ICM. I therefore think the authors are right to state that their mechanism operates in a different context, a context that could still be developmentally highly relevant.'

- 'The comparison between PE/EPI segregation is, in my opinion, not particularly helpful since a whole genetic program (that of the PE cells) that is involved in establishing the reciprocal relationship between FGF4 and FGFR2 observed in the embryo.'

- 'Moreover, the literature has proven that ESCs recapitulate a stage in development equivalent to the peri-implantation development, not the ICM. The Nanog low cells are not turning towards a primitive endoderm lineage. All evidence suggests these cells are differentiating further towards a post-implantation-like stage (pre-gastrulation). Therefore, it's more likely that the autoregulation described here is part of the circuit that allows the naïve pluripotency to collapse as the embryo implants.'

Considering these comments, I decided to proceed with the manuscript and to ask you to address the remaining points of referees #1 and #2 in a final revised manuscript. Please also provide a final point-by-point-response to the referee concerns, including your specific responses to referee #3.

Moreover, the manuscript needs formatting according to our journal style. Please carefully review the instructions that follow below.

PLEASE NOTE THAT upon resubmission revised manuscripts are subjected to an initial quality control prior to exposition to re-review. Upon failure in the initial quality control, the manuscripts are sent back to the authors, which may lead to delays. Frequent reasons for such a failure are the lack of the data availability section (please see below) and the presence of statistics based on n=2 (the authors are then asked to present scatter plots or provide more data points).

When submitting your final revised manuscript, we will require:

1) a .docx formatted version of the final manuscript text (including legends for main figures, EV figures and tables), but without the figures included. Figure legends should be compiled at the end of the manuscript text.

2) individual production quality figure files as .eps, .tif, .jpg (one file per figure), of main figures and EV figures. Please upload these as separate, individual files upon re-submission.

The Expanded View format, which will be displayed in the main HTML of the paper in a collapsible format, has replaced the Supplementary information. You can submit up to 5 images as Expanded View. Please follow the nomenclature Figure EV1, Figure EV2 etc. The figure legend for these should be included in the main manuscript document file in a section called Expanded View Figure Legends after the main Figure Legends section. Additional Supplementary material should be supplied as a single pdf file labeled Appendix. The Appendix should have page numbers and needs to include a table of content on the first page (with page numbers) and legends for all content. Please follow the nomenclature Appendix Figure Sx, Appendix Table Sx etc. throughout the text, and also label the figures and tables according to this nomenclature.

For more details, please refer to our guide to authors:
http://www.embopress.org/page/journal/14693178/authorguide#manuscriptpreparation

Please consult our guide for figure preparation:
http://wol-prod-cdn.literatumonline.com/pb-assets/embo-site/EMBOPress_Figure_Guidelines_061115-1561436025777.pdf

See also the guidelines for figure legend preparation:
https://www.embopress.org/page/journal/14693178/authorguide#figureformat

3) a complete author checklist, which you can download from our author guidelines (https://www.embopress.org/page/journal/14693178/authorguide). Please insert page numbers in the checklist to indicate where the requested information can be found in the manuscript. The completed author checklist will also be part of the RPF.

Please also follow our guidelines for the use of living organisms, and the respective reporting guidelines: http://www.embopress.org/page/journal/14693178/authorguide#livingorganisms

4) that primary datasets produced in this study (e.g. RNA-seq, ChIP-seq, structural and array data) are deposited in an appropriate public database. If no primary datasets have been deposited, please also state this in a dedicated section (e.g. 'No primary datasets have been generated and deposited'), see below.

See also: http://embor.embopress.org/authorguide#datadeposition

Please remember to provide a reviewer password if the datasets are not yet public.

The accession numbers and database should be listed in a formal "Data Availability" section (placed after Materials & Methods) that follows the model below. This is now mandatory (like the COI statement). Please note that the Data Availability Section is restricted to new primary data that are part of this study. This section is mandatory. As indicated above, if no primary datasets have been deposited, please state this in this section

Data availability

The datasets produced in this study are available in the following databases:

- RNA-Seq data: Gene Expression Omnibus GSE46843 (https://www.ncbi.nlm.nih.gov/geo/query/acc.cgi?acc=GSE46843)
- [data type]: [name of the resource] [accession number/identifier/doi] ([URL or identifiers.org/DATABASE:ACCESSION])

*** Note - All links should resolve to a page where the data can be accessed. ***

Moreover, I have these editorial requests:

6) As they are significantly cropped, please provide the source data for the Western blots shown in the manuscript (including the EV figures). The source data will be published in separate source data files online along with the accepted manuscript and will be linked to the relevant figures. Please submit scans of entire gels or blots together with the final revised manuscript. Please include size markers for scans of entire gels, label the scans with figure and panel number, and send one PDF file per figure (main and EV figures).

7) Our journal encourages inclusion of *data citations in the reference list* to directly cite datasets that were re-used and obtained from public databases. Data citations in the article text are distinct from normal bibliographical citations and should directly link to the database records from which the data can be accessed. In the main text, data citations are formatted as follows: "Data ref: Smith et al, 2001" or "Data ref: NCBI Sequence Read Archive PRJNA342805, 2017". In the Reference list, data citations must be labeled with "[DATASET]". A data reference must provide the database name, accession number/identifiers and a resolvable link to the landing page from which the data can be accessed at the end of the reference. Further instructions are available at: http://www.embopress.org/page/journal/14693178/authorguide#referencesformat

8) Regarding data quantification and statistics, please make sure that the number "n" for how many independent experiments were performed, their nature (biological versus technical replicates), the bars and error bars (e.g. SEM, SD) and the test used to calculate p-values is indicated in the respective figure legends (also for potential EV figures and all those in the final Appendix). Please also check that all the p-values are explained in the legend, and that these fit to those shown in the figure. Please provide statistical testing where applicable. Please add complete statistical testing to all diagrams (for main, EV figures). Please also indicate (e.g. with n.s.) if testing was performed, but the differences are not significant. Please avoid the phrase 'independent experiment', but clearly state if these were biological or technical replicates. See also: http://www.embopress.org/page/journal/14693178/authorguide#statisticalanalysis

9) Please also note our reference format: http://www.embopress.org/page/journal/14693178/authorguide#referencesformat

10) For microscopic images, please add scale bars of similar style and thickness to all the microscopic images (main, EV and Appendix figures), using clearly visible black or white bars (depending on the background). Please place these in the lower right corner of the images. Please do not write on or near the bars in the image but define the size in the respective figure legend.

11) We updated our journal's competing interests policy in January 2022 and request authors to consider both actual and perceived competing interests. Please review the policy https://www.embopress.org/competing-interests and update your competing interests if necessary. Please name this section 'Disclosure and Competing Interests Statement' and put it after the Acknowledgements section.

12) Please make sure that all the funding information is entered into the online submission system and is complete and similar to the one in the acknowledgements section of the manuscript text file.

13) The colour coding of panels A and B of Fig. 3 is not ideal, as it is difficult to distinguish adjacent bars. Please try to depict this differently.

14) Please make sure that the manuscript sections are ordered like this:
Title page - Abstract - Introduction - Results - Discussion - Materials and Methods - Data availability section (DAS) - Acknowledgements - Author contributions - Disclosure and Competing Interests Statement - References - Figure legends - Expanded View Figure legends

In addition, I would need from you:
- a short, two-sentence summary of the manuscript (not more than 35 words).
- three to four short one sentence bullet points highlighting the key findings of your study.
- a schematic summary figure (synopsis image) in jpeg or tiff format with the exact width of 550 pixels and a height of not more than 400 pixels that can be used as a visual synopsis on our website.

I look forward to seeing a revised version of your manuscript when it is ready. Please let me know if you have questions or comments regarding the revision.

Please use this link to submit your revision: https://embor.msubmit.net/cgi-bin/main.plex

Best,

Achim Breiling
Senior Editor
EMBO Reports

Referee #1:

In the revised version of their manuscript, the authors have added an extensive set of additional experiments that address a large number of my concerns raised in the first round of review. They have also re-organized some of the figures, and re-written the manuscript accordingly. Overall, the new analyses largely support their original claims. One exception is the data in the new Figure 6D, where, in contrast to the previous version where pERK and NANOG were anticorrelated, the authors now find a positive correlation. This discrepancy however does not necessarily invalidate the author's model, it rather indicates that the timescales over which the proposed regulatory circuit operates are still not fully understood.

I notice that the ChIP-qPCR data in Fig. 3C,D and 5 G-K is still lacking negative controls. These had been requested by reviewers #2 and #3, and I agree that these controls are important to distinguish specific binding to the respective loci from a mere increase in background due to higher levels of the respective proteins. If I've been missing something here please clarify.

Overall, the manuscript now makes a convincing case for the existence of a new type of negative feedback loop acting on Nanog expression that relies on the FGF signaling system, and that might complement previously proposed mechanisms for Nanog autorepression. In my opinion, the work now merits publication in EMBO Reports.

Referee #2:

In this revised manuscript the authors have answered some of the key concerns raised in the first round of reviewing. However, I would suggest that the western blot data of the ERK levels in the Fgf4, Fgfbp1 and Fgfr2 mutant lines {plus minus} OHT is an essential control and should be included in the paper. Similarly, the flow plots of the Nanog:GFP levels of the mutant lines in S/L should be included. This data is fundamental for showing that ERK activity is altered in the mutants.

Referee #3:

In the revised version, the authors have done some control experiments to improve the technical quality of the paper. My main concern remains that the overall conceptual advance is limited, although the authors provide several arguments as to why this work goes beyond the large amount of literature on interplay between Nanog and FGF-ERK1/2 signalling in both ES cells and the early embryo.

Most of the work in this area supports the notion that FGF-ERK1/2 signalling suppresses Nanog expression, and that Nanog promotes FGF4 expression to establish an amplification loop. Indeed, the authors concede that Nanog knockout embryos fail to

express FGF4 (Frankenberg et al, 2011), but suggest that Fgfr2 regulation has not been studied. However, Fgf4 and Fgfr2 exhibit reciprocal expression in the early embryo (Guo et al, 2010), reflecting Nanog hi/lo states. Therefore, the link between Nanog and FGF4/FGFR2 in the early embryo has been clearly established, and does not support the authors conclusions.

Another argument the authors present is that the context is different - either that this is specific to Nanog auto-regulation, or that Nanog hi/lo ES cells are not equivalent to the process of ICM segregating to Nanog hi EPI and Nanog lo PE in the embryo. Although strictly speaking Nanog heterogeneity represents a short window in embryonic development, ICM segregation to Nanog hi/lo cells nevertheless represents the developmental process most analagous to Nanog heterogeneity observed in ES cells. This raises a further concern that what is being reported here is specific to ES cells and is not relevant for development in vivo.

Referee #1:

In the revised version of their manuscript, the authors have added an extensive set of additional experiments that address a large number of my concerns raised in the first round of review. They have also re-organized some of the figures, and re-written the manuscript accordingly. Overall, the new analyses largely support their original claims. One exception is the data in the new Figure 6D, where, in contrast to the previous version where pERK and NANOG were anticorrelated, the authors now find a positive correlation. This discrepancy however does not necessarily invalidate the author's model, it rather indicates that the timescales over which the proposed regulatory circuit operates are still not fully understood.

Thanks for pointing out the discrepancy between the NANOG and pERK correlation between the previous version and the current version of Figure 6D. This difference is a result of the changes in the experimental design in the previous and current versions. We sorted the *Nanog*-high cells and analyzed the dynamics and correlation of NANOG and pERK over 16 hrs.

In the previous version after cell sorting, we cultured the cells in fresh media which eliminated the autocrine components secreted by the cells, which resulted in an anticorrelation between NANOG and pERK. We realized that to maintain the natural progression of NANOG and pERK dynamics, we should have retained the autocrine components secreted by the cells. To ensure this, in the current version, we cultured the cells in the same media collected from the cells before sorting. This ensured a natural progression of the NANOG and pERK dynamics, where we found a positive correlation between NANOG and pERK. In the light of the change in experimental design, both the results support the proposed model. As pointed out by the referee, these experiments are correlative and suggest that the timescales of operation of the regulatory circuit operate is still unknown.

I notice that the ChIP-qPCR data in Fig. 3C,D and 5 G-K is still lacking negative controls. These had been requested by reviewers #2 and #3, and I agree that these controls are important to distinguish specific binding to the respective loci from a mere increase in background due to higher levels of the respective proteins. If I've been missing something here please clarify.

We have added the ChIP-qPCR data of the non-genic region (negative control) in Fig. 3C, D. The ChIP-qPCR data of a known validated target locus (positive control) and non-genic region (negative control) for Fig. 5G-K have already been provided in Fig. EV5G.

Overall, the manuscript now makes a convincing case for the existence of a new type of negative feedback loop acting on Nanog expression that relies on the FGF signaling system, and that might complement previously proposed mechanisms

for Nanog autorepression. In my opinion, the work now merits publication in EMBO Reports.

Thank you very much for your time and constructive criticism for helping us to improve the quality of our work and the manuscript.

Referee #2:

In this revised manuscript the authors have answered some of the key concerns raised in the first round of reviewing. However, I would suggest that the western blot data of the ERK levels in the Fgf4, Fgfbp1 and Fgfr2 mutant lines {plus minus} OHT is an essential control and should be included in the paper. Similarly, the flow plots of the Nanog:GFP levels of the mutant lines in S/L should be included. This data is fundamental for showing that ERK activity is altered in the mutants.

We have included the flow plots and western data in Fig. 2 G & H respectively. Thank you very much for your time and constructive criticism to help us improve the quality of our work and manuscript.

Referee #3:

In the revised version, the authors have done some control experiments to improve the technical quality of the paper. My main concern remains that the overall conceptual advance is limited, although the authors provide several arguments as to why this work goes beyond the large amount of literature on interplay between Nanog and FGF-ERK1/2 signalling in both ES cells and the early embryo.

We thank the referee for their time and constructive criticism to help us improve the quality of our work and manuscript. We would like to clarify that we have performed all the experiments suggested by all the referees (including referee #3). We have generated new resources and performed additional experiments to further strengthen the findings presented in the manuscript.

We are sorry if the conceptual advances did not come through clearly in our manuscript or our response to referee #3's previous comments. Here, we again attempt to provide the salient conceptual advances of the manuscript.

- The major work in this manuscript is focused on delineating the mechanism of *Nanog* autoregulation. Contrary to the current understanding that ERK signaling is dispensable for *Nanog* autorepression, our data demonstrate that *Nanog* autoregulation operates through ERK signaling.

- The existing literature on *Nanog* autorepression provides some molecular mechanisms but still cannot explain the basic logic of autorepression- how autorepression is triggered and operated only in the *Nanog*-high cells in the ESC population. Here we show a new mechanism for *Nanog* autorepression, which elucidates how NANOG concentration-dependent induction of FGFR2 in *Nanog*-high cells sensitizes the cells for FGF signaling and induces *Nanog* autorepression exclusively in *Nanog*-high cells.
- Dynamic and heterogeneous expression of pERK has been reported in ESCs and embryos. The mechanisms that induce pERK heterogeneity in ESCs and embryos are not understood. Our data show that *Nanog* heterogeneity can feed into pERK heterogeneity and vice versa through the *Nanog* autoregulation mechanism.

Most of the work in this area supports the notion that FGF-ERK1/2 signalling suppresses Nanog expression, and that Nanog promotes FGF4 expression to establish an amplification loop. Indeed, the authors concede that Nanog knockout embryos fail to express FGF4 (Frankenberg et al, 2011), but suggest that Fgfr2 regulation has not been studied.

The fact that *Nanog* function is essential for FGF4 secretion was discussed and cited from our first version of the manuscript in the results and discussion sections as given below. We have also explained - why the *Fgf4* induction by *Nanog* cannot explain the logic of *Nanog* autoregulation, which prompted us to investigate FGFR further.

- "Cell non-autonomous function of *Nanog* in the induction of primitive endoderm(Frankenberg *et al*, 2011; Messerschmidt & Kemler, 2010) and essentiality of secreted proteins FGF4 and FGFBP1 in autoregulation prompted us to investigate cell non-autonomous mechanisms."
- "*Nanog* is considered to induce FGF paracrine signaling through FGF4 secretion and specify primitive endoderm by non-cell-autonomous mechanisms (Frankenberg *et al*, 2011; Messerschmidt & Kemler, 2010). Although FGF4 is essential for *Nanog* autoregulation, it is a secreted protein. Its induction by NANOG can neither explain the functioning of autoregulation exclusively in *Nanog*-high cells nor the heterogenous pERK activation…….".

However, Fgf4 and Fgfr2 exhibit reciprocal expression in the early embryo (Guo et al, 2010), reflecting Nanog hi/lo states. Therefore, the link between Nanog and FGF4/FGFR2 in the early embryo has been established, and does not support the authors conclusions.

We disagree with the comments of the referee. In our opinion, the Fgf4 and Fgfr2 inverse correlation reflecting Nanog hi/lo state in Guo et. al., 2010 may not be comparable to Nanog hi/lo ESCs. The link between *Nanog* and FGF4/FGFR2 established in early embryos may not be extrapolated to ESC for the following reasons.

- Guo et al. analyzed gene expression in single cells of 8C, 16C, 32C, and 64C stage embryos. The *Fgf4* and *Fgfr2* show inverse correlation in 32-cell and 64-cell ICM

in Fig. 5. In the same figure (Fig 5C &D) they clearly show that *Fgf4* correlates with *Nanog*; *Fgfr2* correlates with *Gata4* and *Gata6*. This is due to the segregation of the EPI and the PE. The *Nanog*-low cells at this stage express *Gata6 & Gata4* which have acquired PE fate, this fate choice is driven actively by a PE transcription network involving *Gata6*, *Gata4,* and *Sox17*. On the contrary, the *Nanog*-low cells in ESC culture, do not express *Gata6* or *Gata4* nor they are differentiated to PE. They retain pluripotency and can revert to *Nanog*-high states as shown by Chambers et al., 2007 and Kalmar et al., 2009.

- As suggested by other referees, it's well established in the literature that ESCs are equivalent to the pre-implantation epiblast cells of the embryos and not the ICM (Nichols *et al*, 2009). We think, comparing the links of *Nanog-Fgf4-Fgfr2* in 32-cell and 64-cell stage ICM to ESCs may not be relevant. In our opinion, it may be appropriate to compare ESCs to epiblast of later embryonic stages as described in the response to the below comments.

Another argument the authors present is that the context is different - either that this is specific to Nanog auto-regulation, or that Nanog hi/lo ES cells are not equivalent to the process of ICM segregating to Nanog hi EPI and Nanog lo PE in the embryo. Although strictly speaking Nanog heterogeneity represents a short window in embryonic development, ICM segregation to Nanog hi/lo cells nevertheless represents the developmental process most analagous to Nanog heterogeneity observed in ES cells. This raises a further concern that what is being reported here is specific to ES cells and is not relevant for development in vivo.

While we agree that *Nanog* heterogeneity represents a short window in embryonic development, we disagree with "ICM segregation to Nanog hi/lo cells nevertheless represents the developmental process most analagous to Nanog heterogeneity observed in ES cells".

ESCs are shown to be the cell culture counterparts of the pre-implantation epiblast (Nichols *et al.*, 2009). We suggest ESCs may be comparable to the Nanog hi/lo cells of the epiblast rather than ICM. Although *Nanog* expression is mostly homogenous in epiblast from E3.5 to E4.5, some of the cells lose *Nanog* expression and re-establish *Nanog* heterogeneity in the epiblast from E4.5-E4.7 as shown in Fig.1 of Acampora et al., 2016. Acampora et al., suggest that during the naïve-to-prime pluripotency transition, *Nanog* expression is heterogeneous in the epiblast and ESCs (Acampora *et al,* 2016, Acampora et al, 2017). The *Nanog* hi/lo cells in ESCs may be more analogous to the peri-implantation stage epiblast. Other referees have suggested that the *Nanog* autoregulation might be part of the circuit that allows exit from naïve pluripotency in the peri-implantation stages. In the light of this information, We suggest that the autoregulation mechanism and the reciprocal circuit being reported by us in ESCs is not an *in vitro* artifact, but a highly relevant mechanism for embryonic development, which opens up possibilities for further investigations.

References:

Frankenberg S, Gerbe F, Bessonnard S, Belville C, Pouchin P, Bardot O, Chazaud C (2011) Primitive endoderm differentiates via a three-step mechanism involving Nanog and RTK signaling. *Developmental cell* 21: 1005-1013

Messerschmidt DM, Kemler R (2010) Nanog is required for primitive endoderm formation through a non-non-cell-autonomoushanism. *Developmental biology* 344: 129-137

Nichols J, Silva J, Roode M, Smith A (2009) Suppression of Erk signsignalingmotes ground state pluripotency in the mouse embryo. *Development* 136: 3215-3222

Acampora D, Omodei D, Petrosino G, Garofalo A, Savarese M, Nigro V, et al. (2016) Loss of the Otx2-binding site in the Nanog promoter affects the integrity of embryonic stem cell subtypes and specification of inner cell mass-derived epiblast. *Cell reports* 15: 2651-2664

D. Acampora, L. G. Di Giovannantonio, A. Garofalo, V. Nigro, D. Omodei, A. Lombardi, et al. (2017) Functional antagonism between OTX2 and NANOG specifies a spectrum of heterogeneous identities in embryonic stem cells. *Stem cell reports* 9: 1642-1659

2nd Revision - Editorial Decision 22nd Jul 2022

Dear Dr. Shekar,

Thank you for the submission of your revised manuscript to our editorial offices. I went through this, and we are nearly done.

I have these final editorial requests I ask you to address in a further revised manuscript:

- Please reduce the number keywords on the title page to five.

- We now use CRediT to specify the contributions of each author in the journal submission system. CRediT replaces the author contribution section. Please use the free text box to provide more detailed descriptions. Thus, please remove the author contributions section from the manuscript text file. See also guide to authors: https://www.embopress.org/page/journal/14693178/authorguide#authorshipguidelines

- The synopsis image provided has too small fonts. Please provide this with bigger fonts but keeping the size (exact width of 550 pixels and a height of not more than 400 pixels).

- Please provide as separate related manuscript text file a short, two-sentence summary of the manuscript (not more than 40 words) and three to four short bullet points highlighting the key findings of your study (not more than 2 lines each).

- The dotted line in the FACS profiles shown (unstained control -?) is still not uniformly labelled. There should be a legend for this line in all the FACS profiles shown using the same symbol and name. Please define the name ('-ve c') in the respective figure legends. There are still profiles where the dotted line is not defined in the legend in the figure (2B, 3J, 4A, EV2C/D and EV4B), or where in the legend a full line is shown (2H and 3F). Moreover, in the legend other profiles shown a clear dotted line should be shown (resembling the line in the profile - 1A/B, 2A/E, 3K/L, 4C/F, 5B, EV3B and EV5A). Please check.

- Moreover, for some FAX profiles shown, this unstained control peak looks identical (e.g. Fig. 2B second and third panel, or 2B second and third panel - there are more examples in 2E, 4F and EV4B). Why is that? Aren't these different experiments and even the control should look individual for each experiment?

- Regarding data quantification and statistics, please add complete statistical testing to all diagrams (for main, EV figures). Please also indicate (e.g. with n.s.) if testing was performed, but the differences are not significant. There are still many diagrams with only partial testing. Please check.

- There are figures in the Appendix and Tables in the Appendix file with no name ('Mycoplasma Test', 'PCR control for genomic DNA', 'STR Analysis Report' and 'PSet- Primer set used for STR analysis'). Please name these as the other Appendix items (Appendix Figure Sx; Appendix Table Sx), add legends, mention these with these names in the TOC and add callouts for these in the manuscript text.

- Appendix Figure 1 has only one panel, thus it does not need the 'A' label. Please remove this and correct the callouts.

I look forward to seeing the final revised version of your manuscript when it is ready. Please let me know if you have questions regarding the revision.

Please use this link to submit your revision: https://embor.msubmit.net/cgi-bin/main.plex

Best,

Achim Breiling
Senior Editor
EMBO Reports

Dear Dr Breiling,

Thanks for your final editorial suggestions. We sincerely appreciate the due diligence and suggestion made to improve the quality of the manuscript. We have addressed all the points raised by you. We have also carefully looked at the manuscript and figures, made small changes to keep up the consistency and remove inadvertent errors, where-ever we could notice. Please find below details of the changes made and actions taken by us to address the comments.

Yours sincerely

Chandra

Response to the final editorial suggestions:

- Please reduce the number keywords on the title page to five.

> We have reduced the number of key words to five in the title page.

- We now use CRediT to specify the contributions of each author in the journal submission system. CRediT replaces the author contribution section. Please use the free text box to provide more detailed descriptions. Thus, please remove the author contributions section from the manuscript text file. See also guide to authors: https://www.embopress.org/page/journal/14693178/authorguide#authorshipguidelines

> We have specified the author contributions in the journal submission system as per CRediT. We have removed the author contributions section in the revised manuscript text file.

- The synopsis image provided has too small fonts. Please provide this with bigger fonts but keeping the size (exact width of 550 pixels and a height of not more than 400 pixels).

> We changed the text to bigger and bold fonts keeping the size of the synopsis image same.

- Please provide as separate related manuscript text file a short, two-sentence summary of the manuscript (not more than 40 words) and three to four short bullet points highlighting the key findings of your study (not more than 2 lines

each).

> We have provided a separate manuscript related file with two sentence summary and four bullet points highlighting key findings of our study.

- The dotted line in the FACS profiles shown (unstained control -?) is still not uniformly labelled. There should be a legend for this line in all the FACS profiles shown using the same symbol and name. Please define the name ('-ve c') in the respective figure legends. There are still profiles where the dotted line is not defined in the legend in the figure (2B, 3J, 4A, EV2C/D and EV4B), or where in the legend a full line is shown (2H and 3F). Moreover, in the legend other profiles shown a clear dotted line should be shown (resembling the line in the profile - 1A/B, 2A/E, 3K/L, 4C/F, 5B, EV3B and EV5A). Please check.

> We have added dotted lines in the FACS profiles figure panels of Figure 2B, 3J, 4A, EV2C, Ev2D and EV4B.
> We have described the '-ve c' in the all the respective figure legends. The name '-ve c' is described as unstained control in the legends of Fig1A,B; Fig 2A,B,E,H; Fig 3F,J,K,L; Fig 4A,C,F and Fig 5B. Also in the legends of EV figures - EV2C,D; Ev3B, Ev4B and EV5A.
> The full line for '-ve c' in 2H and 3F has been changed to dotted line consistent with other figures.
> We have labelled all the dotted lines in the FACS profile uniformly in all figure panels of the main figures and the EV figures.

- Moreover, for some FAX profiles shown, this unstained control peak looks identical (e.g. Fig. 2B second and third panel, or 2B second and third panel - there are more examples in 2E, 4F and EV4B). Why is that? Aren't these different experiments and even the control should look individual for each experiment?

> Yes, these are different experiments. We agree that the controls should look individual for each experiment.
> Although superficially, the FACS profiles of unstained control peaks in Fig2B (2nd and 3rd panel), Fig2E (3rd and 4th panel), Fig4F (1st and 3rd panel) and also Fig2A (2nd and 3rd panel) might appear identical, but they are not identical. The differences among these profiles can be noticed under higher magnification. Since these profiles were acquired around same time for each experiment, and the unstained controls only have autofluorescence, their FACS profile will appear nearly similar (they may not be identical). We understand that having similar FACS profiles of the unstained controls can lead to confusion about the experimental design.

Keeping this mind, we have replaced panels in Fig.2A (1st and 2nd panel), Fig.2B (2nd panel), Fig.2E (3rd panel) and Fig. 4F (3rd panel) with different set of representative images from other sets of replicate data.

> Incase of FigEV4B, there was inadvertent mistake. We had used same control in 1st and 2nd panel. We regret this oversight. We have now replaced the second panel in EV4B with FACS profile of a different replicate data.

- Regarding data quantification and statistics, please add complete statistical testing to all diagrams (for main, EV figures). Please also indicate (e.g. with n.s.) if testing was performed, but the differences are not significant. There are still many diagrams with only partial testing. Please check.

> We have added complete statistical testing to Fig. 1A,B,E & I; Fig. 3A-D,G & H; Fig. 4G-K; Fig. EV1H; Fig. EV3D-F & H-K; Fig. EV5 F & H. We have indicated n.s in the figure panels, where ever test was done and no difference were found.

- There are figures in the Appendix and Tables in the Appendix file with no name ('Mycoplasma Test', 'PCR control for genomic DNA', 'STR Analysis Report' and 'PSet- Primer set used for STR analysis'). Please name these as the other Appendix items (Appendix Figure Sx; Appendix Table Sx), add legends, mention these with these names in the TOC and add callouts for these in the manuscript text.

> The appendix and tables in the Appendix file are given names, the legends for each figure is provided. These are now indexed in the TOC with page numbers. Call outs for these are given in the manuscript text in the materials and method section in line number 412.

- Appendix Figure 1 has only one panel, thus it does not need the 'A' label. Please remove this and correct the callouts.

> We have removed ‘A’ label in the Appendix Figure 1 and also changed the call out in the manuscript text.

Dr. P Chandra Shekar
CSIR-Centre for Cellular and Molecular Biology
Uppal road
Habsiguda
Hyderabad, Telangana 500007
India

Dear Dr. Shekar,

I am very pleased to accept your manuscript for publication in the next available issue of EMBO reports. Thank you for your contribution to our journal.

At the end of this email I include important information about how to proceed. Please ensure that you take the time to read the information and complete and return the necessary forms to allow us to publish your manuscript as quickly as possible.

As part of the EMBO publication's Transparent Editorial Process, EMBO reports publishes online a Review Process File to accompany accepted manuscripts. As you are aware, this File will be published in conjunction with your paper and will include the referee reports, your point-by-point response and all pertinent correspondence relating to the manuscript.

If you do NOT want this File to be published, please inform the editorial office within 2 days, if you have not done so already, otherwise the File will be published by default [contact: emboreports@embo.org]. If you do opt out, the Review Process File link will point to the following statement: "No Review Process File is available with this article, as the authors have chosen not to make the review process public in this case."

Should you be planning a Press Release on your article, please get in contact with emboreports@wiley.com as early as possible, in order to coordinate publication and release dates.

Thank you again for your contribution to EMBO reports and congratulations on a successful publication. Please consider us again in the future for your most exciting work.

Yours sincerely,

Achim Breiling
Senior Editor
EMBO Reports

****

THINGS TO DO NOW:

Please note that you will be contacted by Wiley Author Services to complete licensing and payment information. The required 'Page Charges Authorization Form' is available here: https://www.embopress.org/pb-assets/embo-site/er_apc.pdf - please download and complete the form and return to embopressproduction@wiley.com

You will receive proofs by e-mail approximately 2-3 weeks after all relevant files have been sent to our Production Office; you should return your corrections within 2 days of receiving the proofs.

Please inform us if there is likely to be any difficulty in reaching you at the above address at that time. Failure to meet our deadlines may result in a delay of publication, or publication without your corrections.

All further communications concerning your paper should quote reference number EMBOR-2021-54421V4 and be addressed to emboreports@wiley.com.

Should you be planning a Press Release on your article, please get in contact with emboreports@wiley.com as early as possible, in order to coordinate publication and release dates.

EMBO Press Author Checklist

Corresponding Author Name: P Chandra Shekar	
Journal Submitted to: EMBO Reports	
Manuscript Number: EMBOR-2021-54421V2-Q	

USEFUL LINKS FOR COMPLETING THIS FORM
The EMBO Journal - Author Guidelines
EMBO Reports - Author Guidelines
Molecular Systems Biology - Author Guidelines
EMBO Molecular Medicine - Author Guidelines

Reporting Checklist for Life Science Articles (updated January 2022)
This checklist is adapted from Materials Design Analysis Reporting (MDAR) Checklist for Authors. MDAR establishes a minimum set of requirements in transparent reporting in the life sciences (see Statement of Task: 10.31222/osf.io/9sm4x). Please follow the journal's guidelines in preparing your manuscript.
Please note that a copy of this checklist will be published alongside your article.

Abridged guidelines for figures
1. Data
The data shown in figures should satisfy the following conditions:
- the data were obtained and processed according to the field's best practice and are presented to reflect the results of the experiments in an accurate and unbiased manner.
- ideally, figure panels should include only measurements that are directly comparable to each other and obtained with the same assay.
- plots include clearly labeled error bars for independent experiments and sample sizes. Unless justified, error bars should not be shown for technical replicates.
- if n<5, the individual data points from each experiment should be plotted. Any statistical test employed should be justified.
- Source Data should be included to report the data underlying figures according to the guidelines set out in the authorship guidelines on Data Presentation.

2. Captions
Each figure caption should contain the following information, for each panel where they are relevant:
- a specification of the experimental system investigated (eg cell line, species name).
- the assay(s) and method(s) used to carry out the reported observations and measurements.
- an explicit mention of the biological and chemical entity(ies) that are being measured.
- an explicit mention of the biological and chemical entity(ies) that are altered/varied/perturbed in a controlled manner.
- the exact sample size (n) for each experimental group/condition, given as a number, not a range;
- a description of the sample collection allowing the reader to understand whether the samples represent technical or biological replicates (including how many animals, litters, cultures, etc.).
- a statement of how many times the experiment shown was independently replicated in the laboratory.
- definitions of statistical methods and measures:
  - common tests, such as t-test (please specify whether paired vs. unpaired), simple χ2 tests, Wilcoxon and Mann-Whitney tests, can be unambiguously identified by name only, but more complex techniques should be described in the methods section;
  - are tests one-sided or two-sided?
  - are there adjustments for multiple comparisons?
  - exact statistical test results, e.g., P values = x but not P values < x;
  - definition of 'center values' as median or average;
  - definition of error bars as s.d. or s.e.m.

Please complete ALL of the questions below.
Select "Not Applicable" only when the requested information is not relevant for your study.

Materials

Newly Created Materials	Information included in the manuscript?	In which section is the information available? (Reagents and Tools Table, Materials and Methods, Figures, Data Availability Section)
New materials and reagents need to be available; do any restrictions apply?	Yes	Materials and methods, Appendix Figure S2 and Appendix Table S2

Antibodies	Information included in the manuscript?	In which section is the information available? (Reagents and Tools Table, Materials and Methods, Figures, Data Availability Section)
For **antibodies** provide the following information:<br>- Commercial antibodies: RRID (if possible) or supplier name, catalogue number and or/clone number<br>- Non-commercial: RRID or citation	Yes	Appendix Table S2

DNA and RNA sequences	Information included in the manuscript?	In which section is the information available? (Reagents and Tools Table, Materials and Methods, Figures, Data Availability Section)
**Short novel DNA or RNA including primers, probes**: provide the sequences.	Yes	Apeendix Table S1

Cell materials	Information included in the manuscript?	In which section is the information available? (Reagents and Tools Table, Materials and Methods, Figures, Data Availability Section)
**Cell lines:** Provide species information, strain. Provide accession number in repository **OR** supplier name, catalog number, clone number, and/**OR** RRID.	Yes	Appendix Figure S2 and Appendix Table S2
**Primary cultures:** Provide species, strain, sex of origin, genetic modification status.	Not Applicable	
Report if the cell lines were recently **authenticated** (e.g., by STR profiling) and tested for mycoplasma contamination.	Yes	Appendix Data page no 10-12

Experimental animals	Information included in the manuscript?	In which section is the information available? (Reagents and Tools Table, Materials and Methods, Figures, Data Availability Section)
**Laboratory animals or Model organisms:** Provide species, strain, sex, age, genetic modification status. Provide accession number in repository **OR** supplier name, catalog number, clone number, **OR** RRID.	Not Applicable	
**Animal observed in or captured from the field:** Provide species, sex, and age where possible.	Not Applicable	
Please detail **housing and husbandry conditions**.	Not Applicable	

Plants and microbes	Information included in the manuscript?	In which section is the information available? (Reagents and Tools Table, Materials and Methods, Figures, Data Availability Section)
**Plants:** provide species and strain, ecotype and cultivar where relevant, unique accession number if available, and source (including location for collected wild specimens).	Not Applicable	
**Microbes:** provide species and strain, unique accession number if available, and source.	Not Applicable	

Human research participants	Information included in the manuscript?	In which section is the information available? (Reagents and Tools Table, Materials and Methods, Figures, Data Availability Section)
If collected and within the bounds of privacy constraints report on age, sex and gender or ethnicity for all study participants.	Not Applicable	

Core facilities	Information included in the manuscript?	In which section is the information available? (Reagents and Tools Table, Materials and Methods, Figures, Data Availability Section)
If your work benefited from core facilities, was their service mentioned in the acknowledgments section?	Yes	Acknowledgment section of the manuscript

Design

Study protocol	Information included in the manuscript?	In which section is the information available? (Reagents and Tools Table, Materials and Methods, Figures, Data Availability Section)
If study protocol has been **pre-registered, provide DOI in the manuscript**. For clinical trials, provide the trial registration number **OR** cite DOI.	Not Applicable	
Report the **clinical trial registration number** (at ClinicalTrials.gov or equivalent), where applicable.	Not Applicable	

Laboratory protocol	Information included in the manuscript?	In which section is the information available? (Reagents and Tools Table, Materials and Methods, Figures, Data Availability Section)
Provide DOI OR other citation details if **external detailed step-by-step protocols** are available.	Yes	Materials and Methods

Experimental study design and statistics	Information included in the manuscript?	In which section is the information available? (Reagents and Tools Table, Materials and Methods, Figures, Data Availability Section)
Include a statement about **sample size** estimate even if no statistical methods were used.	Yes	Figure legends and Materials and Methods
Were any steps taken to minimize the effects of subjective bias when allocating animals/samples to treatment (e.g. **randomization procedure**)? If yes, have they been described?	Yes	Materials and Methods
Include a statement about **blinding** even if no blinding was done.	Not Applicable	No Blinding was done
Describe **inclusion/exclusion criteria** if samples or animals were excluded from the analysis. Were the criteria pre-established?<br><br>If sample or data points were omitted from analysis, report if this was due to attrition or intentional exclusion and provide justification.	Not Applicable	No samples were excluded
For every figure, are **statistical tests** justified as appropriate? Do the data meet the assumptions of the tests (e.g., normal distribution)? Describe any methods used to assess it. Is there an estimate of variation within each group of data? Is the variance similar between the groups that are being statistically compared?	Yes	Materials and Methods

Sample definition and in-laboratory replication	Information included in the manuscript?	In which section is the information available? (Reagents and Tools Table, Materials and Methods, Figures, Data Availability Section)
In the figure legends: state number of times the experiment was **replicated** in laboratory.	Yes	Figure legend
In the figure legends: define whether data describe **technical or biological replicates**.	Yes	Figure legend

Ethics

Ethics	Information included in the manuscript?	In which section is the information available? (Reagents and Tools Table, Materials and Methods, Figures, Data Availability Section)
Studies involving **human participants**: State details of **authority granting ethics approval** (IRB or equivalent committee(s), provide reference number for approval.	Not Applicable	
Studies involving **human participants**: Include a statement confirming that **informed consent** was obtained from all subjects and that the experiments conformed to the principles set out in the WMA Declaration of Helsinki and the Department of Health and Human Services Belmont Report.	Not Applicable	
Studies involving **human participants:** For publication of **patient photos**, include a statement confirming that consent to publish was obtained.	Not Applicable	
Studies involving experimental **animals**: State details of **authority granting ethics approva**l (IRB or equivalent committee(s), provide reference number for approval. Include a statement of compliance with ethical regulations.	Not Applicable	
Studies involving **specimen and field samples:** State if relevant **permits** obtained, provide details of authority approving study; if none were required, explain why.	Not Applicable	

Dual Use Research of Concern (DURC)	Information included in the manuscript?	In which section is the information available? (Reagents and Tools Table, Materials and Methods, Figures, Data Availability Section)
Could your study fall under dual use research restrictions? Please check biosecurity documents and list of **select agents and toxins** (CDC): https://www.selectagents.gov/sat/list.htm	Not Applicable	
If you used a select agent, is the security level of the lab appropriate and reported in the manuscript?	Not Applicable	
If a study is subject to dual use research of concern regulations, is the name of the **authority granting approval and reference number** for the regulatory approval provided in the manuscript?	Not Applicable	

Reporting

The MDAR framework recommends adoption of discipline-specific guidelines, established and endorsed through community initiatives. Journals have their own policy about requiring specific guidelines and recommendations to complement MDAR.

Adherence to community standards	Information included in the manuscript?	In which section is the information available? (Reagents and Tools Table, Materials and Methods, Figures, Data Availability Section)
State if relevant guidelines or checklists (e.g., **ICMJE, MIBBI, ARRIVE, PRISMA**) have been followed or provided.	Not Applicable	
For **tumor marker prognostic studies**, we recommend that you follow the **REMARK** reporting guidelines (see link list at top right). See author guidelines, under 'Reporting Guidelines'. Please confirm you have followed these guidelines.	Not Applicable	
For **phase II and III randomized controlled trials**, please refer to the **CONSORT** flow diagram (see link list at top right) and submit the CONSORT checklist (see link list at top right) with your submission. See author guidelines, under 'Reporting Guidelines'. Please confirm you have submitted this list.	Not Applicable	

Data Availability

Data availability	Information included in the manuscript?	In which section is the information available? (Reagents and Tools Table, Materials and Methods, Figures, Data Availability Section)
Have **primary datasets** been deposited according to the journal's guidelines (see 'Data Deposition' section) and the respective accession numbers provided in the Data Availability Section?	Not Applicable	No primary data sets were generated or deposited
Were **human clinical and genomic datasets** deposited in a public access-controlled repository in accordance to ethical obligations to the patients and to the applicable consent agreement?	Not Applicable	
Are **computational models** that are central and integral to a study available without restrictions in a machine-readable form? Were the relevant accession numbers or links provided?	Not Applicable	
If publicly available data were reused, provide the respective **data citations in the reference list**.	Yes	
